# The identification of BCL-XL and MCL-1 as key anti-apoptotic proteins in medulloblastoma that mediate distinct roles in chemotherapy resistance

**DOI:** 10.1038/s41419-023-06231-y

**Published:** 2023-10-28

**Authors:** Marie-Claire Fitzgerald, Philip J. O’Halloran, Sean A. Kerrane, Triona Ní Chonghaile, Niamh M. C. Connolly, Brona M. Murphy

**Affiliations:** 1 Department of Physiology & Medical Physics, Royal College of Surgeons in Ireland, 31A York Street, Dublin, D02 YN77 Ireland; 2 National Children’s Research Centre at the Children’s Health Ireland at Crumlin, Dublin, D12 N512 Ireland; 3 Department of Neurosurgery, Queen Elizabeth Hospital, Birmingham, UK; 4 Centre for Systems Medicine, Royal College of Surgeons in Ireland, 31A York Street, Dublin, D02 YN77 Ireland

**Keywords:** Cancer, Biochemistry

## Abstract

Medulloblastoma is the most common malignant paediatric brain tumour, representing 20% of all paediatric intercranial tumours. Current aggressive treatment protocols and the use of radiation therapy in particular are associated with high levels of toxicity and significant adverse effects, and long-term sequelae can be severe. Therefore, improving chemotherapy efficacy could reduce the current reliance on radiation therapy. Here, we demonstrated that systems-level analysis of basal apoptosis protein expression and their signalling interactions can differentiate between medulloblastoma cell lines that undergo apoptosis in response to chemotherapy, and those that do not. Combining computational predictions with experimental BH3 profiling, we identified a therapeutically-exploitable dependence of medulloblastoma cells on BCL-XL, and experimentally validated that BCL-XL targeting, and not targeting of BCL-2 or MCL-1, can potentiate cisplatin-induced cytotoxicity in medulloblastoma cell lines with low sensitivity to cisplatin treatment. Finally, we identified MCL-1 as an anti-apoptotic mediator whose targeting is required for BCL-XL inhibitor-induced apoptosis. Collectively, our study identifies that BCL-XL and MCL-1 are the key anti-apoptotic proteins in medulloblastoma, which mediate distinct protective roles. While BCL-XL has a first-line role in protecting cells from apoptosis basally, MCL-1 represents a second line of defence that compensates for BCL-XL upon its inhibition. We provide rationale for the further evaluation of BCL-XL and MCL-1 inhibitors in the treatment of medulloblastoma, and together with current efforts to improve the cancer-specificity of BCL-2 family inhibitors, these novel treatment strategies have the potential to improve the future clinical management of medulloblastoma.

## Introduction

Medulloblastoma is a malignant embryonal tumour of the cerebellum that primarily affects children [1]. A rare cancer, it is nevertheless the most frequently-occurring brain tumour in paediatric patients, accounting for ~20% of all paediatric intercranial tumours [2], with incidence peaking in children aged 6–8 years [1]. Overall survival rates range from 60 to 80% and are dependent on clinical factors such as patient age and disease extent [1]. Current treatment protocols for medulloblastoma incorporate surgery, chemotherapy and radiation therapy. Radiation therapy is associated with high levels of toxicity resulting in permanent neurocognitive disability and other adverse effects in up to 80% of survivors [3, 4]. Due to the heightened susceptibility of the developing brain to neurotoxicity, radiation therapy is avoided in 25–35% of patients aged <3 years [5], negatively impacting survival rates [6]. While multi-agent chemotherapy is curative for some patients, 5-year overall survival rates are highly variable and particularly poor in patients with residual tumour post-surgery, or patients with metastatic disease [7], highlighting a need for more effective therapeutic approaches.

Four molecular subgroups of medulloblastoma have been identified—Wnt, SHH, Group 3 and Group 4. These are associated with different molecular and cytogenetic features, affected demographics and metastatic potential [8–12]. The identification of subgroup-specific targets has led to the development of targeted therapies, some of which have reached clinical trial [13], while the effects of treatment de-escalation are also under investigation for patients with Wnt-subgroup medulloblastomas [1]. However, there is currently no reliable method of accurately predicting the chemotherapy responsiveness of individual medulloblastoma tumours. This highlights a need for new approaches to identify optimal treatments for individual patients. Optimising chemotherapy treatment strategies could facilitate elimination or reduction of the radiation dose and associated side effects and this would be of particular importance for younger patients who cannot receive radiation treatment.

Most chemotherapeutic agents induce death by activating the intrinsic apoptosis pathway [14]. This is regulated by the BCL-2 protein family, which comprises pro- and anti-apoptotic subgroups. Activated pro-apoptotic proteins BAX/BAK oligomerize and form pores in the mitochondrial outer membrane, releasing apoptogenic factors, including cytochrome *c* and SMAC [15]. Anti-apoptotic proteins BCL-2, BCL-XL and MCL-1 sequester BAX/BAK, preventing their activation [16]. BH3-only proteins can act directly to activate BAX/BAK, or indirectly by preventing their inhibition by anti-apoptotic proteins [17]. To this end, evasion of apoptosis is a hallmark of cancer which contributes to therapeutic resistance [18], and altered levels of various apoptosis signalling proteins have been associated with treatment responsiveness in many cancers [19]. As this includes frequent over-expression of anti-apoptotic proteins, the application of BH3 mimetics is a rational approach to sensitise cells to chemotherapy, although little work has been carried out to delineate their potential in medulloblastoma.

As apoptosis activation depends on the interaction of multiple proteins within a signalling network, several systems-based approaches have been applied to holistically investigate the dependence of cancer cells on apoptosis signalling [20]. Statistical modelling approaches demonstrated that integrated analysis of apoptosis protein expression levels could accurately predict the chemosensitivity of a variety of glioblastoma and melanoma cell lines [21–23]. Furthermore, application of ordinary differential equation (ODE)-based models of apoptosis signalling kinetics has also successfully predicted the chemotherapy responsiveness of both cell lines and patient biopsies [20, 24–27].

Here, we combined systems biology and experimental approaches and showed that analysis of basal apoptosis protein signatures is predictive of cisplatin responsiveness in medulloblastoma cell lines. Secondly, we identified BCL-XL as a key anti-apoptotic protein, whose inhibition efficiently potentiated cisplatin-induced cytotoxicity. Finally, we identified MCL-1 as a second critical anti-apoptotic mediator whose targeting in combination with BCL-XL inhibition can trigger apoptosis in the absence of an upstream cytotoxic stimulus.

## Materials and methods

### Cell lines and cell culture

Human medulloblastoma cell lines Daoy, D283-Med, CHLA-01-Med and CHLA-01R-Med, and the cervical cancer cell line HeLa were obtained from American Type Cell Culture (ATCC, Rockville, MD, US). Medulloblastoma lines ONS76 and UW228 lines were kindly provided by Dr. Till Milde, German Cancer Research Centre (DKFZ), and D425-Med and D458-Med lines were received from Dr. Darell Bigner, Preston Robert Tisch Brain Tumor Center.

HeLa, Daoy, ONS76 and UW228 cell lines were grown as a monolayer in DMEM (Lonza, Lisburn, UK) with 10% heat-inactivated foetal bovine serum (FBS), 100 U/mL penicillin, and 100 mg/mL streptomycin (Sigma-Aldrich, Arklow, Ireland). D425-Med and D458-Med cell lines were maintained as a cell suspension in DMEM-F12 (Lonza) with 10% heat-inactivated FBS, 100 U/mL penicillin, and 100 mg/mL streptomycin. CHLA-01-Med and CHLA-01R-Med cell lines were cultured in DMEM-F12 medium containing B27 supplement 50X (2%) (Gibco Life Technologies, Dún Laoghaire, Ireland), human bFGF (20 ng/mL), human EGF (20 ng/mL) (PeproTech EC Ltd, London, UK), penicillin (100 U/mL), and streptomycin (100 mg/mL) (Sigma-Aldrich). D283-Med cells were grown in suspension in EMEM (ATCC) supplemented with 10% heat-inactivated FBS, 100 U/mL penicillin and 100 U/mL streptomycin. All cell lines were maintained in a humidified incubator at 37 °C and 5% CO_2_. Cells were routinely tested for mycoplasma infection. Cell counts were determined using a Countess automated cell counter (Thermo Fisher Scientific, Waltham, MA, US).

### Drug and inhibitor preparation

Cisplatin was purchased from Abcam (Cambridge, UK) and reconstituted in 0.9% w/v NaCl. All other agents were reconstituted in DMSO. ABT-199 was purchased from Active Biochem (Hong Kong, China) and S-63845, WEHI-539 and Q-VD-OPh from Medchemexpress (NJ, US).

### WST-1 cell viability assay

Daoy, ONS76 and UW228 cells were seeded as adherent cells in a 96-well plate at a density of 5000 cells/well, while D425-Med, D458-Med, CHLA-01-Med, CHLA-01R-Med and D283-Med cells were seeded in 96-well plates (10,000 cells/well) rinsed with anti-adherence rinsing solution (StemCell Technologies, Cambridge, UK). Following a 24 h incubation period, cells were treated as indicated. Following treatment, WST-1 reagent (Sigma-Aldrich) was added to a final dilution of 1:10, per manufacturer’s directions. Cells were incubated for a further 3 h to allow the colorimetric WST-1 reaction to proceed. The absorbance in each well was measured at 450 nm using a microplate reader (GENios, Tecan, Weymouth, UK). The background signal (620 nm) was subtracted and mean absorbance values for each condition were normalised to vehicle-treated controls. IC50 values were calculated using nonlinear regression in GraphPad Prism software version 8.3.4 (GraphPad Software Inc., La Jolla, CA, US), and drug synergy was calculated using Webb’s fractional product method [28]. A synergy score of 1 indicated an additive effect, while values > 1 indicated antagonism. Values < 0.8 were indicative of strong synergy and values 0.8–1 were considered weakly synergistic.

### Apoptosis detection using Annexin-V/PI

Cells were seeded at a density of 50,000 cells/well in a 24-well plate and incubated for 24 h before treatment. Where Q-VD-OPh was used to assess the requirement for caspase activity in cell death, cells were pre-treated with 30 µM Q-VD-OPh for 30 min before additional agents were applied. Unless otherwise stated, Daoy, ONS76, UW228, D425-Med and D458-Med cells were treated for 48 h, and D283-Med cells for 72 h, due to their increased doubling time. Following treatment, adherent cells were detached using Trypsin-EDTA (Sigma-Aldrich), pelleted, and washed, while suspension cells were pelleted, washed and clusters were manually dissociated using a pipette. Cells were incubated in 100 µL Annexin-V binding buffer (10 nM HEPES, 135 nM NaCl, 5 mM CaCl_2_, pH 7.4) containing Annexin-V-FITC conjugate (1:200) (BioVision, Mountain View, CA, USA) and propidium iodide (1:500) (Sigma-Aldrich) for 20 min in the dark. Cells were analysed using a BD LSRII flow cytometer (BD Biosciences, Oxford, UK), and acquired data was analysed using Flowing Software Version 2.5.1 (Turku Bioscience, Tykisokatu 6, FI-20520 Turku, Finland).

### Plate-based BH3 profiling assay

The plate-based BH3 profiling assay was carried out using whole cells and JC-1 fluorimetry, as previously described [29]. BH3 peptides were diluted at 2× their desired concentration in DTEB buffer (300 mM trehalose, 10 mM HEPES-KOH, 80 mM KCl, 1 mM EGTA, 1 mM EDTA, 0.1% BSA, 5 mM succinate, pH 7.4) and 15 µL/well was plated in a black 384-well plate in triplicate. Responses to individual peptides provide insight into the anti-apoptotic dependencies of individual cell lines [29]. Cells were collected, washed in PBS, counted, re-suspended in DTEB buffer, and combined with a pre-prepared mix of JC1 dye and digitonin (1 µM JC-1, 0.005% digitonin, 10 µg/µL oligomycin and 5 mM β-mercaptoethanol in DTEB buffer). Following a 10 min incubation at room temperature in the dark, 15 µL containing 30,000 viable cells was added to the 2X BH3 peptides per well of the 384-well plate. The resulting loss of mitochondrial potential was measured using the Varioskan LUX multimode microplate reader (Thermo Fisher Scientific). Measurements were taken every 5 min over 3 h, at an excitation wavelength of 535 nm, and an emission wavelength of 590 nm. The percentage mitochondrial depolarisation induced for each condition was normalised to a DMSO control (0%) and FCCP (carbonyl cyanide 4- (trifluoromethoxy) phenylhydrazone) (100%).

### Dynamic BH3 profiling

Cells were treated with cisplatin (20 µM) or WEHI-539 (1 µM) for 18 h, and then BH3 profiling was performed on viable cells as described in BH3 profiling above.

### Western blotting

Whole-cell lysates were prepared using RIPA lysis buffer (50 mM Tris, 150 mM NaCl, 0.1% sodium dodecyl sulfate, 0.5% sodium deoxycholate, 0.1% Triton X-100) to which protease/phosphatase inhibitor cocktails (Sigma-Aldrich) were freshly added in a 1:100 ratio. Protein concentrations were determined using a BCA protein assay kit (Pierce, Rockford, IL, US) per manufacturer’s instructions. Lysate samples containing 20 μg of protein were prepared with Laemmli loading buffer and separated on 12–15% SDS-polyacrylamide gels and transferred to 0.2 µM nitrocellulose membranes using wet transfer.

Membranes were probed overnight at 4 °C with the following primary antibodies at the indicated concentrations: APAF-1 (#611364, 1:500), BAK (#556382, 1:250), BAX (#556467, 1:250), BCL-2 (#610537, 1:500), BID (#611528, 1:500), caspase 3 (#610322, 1:1000), XIAP (#610716, 1:1000) (BD Biosciences, Oxford, UK), BCL-XL (#2764s, 1:1000), BIM (#2933s, 1:1000), caspase 7 (#9592, 1:1000), caspase 9 (#9508s, 1:1000), cleaved caspase 3 (#9661s, 1:1 000), cleaved caspase 7 (9491s, 1:1000), cleaved caspase 9 (#9501, 1:1000), MCL-1 (#94296s, 1:1000), NOXA (#14766s, 1:250), PUMA (#12450s, 1:500), SMAC (#29545, 1:1000) (Cell Signaling, Danvers, MA, US) and GAPDH (#MAB374, 1:5000, Merck KGaA, Darmstadt, Germany). Membranes were next incubated with mouse (#AP124P, 1:5000) or rabbit (#AP132P, 1:5000) (Merck KGaA, Darmstadt, Germany) horseradish peroxidase-conjugated secondary antibodies and protein bands were visualised using Supersignal West Pico Chemiluminescent Substrate (Pierce). Images were captured using Fuji-film LAS-4000 (Fuji, Sheffield, UK), and densitometry analysis was carried out using Image Studio Lite v5.2 (LI-COR Biosciences Ltd., UK). For quantification of western blots involving all cell lines, Ponceau-S (Sigma-Aldrich) staining was used as a total protein stain due to the unequal expression of standard housekeeping proteins between adherent and suspension cell cultures. HeLa cell lines were used for normalisation to enable the relative and absolute quantification of medulloblastoma protein levels used as input to the systems modelling platforms.

Quantifications were carried out on at least *N* = 3 independent experiments. For clustering analysis of protein expression data, the log2 of protein expression levels relative to HeLa were z-score normalised across proteins and hierarchical clustering was performed using the ComplexHeatmap function [30] in R version 4.1.3 [31]. Code and input files are available at https://github.com/niamhconno/Fitzgerald-et-al-2023.

### Gene knockdown using siRNA transfection

Cells were transiently transfected with control or targeting siRNA using Lipofectamine 3000 reagent (Thermo Fisher Scientific) per manufacturer’s instructions. Cells were seeded at a density of 50,000 cells/well in a 24-well plate and allowed to adhere overnight. Culture media was replaced with OPTI-MEM (Gibco Life Technologies) and cells were transfected with Silencer Select siRNA (Thermo Fisher Scientific) as follows: Control siRNA (#4390843), BCL2L1 (BCL-XL) siRNA (#s1920, 50 nM for 48 h) and MCL-1 siRNA (#s8583, 20 nM for 48 h). Control siRNA was used at the same concentration and incubation time as the targeted siRNA. 24 h post-transfection, OPTI-MEM was replaced with regular cell culture media as described above. Gene knockdown was verified by Western blotting. Transfected cells were treated as indicated following siRNA incubation and effects on apoptosis were determined using Annexin-V/PI staining.

### Co-immunoprecipitation of BCL-XL

The Pierce^TM^ Crosslink IP kit (Thermo Fisher Scientific) was used to crosslink the BCL-XL antibody to 20 µL Protein A/G affinity beads. Beads were washed with 1X coupling buffer before incubation with end-over-end mixing at room temperature for 1 h with 3 µg BCL-XL antibody or rabbit IgG (#2729 s, Cell Signaling) dissolved in 1X coupling buffer. Bound antibodies were cross-linked to the Protein A/G beads using disuccinimidyl suberate by end-over-end mixing for 1 h at room temperature. Columns were centrifuged and washed with elution buffer and IP lysis/wash buffer, and stored at 4 °C. For co-immunoprecipitation, cells were treated as indicated in figure legends and lysed in RIPA lysis buffer. 500 µg of cell lysate was pre-cleared using control agarose beads for 1 h with end-over-end mixing, and the pre-cleared lysate was then incubated with the cross-linked antibody overnight at 4 °C. Bound protein complexes were eluted from the columns using elution buffer and precipitates were combined with 6X Laemlli buffer, boiled for 5 min at 95 °C and analysed via western blotting. Membranes were probed for BCL-XL, BIM, BAX, BAK and PUMA, with GAPDH as a loading control.

### Statistical analysis

Statistical analyses were performed using GraphPad Prism software version 8.4.3. Results are presented as mean ± SEM. Unless stated otherwise, all data presented are the mean of three independent experiments. Data were tested for significance using appropriate statistical tests as described in figure legends and text (* denotes *p* < 0.05, ** denotes *p* < 0.01, *** denotes *p* < 0.001, **** denotes *p* < 0.0001 and are considered statistically significant; ns = not significant).

### Calculation of functional groups and principal component analysis

Principal component analysis was performed using a customised version of a previously developed pipeline [23] in MATLAB 2019a (Mathworks, UK). Mean protein levels relative to HeLa cells were determined and functional groups were calculated by combining individual protein expressions using arithmetic operations according to their protein interactions [21, 22]. The relative expressions of BAX and BAK were summed (BAX + BAK), as were the relative expression of BCL-2, BCL-XL and MCL-1 (BCL-2 + BCL-XL + MCL-1), as these proteins mediate similar functions. APAF-1 was multiplied by pro-caspase 9 (APAF*C9), representing apoptosome formation, while XIAP levels were divided by pro-caspase 3 (XIAP/C3) to reflect the inhibitory effect of XIAP on caspase activation. SMAC, BID, BIM, PUMA and NOXA were left as independent variables. Functional group data were z-score normalised, and Principal component analysis (PCA; pca function) was performed. Principal components (PCs) with eigenvalues > 1 [3] were retained for subsequent analysis according to the Kaiser criterion [32]. Linear Discriminant Analysis (LDA; classify function) enabled determination of a plane of separation between cell lines with high and low cisplatin sensitivity. Code and input files are available at https://github.com/niamhconno/Fitzgerald-et-al-2023.

### Patient dataset predictions

mRNA expression levels of the 14 apoptosis proteins and patient characteristics were downloaded from a publicly-available dataset [33] through the open-access GlioVis portal [34]. As we focussed on paediatric medulloblastoma, patients aged >18 or whose age was unknown were excluded. mRNA expression profiles were used to estimate protein expression profiles, expanding on a previously-published approach [23]. mRNA expression values within their interquartile regions were mapped to the corresponding interquartile ranges of the cell-line protein expression values quantified via Western blot. Functional groups were calculated from inferred protein expressions, and patient samples were placed onto the previously-determined PC space by normalising each functional group value to the cell line z-score normalised data and multiplying by PC coefficients to determine their position in the PC space. LDA was performed as above to classify the patient samples. Expression values and code are available at https://github.com/niamhconno/Fitzgerald-et-al-2023.

### DR_MOMP

DR_MOMP is an ODE-based model of BCL-2 protein interactions that calculates a numeric score (*η*) that reflects the stress dose required to induce MOMP in cancer cells. A complete description of DR_MOMP can be found in ref. [25]. The cell line protein expression levels of BCL-2, BCL-XL, MCL-1, BAX and BAK were used as input to the model. The effects of in silico BH3 mimetic administration were also investigated. Binding affinities of the BH3 mimetics ABT-199, WEHI-539 and S63845 were implemented in DR_MOMP as previously described [25, 27], based on their published dissociation constants.

### APOPTO-CELL

APOPTO-CELL is an ODE-based model of apoptosome-dependent caspase activation resulting in apoptosis [35]. It determines the kinetics of apoptosis execution based on input concentrations of APAF-1, caspase 9, caspase 3, SMAC and XIAP. Protein concentrations were determined by converting relative protein concentrations using previously determined HeLa concentrations [20]. APOPTO-CELL determines substrate cleavage by caspase-3/-7 over time, and cell lines are defined as apoptosis competent if 80% substrate cleavage is reached within 60 min [20].

## Results

### Medulloblastoma cell lines respond heterogeneously to cisplatin treatment and express different levels of apoptosis signalling proteins

**Fig. 1:**
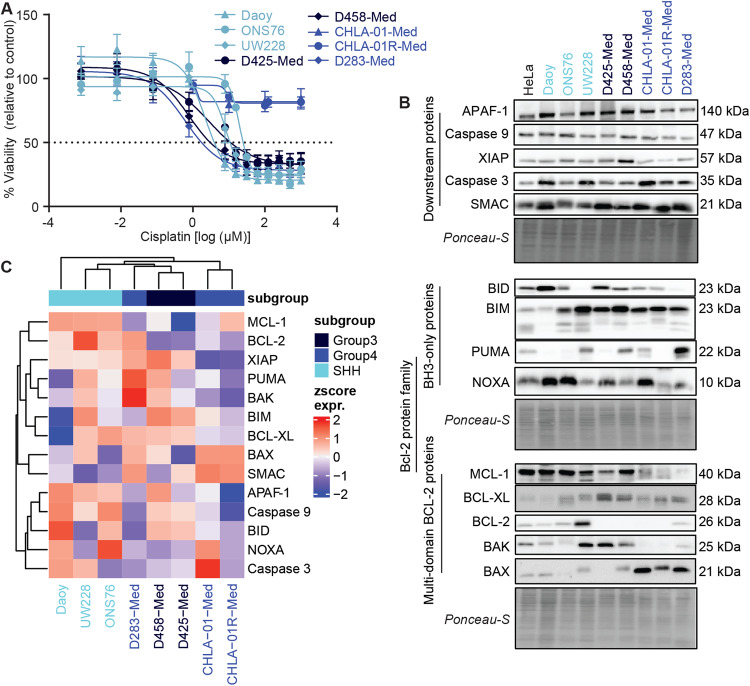
Medulloblastoma cell lines differ in their expression of apoptosis proteins and in their cisplatin responsiveness. **A** Medulloblastoma cell lines were treated with increasing concentrations of cisplatin for 48 h (Daoy, ONS76, UW228, D425-Med, D458-Med) or 72 h (CHLA-01-Med, CHLA-01R-Med, D283-Med), and cell viability was measured using the WST-1 viability assay. Data are expressed as mean ± SEM of *N* = 3 independent experiments performed in duplicate. **B** Expression levels of apoptosis signalling proteins were determined in the eight medulloblastoma lines. Medulloblastoma whole-cell lysates were run alongside a HeLa whole-cell lysate to enable calculation of expression levels relative to HeLa cell expression. Blots shown are representative of at least *N* = 4 independent experiments. Ponceau-S staining was used as a loading control. Relative quantifications of protein expression are shown in Supplementary Table 1. **C** Hierarchical clustering of apoptosis protein expression in all medulloblastoma cell lines. Log2 expression levels (relative to HeLa) were z-score normalised across proteins.

We studied a panel of eight MB cell lines in this study (Fig. 1A, Table 1), reflecting three main medulloblastoma subgroups (SHH, Group 3 and Group 4) and of varying MYC and p53 mutational status. These cell lines have been previously molecularly characterised and were chosen on the basis that they represent well-established examples of their molecular subgroup [36, 37]. We first characterised cell line sensitivity to cisplatin, a commonly used chemotherapy in medulloblastoma treatment. Following treatment, cell viability was determined using the Wst-1 viability assay. A range of responses were observed (Fig. 1A, Table 1) with D283-Med displaying the highest cisplatin sensitivity (IC50 = 0.61 ± 1.52 µM), Daoy, D425-Med, D458-Med and UW228 demonstrating IC50 values < 10 µM, and the two group four cell lines CHLA-01-Med and CHLA-01R-Med displaying a cisplatin-resistant phenotype.

**Table 1:** Summarised characteristics of cell lines.

Cell line	Subgroup	Patient details	P53 status	MYC status (amplification)	Cisplatin IC50 (µM)
Daoy	SHH	4 year old Male	Mt	No	2.26 ± 1.23
ONS76	SHH	2 year old Female	Wt	No	19.77 ± 1.12
UW228	SHH	9 year old Female	Mt	No	8.46 ± 1.15
D425-Med	Group 3	5 year old Male	Mt	Yes	2.71 ± 1.53
D458-Med	Group 3	5 year old Male	Wt	Yes	0.77 ± 1.48
CHLA-01-Med	Group 4	8 year old Male	Mt	Yes	>1000
CHLA-01R-Med	Group 4	8 year old Male	Wt	Yes	>1000
D283-Med	Group 4	6 year old Male	Wt	Yes	0.61 ± 1.52

As cisplatin induces apoptosis via the intrinsic pathway, we were interested in determining whether differences in the expression of apoptosis signalling proteins were associated with cisplatin response. We measured the basal levels of 14 regulatory proteins in the pathway (APAF-1, BAK, BAX, BCL-XL, BCL-2, BID, BIM, MCL-1, NOXA, pro-caspase 3, pro-caspase 9, PUMA, SMAC and XIAP), and quantified their expression relative to that of HeLa cells and found heterogeneous expression across the cell line panel (Fig. 1B). BAX and SMAC were most highly expressed in Group 4 medulloblastoma cell lines compared with the SHH and Group 3 subgroups, while pro-caspase 9 was more highly expressed in the SHH subgroup, compared to Group 4 cell lines (Supplementary Fig. 1A).

Unsupervised clustering of protein expression segregated the cisplatin-resistant pair of cell lines [the primary CHLA-01-Med and recurrent CHLA-01R-Med] from the rest of the panel, where most proteins were downregulated compared to other cell lines (Fig. 1C). Interestingly, these cell lines were characterised by significantly higher levels of BAX and SMAC, and significantly lower levels of XIAP (Supplementary Fig. 1B). These findings were somewhat counter-intuitive, given the known pro-apoptotic roles of BAX and SMAC [38, 39], while XIAP is an anti-apoptotic protein associated with chemoresistance in many cancers [40]. Given their low expression profiles of most of the key apoptotic proteins, we excluded this cell-line pair, which are derived from the same patient, from our subsequent investigations of apoptosis-related responses.

### Systems modelling identifies signalling upstream of MOMP as a key determinant of cisplatin sensitivity

**Fig. 2:**
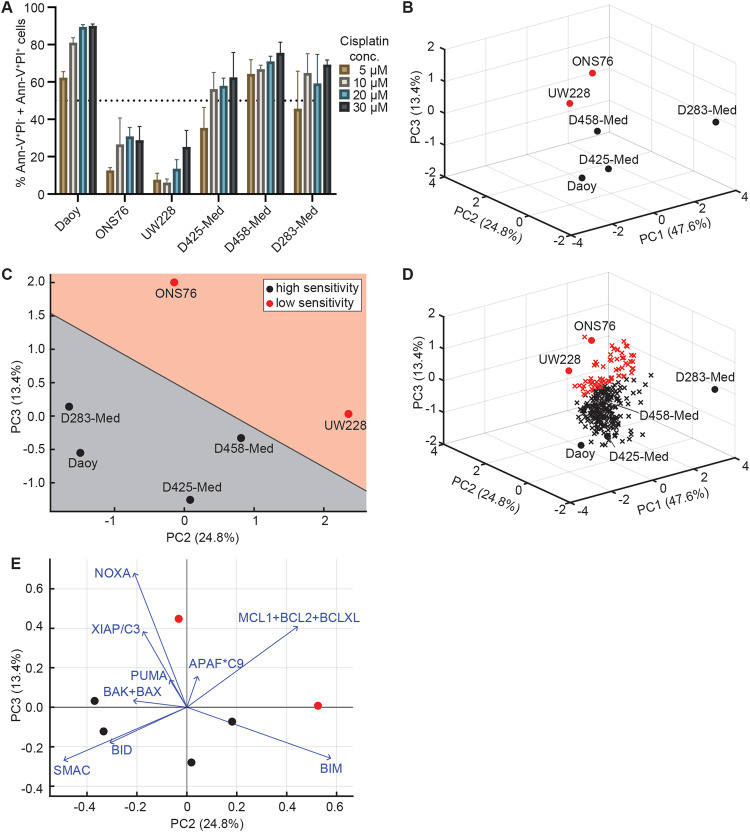
Principal Component Analysis (PCA) of apoptosis signalling proteins separates medulloblastoma cell lines according to their cisplatin sensitivity. **A** Flow cytometry was used to assess the number of AnnexinV+PI- + AnnexinV+PI+ cells following treatment with the indicated concentrations of cisplatin. Data are expressed as mean of *N* = 3 independent experiments, ±SEM. **B** PCA was carried out on functional group data representing the expression profiles of individual medulloblastoma cell lines. Cell lines are positioned in the 3D space defined by the first three PCs. Colour coding indicates cell line responsiveness to cisplatin, where black indicates high cisplatin sensitivity, and red indicates reduced sensitivity. **C** Following PCA, linear discriminant analysis (LDA) was carried out to segment the PC space into areas corresponding to cisplatin sensitivity. The 2D plot shows cell line distribution in the 2nd and 3rd PCs. **D** Estimated protein expression profiles for medulloblastoma tumours were generated and samples were positioned into the previously generated PC space. Samples are colour-coded by their LDA-predicted sensitivity to cisplatin-induced apoptosis. 199/266 samples were predicted to be highly sensitive (black) and 67/266 were predicted to have reduced sensitivity (red). **E** The same data as (**C**) overlaid on a biplot showing the contributions of each functional group to the variance accumulated in the 2nd and 3rd PCs. Scores and coefficients (and therefore axes) are scaled according to the MATLAB biplot function.

To investigate the apoptosis response of cell lines that demonstrated a response to cisplatin in the cell viability assay, we carried out Annexin-V/PI staining. This identified that, while four cell lines (Daoy, D425-Med, D458-Med and D283-Med) underwent apoptosis at clinically-relevant cisplatin concentrations (10–20 µM) [41], ONS76 and UW228 exhibited lower levels of apoptotic death even at the highest drug concentration (30 µM, Fig. 2A), in line with their higher IC50 values in the viability assay (Fig. 1B). Given that the UW228 and ONS76 protein expression profiles also clustered together (Fig. 1C), this suggested that differential apoptosis protein expression may contribute to the varied response to cisplatin. However, as the relative expression of individual proteins did not correlate with death induced by 20 µM cisplatin (Supplementary Table 2), we hypothesised that differences in apoptosis response may be explained by the signalling interactions required between these proteins for successful apoptosis execution.

We, therefore, performed systems-level analyses of the 14 quantified apoptotic proteins. We first applied a PCA-based method that integrates apoptosis pathway-level knowledge by calculating functional groups using arithmetic operations representing protein interactions [21] (Fig. 2B). This approach previously predicted the response of glioblastoma and melanoma cell lines to both genotoxic chemotherapy (temozolomide and dacarbazine, respectively) and TRAIL-induced apoptosis [21–23]. The first three PCs explained 85.8% of the variance in the functional groups. Interestingly, while the first two PCs alone were insufficient to explain the differences in cisplatin-sensitivity, ONS76 and UW228 were clearly separated from the other cell lines by PC2 and PC3, as confirmed by LDA (Fig. 2C). This suggests that the response of cell lines to cisplatin can be determined based on functional integration of basal apoptosis protein expression profiles.

Having identified that functional grouping of apoptosis regulatory proteins provided power to predict cisplatin sensitivity, we were interested in exploring what proportion of patient tumours may be predicted to be chemosensitive. Using publicly available mRNA measurements from 266 medulloblastoma tumour samples [33], we generated protein expression profiles by mapping mRNA expression values onto cell line protein expression data (see Methods) and positioned patient samples onto the segmented PC space (Fig. 2D). This suggested that a significant proportion of tumours (199/266, 74.8%) may be sensitive to cisplatin-induced apoptosis. However, we note that additional in vivo factors such as blood-brain barrier permeability [42] would influence the patient’s response to treatment.

Next, we were interested in identifying which functional groups contributed most to the separation of the cell lines. Visualising the magnitude and sign of each functional group’s contribution to the second and third PCs (Fig. 2E) demonstrated that the ‘SMAC’, ‘BID’ and ‘BCL-2 + BCL-XL + MCL-1’ groups contributed most strongly to the linear separation of ONS76 and UW228, suggesting that reduced levels of the pro-apoptotic proteins SMAC or BID, and increased expression of the anti-apoptotic proteins may contribute to the decreased cisplatin sensitivity of ONS76 and UW228.

**Fig. 3:**
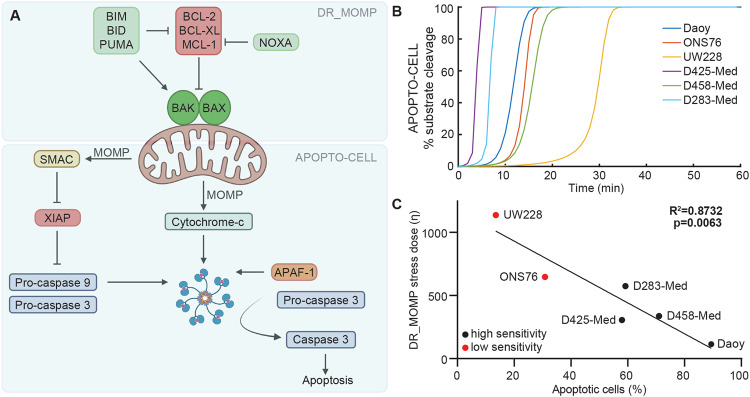
Ordinary differential equation (ODE-) modelling of apoptosis signalling upstream of MOMP predicts cisplatin sensitivity. **A** Schematic representation of the apoptosis pathway highlighting the parts of the pathway modelled by DR_MOMP (upstream of MOMP) and APOPTO-CELL (downstream of MOMP). Created with BioRender.com. **B** APOPTO-CELL models the % caspase-3/-7-mediated substrate cleavage over time-based on basal levels of APAF-1, caspase 3, caspase 9, SMAC and XIAP. Cell lines are defined as capable to execute apoptosis if 80% substrate cleavage is achieved within 60 min of cytochrome-c release (time 0 on graph). **C** The DR_MOMP η score was calculated based on basal levels of BAX, BAK, BCL-2, BCL-XL and MCL-1 and this was correlated with the proportion of experimentally measured apoptotic cells following cisplatin treatment (Fig. 2A). Correlation was assessed using Pearson’s correlation coefficient.

Therefore, as we identified both downstream (SMAC) and upstream (BCL-2 + BCL-XL + MCL-1 and BID) proteins as determinants of cisplatin sensitivity, we applied two ODE-based computational models of the downstream (APOPTO-CELL) and upstream (DR_MOMP) apoptosis pathways to investigate the mechanisms underlying these differential responses (Fig. 3A). APOPTO-CELL simulates the kinetics of apoptosome-dependent activation of executioner caspases following SMAC and cytochrome-c release from the mitochondria and their subsequent substrate cleavage over time [20, 24, 35]. APOPTO-CELL predicted that all medulloblastoma cell lines are competent to undergo apoptosis following MOMP, as they exceeded 80% substrate cleavage within 60 min of MOMP (Fig. 3B). This suggests that expression differences in downstream proteins do not explain the observed differences in cisplatin sensitivity. DR_MOMP determines apoptosis sensitivity by calculating a ‘stress dose’ (*η*) representing the amount of stress-induced BH3-only proteins required to permeabilize the mitochondrial outer membrane [25]. DR_MOMP predicted that UW228 and ONS76 required a higher stress dose to execute apoptosis and the simulated stress dose for all six cell lines correlated with the proportion of apoptotic cells experimentally measured following treatment with 20 µM cisplatin (*p* = 0.0063) (Fig. 3C). This suggested that signalling interactions upstream of MOMP may determine cisplatin-induced apoptosis sensitivity, and highlights this as a potential therapeutic target.

### BCL-XL is a key anti-apoptotic mediator in medulloblastoma whose targeting sensitises cells to cisplatin treatment

**Fig. 4:**
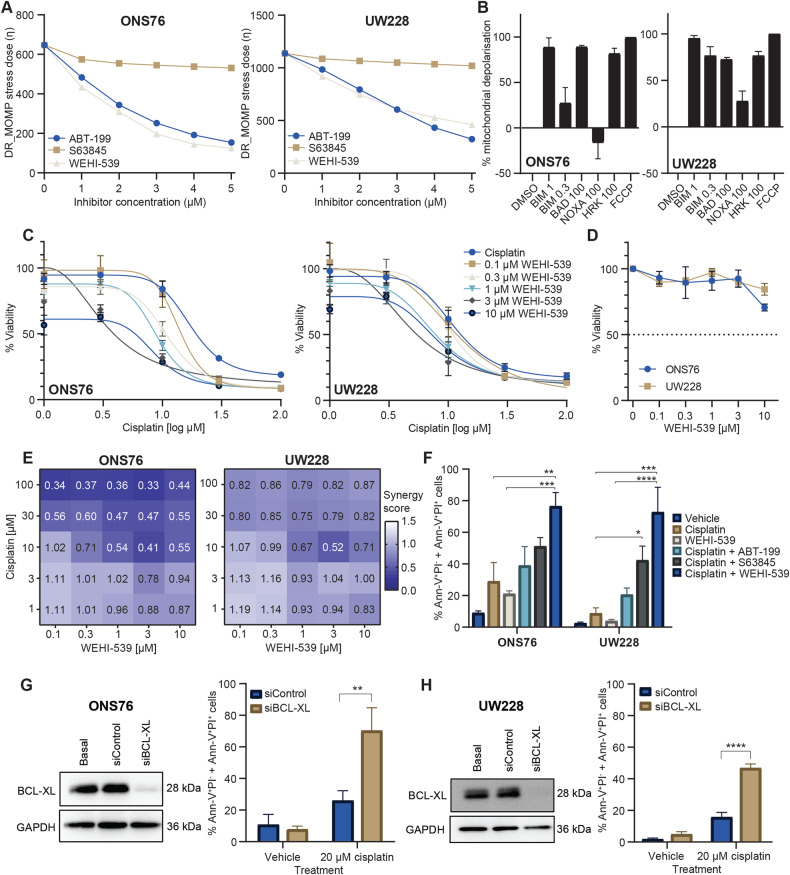
Inhibiting BCL-XL sensitises medulloblastoma cells to the apoptosis-inducing effects of cisplatin treatment. **A** DR_MOMP calculated the *η* score for ONS76 and UW228 cell lines following in silico administration of the indicated concentrations of ABT-199, S63845 or WEHI-539. **B** BH3 profiling was carried out to determine the basal anti-apoptotic dependencies of ONS76 (left) and UW228 (right) cell lines using the indicated BH3 peptides. Briefly, BAD targets BCL-2 and BCL-XL, HRK targets BCL-XL, and NOXA and A12 are specific for MCL-1. BIM is a measure of overall priming as it promiscuously interacts with all anti-apoptotic proteins. Data are expressed as the mean ± SEM of *N* = 3 independent experiments performed in triplicate, normalised to DMSO (0% mitochondrial depolarisation) and FCCP (100% mitochondrial depolarisation). **C** Cell viability in ONS76 (left) and UW228 (right) was measured following 48 h treatment with increasing concentrations of cisplatin combined with the indicated concentrations of WEHI-539 using the WST-1 viability assay. Data are expressed as mean ± SEM of *N* = 3 independent experiments performed in duplicate. **D** ONS76 and UW228 cells were treated with the indicated concentrations of WEHI-539 for 48 h and viability was determined using the Wst-1 viability assay. Data points represent the mean ± SEM of 3 independent experiments carried out in duplicate. The *x*-axis is not continuous. **E** Combination index values were calculated based on the above data (**C**) to assess synergy between cisplatin and WEHI-539 in ONS76 (left) and UW228 (right) cells. A score <1 indicates synergy between the two agents, while <0.8 indicates strong synergy. **F** Flow cytometry was used to assess the number of AnnexinV+PI- + AnnexinV+PI+ cells following 48 h treatment with cisplatin (20 µM), or cisplatin with BH3 mimetics (1 µM). The effect of treatment with WEHI-539 (1 µM) alone was also investigated. Data are expressed as mean of *N* = 3 independent experiments, ±SEM. Two-way ANOVA followed by Tukey’s multiple comparisons test was used for statistical analysis, whereby *p < 0.05, **p < 0.01, ***p < 0.001, ****p < 0.0001. ONS76 (**G**) and UW228 (**H**) cells were transiently transfected with non-targeting control siRNA (siControl) or targeted siRNA against BCL-XL (siBCL-XL). BCL-XL protein expression was assessed with Western blotting 48 h post-transfection to verify successful knockdown, with GAPDH used as a loading control. Representative blots of *N* = 3 independent experiments are shown. Cells were subsequently treated with cisplatin (20 µM) for a further 24 h, and Annexin-V/PI staining followed by flow cytometry analysis was used to determine the proportion of apoptotic cells. Data are expressed as mean of *N* = 3 independent experiments ±SEM. Data were analysed using One-Way ANOVA with Tukey’s multiple comparison test, whereby **p < 0.01, ****p < 0.0001.

Collating PCA and DR_MOMP findings suggested that the BCL-2 proteins play a key role in mediating cisplatin sensitivity in medulloblastoma cells. We applied DR_MOMP to predict whether BCL-2 family inhibitor could sensitise the ONS76 and UW228 cell lines to cisplatin treatment, and identified that inhibitors of BCL-XL (WEHI-539) and BCL-2 (ABT-199) were predicted to most strongly reduce the stress dose required to induce apoptosis in both cell lines (Fig. 4A).

We next employed BH3 profiling as an in vitro technique to determine the cell lines’ anti-apoptotic dependencies. We observed that HRK (BCL-XL specific) and BAD (BCL-2 and BCL-XL specific) both induced ~60–85% mitochondrial depolarisation compared with the positive control, FCCP (Fig. 4B). This suggested that the depolarising effects of BAD were due to its BCL-XL- rather than its BCL-2-inhibitory role, and that both cell lines were BCL-XL dependent. Meanwhile, the response to the NOXA peptide suggested a less significant role for MCL-1 in UW228 cells (Fig. 4B). Taken together, our data indicated that the cell lines are mainly dependent on BCL-XL for survival. To validate these predictions, we combined cisplatin with ABT-199, S63846 and WEHI-539 (inhibitors of BCL-2, MCL-1 and BCL-XL, respectively). In agreement with predictions, combining WEHI-539 with cisplatin reduced viability in both cell lines compared to cisplatin treatment alone (Fig. 4C), though WEHI-539 had little effect as a single agent (Fig. 4D). Nevertheless, the combination yielded synergistic effects at therapeutically relevant cisplatin concentrations (Fig. 4E, values < 0.8). Combined treatment of WEHI-539 (1 µM) and cisplatin (20 µM) also significantly increased levels of apoptotic cells compared to that induced by cisplatin alone (Fig. 4F), where again, WEHI-539 had little effect as a single agent. Addition of ABT-199 or S63845 to cisplatin did not yield such pronounced effects (Fig. 4F, Supplementary Fig. 2), although S63845 potentiated cisplatin cytotoxicity in UW228 cells, in agreement with BH3 profiling predictions. In parallel, we used an siRNA-mediated approach to knock down BCL-XL expression, and show that BCL-XL silencing resulted in a significant enhancement of cisplatin-induced apoptosis compared with control siRNA (Fig. 4G, H). This is in line with the effect of pharmacologically targeting BCL-XL, and demonstrates a functional role for BCL-XL in mediating protection against cisplatin-induced apoptosis. Finally, BH3 profiling analysis of the cisplatin-sensitive cell lines, D425-Med and D283-Med, also showed BCL-XL dependence. Here cisplatin and WEHI-539 elicited some weak synergistic effects on cell viability, due to the higher activity of the individual agents (Supplementary Fig. 3), while the addition of WEHI-539 to cisplatin significantly increased the levels of apoptotic cells. Collectively, these experiments identified BCL-XL as a target to sensitise cisplatin-resistant medulloblastoma cells to treatment, and to further increase the responsiveness of cisplatin-sensitive cells.

### Cisplatin alters BCL-2 family expression and resistance is overcome by BCL-XL inhibition

**Fig. 5:**
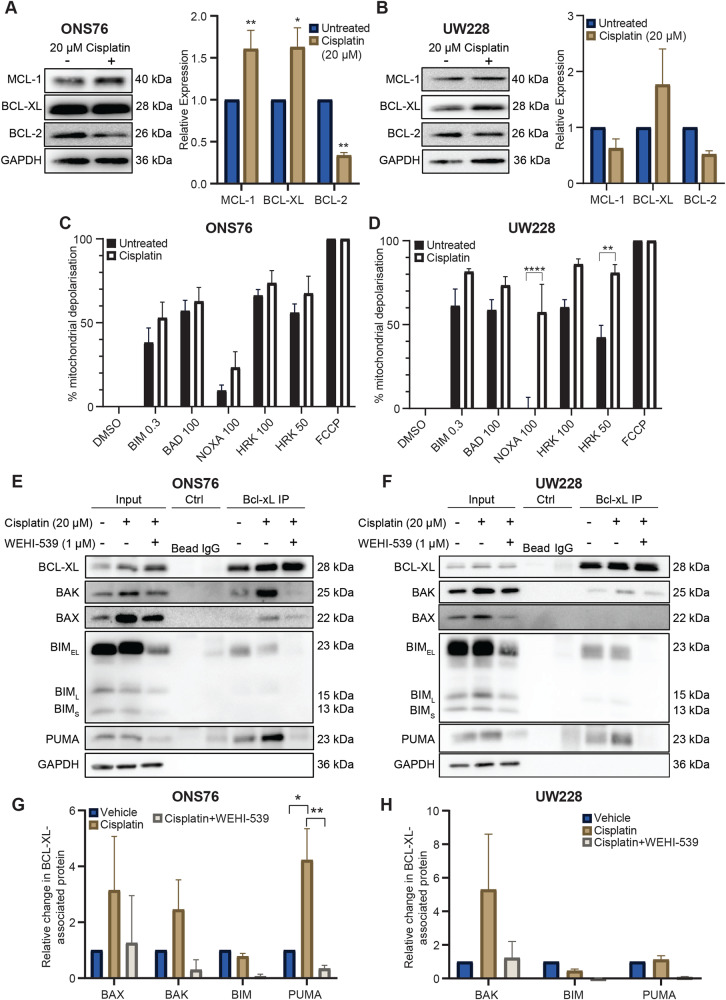
Cisplatin treatment alters anti-apoptotic protein expression and promotes interaction of pro-apoptotic proteins with BCL-XL. ONS76 (**A**) and UW228 (**B**) cells were treated with 20 µM cisplatin, collected, lysed and their expression of anti-apoptotic proteins was analysed using Western blotting. Protein levels were normalised to GAPDH and compared to that of untreated cells. Blots are representative of *N* = 3 independent experiments. Bars represent the mean ± SEM of three independent experiments. Two-way ANOVA followed by Sidak’s multiple comparisons test was used for statistical analysis, whereby ****p < 0.0001, **p < 0.01, *p < 0.05. ONS76 (**C**) and UW228 (**D**) cells were treated with PBS or 20 µM cisplatin for 18 h followed by BH3 profiling. Bars represent the mean ± SEM of N = 3 independent experiments carried out in triplicate, normalised to DMSO (0% mitochondrial depolarisation) and FCCP (100% mitochondrial depolarisation). Data was analysed using Two-Way ANOVA followed by Sidak’s test for multiple comparisons, whereby ****p < 0.0001, **p < 0.01. **E**, **F** Cells were treated with PBS, cisplatin or cisplatin and WEHI-539 for 48 h and their lysates were subjected to immunoprecipitation with an anti-BCL-XL antibody. The presence of BAX, BIM, BAK and PUMA were analysed via Western blotting, with GAPDH as a loading control. Blots are representative of *N* = 3 independent experiments. **G**, **H** Quantification of the above data. The intensity of BCL-XL-associated pro-apoptotic protein bands is represented relative to that in vehicle-treated cells, normalised to GAPDH expression. Bars represent the mean ± SEM of three independent experiments. Two-way ANOVA followed by Sidak’s multiple comparisons test was used for statistical analysis, whereby **p < 0.01, *p < 0.05.

Having identified that BCL-XL inhibition potentiates cisplatin toxicity, we wanted to understand how cisplatin affects expression of the BCL-2 protein family, and the observed BCL-XL dependency of these cells. Cisplatin treatment in ONS76 cells increased BCL-XL and MCL-1 levels, and decreased BCL-2 levels (Fig. 5A), indicating that cisplatin influences BCL-2 protein family expression in this cell line, though changes in the UW228 cell line were not significant (Fig. 5B). Cell-line dependent fluctuations were also observed in the expression levels of pro-apoptotic proteins following treatment (Supplementary Fig. 4). Such cisplatin-induced alterations in BCL-2 family protein expression may explain the lack of specificity in the DR_MOMP predictions of cell-line sensitisation mediated by BH3 mimetics.

Next, we investigated if cisplatin altered the anti-apoptotic dependencies in the medulloblastoma cell lines. Dynamic BH3 profiling showed that apoptotic priming increased following treatment with cisplatin, as indicated by the response to the BIM BH3 peptide, though not to significant extents (Fig. 5C, D). No significant cisplatin-induced changes in anti-apoptotic dependency were observed in ONS76 cells (Fig. 5C), while in UW228 cells, cisplatin significantly increased both MCL-1 and BCL-XL dependencies, as is evident by the increased response to the HRK and NOXA BH3 peptides (Fig. 5D). It is interesting to note the differential response of the cell lines to cisplatin treatment, where ONS76 cells significantly increase their protein levels of BCL-XL and MCL-1, and thus their buffering capacity against pro-apoptotic proteins, UW228 cells increase their dependence on BCL-XL and MCL-1, without significantly increasing their anti-apoptotic protein levels. Collectively, these data indicate that while BCL-XL maintains an essential basal role in protecting cells from apoptosis, both BCL-XL and MCL-1 may play a further protective role following cisplatin treatment.

To confirm the altered interaction of pro-death proteins with BCL-XL in the presence of cisplatin we performed an immunoprecipitation of BCL-XL in the two medulloblastoma cell lines following treatment with cisplatin or cisplatin and WEHI-539. Cisplatin treatment somewhat increased interactions between BAK and BCL-XL in both cell lines (Fig. 5E, F), and between BAX and BCL-XL in ONS76 cells, suggesting a potential resistance mechanism whereby BAX/BAK are sequestered by BCL-XL upon treatment with cisplatin, though these increases did not reach statistical significance (Fig. 5G, H). PUMA:BCL-XL binding also increased post-treatment in ONS76 cells, while levels of BIM:BCL-XL complexes remained unchanged. In line with its established mechanism of action, WEHI-539 released bound pro-apoptotic proteins from BCL-XL, demonstrating the mechanism of increased cisplatin sensitivity. Overall, our data show that cisplatin treatment may alter the expression of BCL-2 family proteins and increase anti-apoptotic dependencies in medulloblastoma cells. While altered protein expression may contribute to cisplatin resistance, the addition of the BCL-XL inhibitor WEHI-539 can target anti-apoptotic dependencies to unleash the death-inducing potential of cisplatin through the release of pro-apoptotic proteins from BCL-XL, enabling cell death.

### Co-inhibition of BCL-XL and MCL-1 is required to induce BH3 mimetic-mediated cell death

**Fig. 6:**
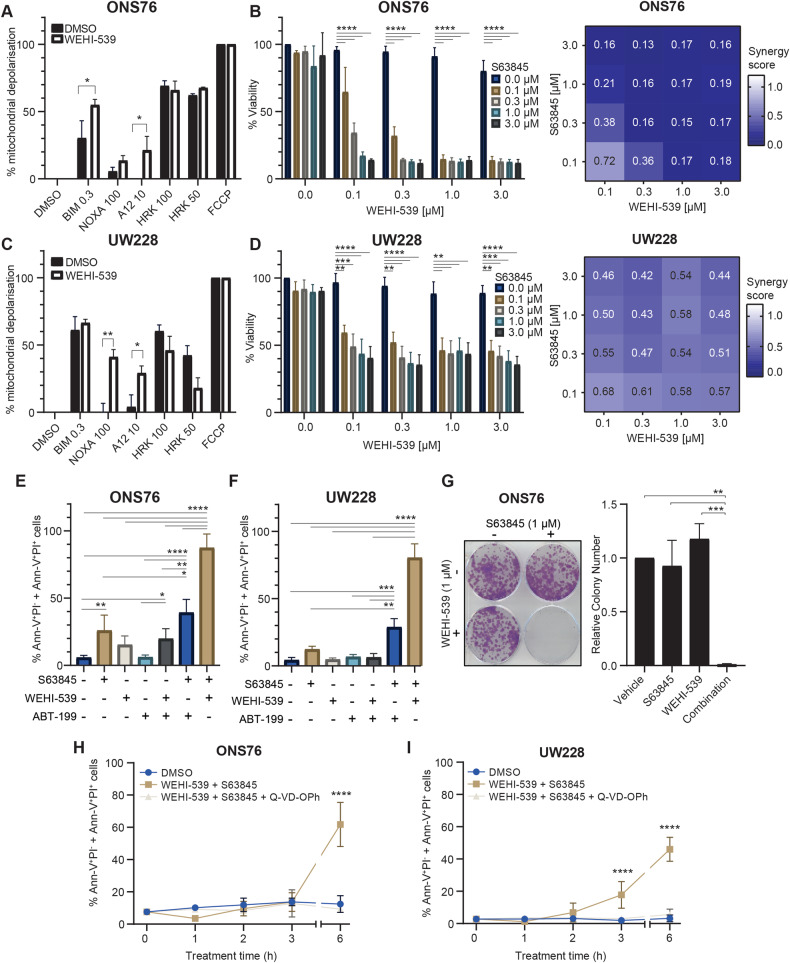
BCL-XL inhibition triggers MCL-1 dependence, and resistance to WEHI-539 is overcome by combination treatment with S63845. ONS76 (**A**) and UW228 (**C**) cells were treated with PBS or 1 µM WEHI-539 for 18 h followed by BH3 profiling. Bars represent the mean ± SEM of *N* = 3 independent experiments carried out in triplicate, normalised to DMSO (0% mitochondrial depolarisation) and FCCP (100% mitochondrial depolarisation). Data were analysed using Two-Way ANOVA followed by Sidak’s test for multiple comparisons, whereby **p < 0.01, *p < 0.05. Cell viability of ONS76 (**B**) and UW228 (**D**) was measured following 48 h treatment with increasing concentrations of WEHI-539 combined with the indicated concentrations of S63845, using the WST-1 viability assay. Bars represent the mean of *N* = 3 independent experiments carried out in duplicate ±SEM. Data were analysed using Two-Way ANOVA followed by Tukey’s test for multiple comparisons, whereby *p < 0.05, **p < 0.01, ***p < 0.001, ****p < 0.0001. Combination index values were calculated based on the above data to assess synergistic effects between WEHI-539 and S63845 where <1 indicates synergy between the two agents, while <0.8 indicates strong synergy. ONS76 (**E**) and UW228 (**F**) cells were treated for 48 h with 1 µM of the indicated BH3 mimetics either singly or in combination, and Annexin-V/PI staining followed by flow cytometry analysis was used to determine the proportion of apoptotic cells. Data are expressed as mean of *N* = 3 independent experiments ± SEM. Data were analysed using One-Way ANOVA with Tukey’s multiple comparison test, whereby *p < 0.05, **p < 0.01, ***p < 0.001, ****p < 0.0001. **G** ONS76 cells were treated as indicated for 48 h and the colony forming capacity of surviving cells was assessed by clonogenic assay. 14 days post-treatment, colonies were stained with crystal violet (left) and the number of colonies counted in ImageJ (right). Data are expressed as the mean of N = 3 independent experiments ± SEM. Data were analysed using One-Way ANOVA with Tukey’s multiple comparison test, whereby ***p < 0.001, **p < 0.01. ONS76 (**H**) and UW228 (**I**) cells were treated with either DMSO, 1 µM of the indicated BH3 mimetics, or 1 µM of the indicated BH3 mimetics following a 30 min pre-treatment with 30 µM Q-VD-OPh. Cells were treated for 0, 1, 2, 3 and 6 h, and Annexin-V/PI staining followed by flow cytometry analysis was used to determine the proportion of apoptotic cells. Data are expressed as mean of *N* = 3 independent experiments ± SEM, and were analysed Two-Way ANOVA with Tukey’s multiple comparison test, whereby ****p < 0.0001. The break in the *x* axis indicates a shortened axis.

Having shown that BCL-XL is a critical anti-apoptotic protein in protecting medulloblastoma cells from apoptosis upon treatment, we were interested in investigating why BCL-XL targeting alone failed to elicit apoptosis. Notably, a similar lack of efficacy was noted for ABT-199 and S63845 treatment (Supplementary Fig. 5). To understand the underlying BCL-2 family dynamics, we performed dynamic BH3 profiling. In both cell lines, treatment with WEHI-539 for 18 h increased MCL-1 dependence significantly, as indicated by the response to both the NOXA and A12 BH3 peptides, both of which bind and inhibit the function of MCL-1 [43] (Fig. 6A, C). This suggested a compensatory role for MCL-1 following BCL-XL inhibition.

Therefore, we hypothesised that combining WEHI-539 with S63845 would be toxic to the cells. This combination synergistically reduced viability (Fig. 6B, D), and triggered significantly more apoptosis than either agent alone (Fig. 6E, F). Confirming BCL-XL and MCL-1, and not BCL-2, as key targets, ABT-199 alone failed to induce apoptosis, and its combination with WEHI-539 or S63845 led to significantly less apoptosis than co-treatment of WEHI-539 and S63845 (Fig. 6E, F). Similar results were observed for cisplatin-sensitive D425-Med and D283-Med cells although these cell lines demonstrated somewhat higher sensitivity to individual inhibitors (Supplementary Figs. 6, 7), highlighting the potential relevance of this combination treatment across medulloblastoma subgroups, though testing in additional cell lines would establish this in the context of different subgroups and molecular backgrounds. Furthermore, combination treatment eliminated the clonogenic potential of ONS76 cells, demonstrating the long-term effects on cell survival (Fig. 6G). Confirming the functional relevance of BCL-XL and MCL-1 in mediating survival, siRNA-mediated knockdown of both proteins was required for apoptosis induction in ONS76 and UW228 cells (Supplementary Fig. 8), while combining genetic knockdown of one target with pharmacological inhibition of the other also triggered similar levels of apoptosis (Supplementary Fig. 8).

Investigating the mechanism of combination treatment-induced death, we demonstrated that apoptosis induced by combination treatment was caspase-dependent, as pre-treatment with the caspase inhibitor Q-VD-OPh prevented co-treatment-induced caspase cleavage and apoptosis (Supplementary Fig. 9). Apoptosis also occurred rapidly following treatment, with high apoptosis levels detectible from 3 h and 6 h post-treatment in UW228 and ONS76 cells respectively (Fig. 6H, I). Levels of the activator caspase pro-caspase-9 declined sharply from 1 h post-treatment, accompanied by steadily increasing levels of the active fragments, while the pro-caspase forms of the executioner caspases −3 and −7 underwent similar changes (Supplementary Fig. 9). This is in line with the mechanism of action of BH3 mimetics, where apoptosis proceeds upon release of pro-apoptotic proteins without the requirement for upstream signalling [44]. Together, this demonstrates that the apoptosis induced by the combination of WEHI-539 and S63845 is both caspase-mediated and rapid.

## Discussion

Despite increased understanding of the molecular and genetic events underlying medulloblastoma development, a lack of effective treatment options leads to poor prognosis for some patient groups [1]. In addition, radiation therapy is limited or avoided in patients for whom the side effect burden is too high. In some cases, this is successful [7], suggesting a role for chemotherapy-only treatment for a subset of patients. There is therefore a need for strategies to identify patients likely to respond to chemotherapy-only treatment, and for novel treatment combinations that do not rely on radiation therapy. To this end, previous reports demonstrated that desmoplastic/nodular histology in young patients predicted good chemotherapy response [7, 45, 46], and the Wnt molecular subtype is also associated with a good prognosis [1].

Apoptosis induction is the principal means by which chemotherapy mediates its effects, and here we aimed to identify patterns of apoptosis protein expression associated with cisplatin sensitivity, and targetable therapeutic vulnerabilities within the apoptosis pathway. We began by characterising a panel of medulloblastoma cell lines with respect to their cisplatin sensitivity and expression of apoptosis signalling proteins, and identified heterogeneous protein expression and cisplatin response across all cell lines. The prevalence of chemosensitive cell lines in our study supports the idea that many medulloblastoma tumours are intrinsically chemosensitive [37]. We also identified two highly resistant cell lines in our panel, the group 4 primary and recurrent pair CHLA-01-Med and CHLA-01R-Med, characterised by increased expression of the pro-apoptotic proteins BAX and SMAC, and reduced levels of the anti-apoptotic XIAP. While these expression patterns are counter-intuitive to cisplatin resistance, similar findings have recently been reported in melanoma, where reduced expression of BAK, BAX and SMAC was associated with increased progression-free survival [47]. Nevertheless, given the nature of our study, we reasoned that these expression patterns in this pair of cell lines were not representative of resistance to cisplatin-mediated apoptosis, limiting our studies in the group 4 subgroup to one cell line.

Previous research comprehensively demonstrated that, as defective apoptosis may underlie chemoresistance [18], quantitative analysis of apoptosis signalling proteins can accurately predict the treatment response of cancer cell lines [21–23]. Here, we integrated pathway knowledge with a systems-based statistical analysis and accurately predicted the chemosensitivity of medulloblastoma cell lines. The clinical implementation of such an approach could help stratify patients and identify those likely to respond to chemotherapy, reducing the unnecessary toxicity associated with radiation therapy in these patients. Our approach could be easily adapted to include other patient information such as molecular subgroup or mutational status data. Further development is required, and incorporating analysis of patient tumour biopsies to compare with treatment outcome would establish its potential in this setting.

We next showed that modelling the interactions of the BCL-2 protein family upstream of MOMP could also accurately predict sensitivity of medulloblastoma cells to cisplatin-induced apoptosis, with similar results previously observed in colorectal and breast cancer [27, 48]. Anti-apoptotic BCL-2 proteins are commonly highly expressed in cancer and are readily targetable by a suite of BH3 mimetics [49]. In this regard, a small number of studies previously suggested BCL-XL as a potential target in medulloblastoma [50–52]. Using BH3 profiling and dynamic BH3 profiling following cisplatin treatment, we identified BCL-XL as the dominant anti-apoptotic dependency, while cisplatin with WEHI-539 had a synergistic effect on cell viability. This was borne out in cell death analysis, and we showed that combining WEHI-539 with cisplatin releases of pro-apoptotic proteins, restoring apoptotic signalling. Elsewhere, BCL-XL inhibition has enhanced chemosensitivity in lung [53], colorectal [54], triple-negative breast [27] and ovarian cancer [55], as well as osteosarcoma [56], mesothelioma [57], glioblastoma [58], and solid paediatric malignancies including rhabdomyosarcoma [59]. BCL-XL is the most highly expressed anti-apoptotic protein in medulloblastoma biopsies [60], bolstering its relevance as a target, and this is unsurprising, given the prominent role BCL-XL mediates in normal brain development [61]. Further investigation is required to assess the potential therapeutic role of BCL-XL targeting in enhancing medulloblastoma chemosensitivity, particularly in an in vivo context.

Despite the observed BCL-XL dependence of medulloblastoma, we found that WEHI-539 treatment alone failed to effectively induce apoptosis, but that co-inhibition of MCL-1 successfully resulted in cell death. This finding is in alignment with increasing evidence that cancer cells derived from solid malignancies frequently depend on two BCL-2 family members [62], with BCL-XL and MCL-1 the most frequent pair [63]. Separate studies targeting both proteins have found success in solid malignancies where neither agent alone induced apoptosis [64–69], and this co-dependence is also evident in paediatric cancer cells including rhabdomyosarcoma, Ewing sarcoma, osteosarcoma and neuroblastoma [70]. Our finding concerning the co-dependence of medulloblastoma cells on BCL-XL and MCL-1 is supported by previous studies highlighting that cellular apoptotic sensitivity and anti-apoptotic dependence is linked with the tissue of origin [71], and as medulloblastoma cells resemble cerebellar precursor cells [72] which are also BCL-XL and MCL-1 co-dependent [73], this finding may be somewhat expected. Furthermore, previous data demonstrated a common BCL-XL dependence in murine cerebellar granule neuron progenitors and medulloblastoma cells [74].

Investigating the underlying mechanisms, we utilised dynamic BH3 profiling to demonstrate that WEHI-539 treatment triggers an exploitable MCL-1 dependence, explaining how WEHI-539 and S63845 induce death only when combined. That MCL-1 antagonism is required for apoptosis mediated by BCL-XL inhibition was hinted at in early papers reporting the activity of ABT-737, where ABT-737 induced apoptosis only in tumours with low MCL-1 levels, and MCL-1 downregulation sensitised resistant cells [75].

However, ubiquitous expression of BCL-2 family proteins limits the clinical application of certain BH3 mimetics, as some non-cancer cells are intrinsically dependent on BCL-XL or MCL-1 [62]. For example, of particular concern is the reliance of cardiomyocytes on MCL-1 for its pro-survival function [76]. Furthermore, paediatric tissues are more primed to apoptosis-inducing stimuli than corresponding adults [71] and more susceptible to damage. A potential solution is to indirectly target BCL-2 family proteins, for example with the CDK inhibitors Seliciclib [77], Dinaciclib [78], or THZ1 [67], which suppress MCL-1 levels. Secondly, ongoing development of more targeted inhibitors is addressing the lack of cancer-cell specificity of current BH3 mimetics. DT2216, for instance, is a PROTAC degrader that targets BCL-XL for degradation via Von Hippel-Lindau (VHL) E3 ligase [79], and spares platelets due to their low VHL expression, thereby circumventing the dose-limiting platelet toxicity of current BCL-XL inhibitors. Another such agent is ABBV-155 [80], a BCL-XL inhibitor conjugated to a monoclonal antibody against B7H3, which is selectively expressed by cancer cells [81] and by the majority of medulloblastoma tumours [82]. A third solution is to use sequential treatments, to initially increase the cells’ anti-apoptotic dependency by administering a cytotoxic agent, and exploiting the heightened anti-apoptotic dependency with subsequent BH3 mimetic treatment [83].

**Fig. 7:**
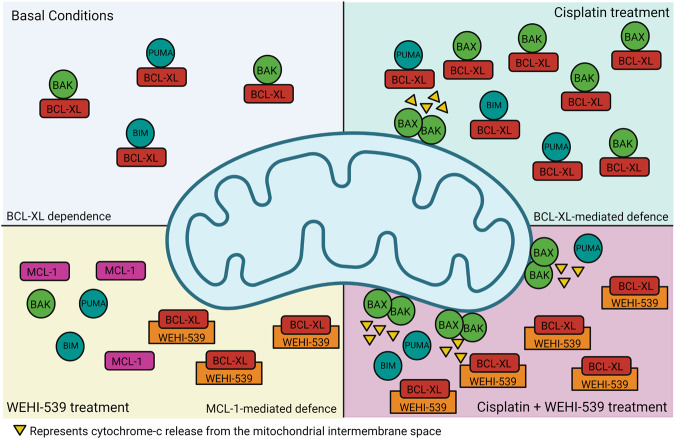
Schematic representation of BCL-XL and MCL-1-mediated protection against apoptosis in medulloblastoma cells. Under basal conditions, BCL-XL sequesters pro-apoptotic proteins BAK, PUMA and BIM. Treatment with cisplatin or WEHI-539 changes the interactions of pro-apoptotic proteins. Cisplatin treatment promotes BCL-XL mediated sequestration of these proteins, as well as BAX sequestration. The addition of WEHI-539 to cisplatin treatment results in the release of pro-apoptotic proteins and restoration of apoptotic signalling, leading to significant cell death. WEHI-539 treatment alone, on the other hand, does not result in apoptosis, and rather increases MCL-1 dependence, demonstrating that co-treatment with BCL-XL and MCL-1 inhibitors is required for BH3 mimetic mediated apoptosis in medulloblastoma cells. Created with BioRender.com.

Collectively, our data demonstrate that the cisplatin sensitivity of medulloblastoma cell lines is associated with their expression of apoptosis signalling proteins. Signalling upstream of MOMP plays a central role in mediating apoptosis susceptibility, with BCL-XL identified as a key resistance factor. Targeting BCL-XL sensitises cells to cisplatin-induced cytotoxicity, suggesting that such an approach could enhance chemotherapy response and remove the reliance of current treatment paradigms on radiation therapy (Fig. 7). MCL-1 is identified as a second line of defence that compensates for BCL-XL upon inhibition in the absence of chemotherapy, and dual targeting of BCL-XL and MCL-1 mediates highly effective cell killing (Fig. 7). Together with current efforts to improve the tolerability and cancer-specificity of BCL-2 targeting drugs, these novel treatment strategies have the potential to significantly improve the clinical management of medulloblastoma patients.

### Supplementary information

Supplementary figures and tables

Original Data File

Reproducibility checklist

## Data Availability

All datasets generated and used in this study are included in this published article and in its supplementary information files. Codes used for computational analysis can be found at https://github.com/niamhconno/Fitzgerald-et-al-2023.
