# Supplementary material for: The identification of BCL-XL and MCL-1 as key anti-apoptotic proteins in medulloblastoma that mediate distinct roles in chemotherapy resistance
Source: Cell Death Dis. 2023 Oct 28;14(10):705. doi: 10.1038/s41419-023-06231-y (PMC10613306; doi:10.1038/s41419-023-06231-y)
Supplement: Supplementary file 1 — Supplementary figures and tables [file 41419_2023_6231_MOESM1_ESM.pdf]

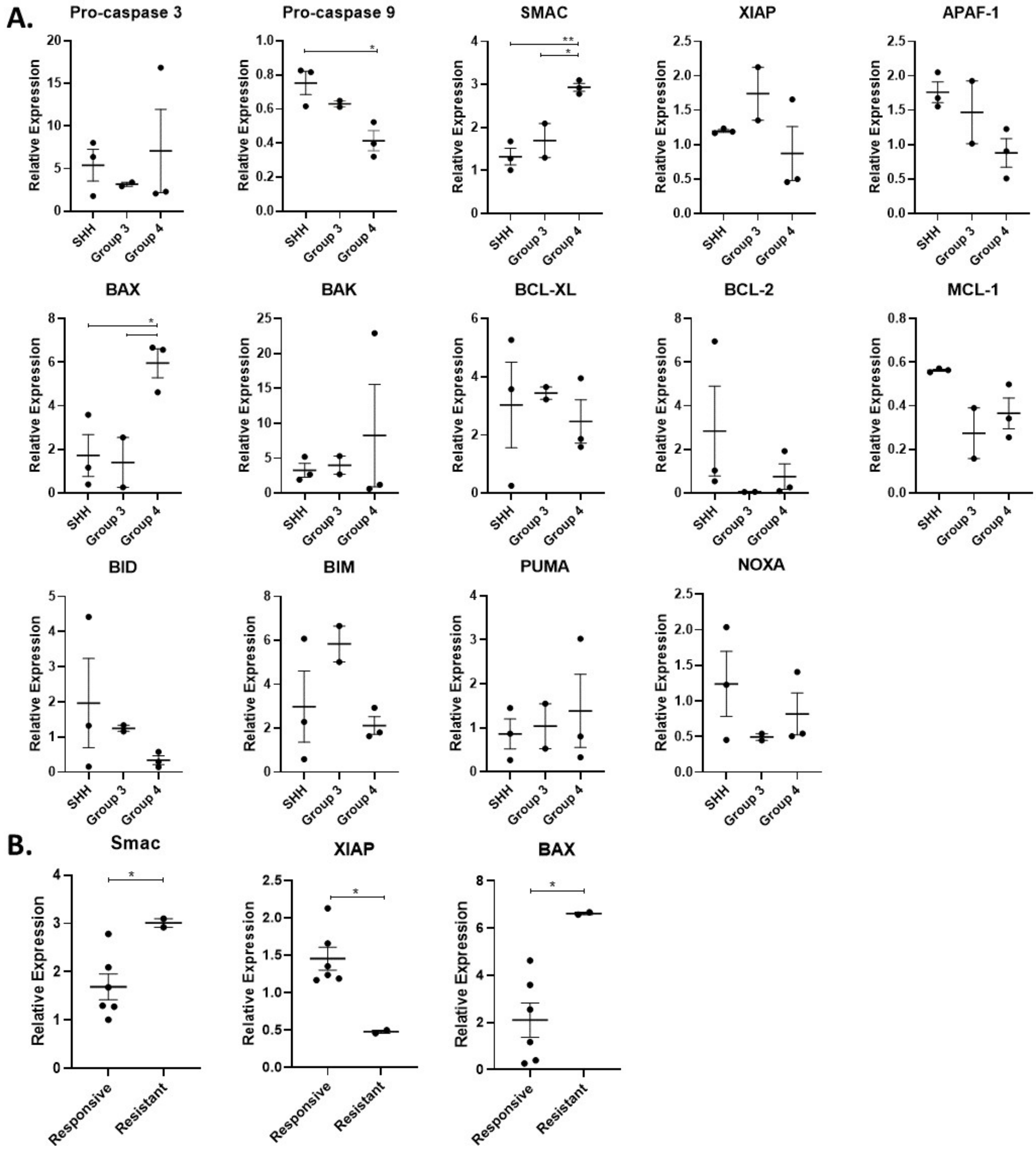

Supplementary Figure 1: Expression of apoptosis signalling proteins across molecular subgroups of medulloblastoma. (A) The mean levels of the indicated apoptosis signalling proteins were compared between the different molecular subgroups of medulloblastoma. Data points represent the mean of at least N=4 experiments. Data were compared using One-Way ANOVA followed by Tukey's multiple comparisons test. $^{**}p < 0.01$, $^{*}p < 0.05$. (**B)** The mean levels of the indicated apoptosis signalling proteins were compared between cell lines that showed a response to cisplatin treatment (Responsive) and those that did not (Resistant), based on the Wst-1 cell viability assay (Figure 1B). Data points represent the mean of at least N=4 experiments. Data were compared using the unpaired t-test. $^{*}p < 0.05$.

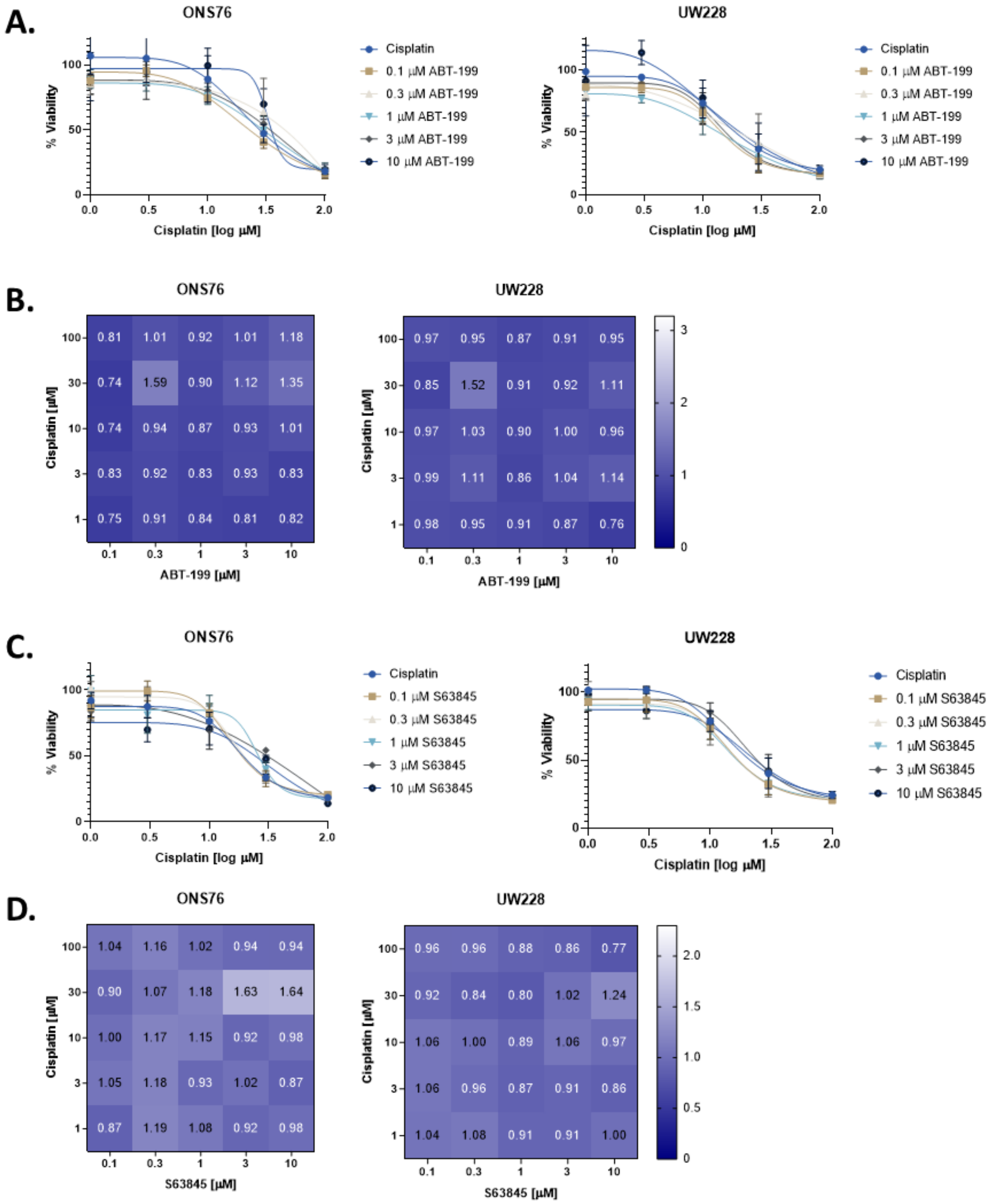

Supplementary Figure 2: Neither pharmacological inhibition of BCL-2 nor MCL-1 synergises with cisplatin to reduce cell viability in ONS76 and UW228 cell lines. (A, C) ONS76 and UW228 cells were treated with cisplatin in combination with the indicated concentrations of **(A)** ABT-199 or **(C)** S63845 for 48 h, and viability was determined using the Wst-1 viability assay. Plotted values are the mean of N=3 independent experiments carried out in duplicate, ± SEM. **(B, D)** Combination index values were calculated based on the above data to assess synergy between cisplatin and **(B)** ABT-199 and **(D)** S63845.

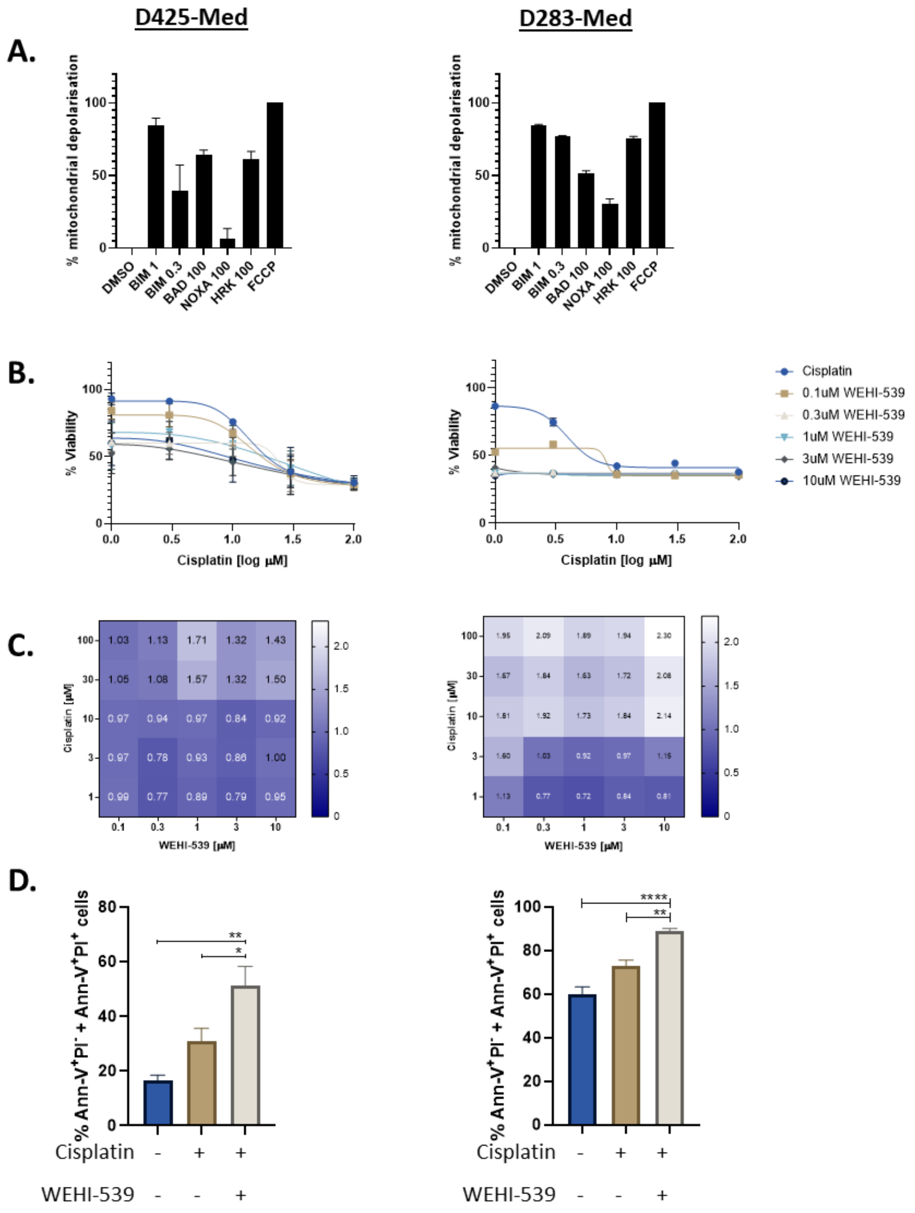

Supplementary Figure 3: Cisplatin-responsive cell lines D425-Med and D283-Med are BCL-XL dependent and WEHI-539 treatment further potentiates cisplatin cytotoxicity. (A) D425-Med and D283-Med cells were subjected to BH3 profiling and the percentage of mitochondrial depolarisation induced by incubation with the indicated peptides were determined. Bars represent the mean of N=3 independent experiments carried out in triplicate, ± SEM. **(B)** D425-Med and D283-Med cells were treated with cisplatin in combination with the indicated concentrations of WEHI-539 for 48 h, and viability was determined using the Wst-1 viability assay. Plotted values are the mean of N=3 independent experiments carried out in duplicate, ± SEM. **(C)** Combination index values were calculated based on the above viability data to assess synergy between cisplatin and WEHI-539. **(D)** D425-Med and D283-Med cells were treated as indicated with DMSO, cisplatin (1 µM) or cisplatin (1 µM) plus WEHI-539 (1 µM). Annexin-V/PI staining was carried out 48 h post-treatment and flow cytometry was used to determine the proportion of apoptotic cells. Bars represent the mean of N=3 independent experiments ± SEM. Data were analysed with One-way ANOVA followed by Tukey's multiple comparisons test, whereby ****p < 0.0001, **p < 0.01, *p < 0.05.

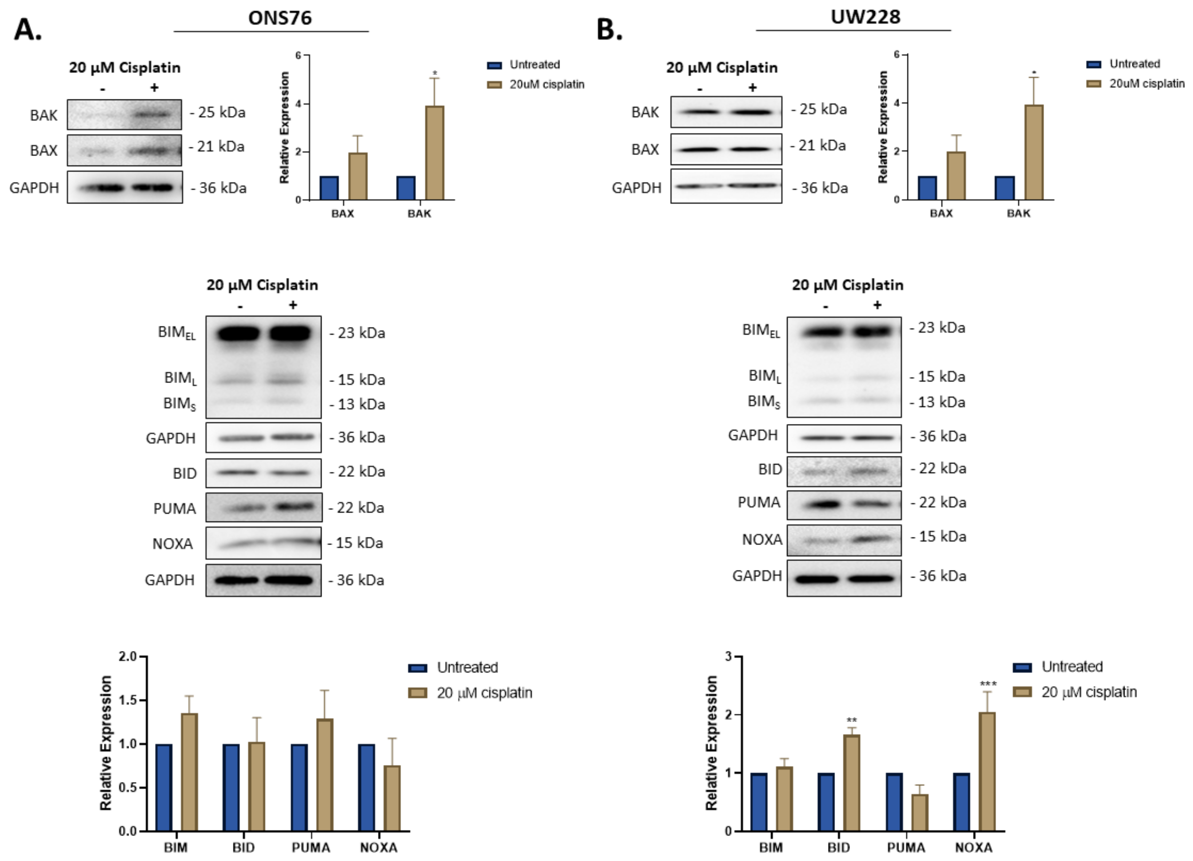

Supplementary figure 4: Cisplatin treatment results in altered expression of pro-apoptotic proteins. (A) ONS76 and **(B)** UW228 cells were treated for 48 h with 20 μM cisplatin, were collected, lysed and their expression of pro-apoptotic proteins was analysed using western blotting. Protein levels were normalized to GAPDH and compared to that of untreated cells. Blots are representative of N=3 independent experiments. Bars represent the mean ± SEM of 3 independent experiments. Two-way ANOVA followed by Sidak's multiple comparisons test was used for statistical analysis, whereby ***$p < 0.001$, **$p < 0.01$, *$p < 0.05$.

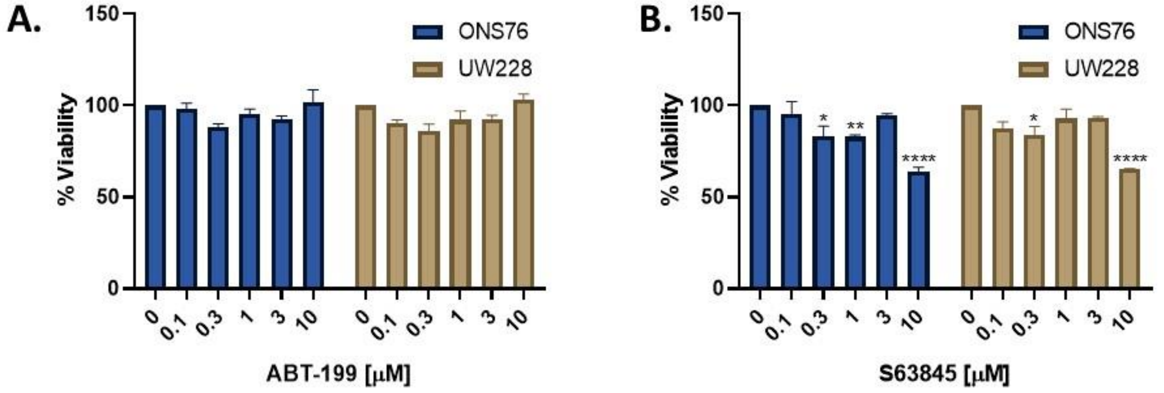

Supplementary figure 5: Sensitivity of ONS76 and UW228 cells to pharmacological inhibition of BCL-2 or MCL-1. ONS76 and UW228 cells were treated with the indicated concentrations of **(A)** ABT-199 or **(B)** S63845 for 48 h and viability was determined using the Wst-1 viability assay. Data points represent the mean ± SEM of 3 independent experiments. Data were analysed using Two-Way ANOVA followed by Dunnett's test for multiple comparisons, whereby ***$p < 0.001$, **$p < 0.01$, *$p < 0.05$. The x-axes are not continuous.

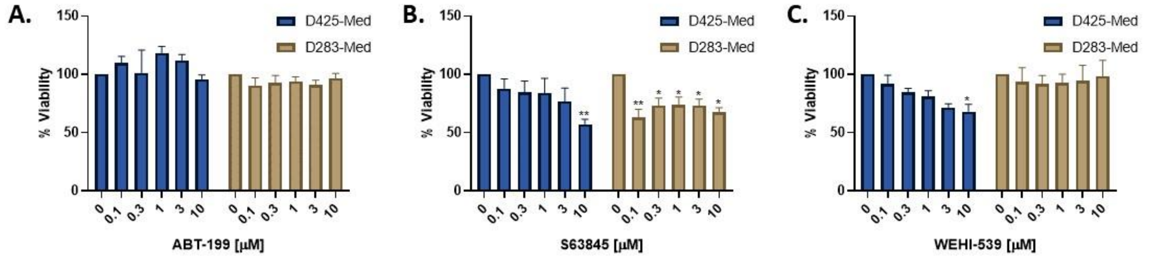

Supplementary figure 6: Sensitivity of D425-Med and D283-Med cells to BH3 mimetic treatment. D425-Med and D283-Med cells were treated with the indicated concentrations of **(A)** ABT-199, **(B)** S63845 or **(C)** WEHI-539 for 48 h and viability was determined using the Wst-1 viability assay. Data points represent the mean ± SEM of 3 independent experiments. Data were analysed using Two-Way ANOVA followed by Dunnett's test for multiple comparisons, whereby **$p < 0.01$, *$p < 0.05$. The x-axes are not continuous.

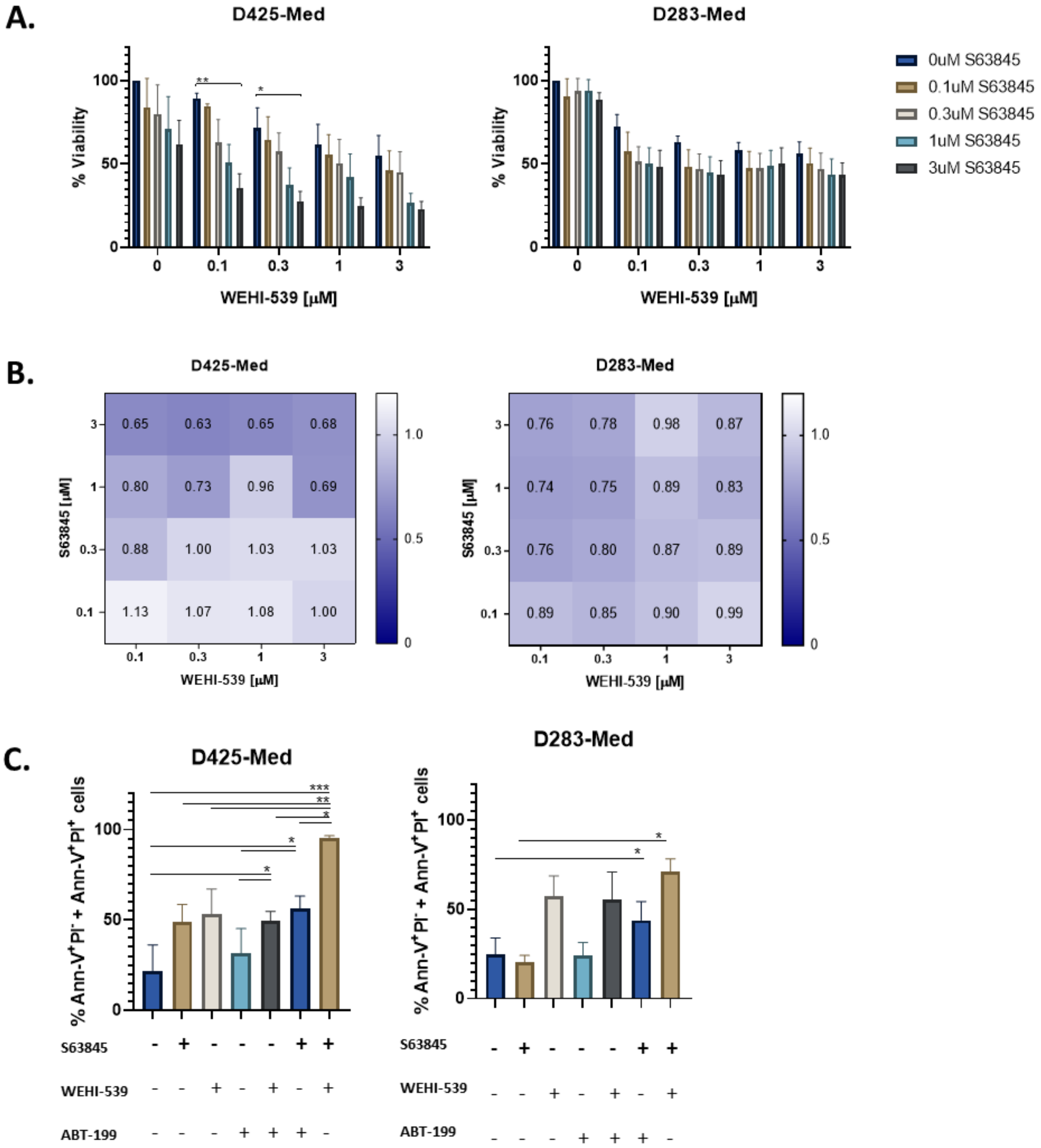

Supplementary Figure 7: Co-treatment of WEHI-539 and S63845 mediates synergistic effects on cell viability and increases cell death in D425-Med and D283-Med cells. (A) D425-Med and D283-Med cells were treated with the indicated concentrations of WEHI-539 and S63845 for 48 h, and viability was determined using the Wst-1 viability assay. Bars represent the mean of N=3 independent experiments carried out in duplicate, ± SEM. Data were analysed using Two-Way ANOVA followed by Tukey's test for multiple comparisons, whereby $^{**}p < 0.01$, $^{*}p < 0.05$. **(B)** Combination index values were calculated based on the above data to assess synergy between WEHI-539 and S63845. **(C)** Cells were treated with 1 μM of the indicated BH3 mimetics either singly or in combination, and Annexin-V/PI staining followed by flow cytometry analysis was used to determine the proportion of apoptotic cells. Data are expressed as mean of N=3 independent experiments ± SEM. Data were analyzed using One-Way ANOVA with Tukey's multiple comparison test, whereby $^{***}p < 0.001$, $^{**}p < 0.01$, $^{*}p < 0.05$.

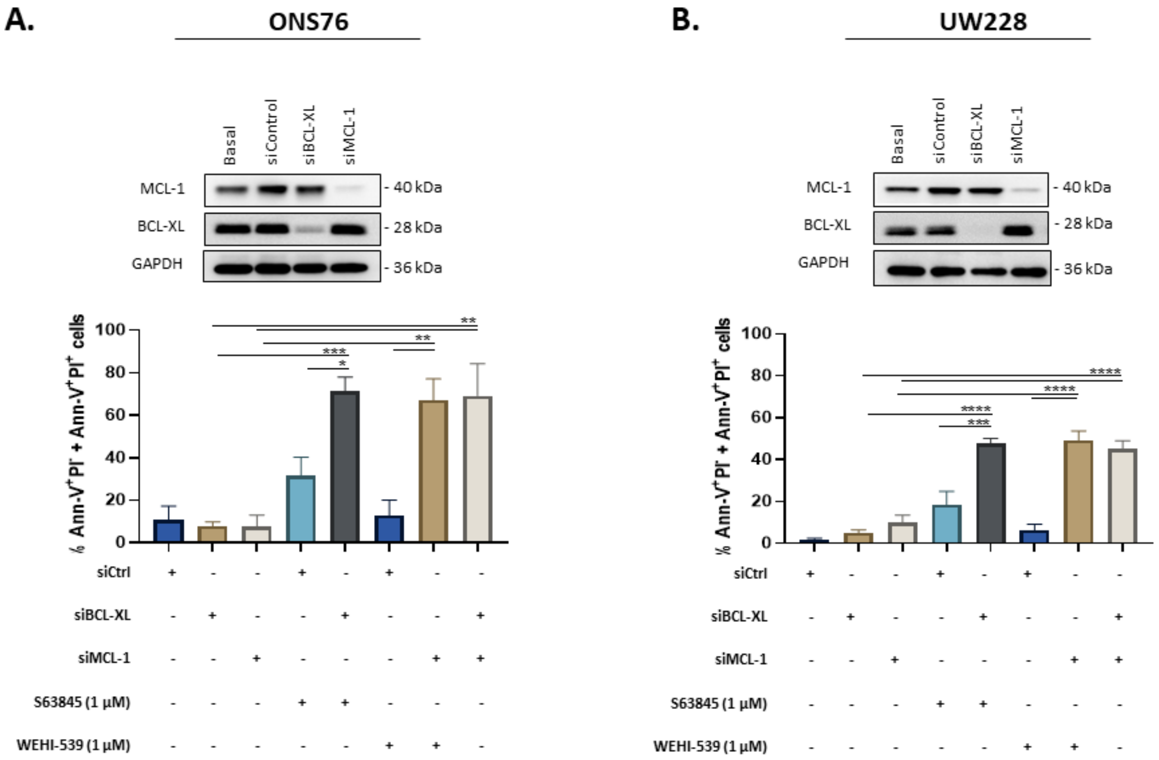

Supplementary figure 8: Specific inhibition of BCL-XL and MCL-1 induces apoptosis in ONS76 and UW228 cells. (A) ONS76 and **(B)** UW228 cells were transfected with siRNA against BCL-XL and/or MCL-1, with non-targeting siRNA used as a control. Protein expression of BCL-XL and MCL-1 was examined by western blotting to verify knockdown 48 h post-transfection, and GAPDH was used as a loading control. Blots shown are representative of N=3 independent experiments. Cells were treated as indicated with 1 µM WEHI-539 or S63845, and cell death was determined using Annexin-V/PI staining and flow cytometry. Bars represent the mean of N=3 independent experiments, and data was analyzed using One-Way ANOVA followed by Tukey's test for multiple comparisons, whereby ****$p < 0.0001$, ***$p < 0.001$, **$p < 0.01$, *$p < 0.05$.

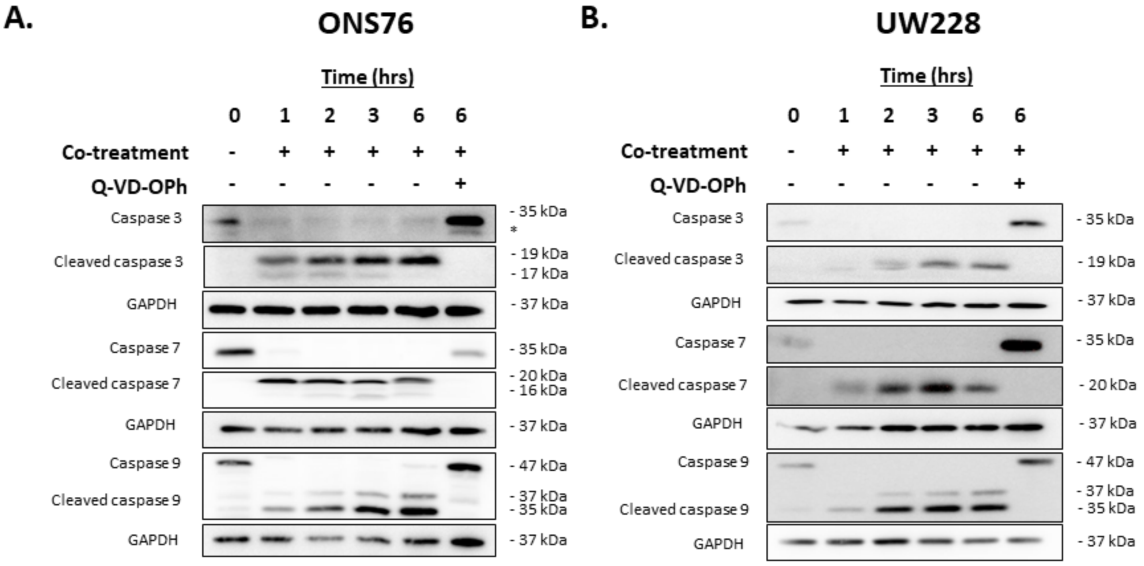

Supplementary figure 9: Apoptosis triggered by co-treatment of WEHI-539+S63845 is rapid and caspase-dependent. (A) ONS76 and **(B)** UW228 cells were treated as indicated and caspase activation following combination treatment of WEHI-539 and S63845 with/without the addition of Q-VD-OPh was followed over 6 h. Changing levels of caspase 3, -7 and -9, and cleaved caspases 3, -7 and -9 were followed by western blot analysis. GAPDH was used as a loading control. Representative blots of N=3 independent experiments are shown here. * indicates non-specific antibody binding.

Supplementary table 1

	APAF-1	BAK	BAX	BCL-2	BCL-XL	BID	CASPA-SE 3	CASPA-SE 9	MCL-1	SMAC	XIAP	BIM	PUMA	NOXA
**Daoy**	2.0511	1.9213	1.1715	0.5427	0.2534	4.4177	8.0245	0.8262	0.5718	1.6767	1.1885	0.5894	0.2687	1.2280
**ONS76**	1.5544	2.6527	0.3991	1.0376	5.2692	1.3237	1.7799	0.8164	0.5544	1.2779	1.2357	2.2918	0.8681	2.0364
**UW228**	1.6787	5.2036	3.5924	6.9570	3.5714	0.1562	6.3693	0.6160	0.5634	1.0086	1.1680	6.0740	1.4479	0.4532
**D425-Med**	1.0148	2.6955	0.2604	0.0585	3.2232	1.1585	3.3856	0.6123	0.1580	2.0915	1.3541	5.0170	0.5297	0.5413
**D458-Med**	1.9262	5.3007	2.5524	0.0492	3.6478	1.3347	2.9194	0.6483	0.3905	1.2984	2.1265	6.6571	1.5491	0.4469
**CHLA-01-Med**	1.2273	1.1979	6.5714	0.2659	1.8614	0.5759	16.8540	0.5232	0.3421	3.0972	0.4579	2.9301	0.8048	1.4067
**CHLA-01R-Med**	0.5085	0.6139	6.6669	0.0883	1.5854	0.2975	2.2853	0.3201	0.4984	2.9232	0.4994	1.8101	0.3302	0.5048
**D283-Med**	0.9057	22.9157	4.6248	1.9247	3.9474	0.1457	2.0791	0.3975	0.2559	2.7805	1.6573	1.6363	3.0222	0.5428

Supplementary table 1: Mean expression levels of the indicated proteins across the medulloblastoma cell lines. Values are the mean of at least N=4 independent experiments, and shown relative to expression in the HeLa cell line.

Supplementary table 2

Protein	R^2	P
BID	0.4901	0.1214
BIM	0.1378	0.4688
PUMA	0.02750	0.7535
NOXA	0.007536	0.8701
MCL-1	0.05937	0.6417
BCL-XL	0.4669	0.1346
BCL-2	0.5634	0.0856
BAK	0.0007	0.9602
BAX	0.02736	0.7542
APAF-1	0.04265	0.6946
Caspase 9	0.01113	0.8423
XIAP	0.1410	0.4633
Caspase 3	0.03799	0.7113
SMAC	0.1725	0.4130

Supplementary table 2: Mean relative expression levels of apoptosis signalling proteins were correlated with apoptosis induced following cisplatin treatment in Daoy, ONS76, UW228, D425-Med, D458-Med and D283-Med cells. Correlation was assessed using Pearson's correlation coefficient.
