# Supplementary material for: The identification of BCL-XL and MCL-1 as key anti-apoptotic proteins in medulloblastoma that mediate distinct roles in chemotherapy resistance
Source: Cell Death Dis. 2023 Oct 28;14(10):705. doi: 10.1038/s41419-023-06231-y (PMC10613306; doi:10.1038/s41419-023-06231-y)

Figure 1C

BID

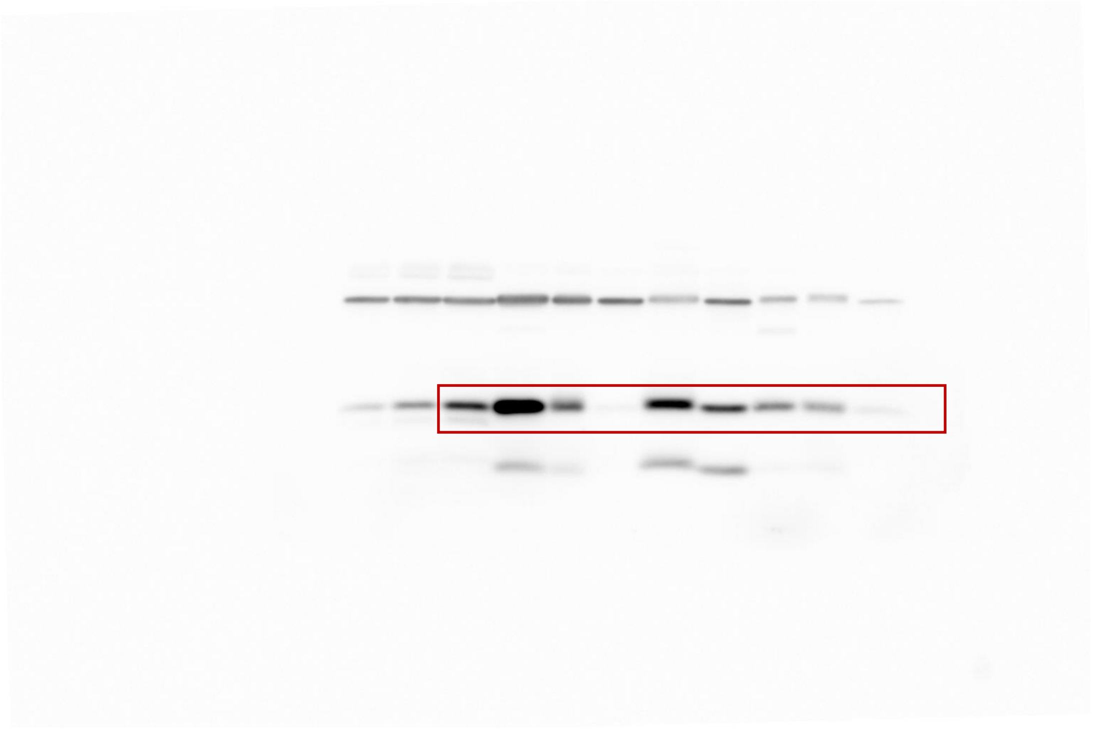

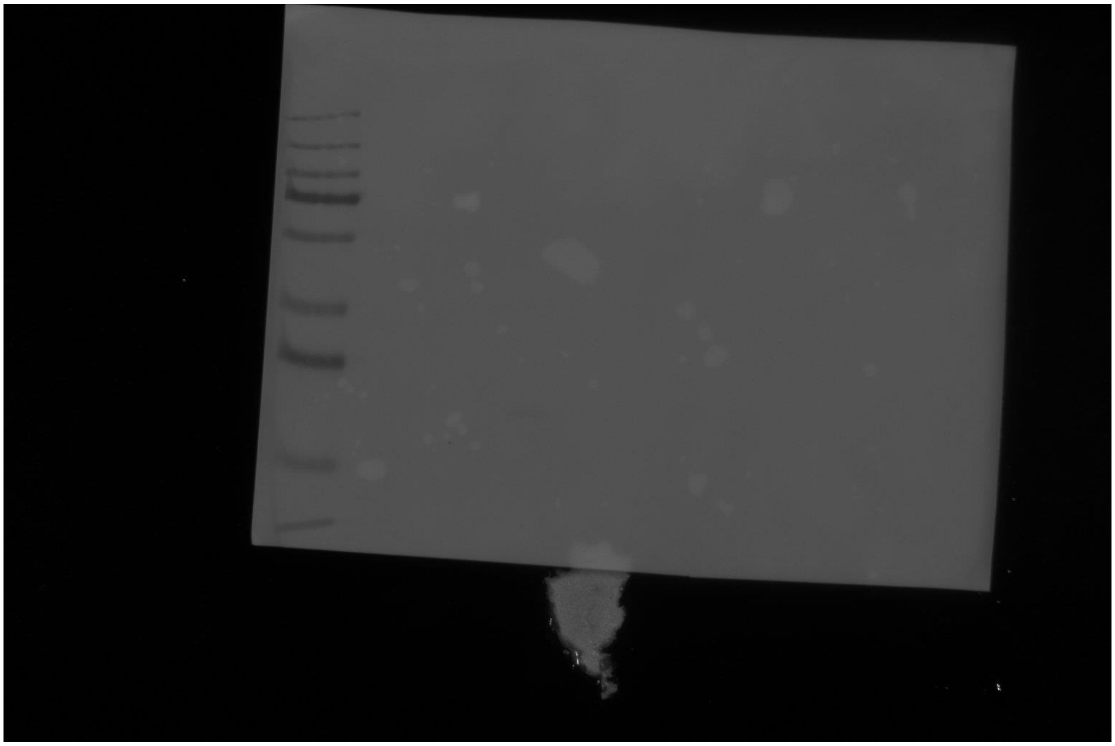

Figure 1C

BIM

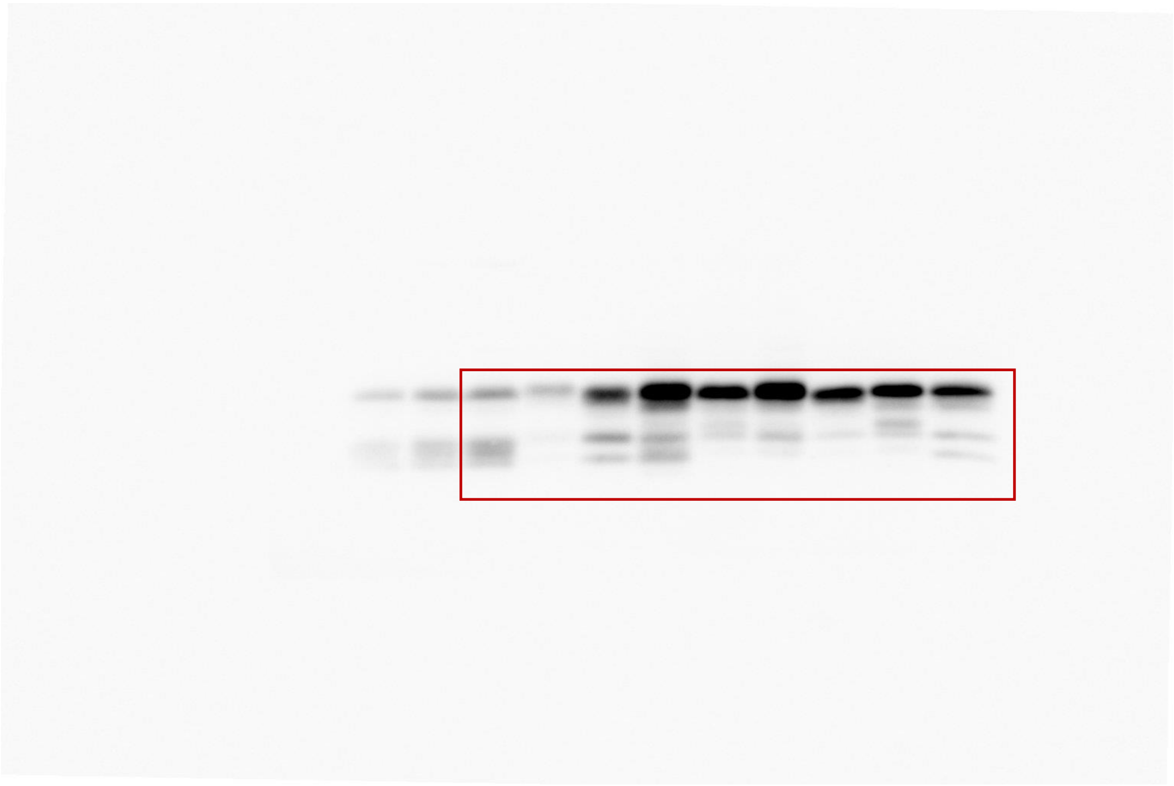

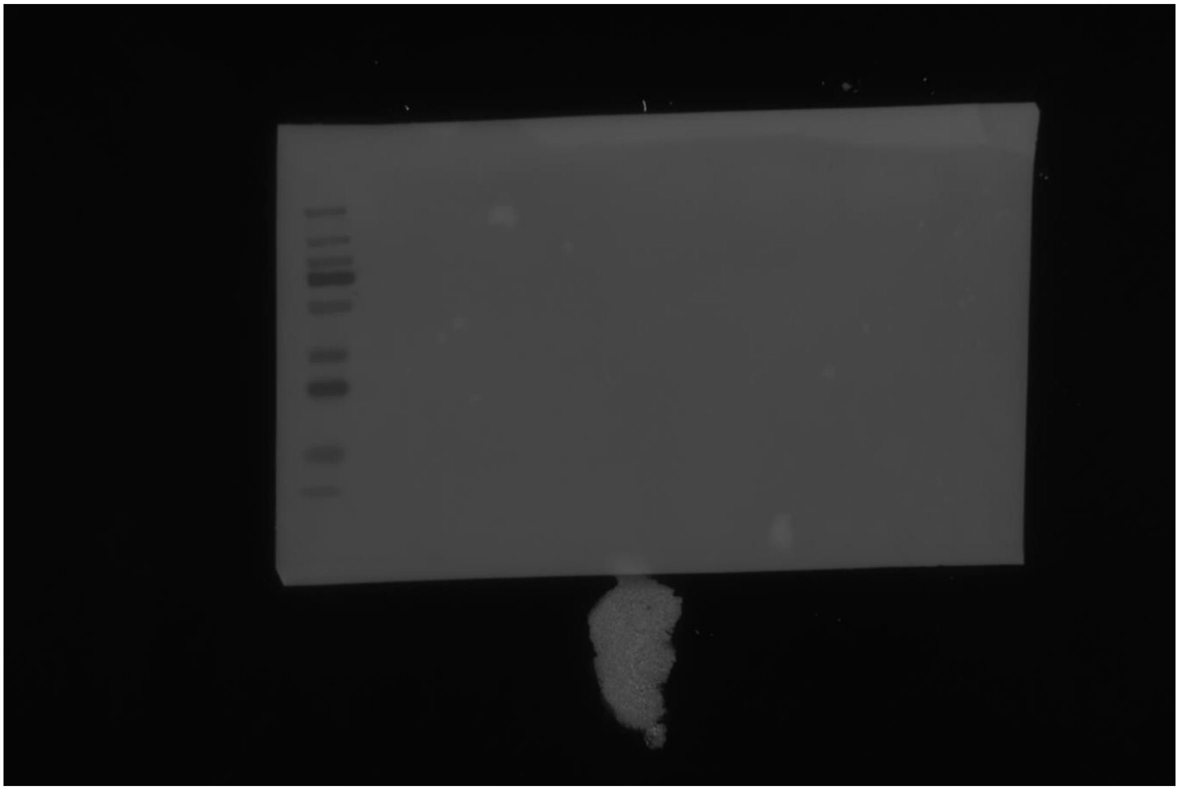

Figure 1C

PUMA

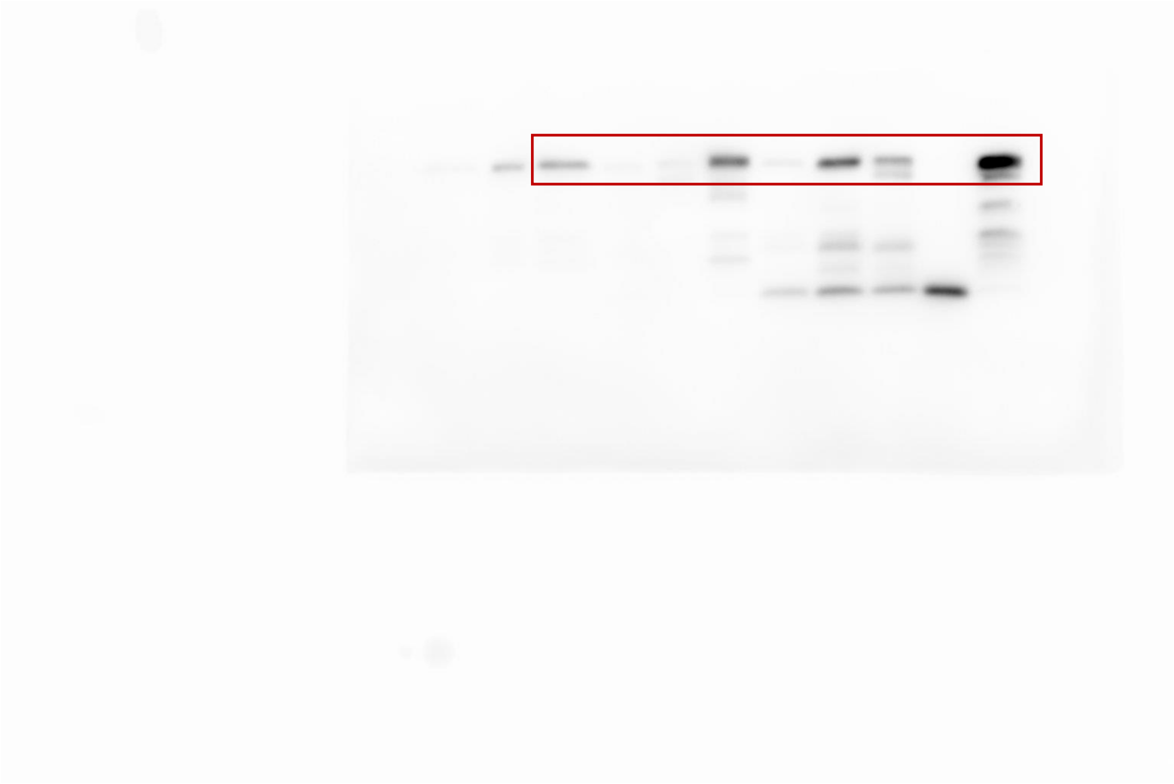

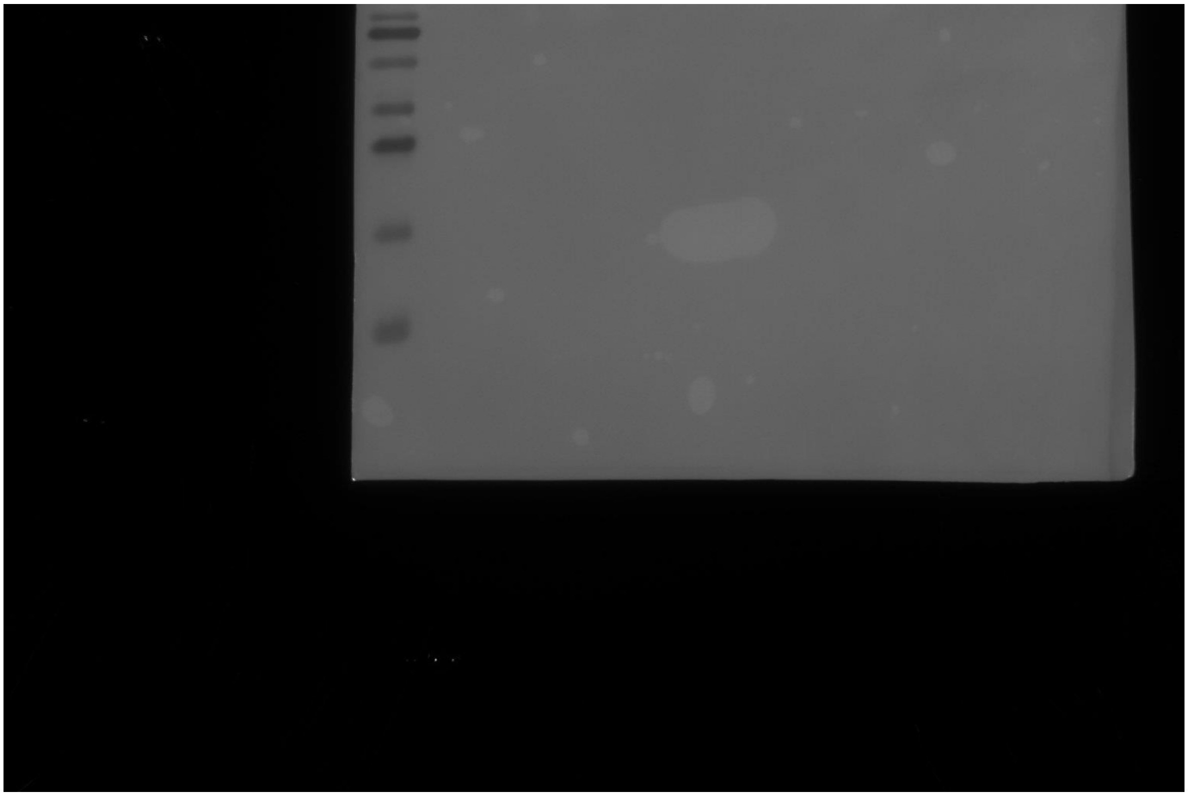

Figure 1C

NOXA

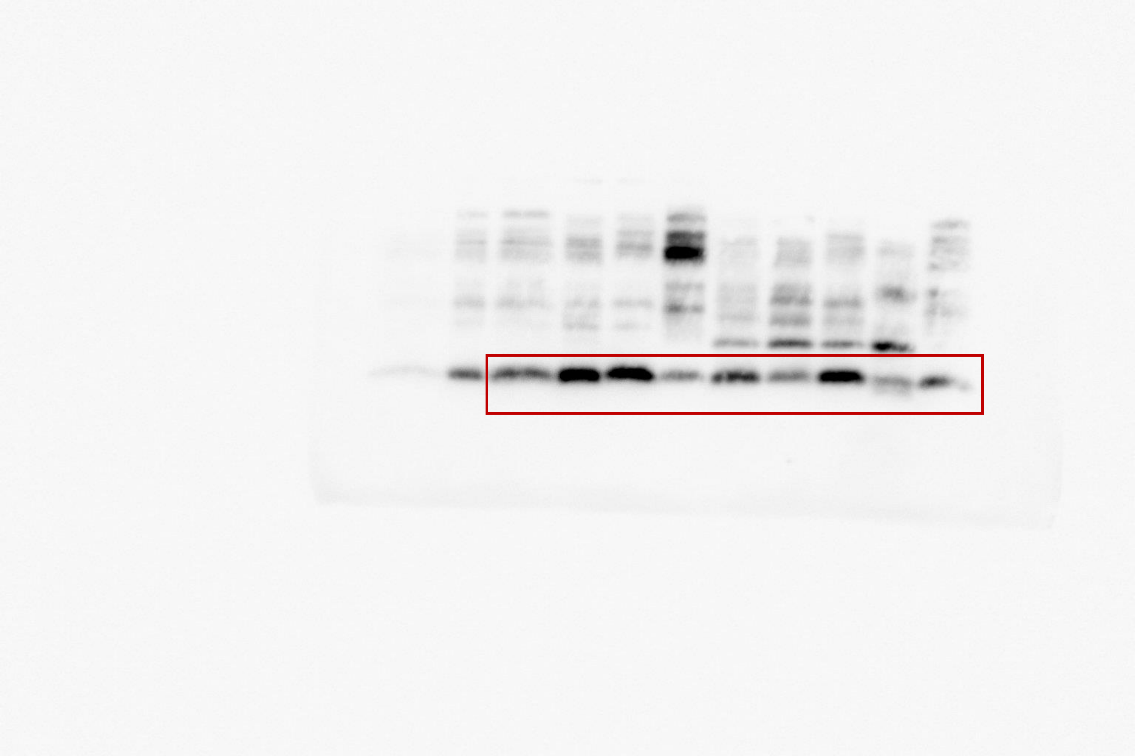

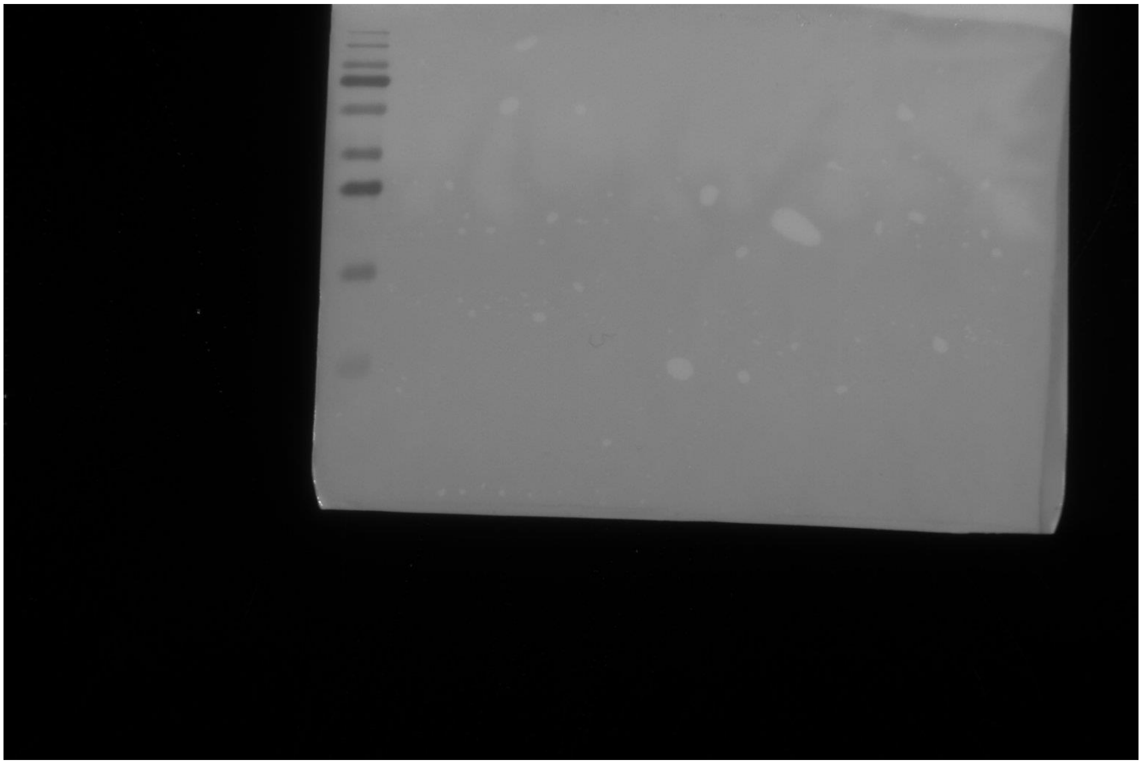

Loading control

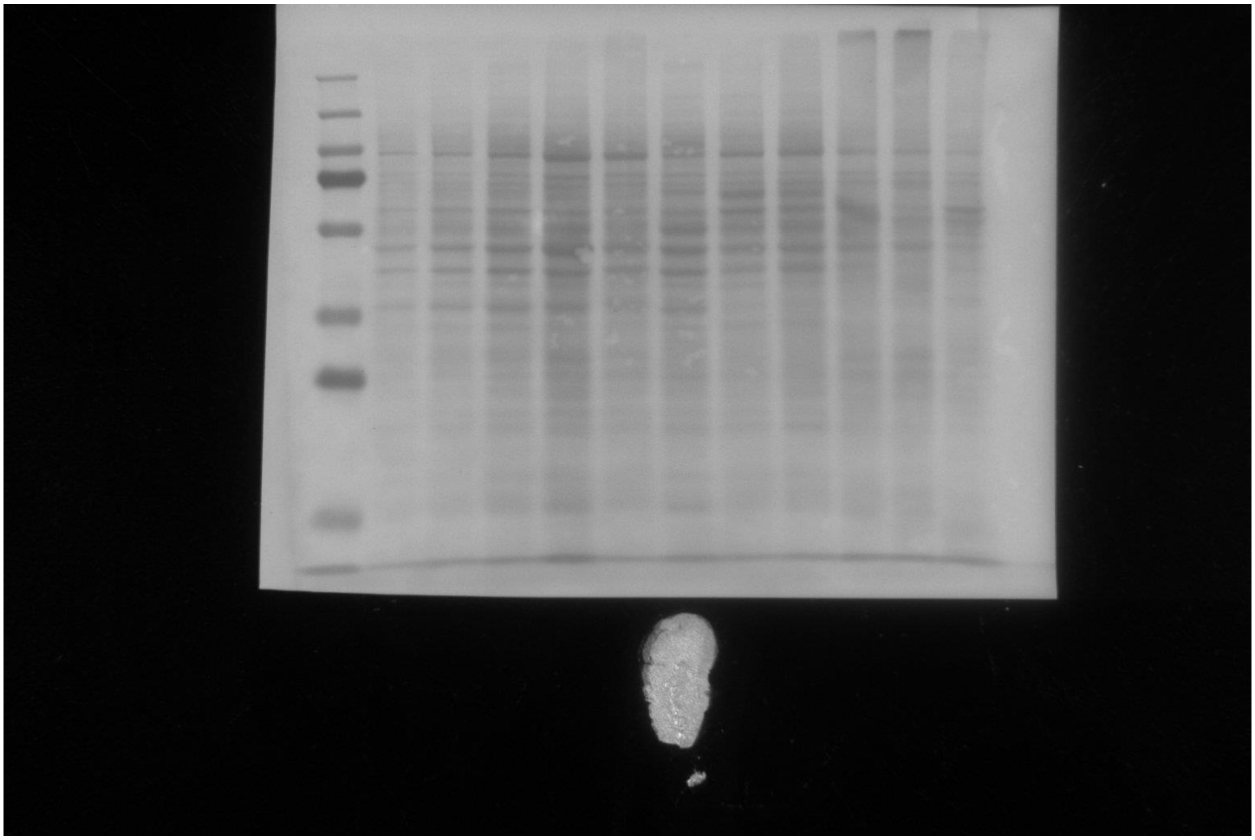

Figure 1C

MCL-1

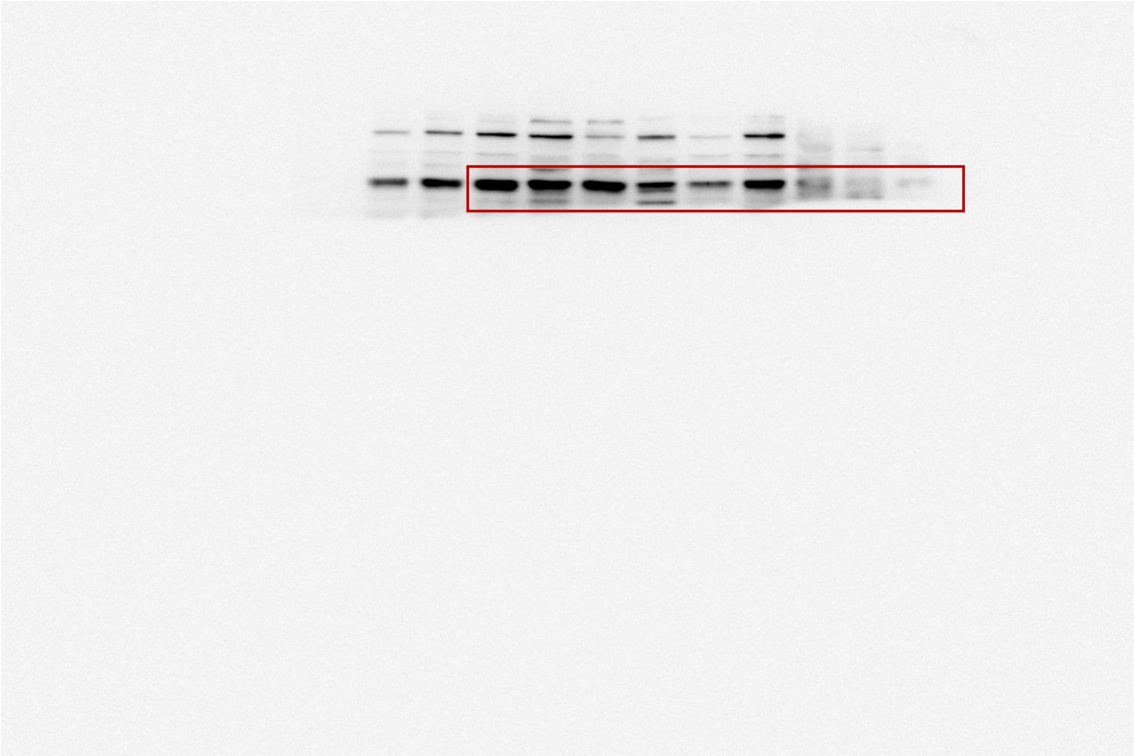

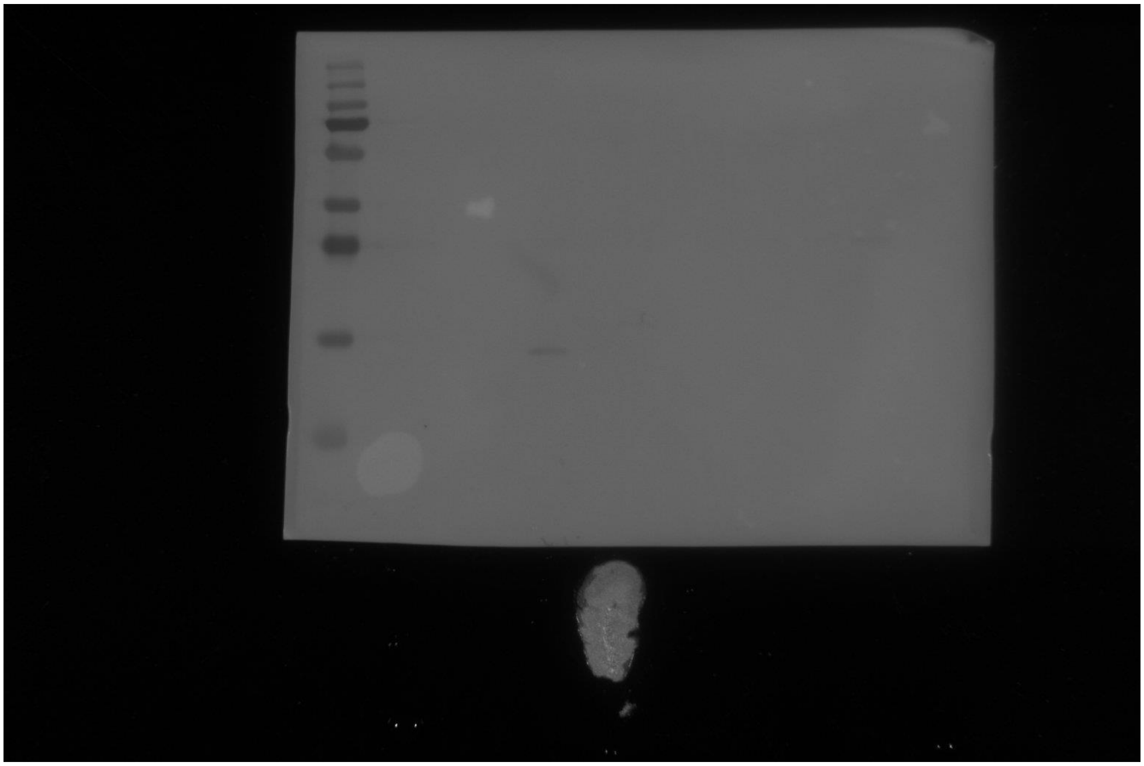

Figure 1C

BCL-XL

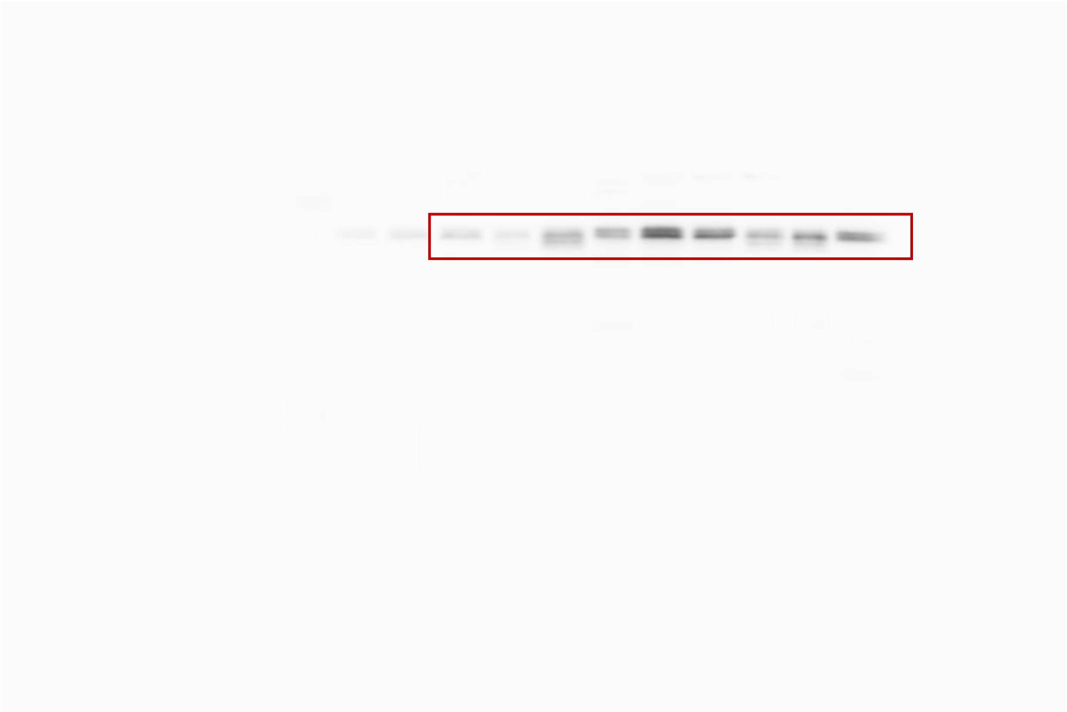

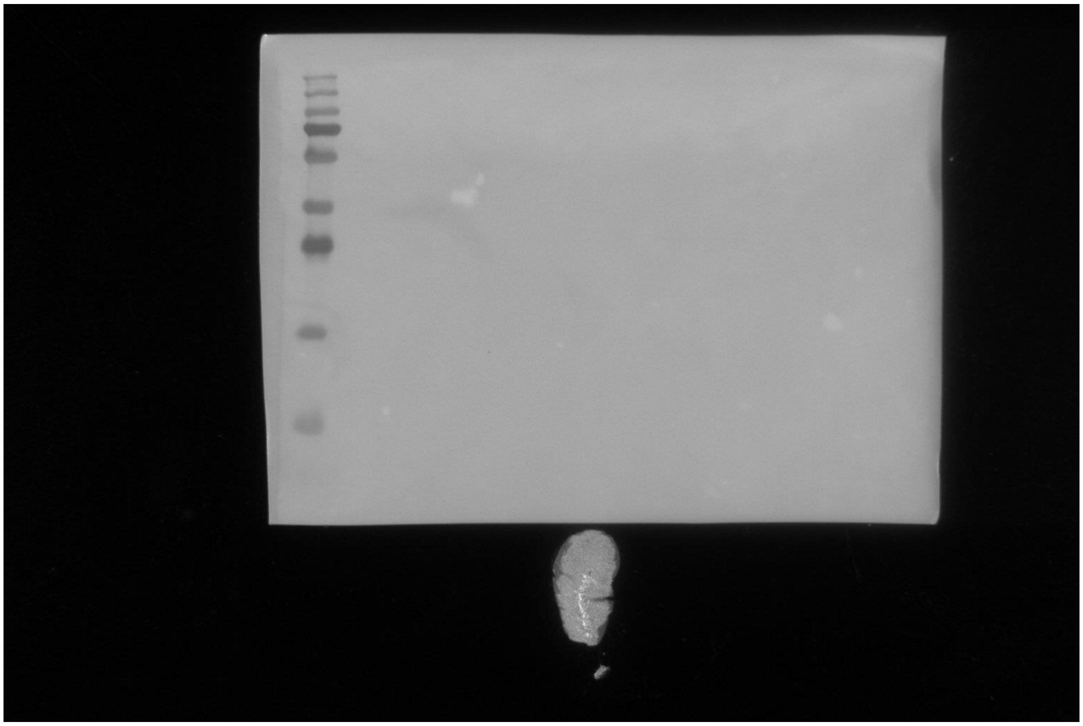

Figure 1C

BCL-2

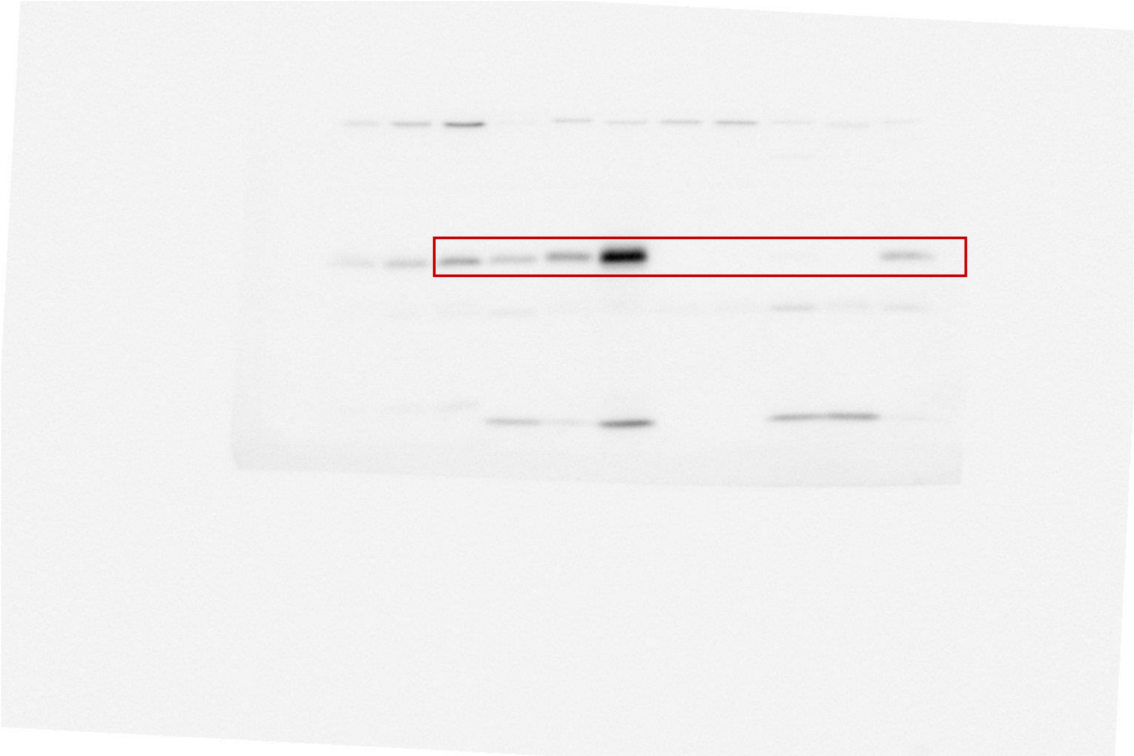

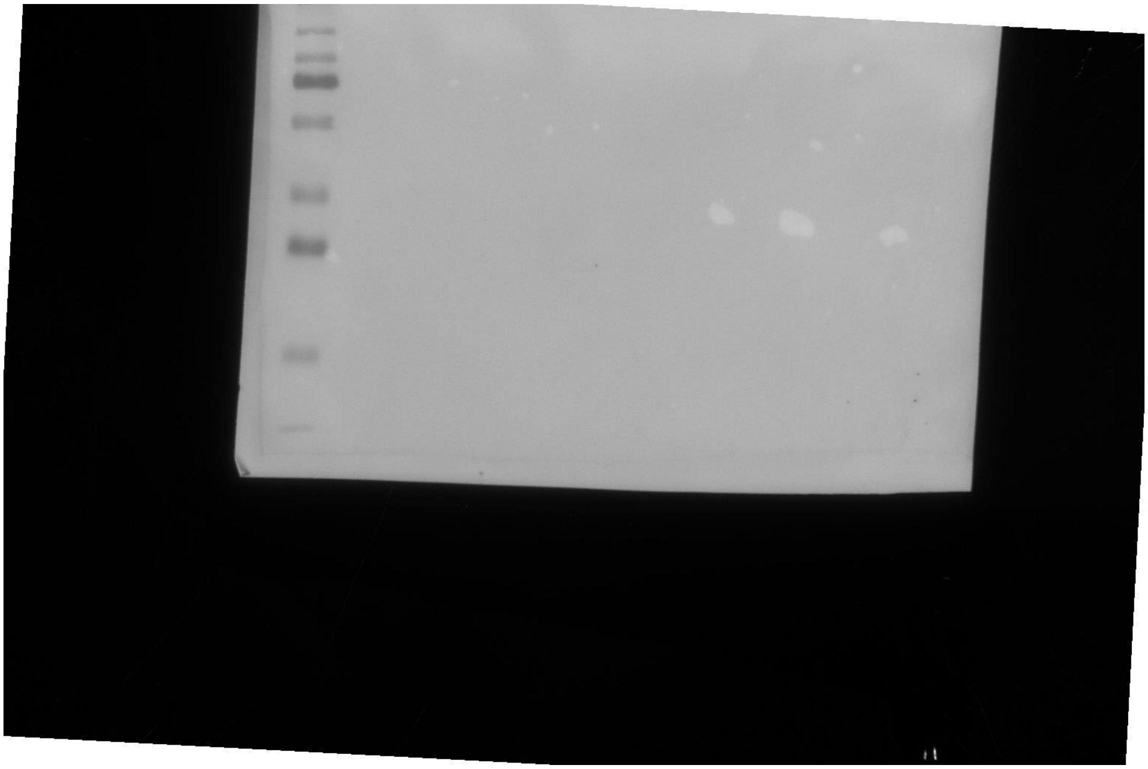

Figure 1C

BAK

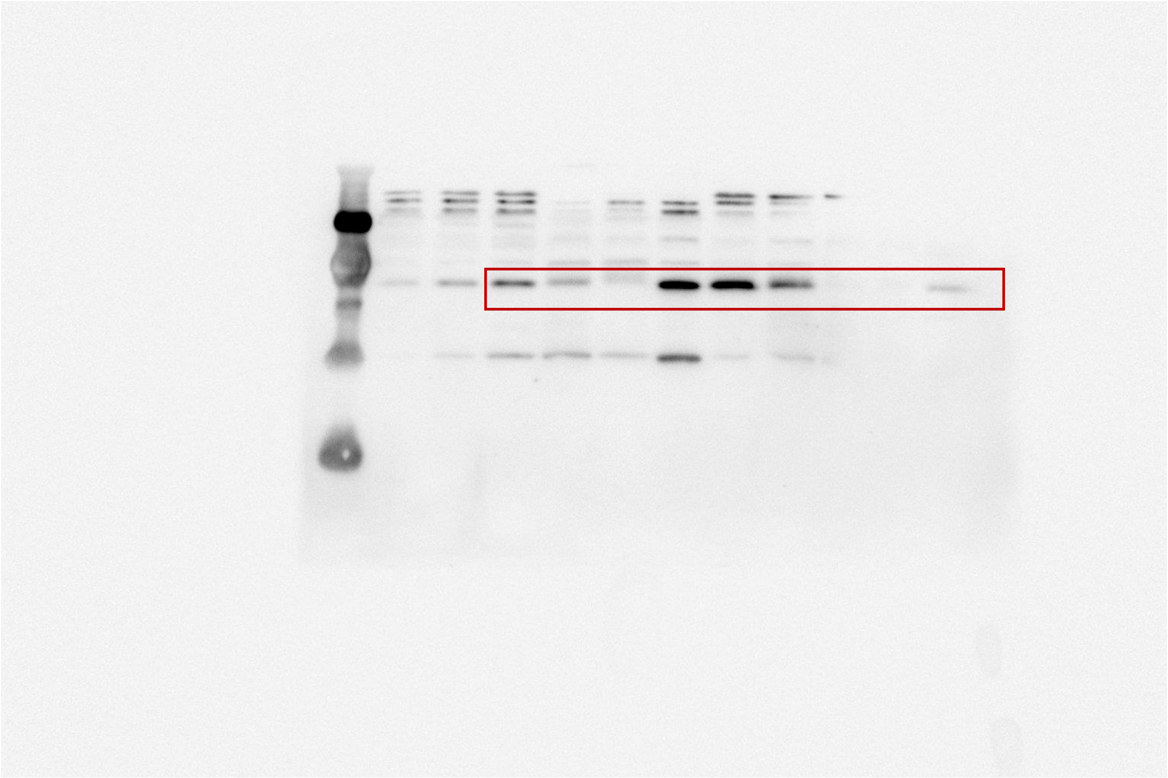

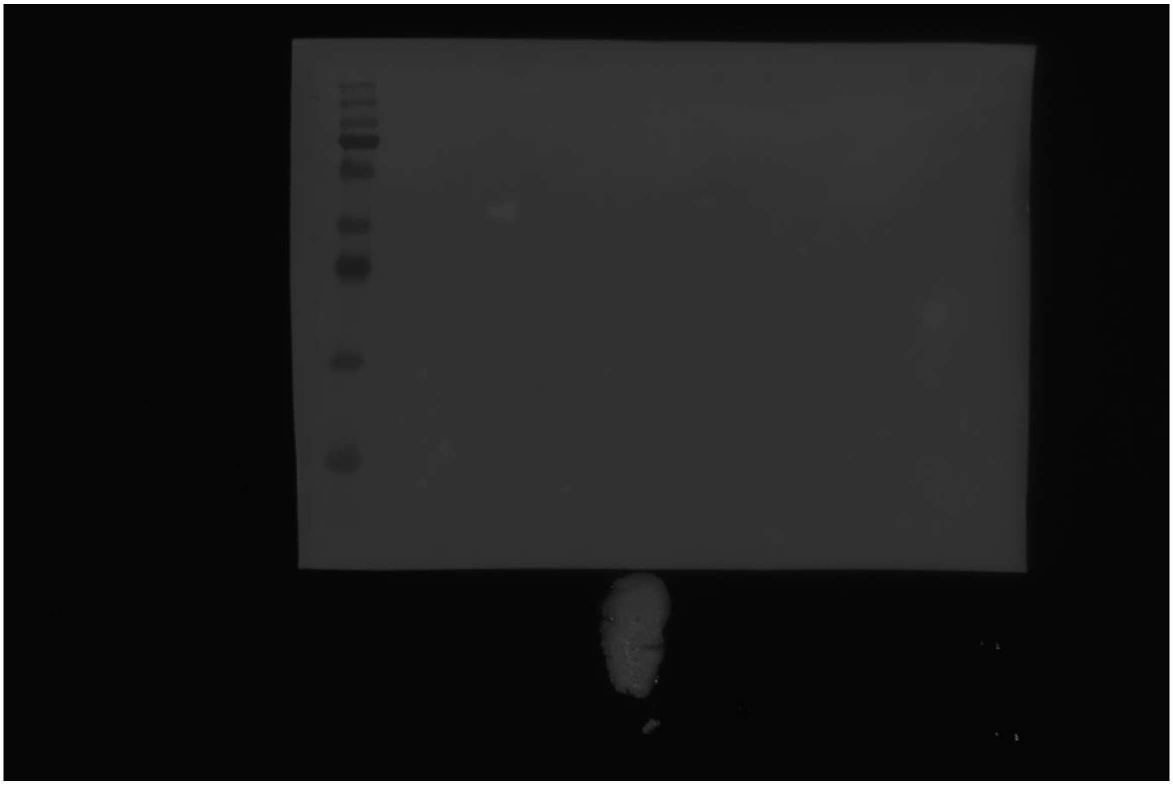

Figure 1C

BAX

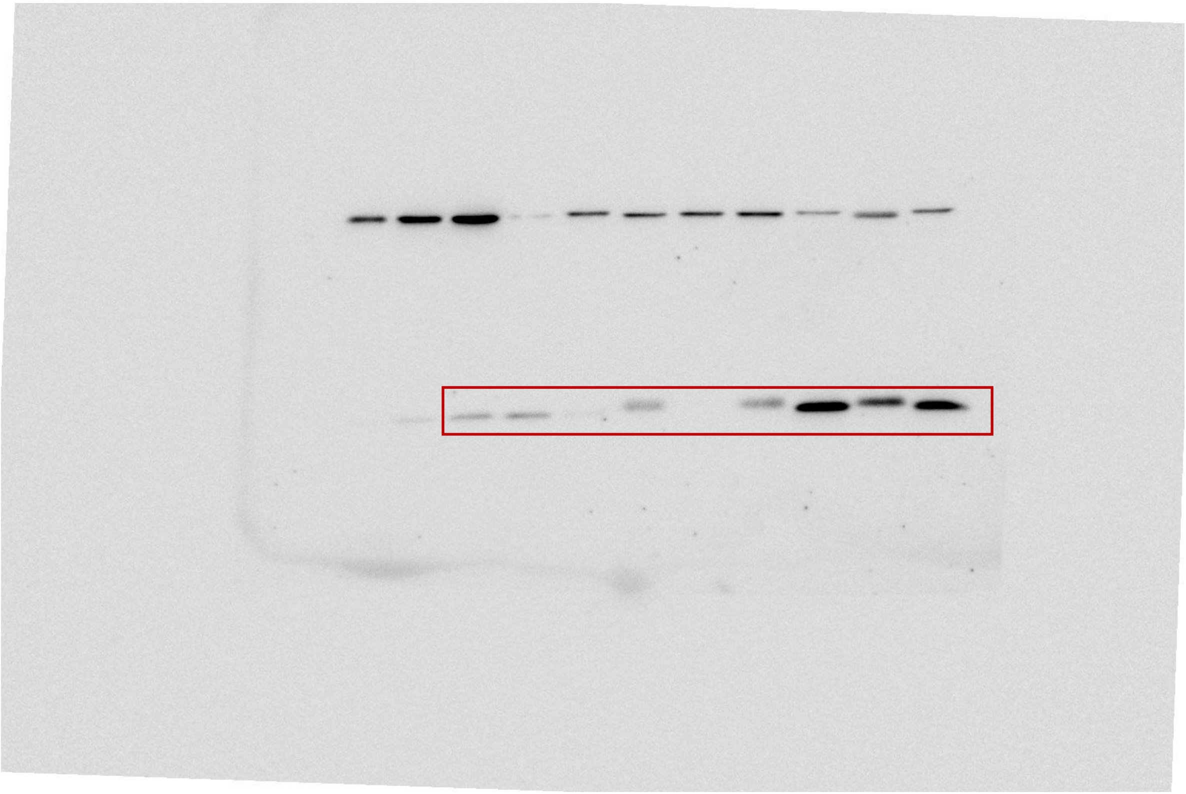

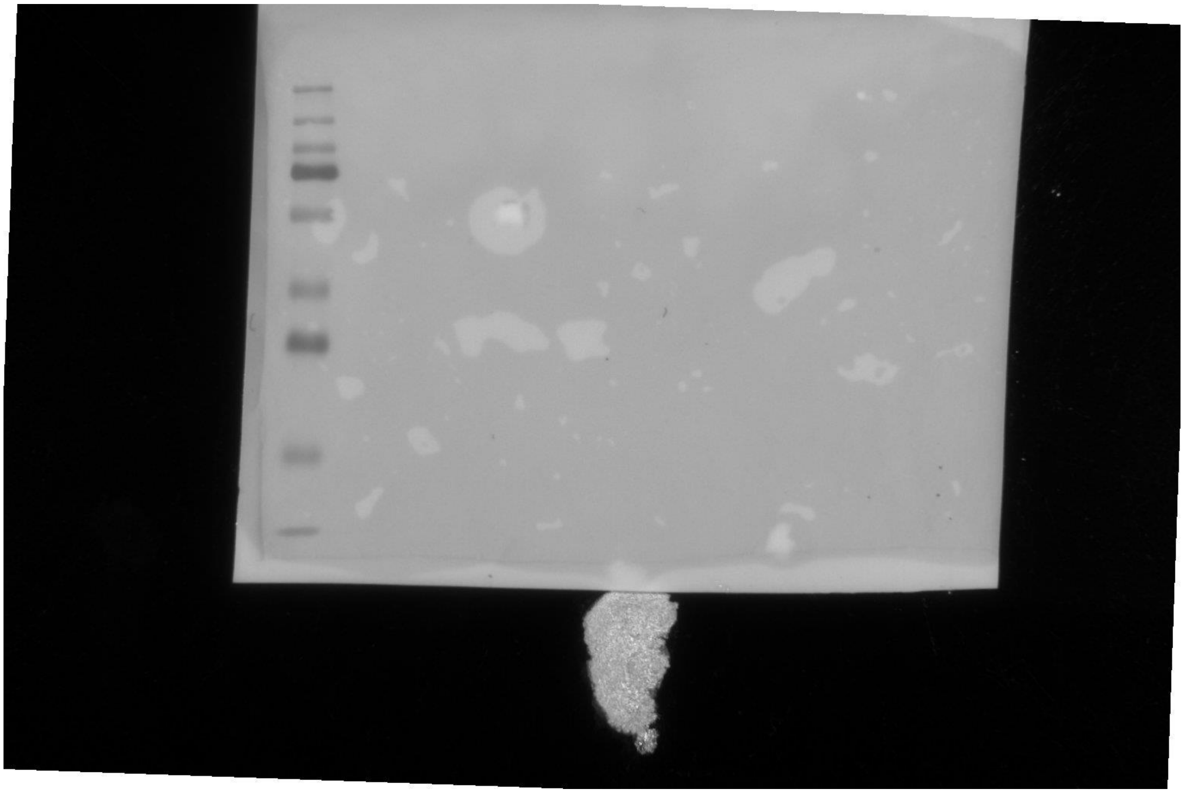

Figure 1C

Loading control

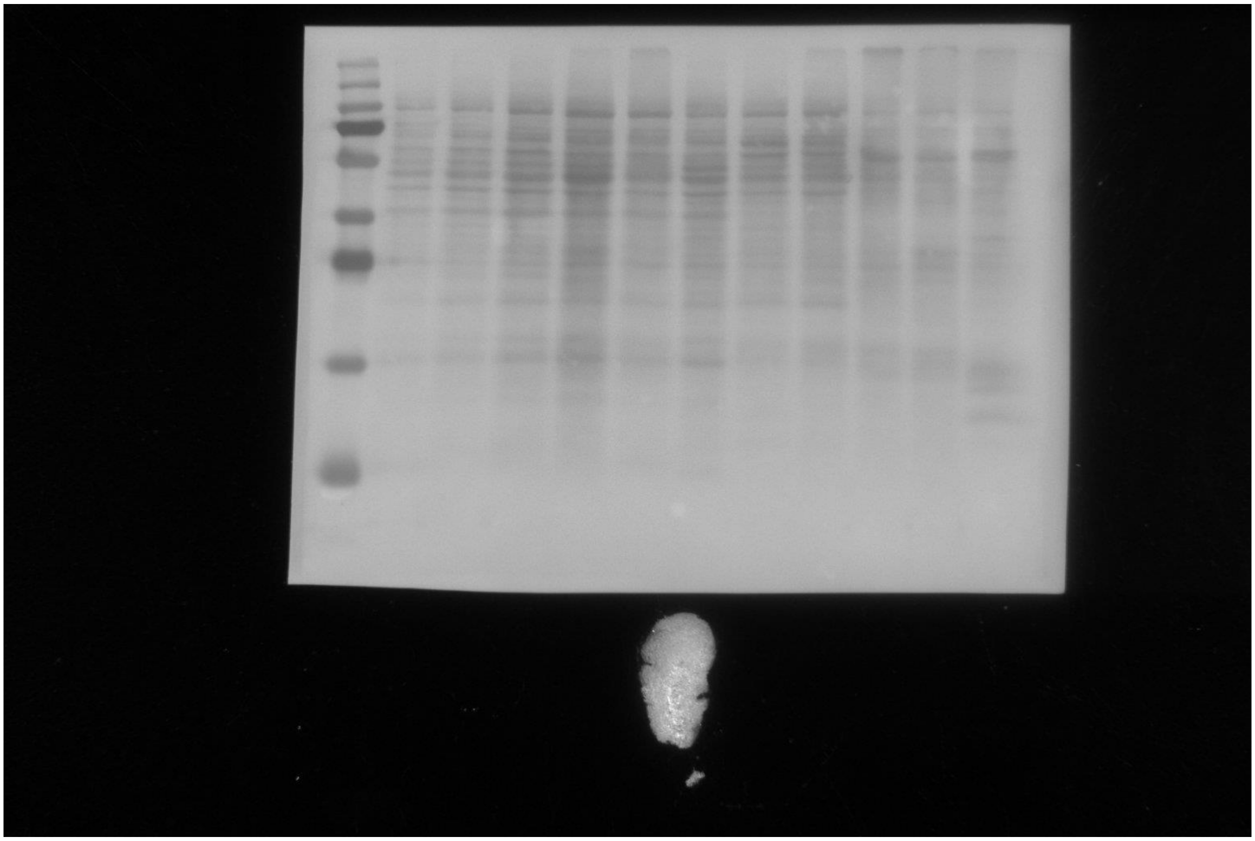

Figure 1C

APAF-1

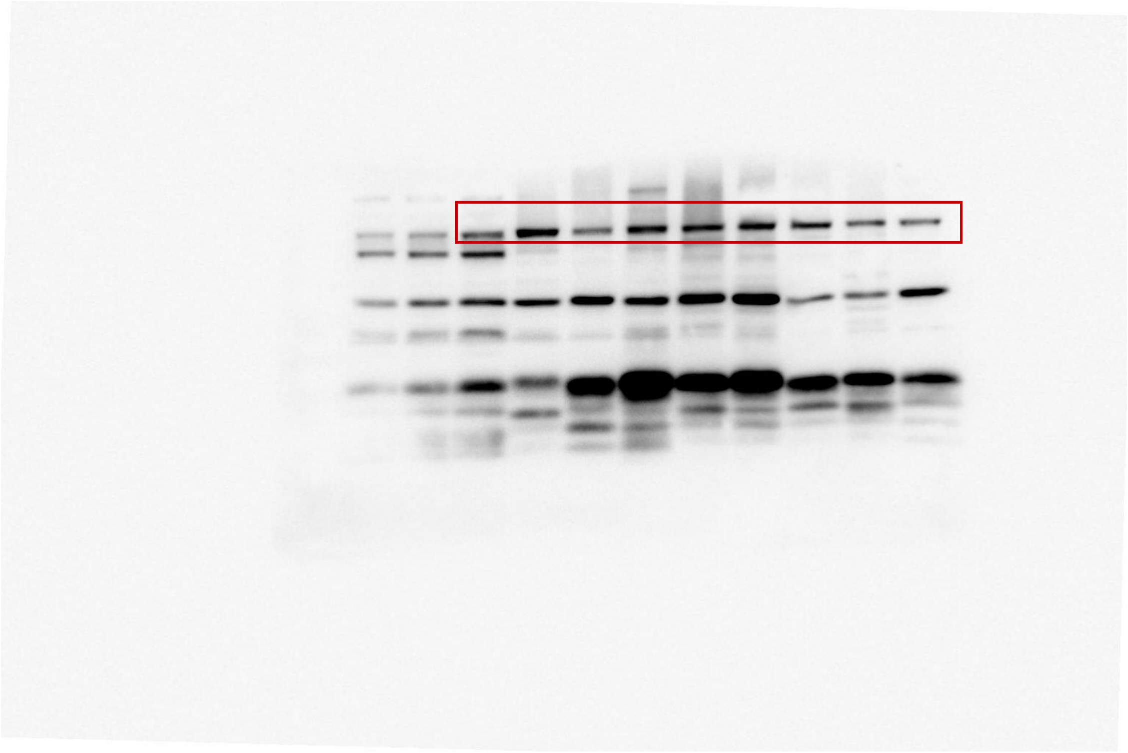

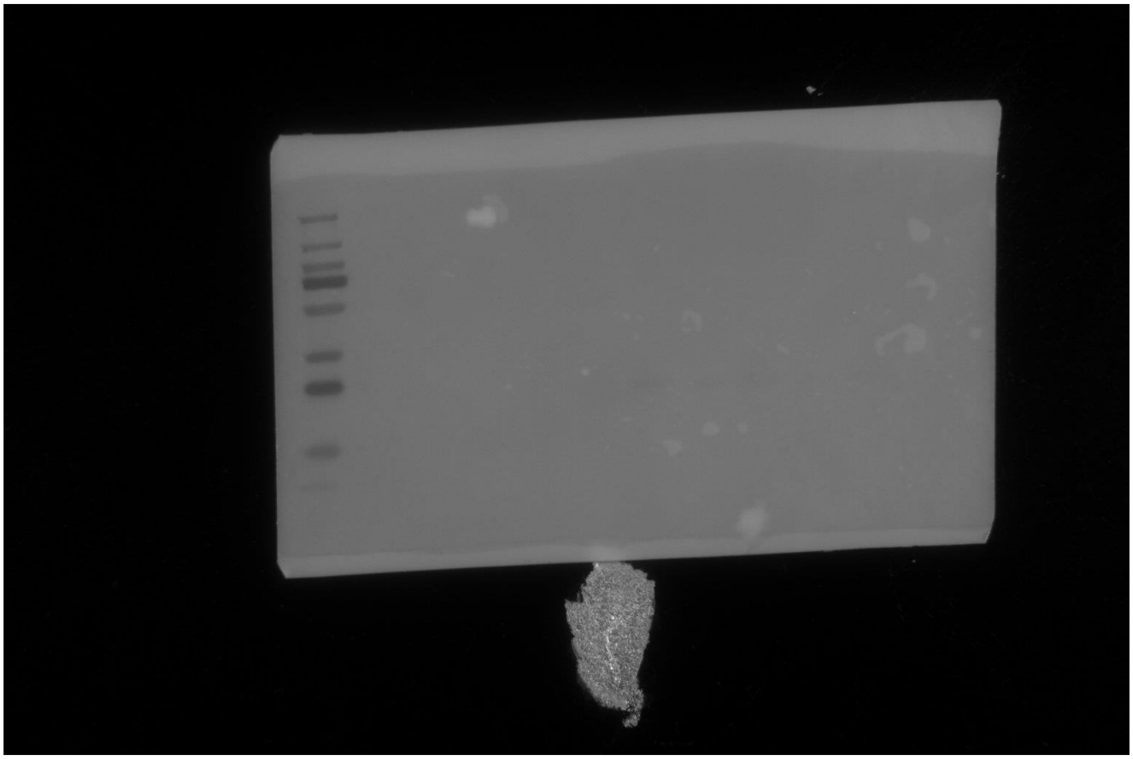

Figure 1C

Caspase 9

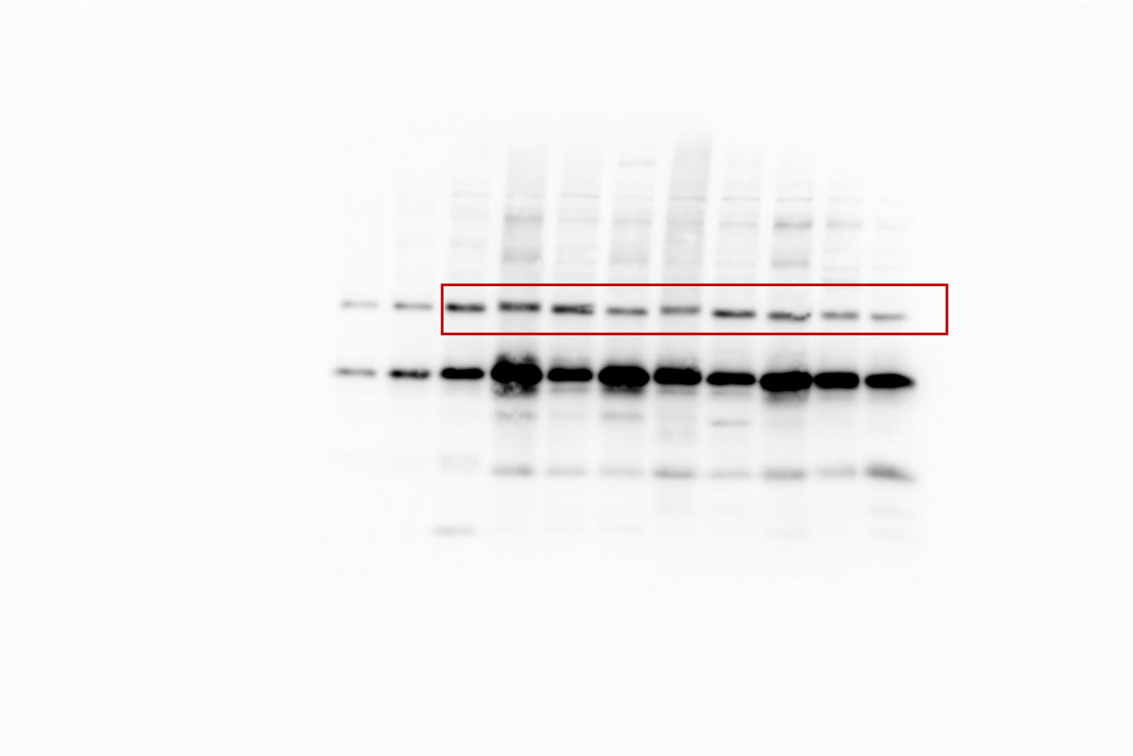

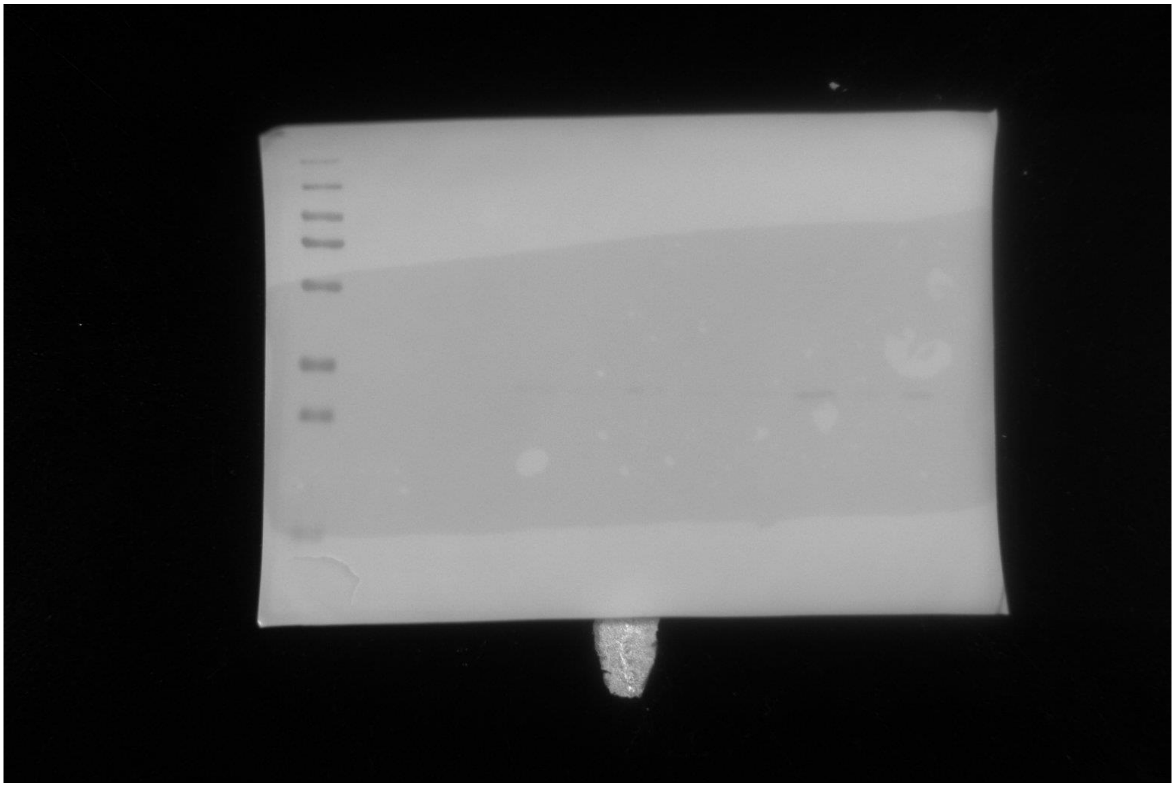

Figure 1C

XIAP

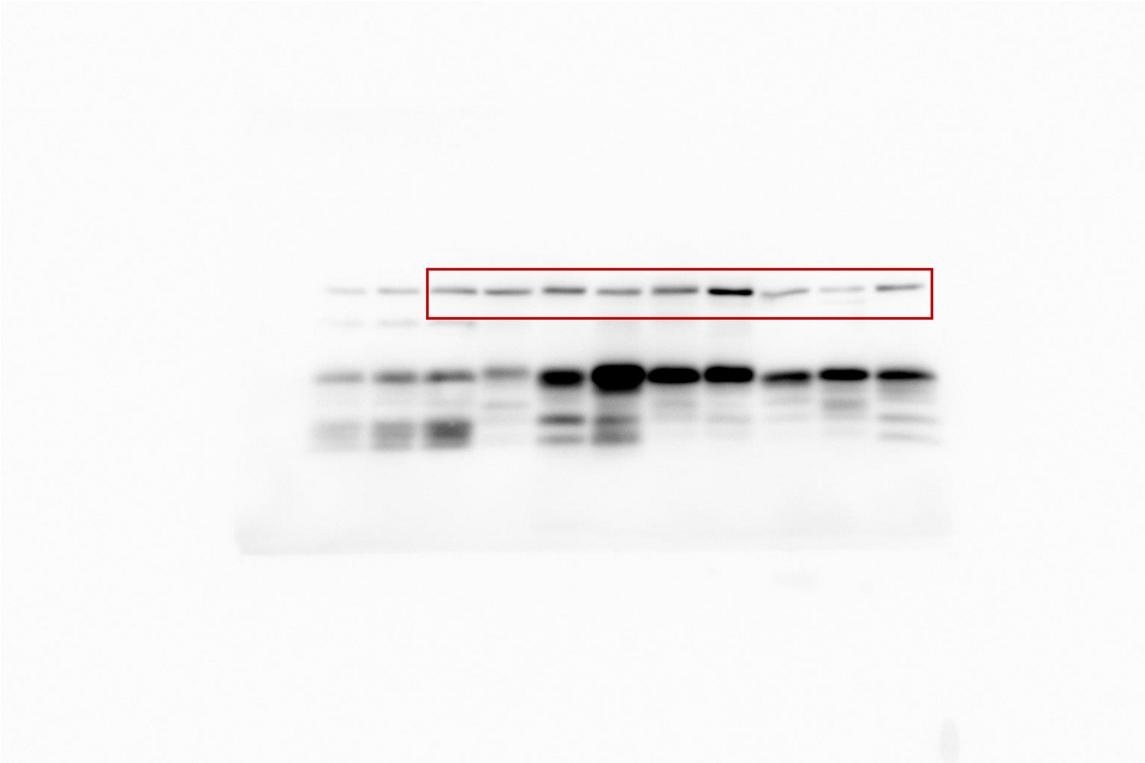

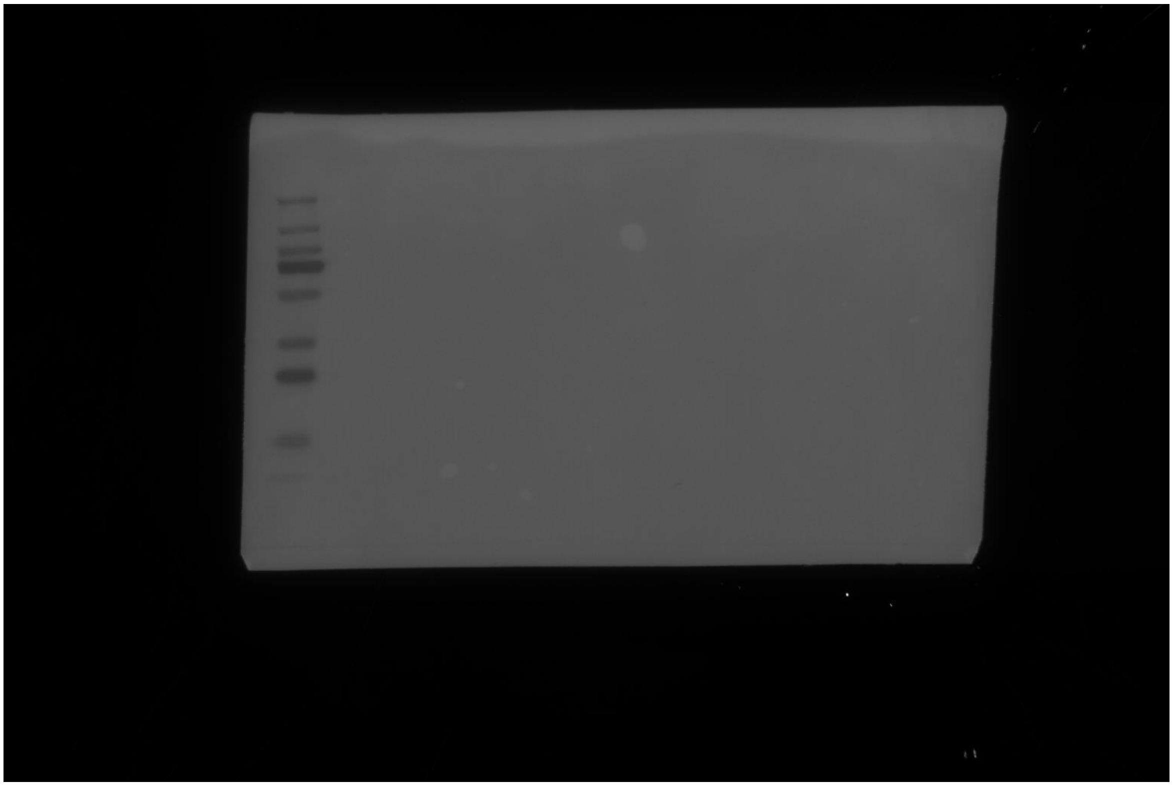

Figure 1C

Caspase 3

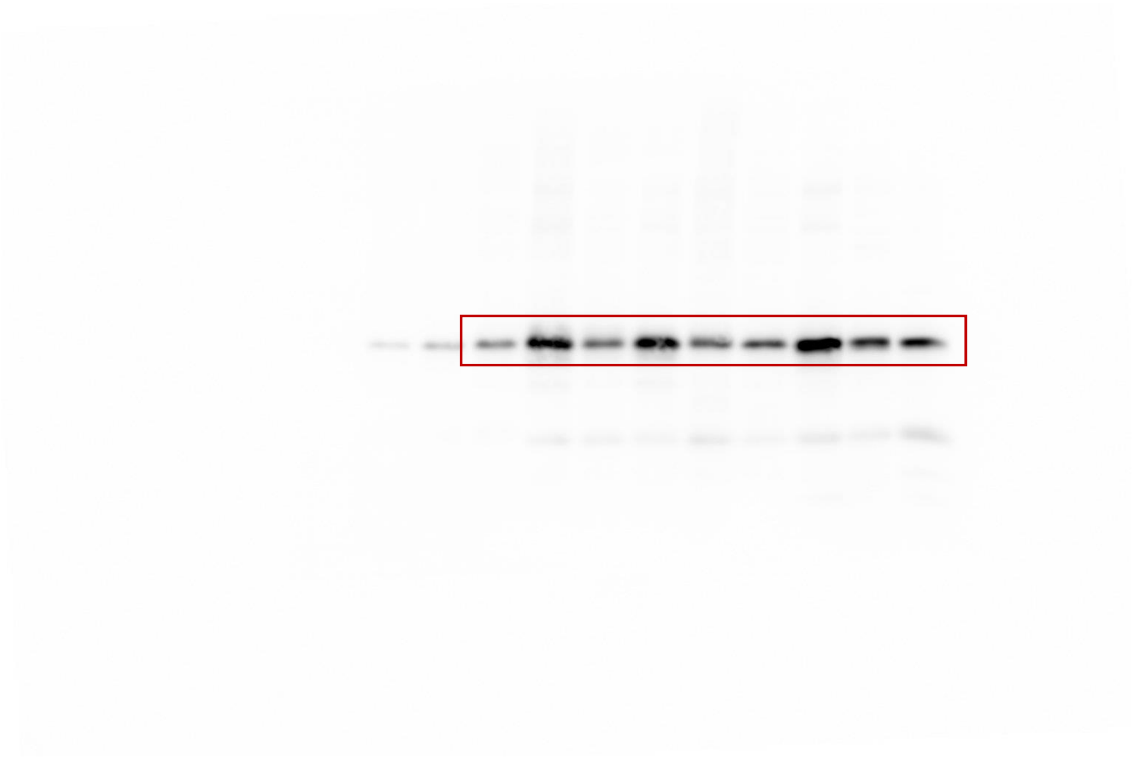

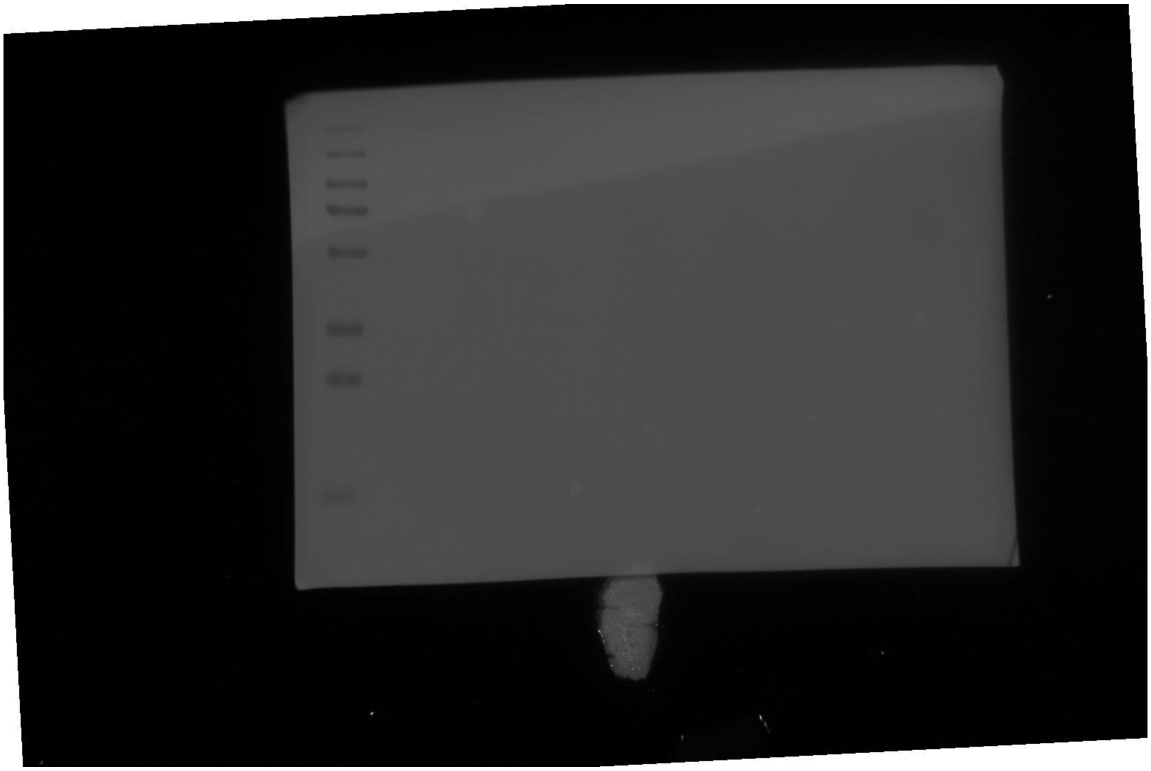

Figure 1C

SMAC

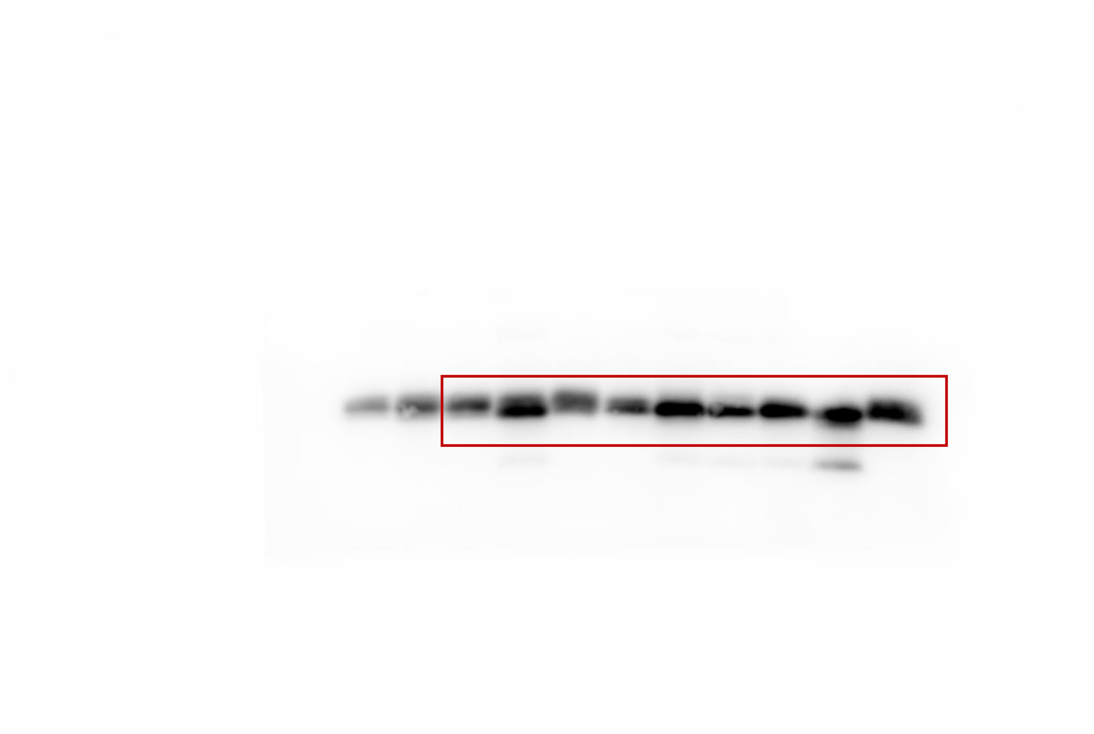

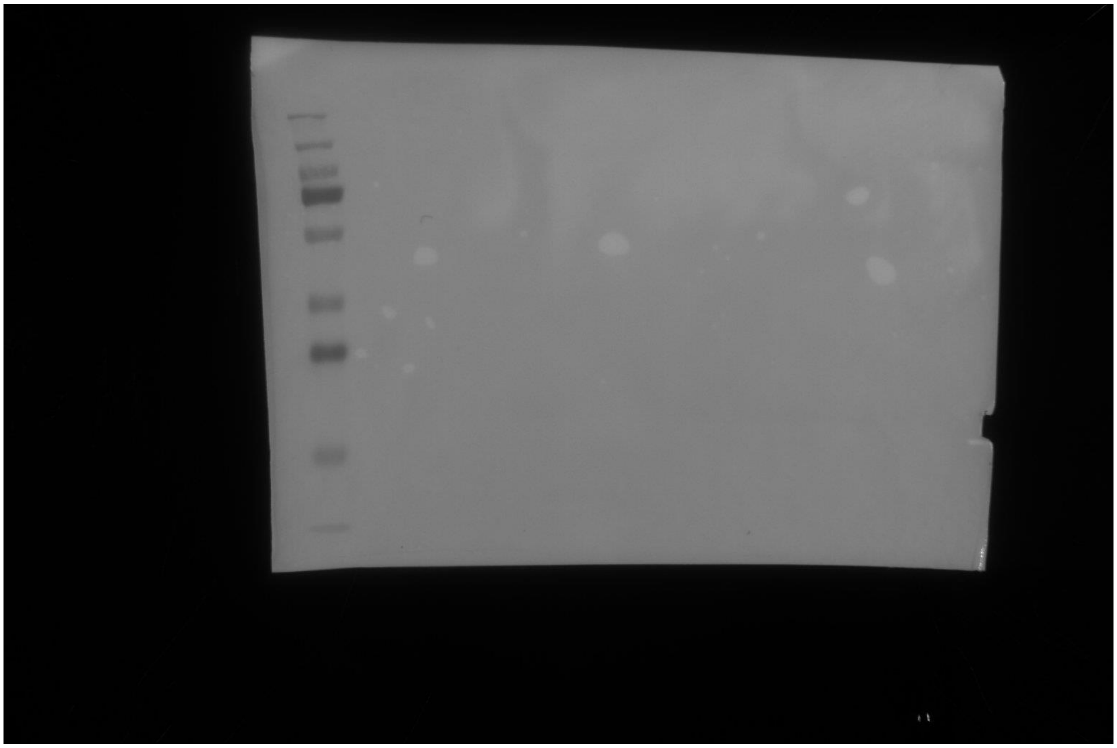

Figure 1C

Loading control

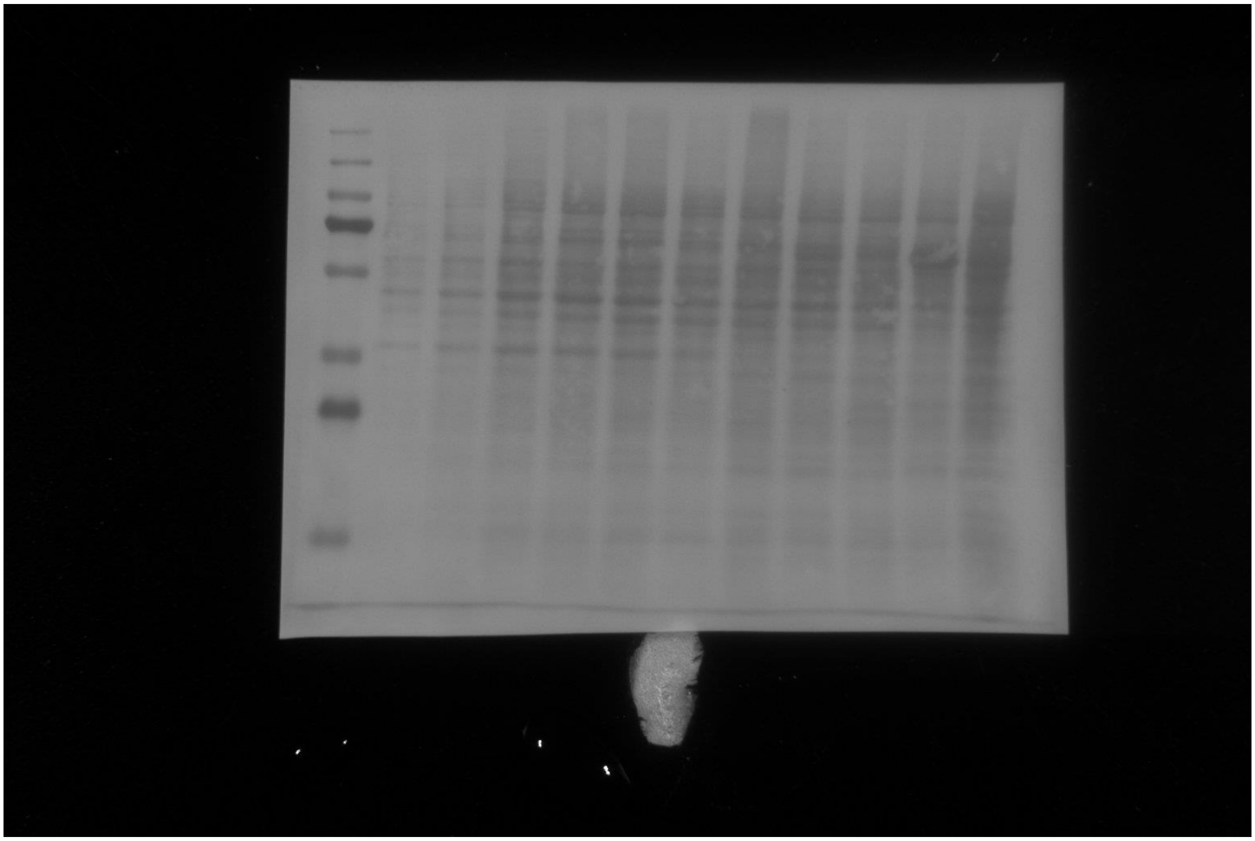

Figure 4G – ONS76

BCL-XL

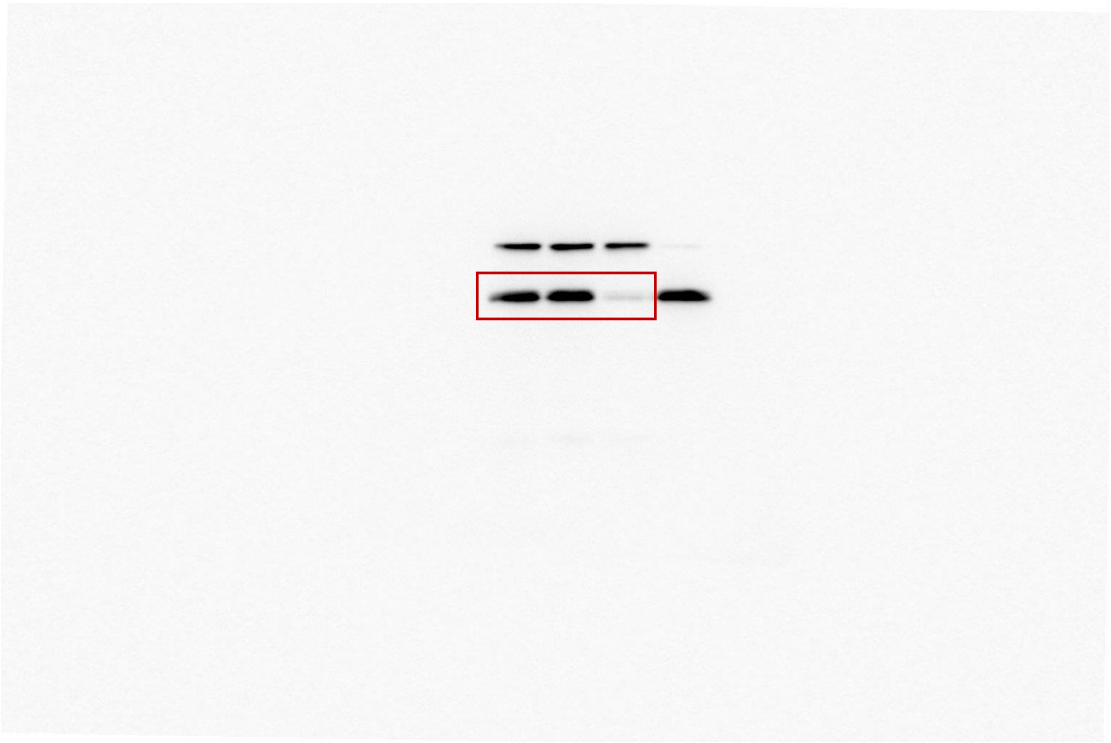

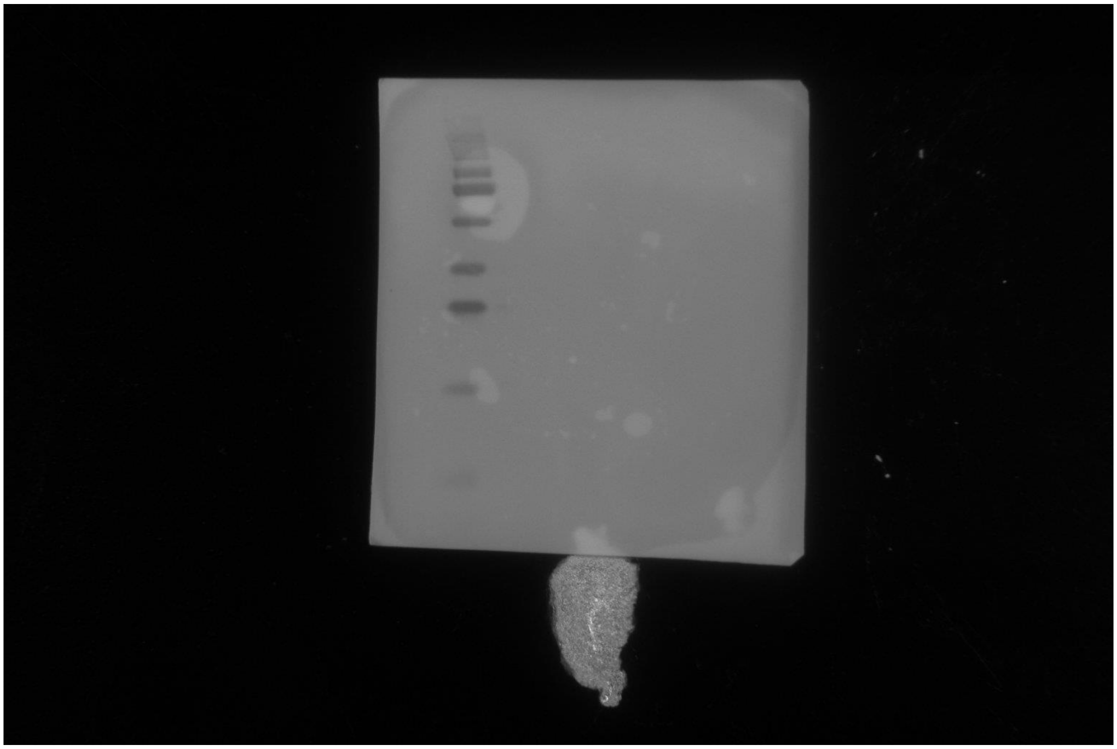

Figure 4G – ONS76

GAPDH

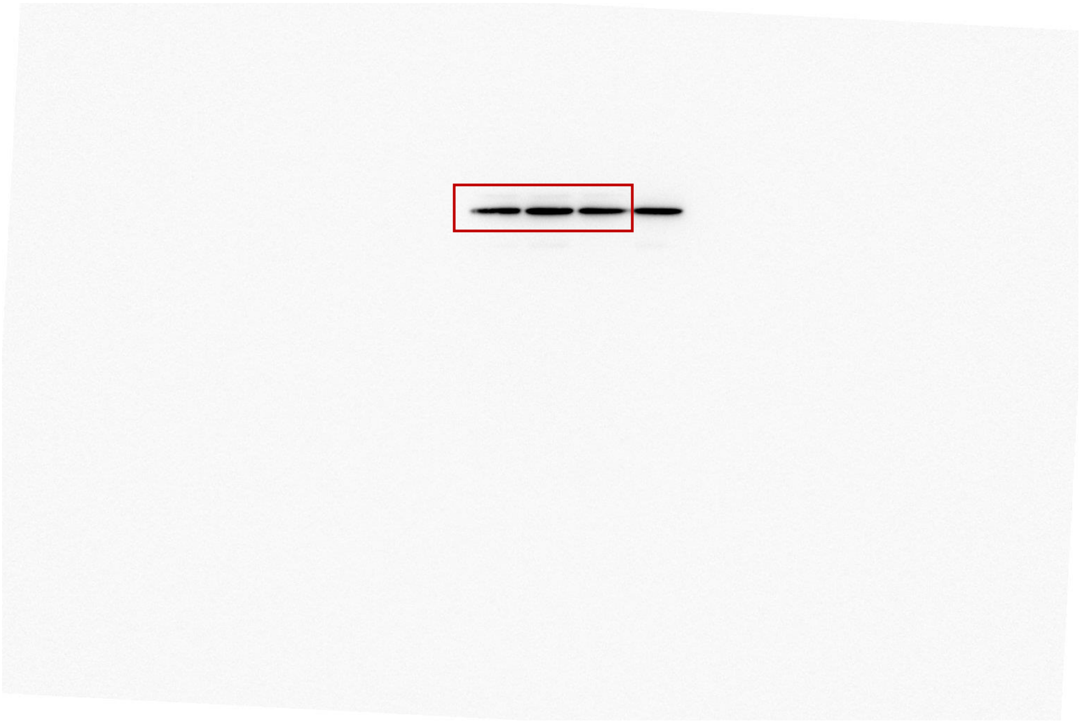

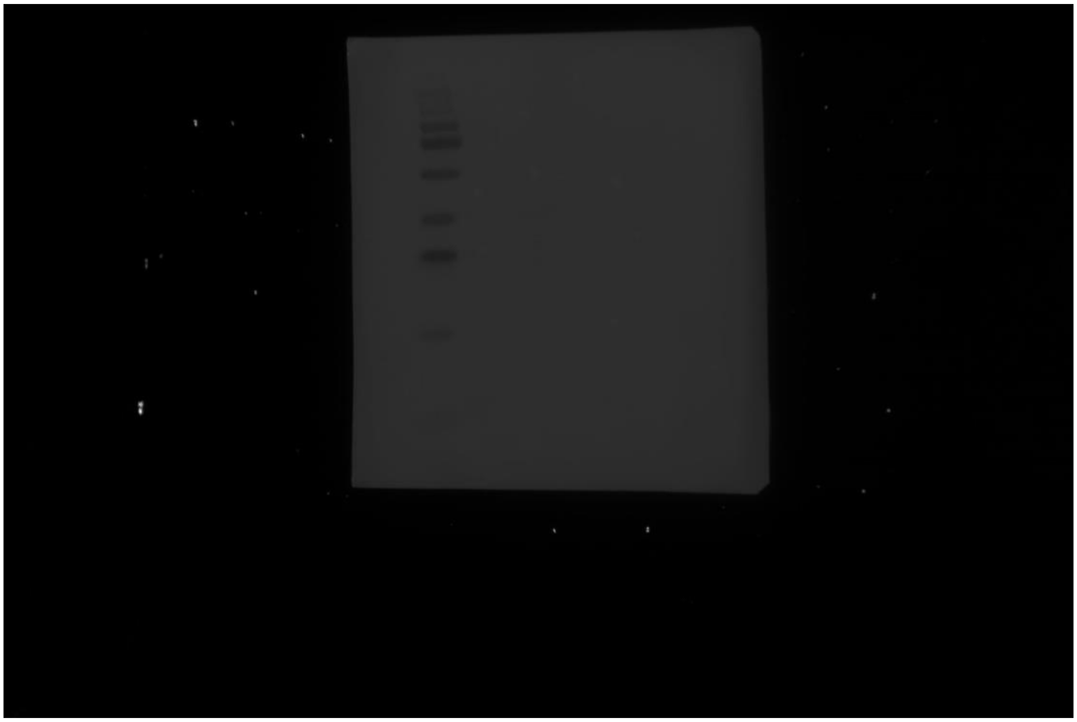

Figure 4H – UW228

BCL-XL

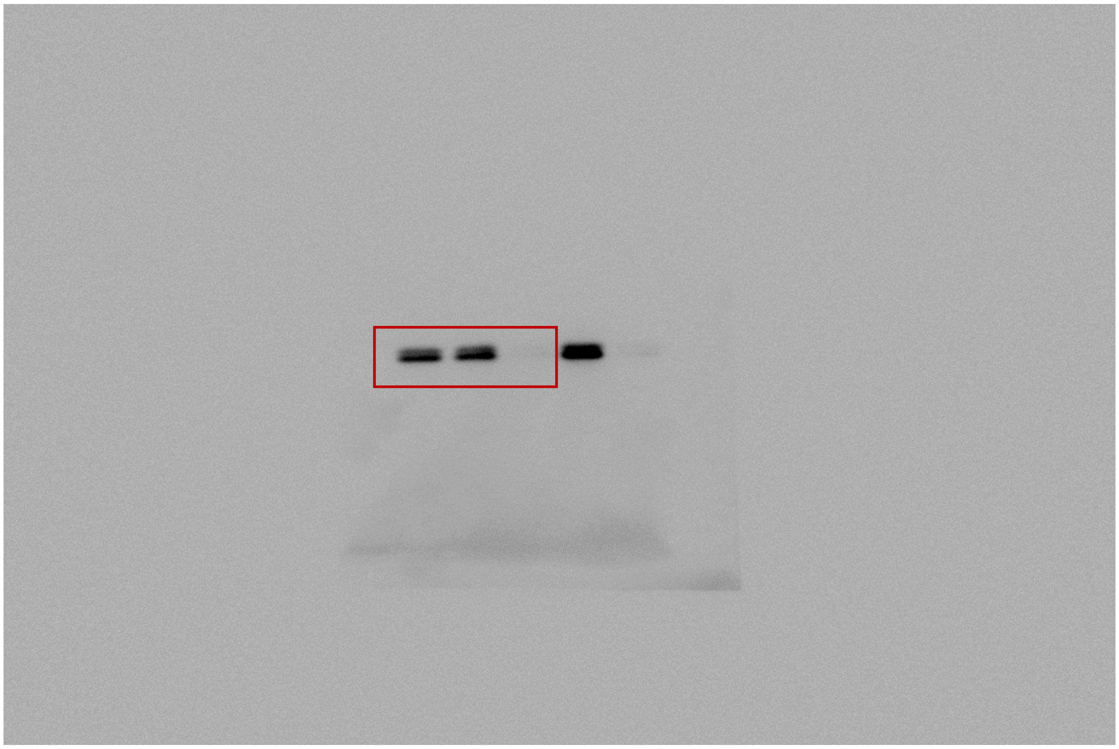

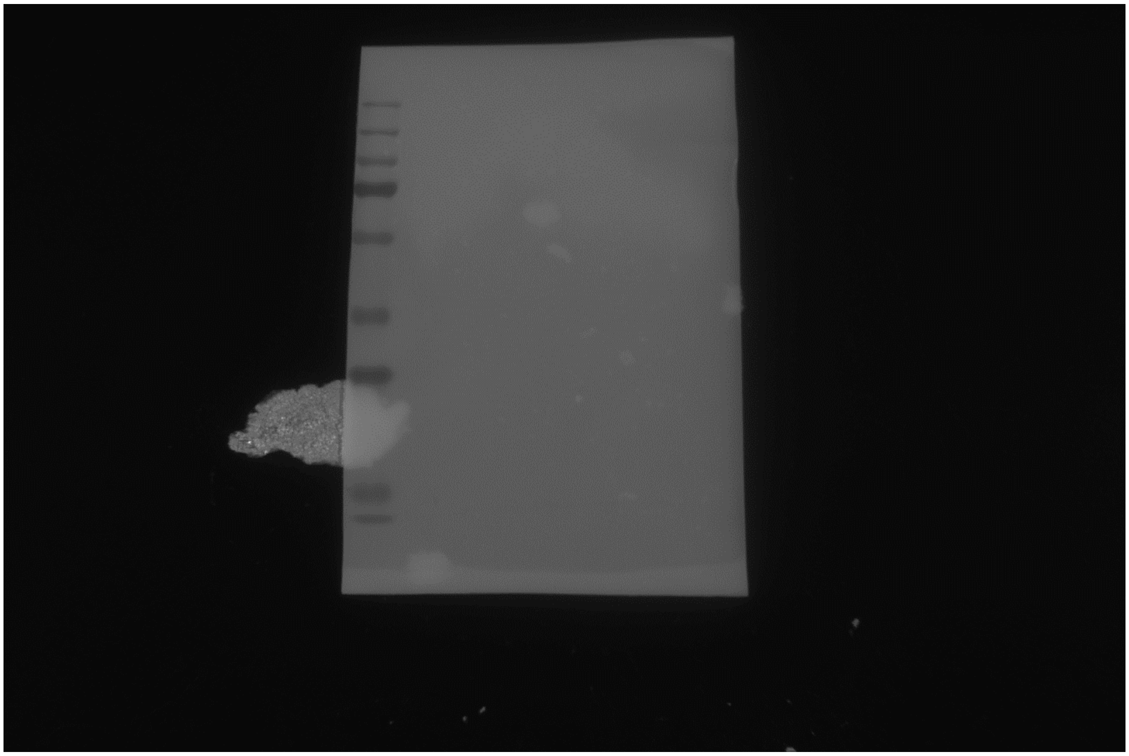

Figure 4H – UW228

GAPDH

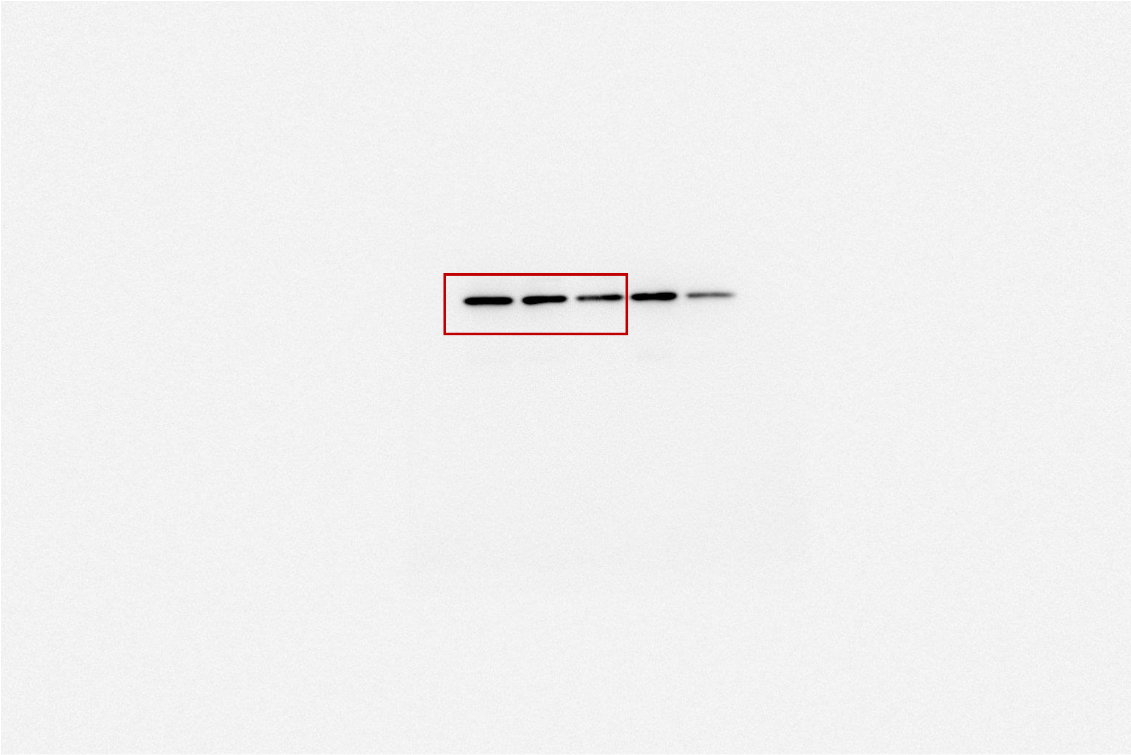

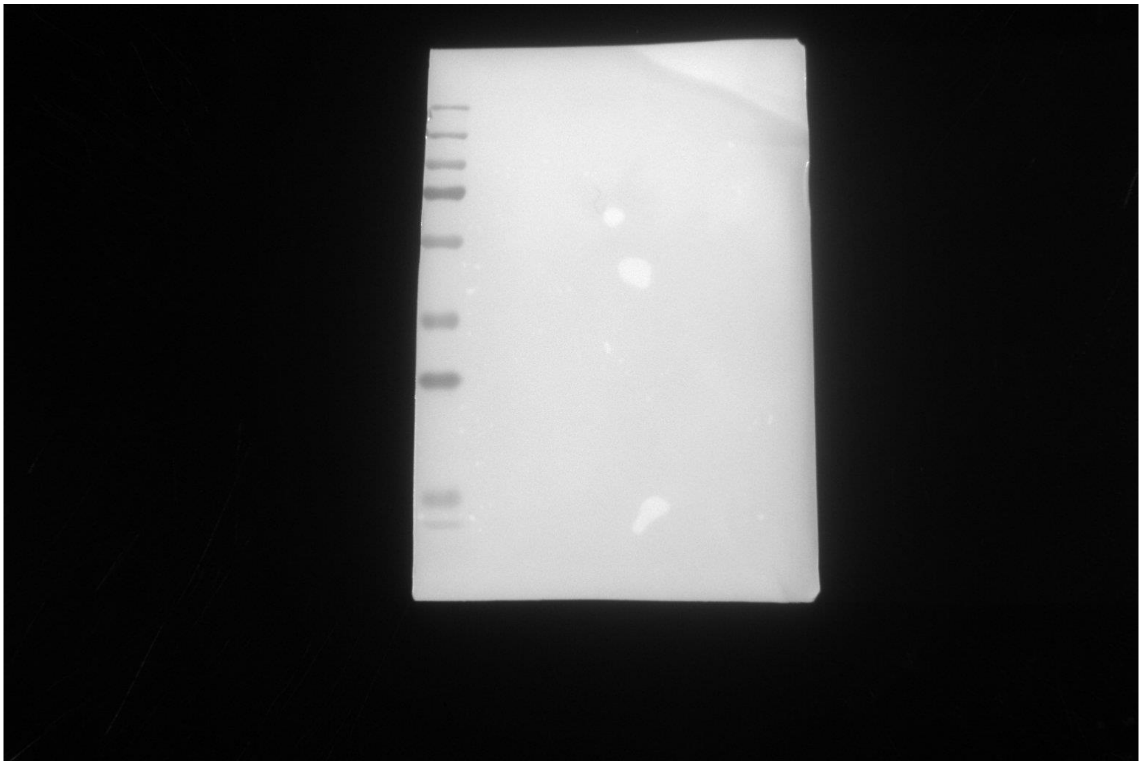

Figure 5A – ONS76

MCL-1

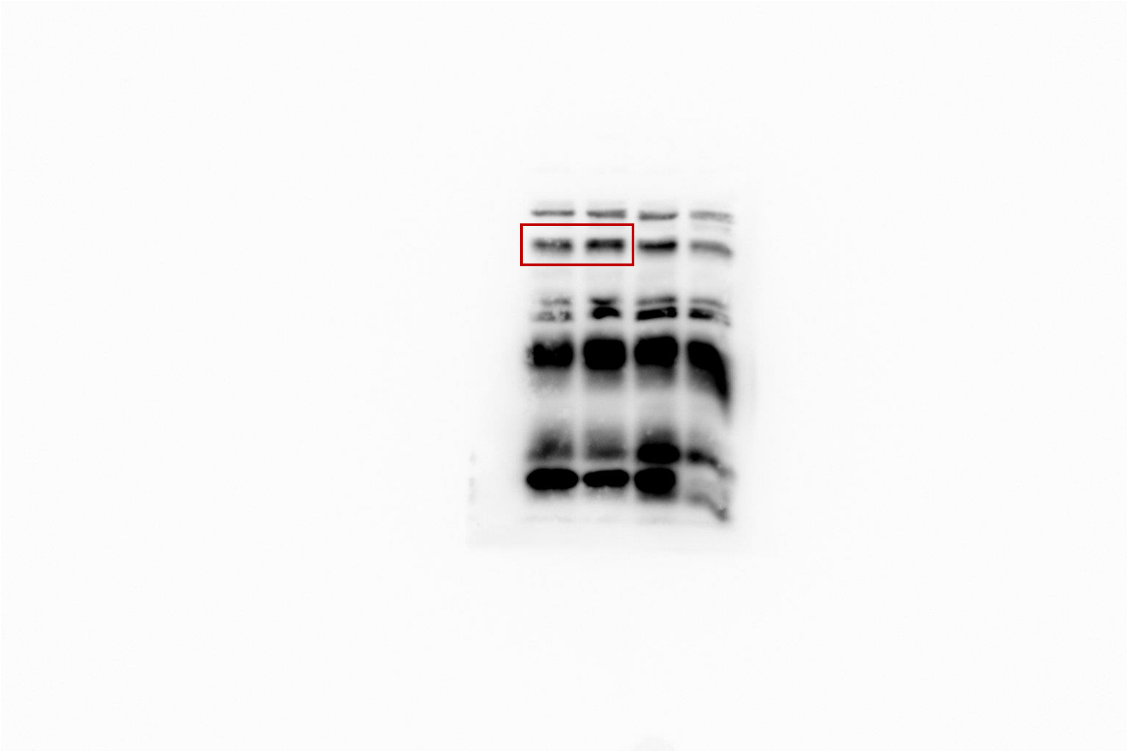

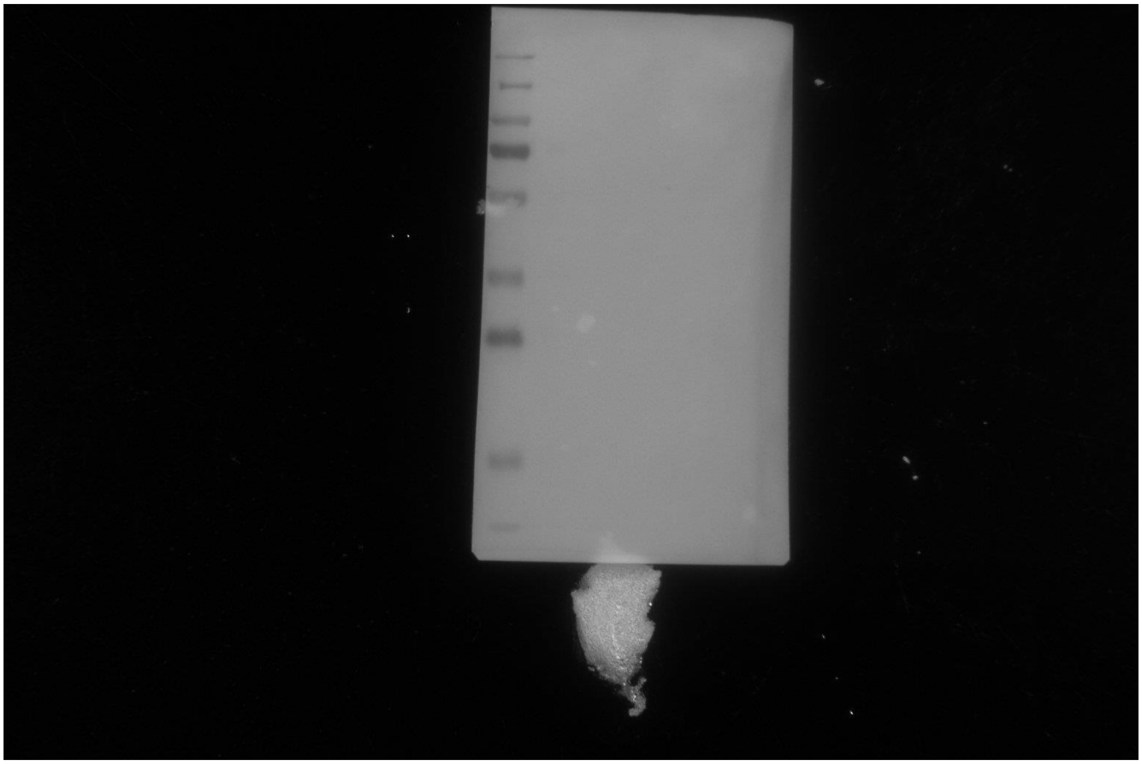

Figure 5A – ONS76

BCL-XL

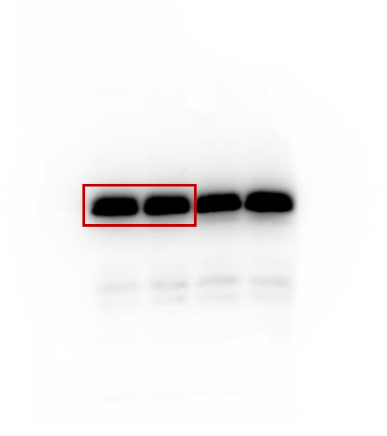

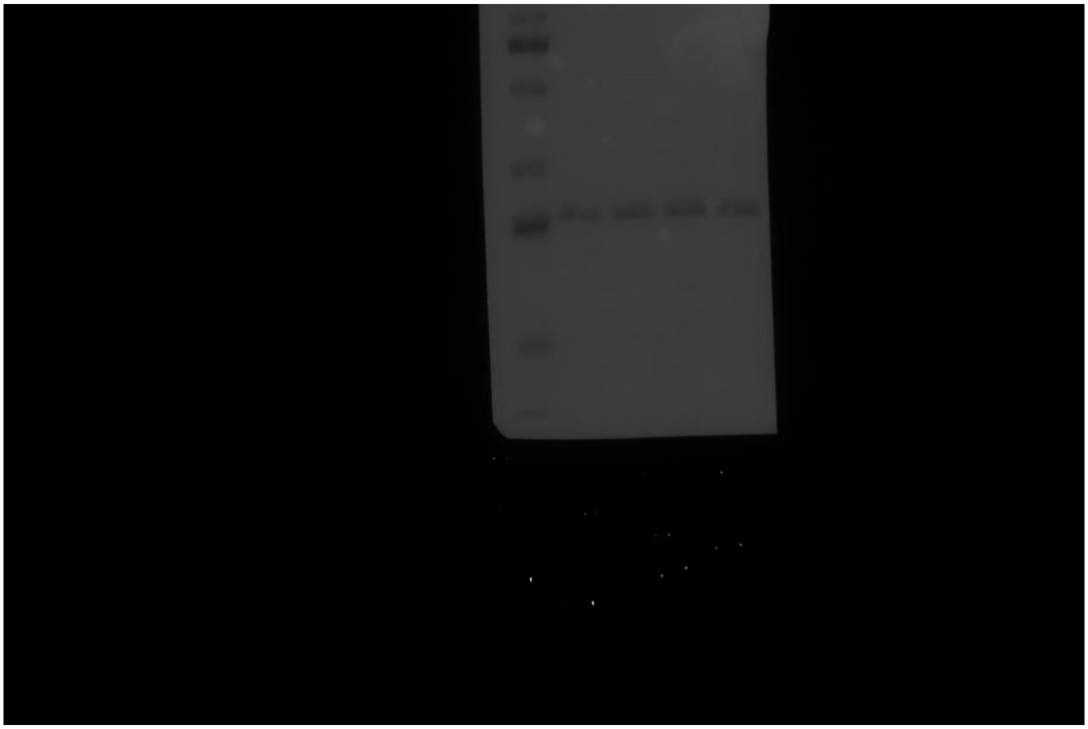

Figure 5A – ONS76

BCL-2

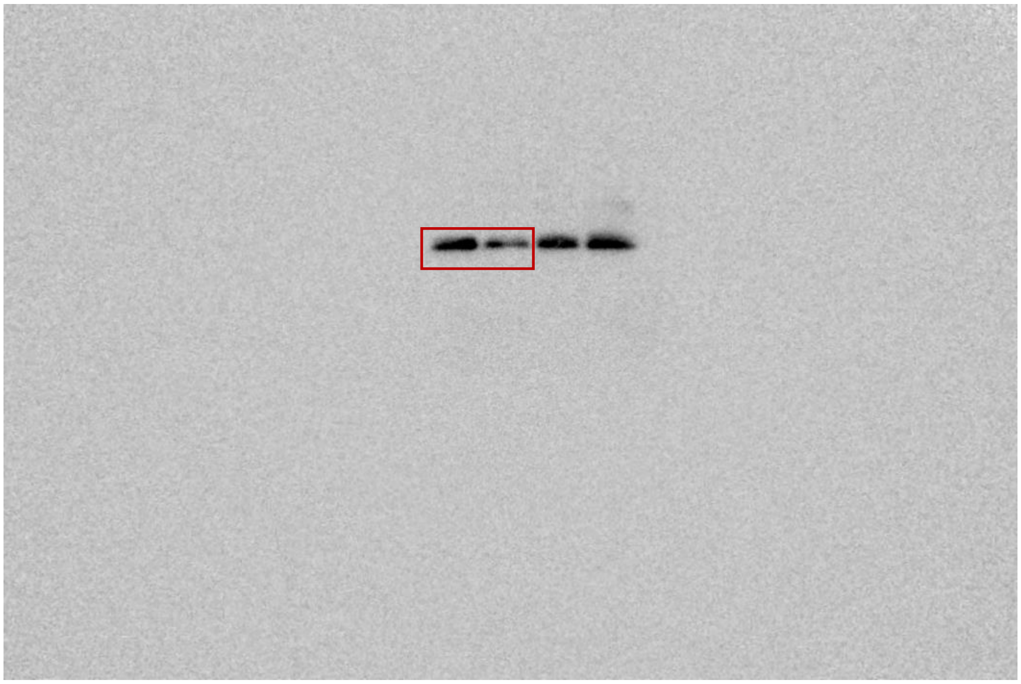

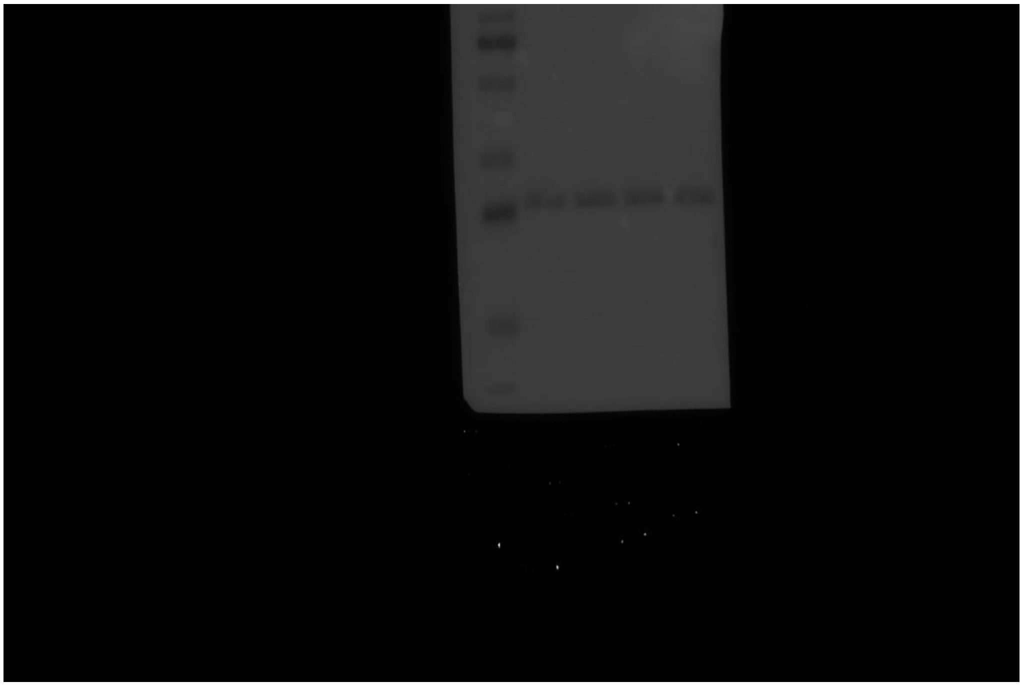

Figure 5A – ONS76

GAPDH

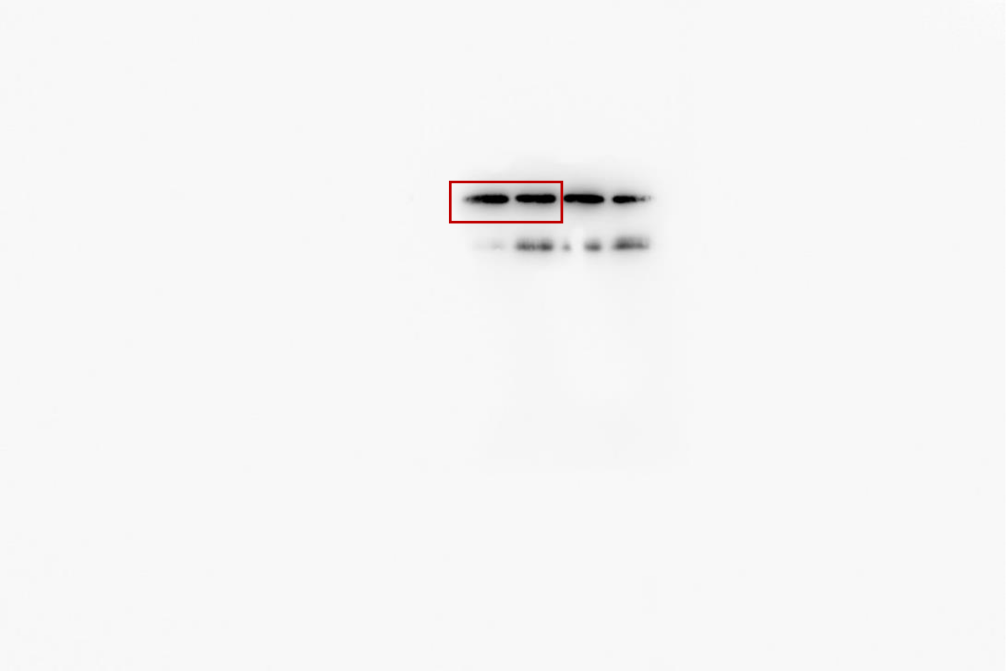

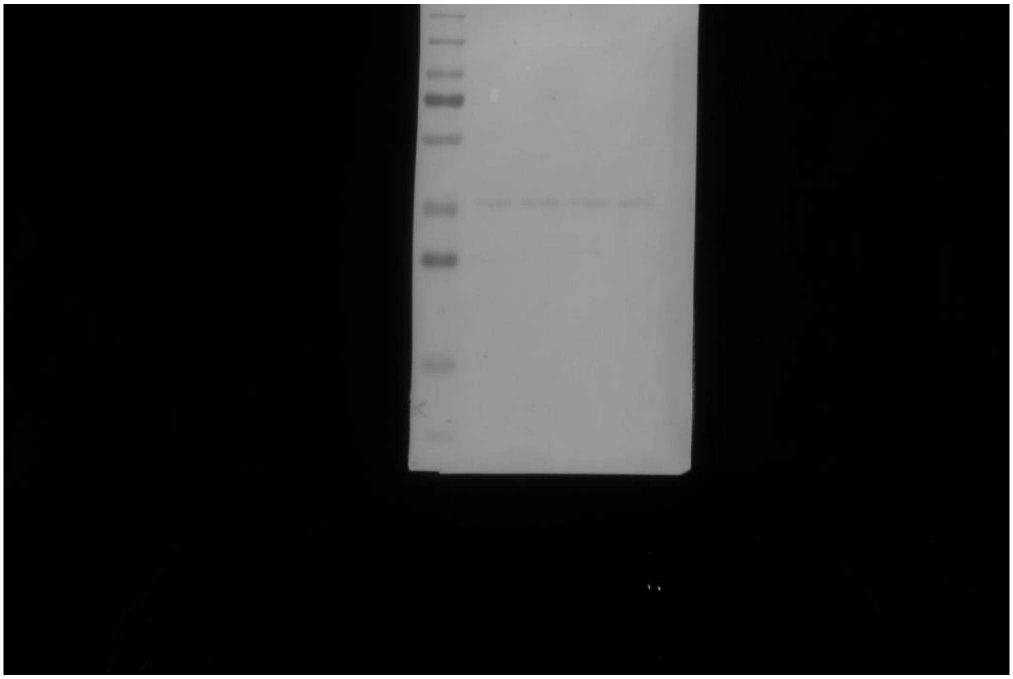

Figure 4B – UW228

MCL-1

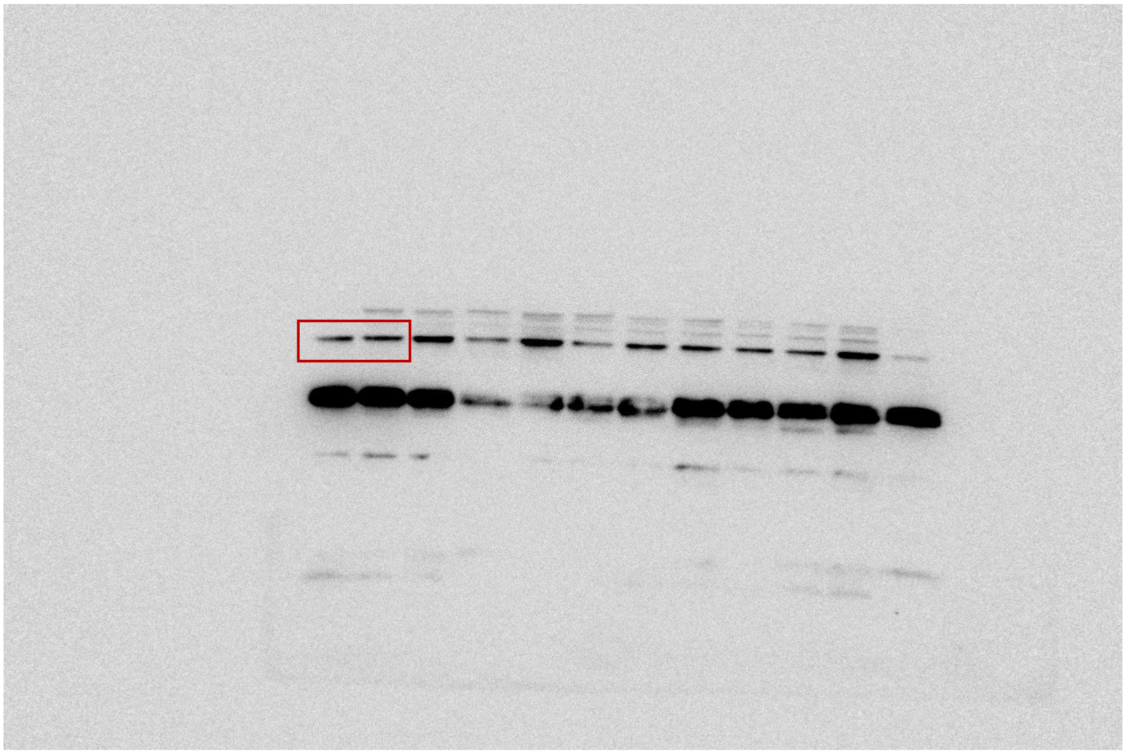

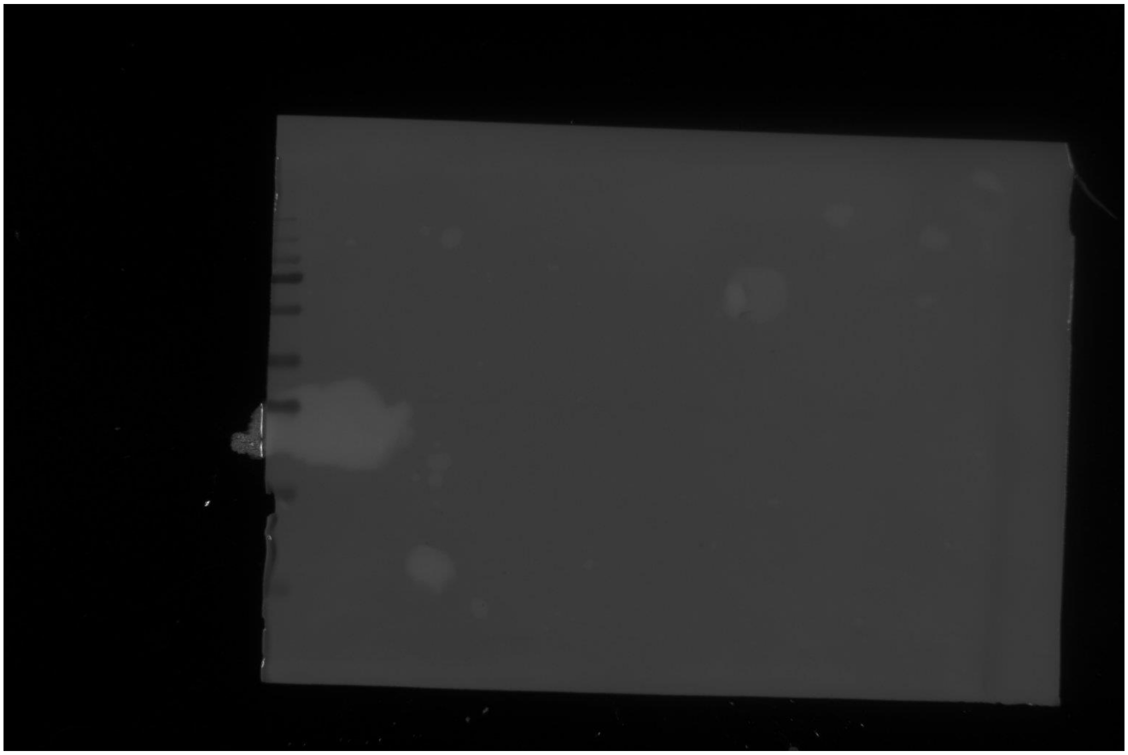

Figure 4B – UW228

BCL-XL

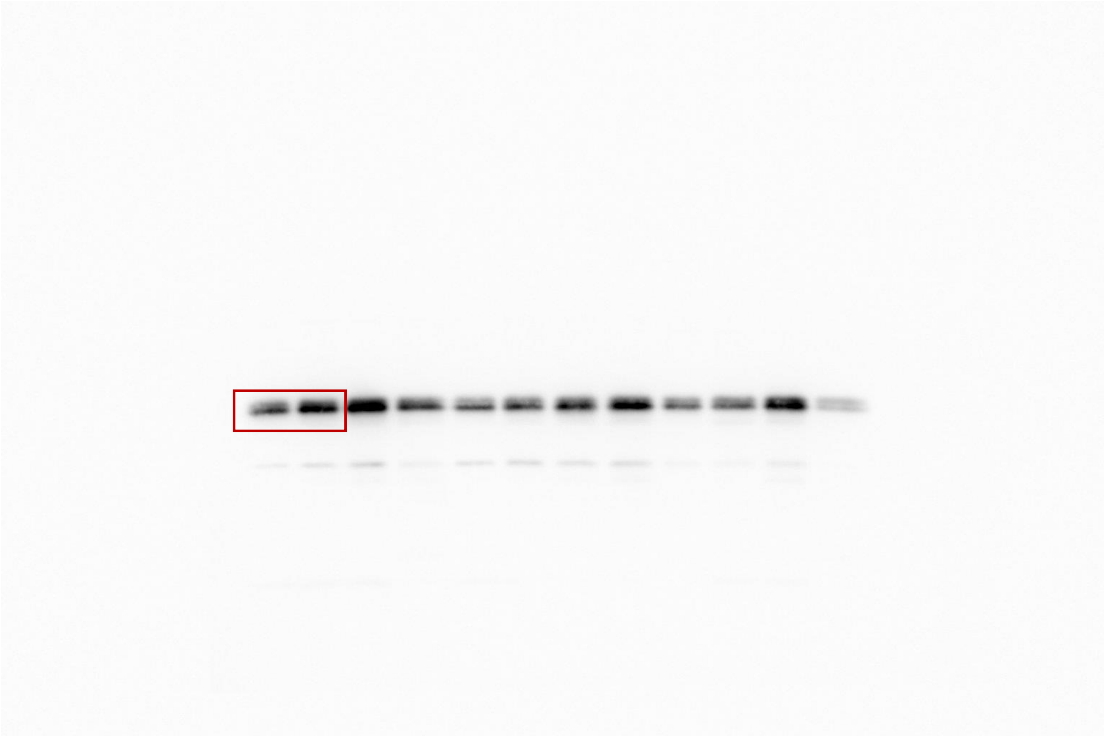

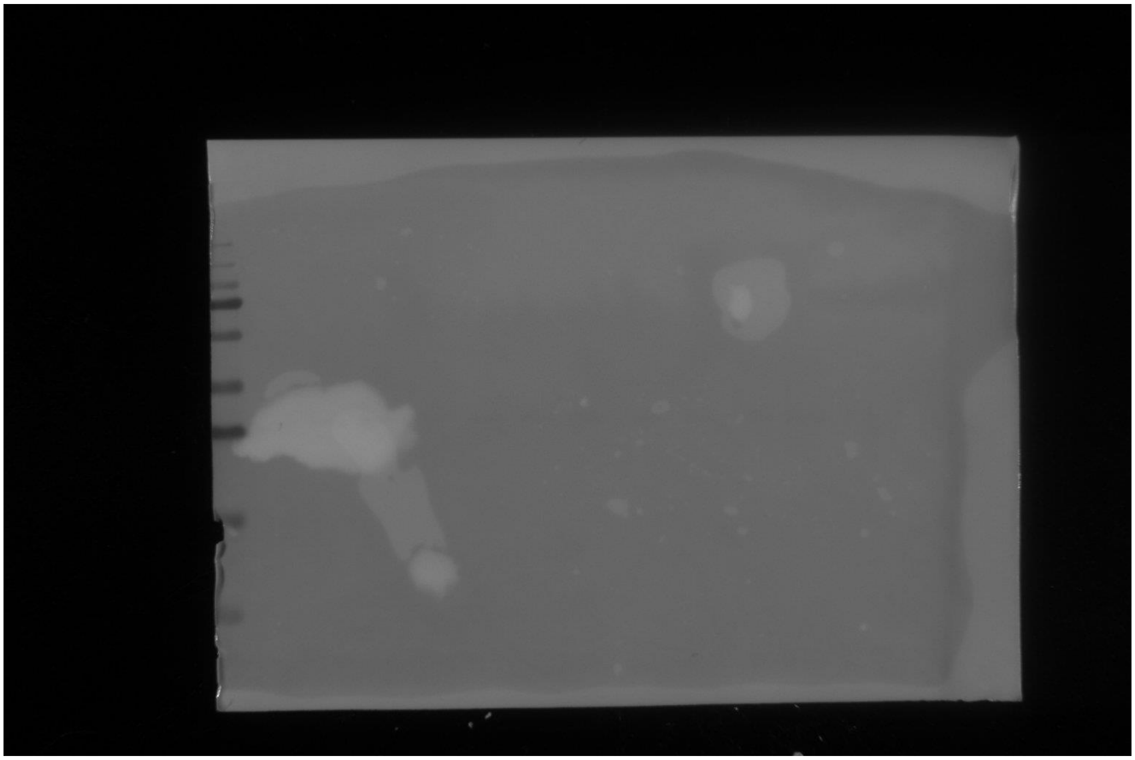

Figure 4B – UW228

BCL-2

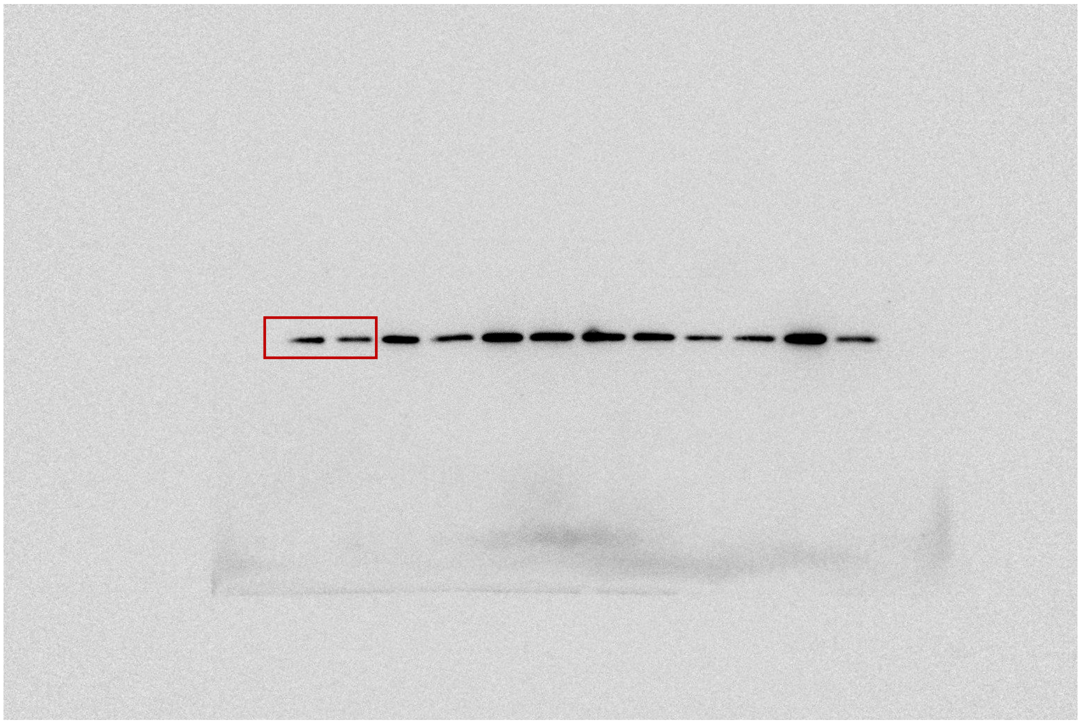

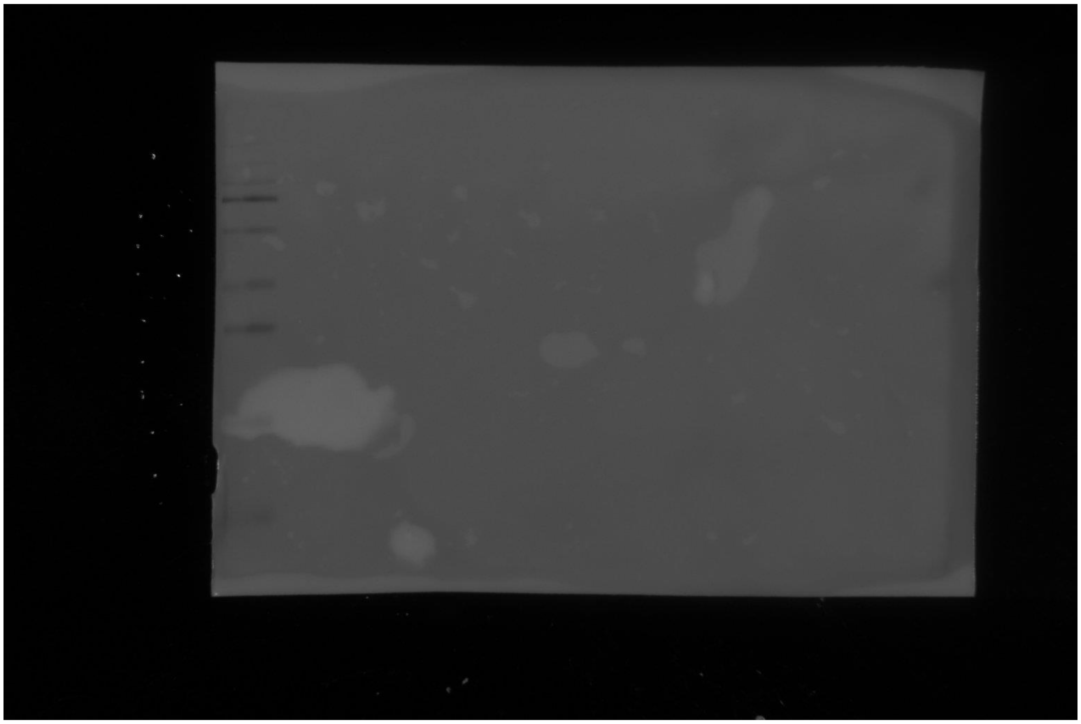

Figure 4B – UW228

GAPDH

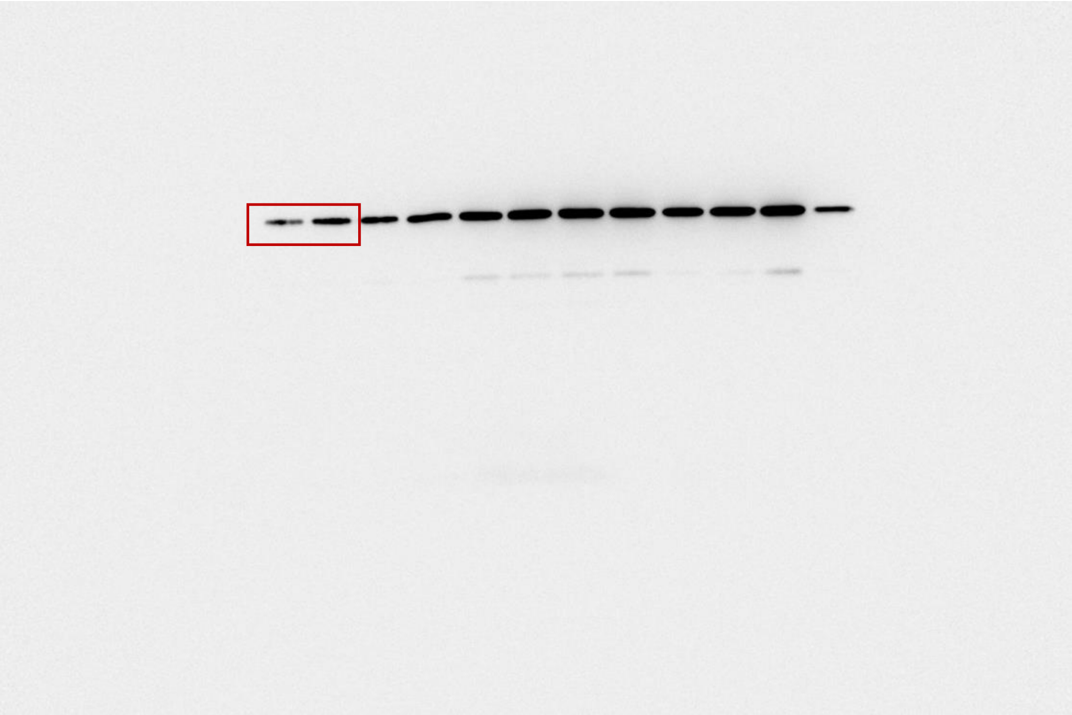

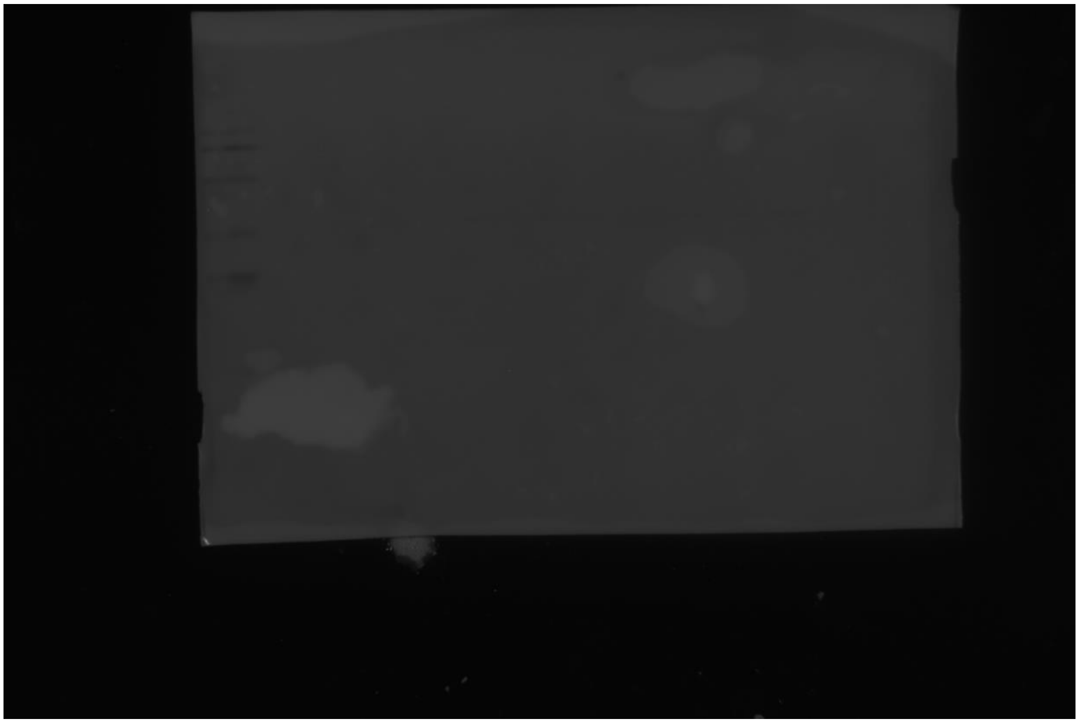

Figure 5E - IP – ONS76

BCL-XL

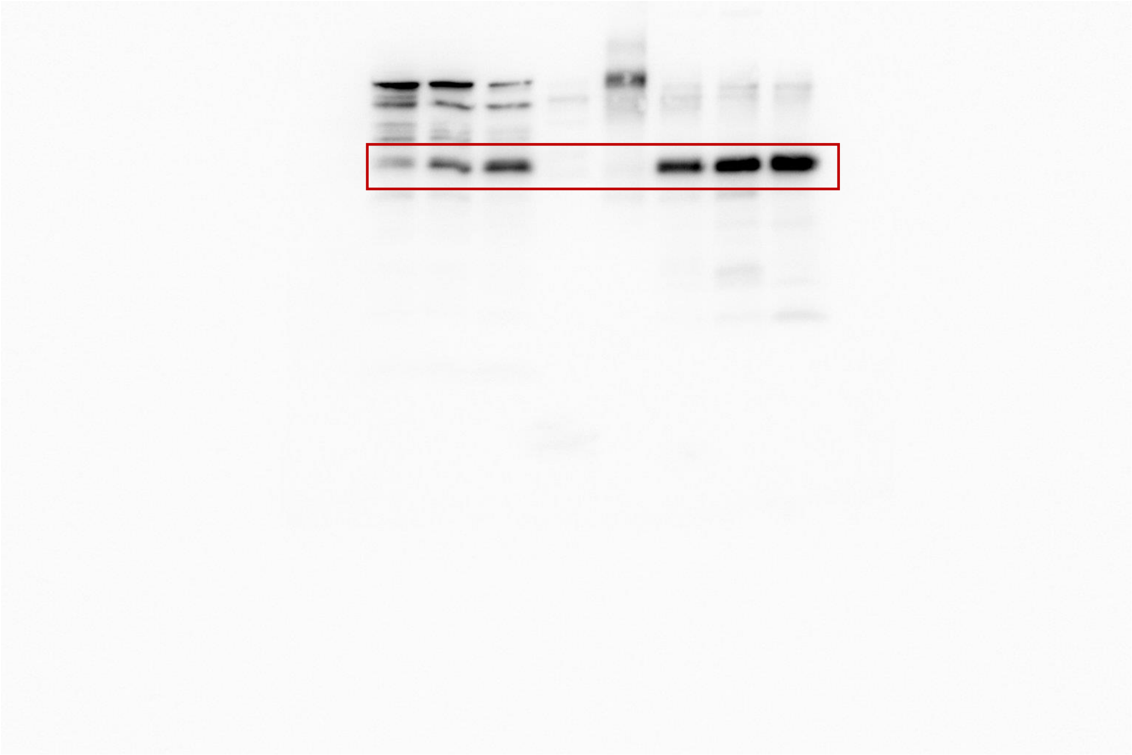

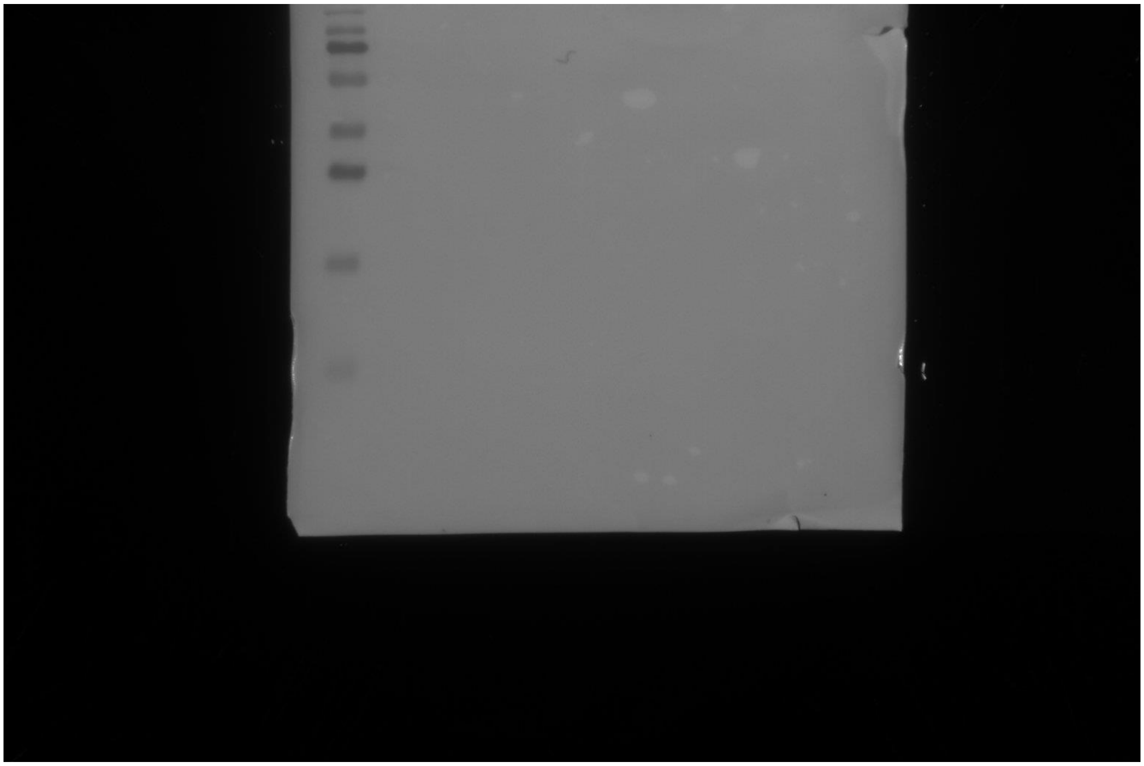

Figure 5E - IP – ONS76

BAK

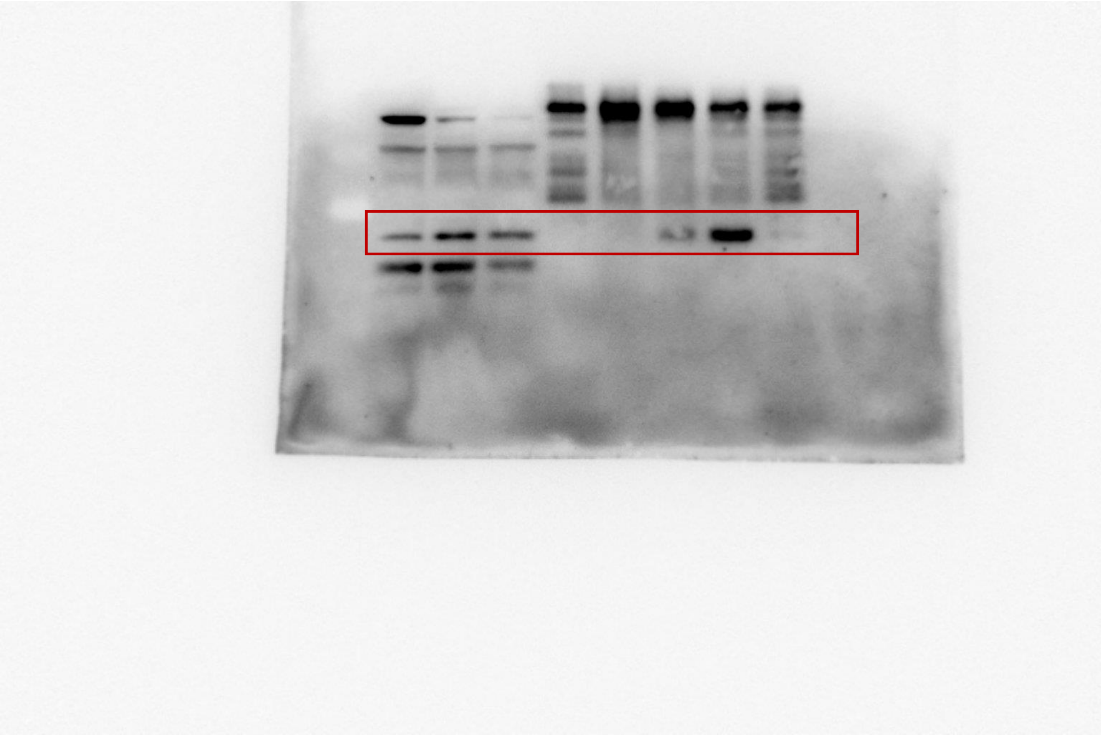

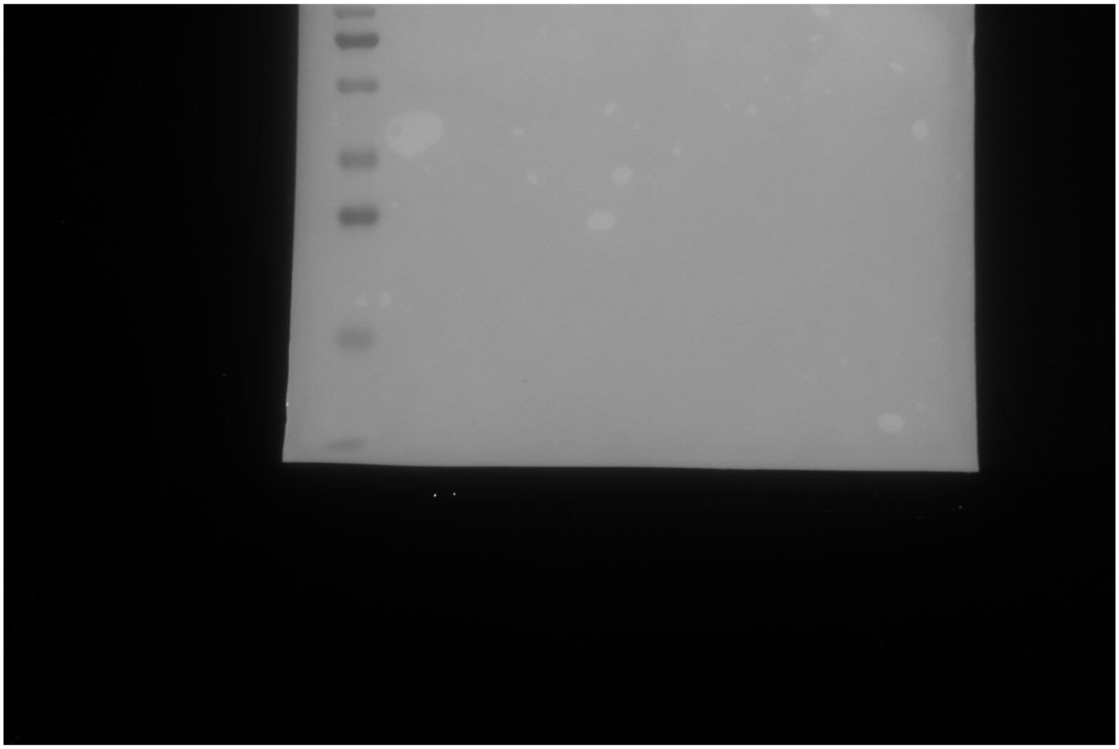

Figure 5E - IP – ONS76

BAX

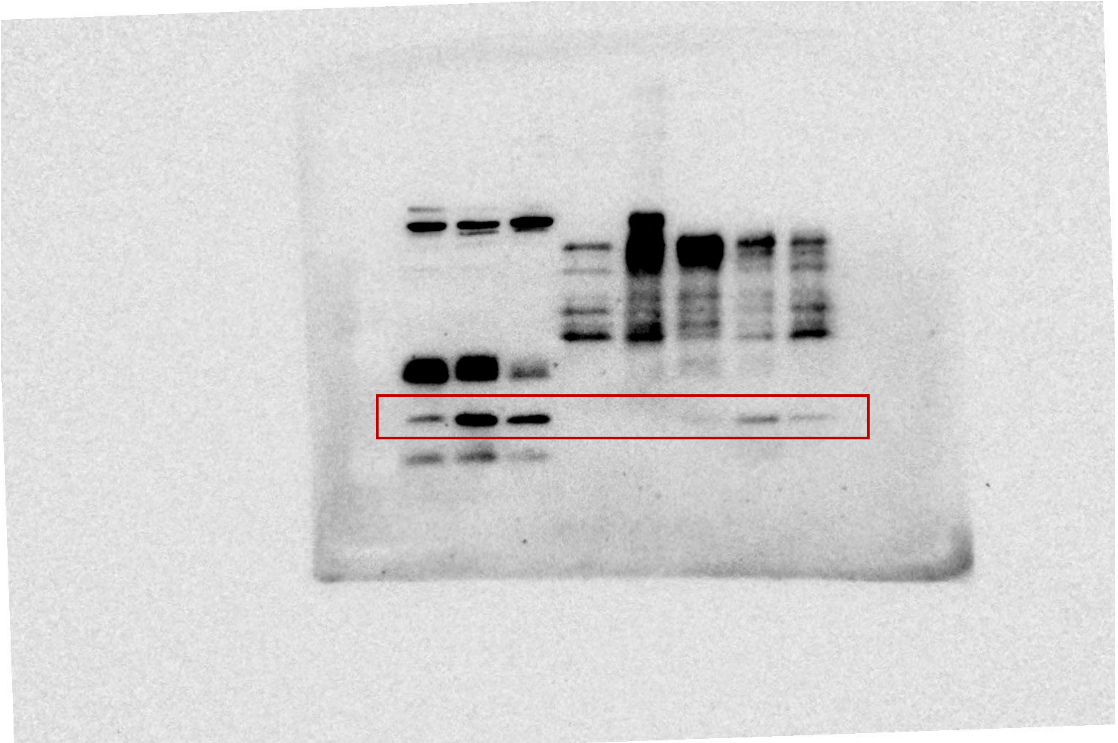

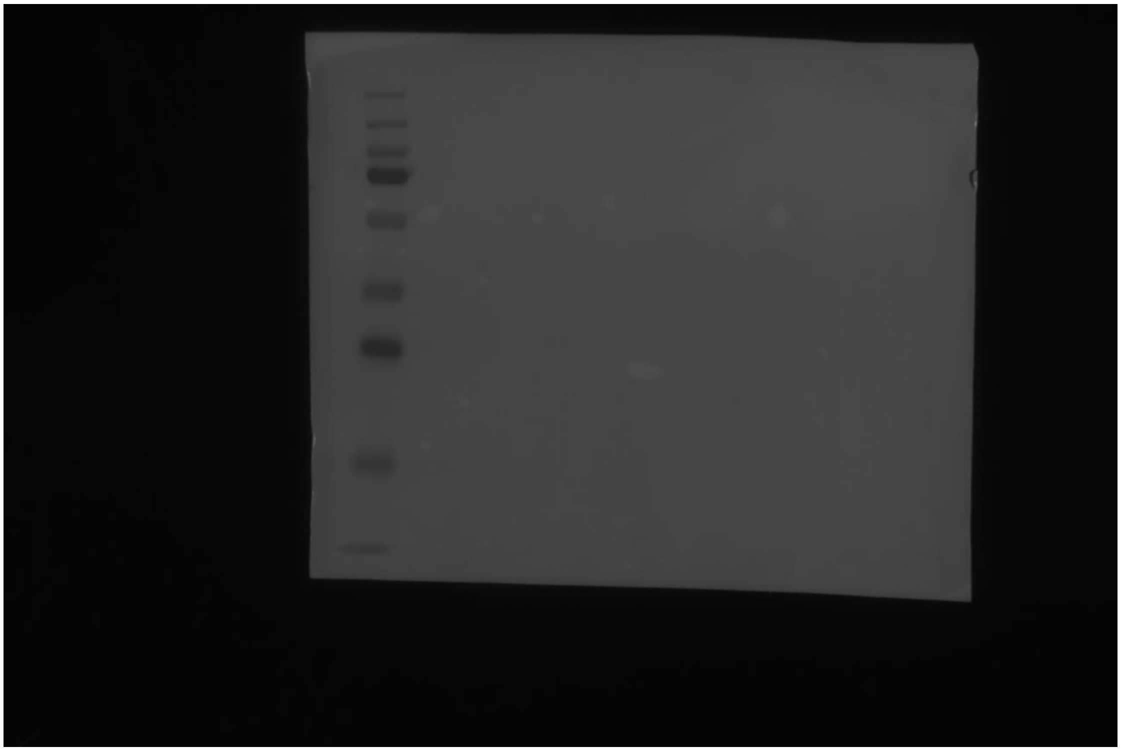

Figure 5E - IP – ONS76

BIM

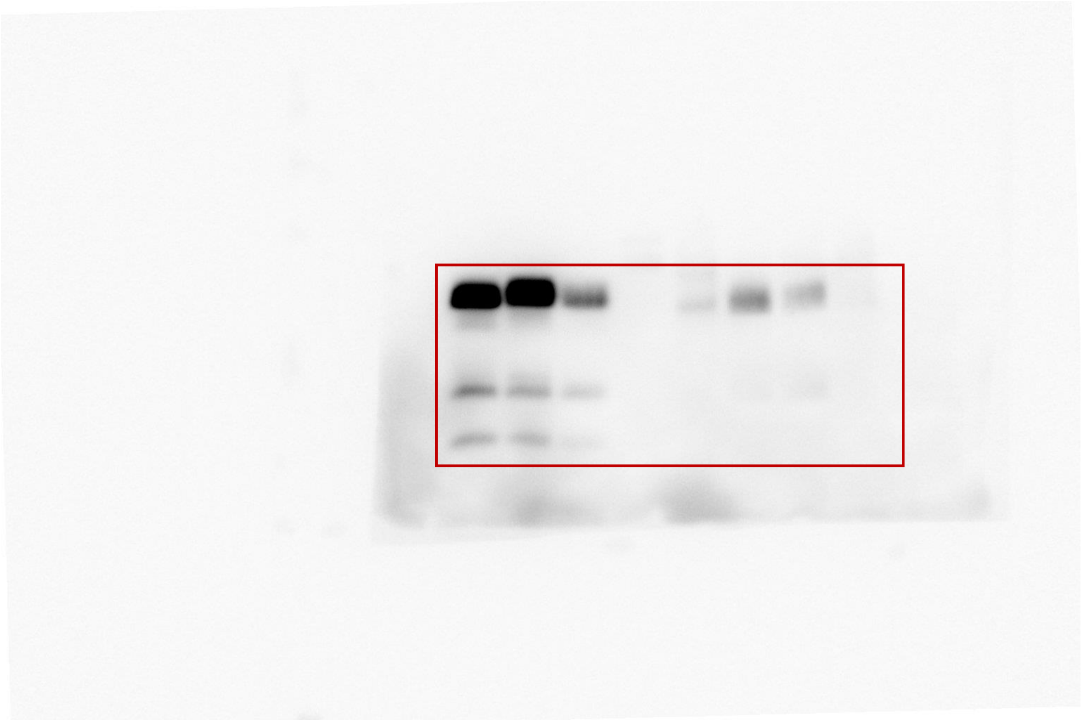

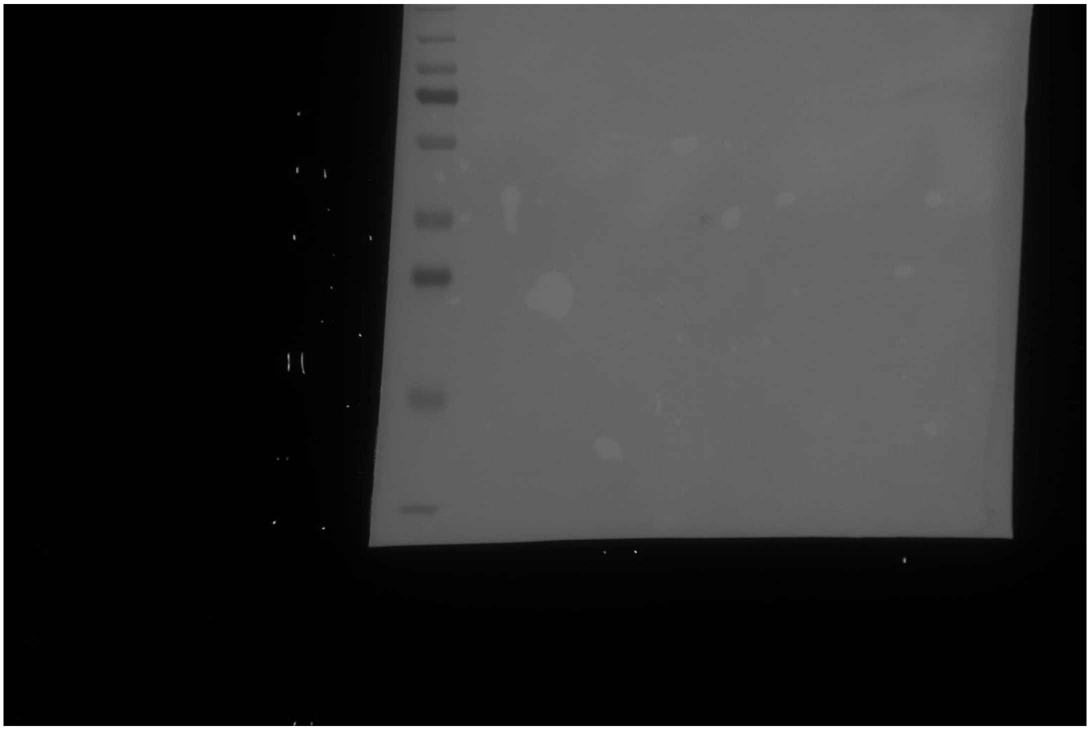

Figure 5E - IP – ONS76

PUMA

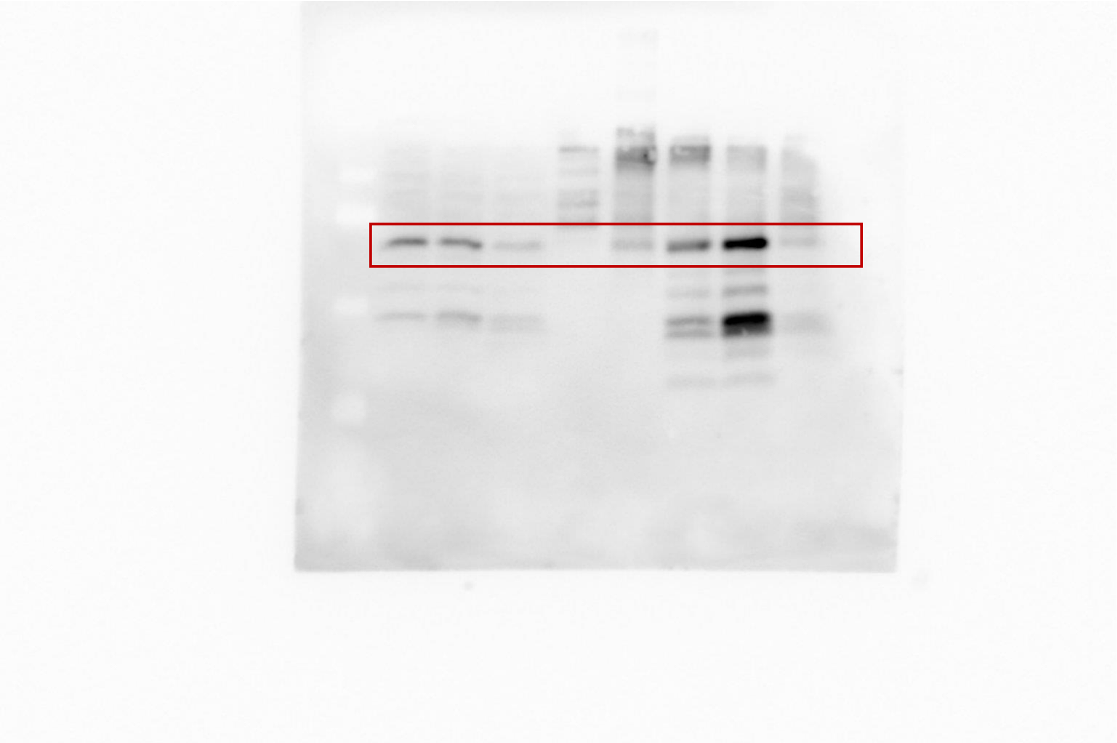

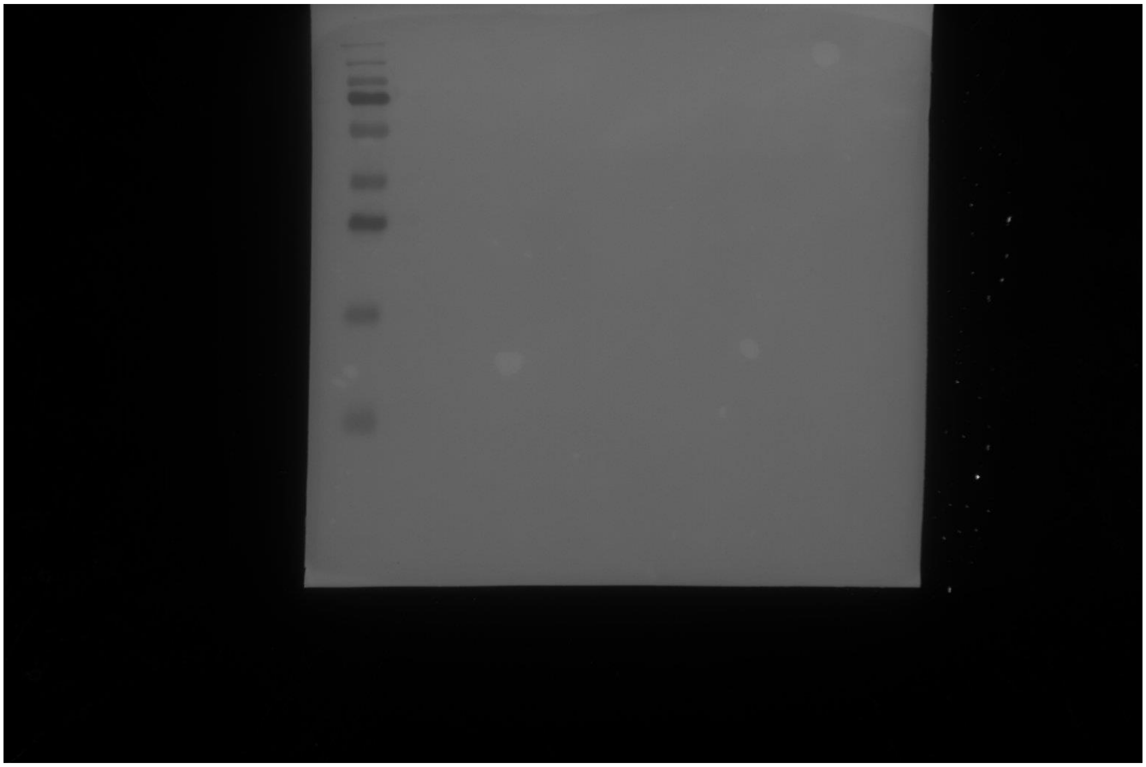

Figure 5E - IP – ONS76

GAPDH

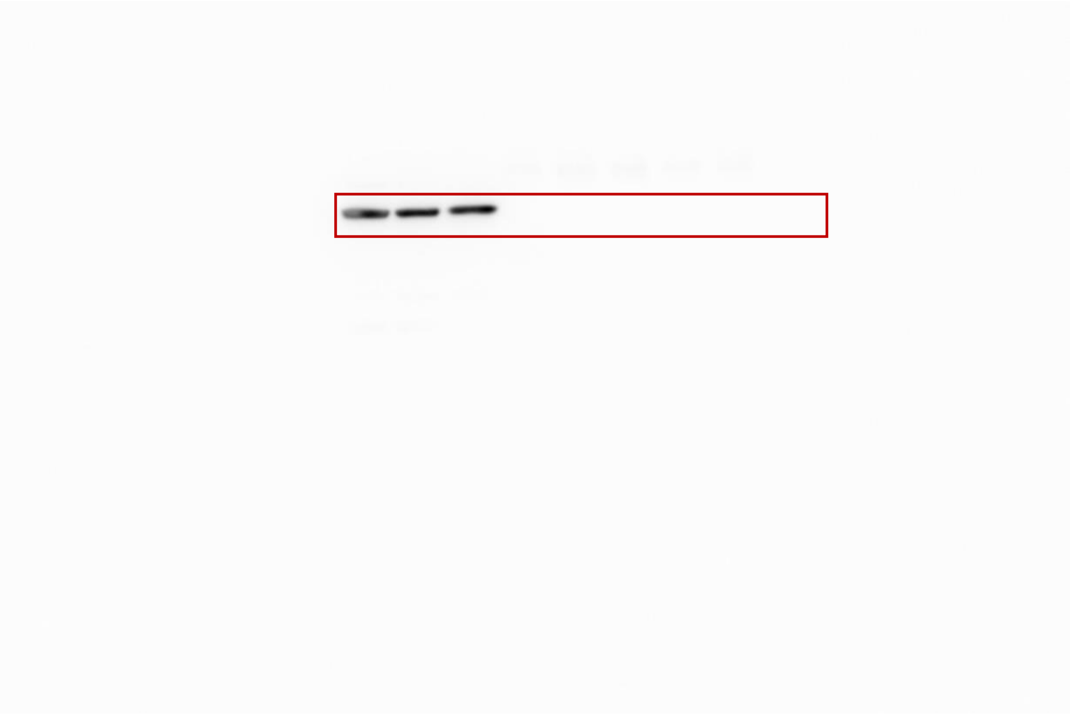

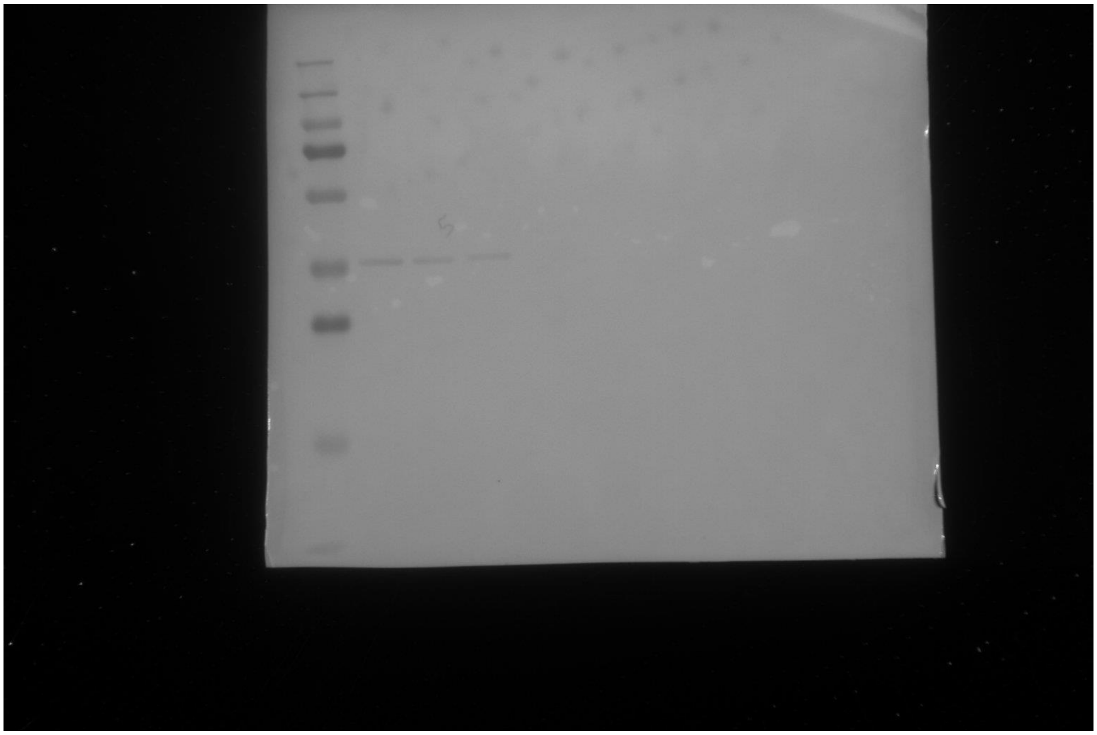

Figure 5F - IP – UW228

BCL-XL

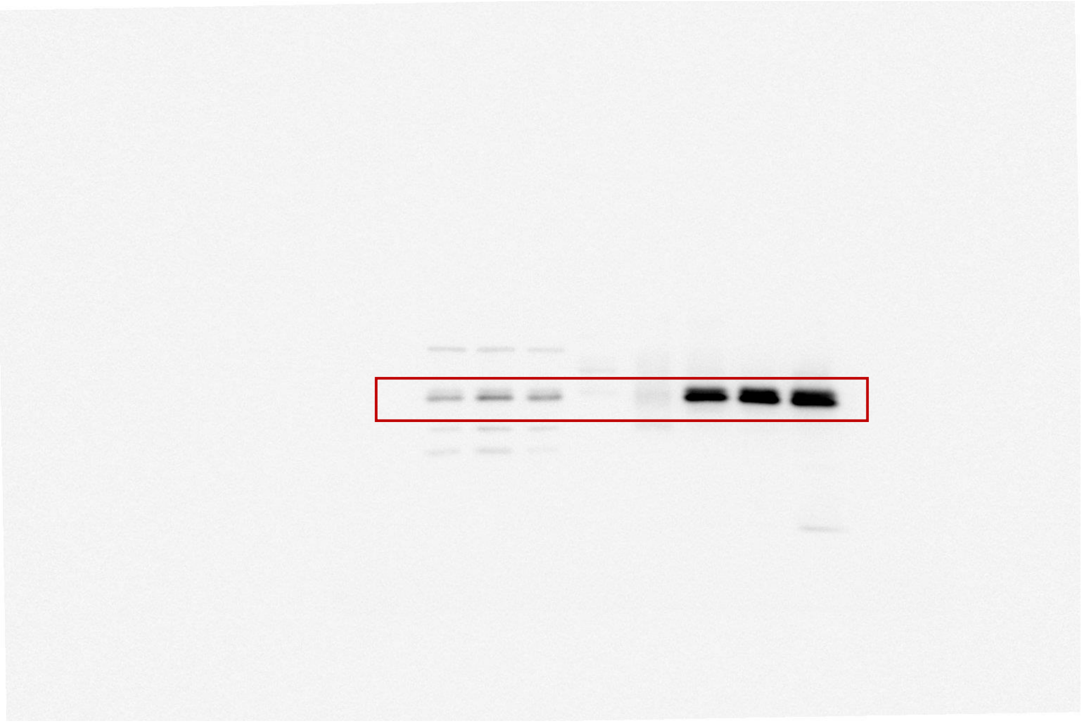

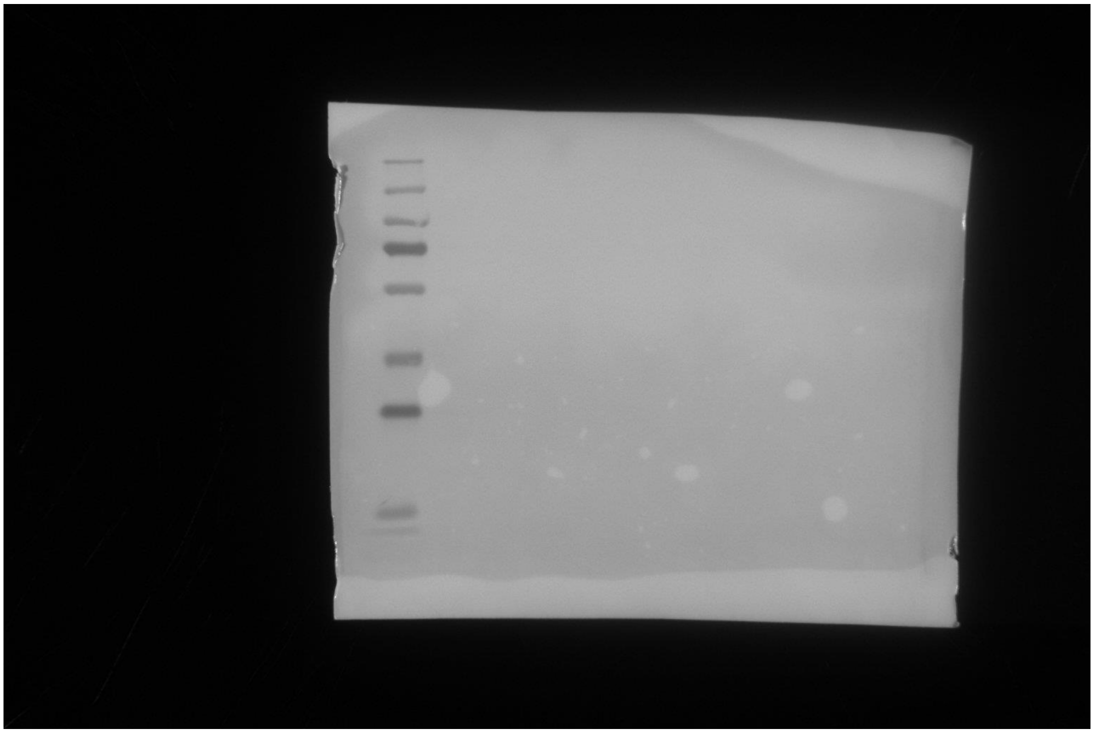

Figure 5F - IP – UW228

BAK

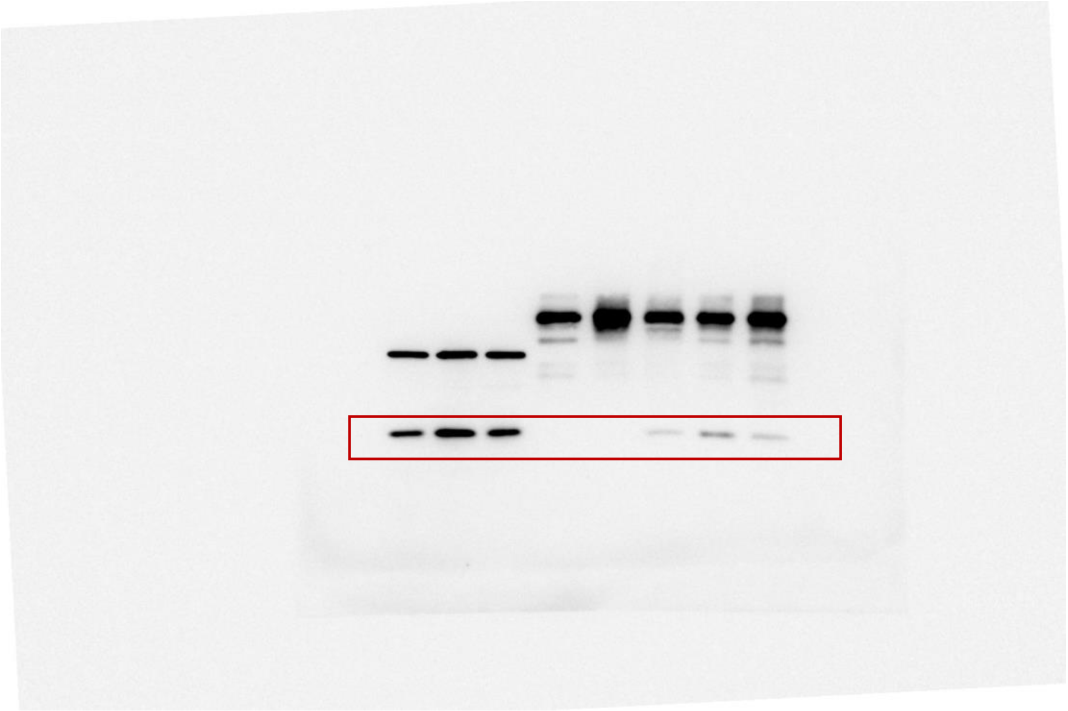

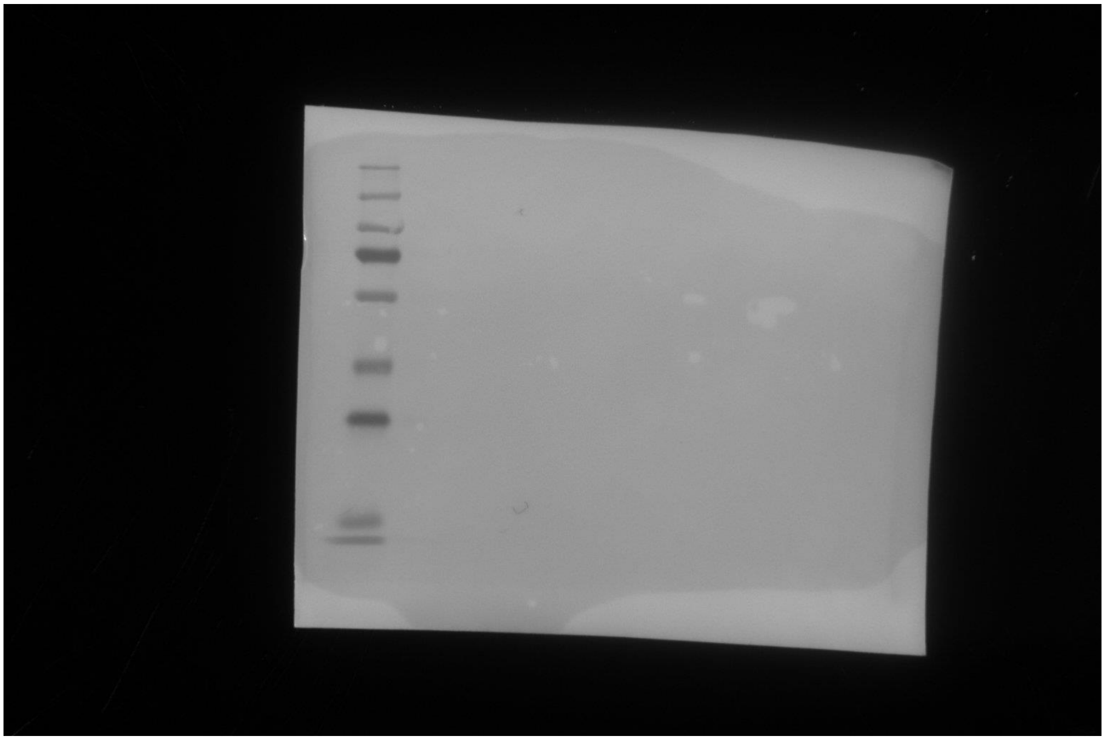

Figure 5F - IP – UW228

BAX

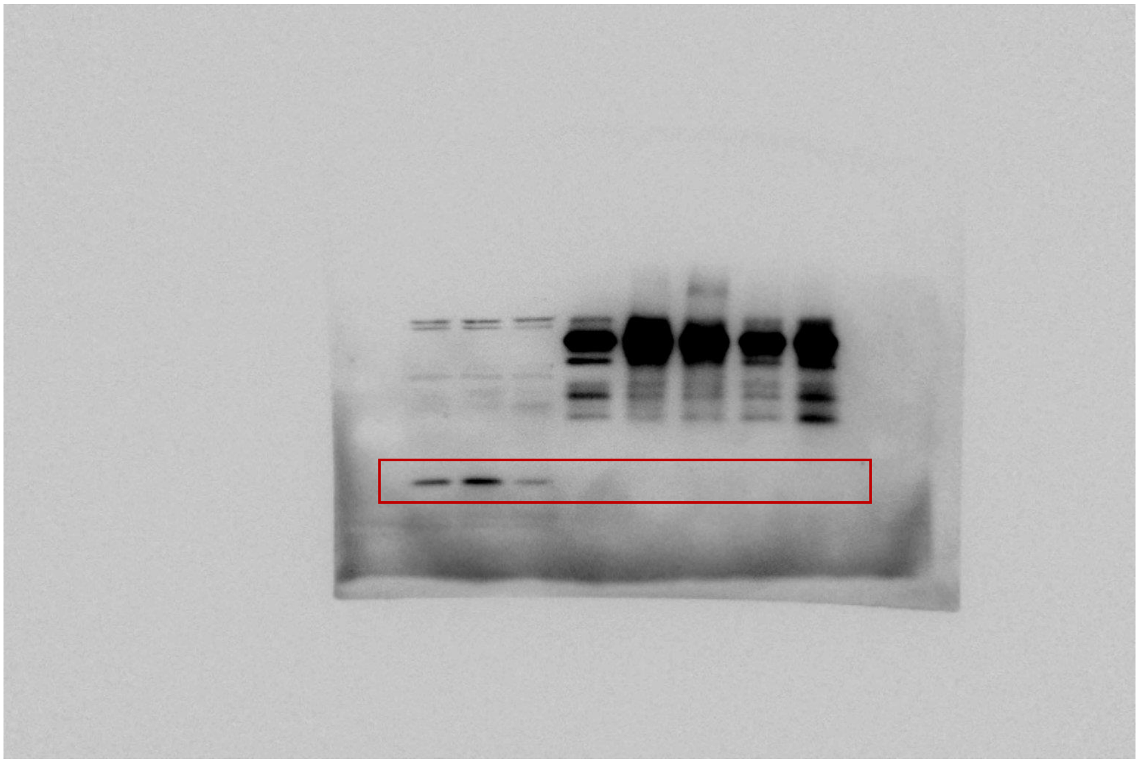

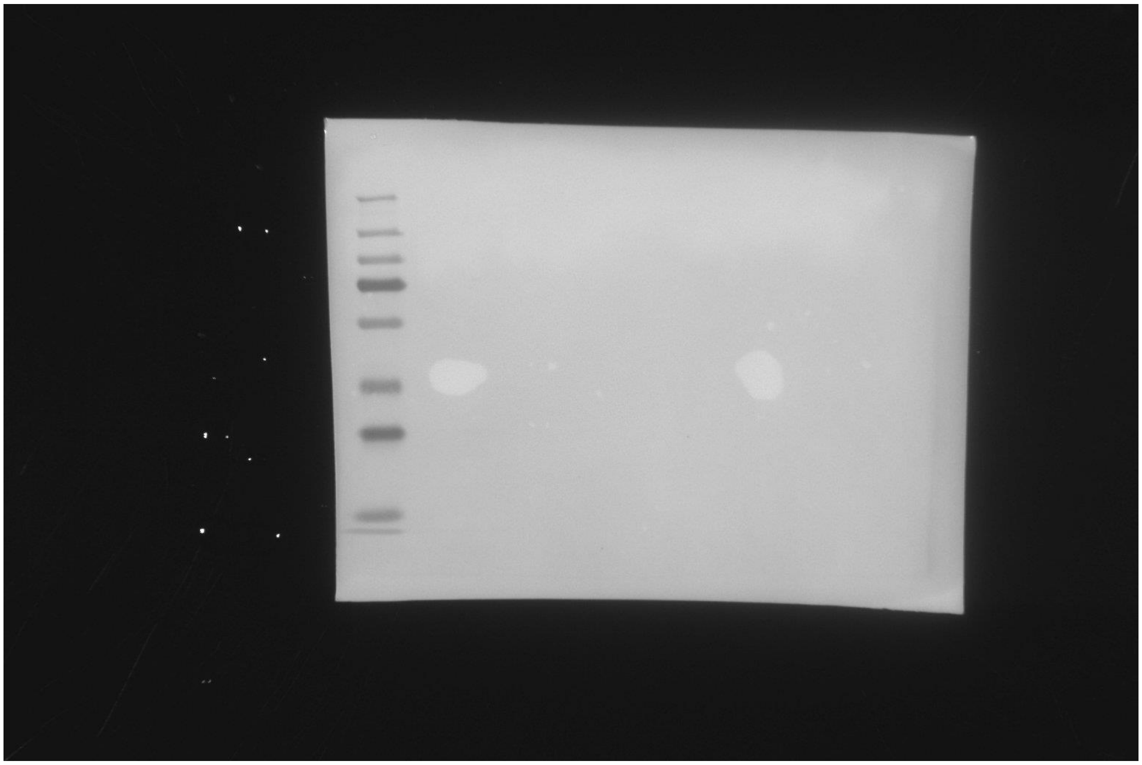

Figure 5F - IP – UW228

BIM

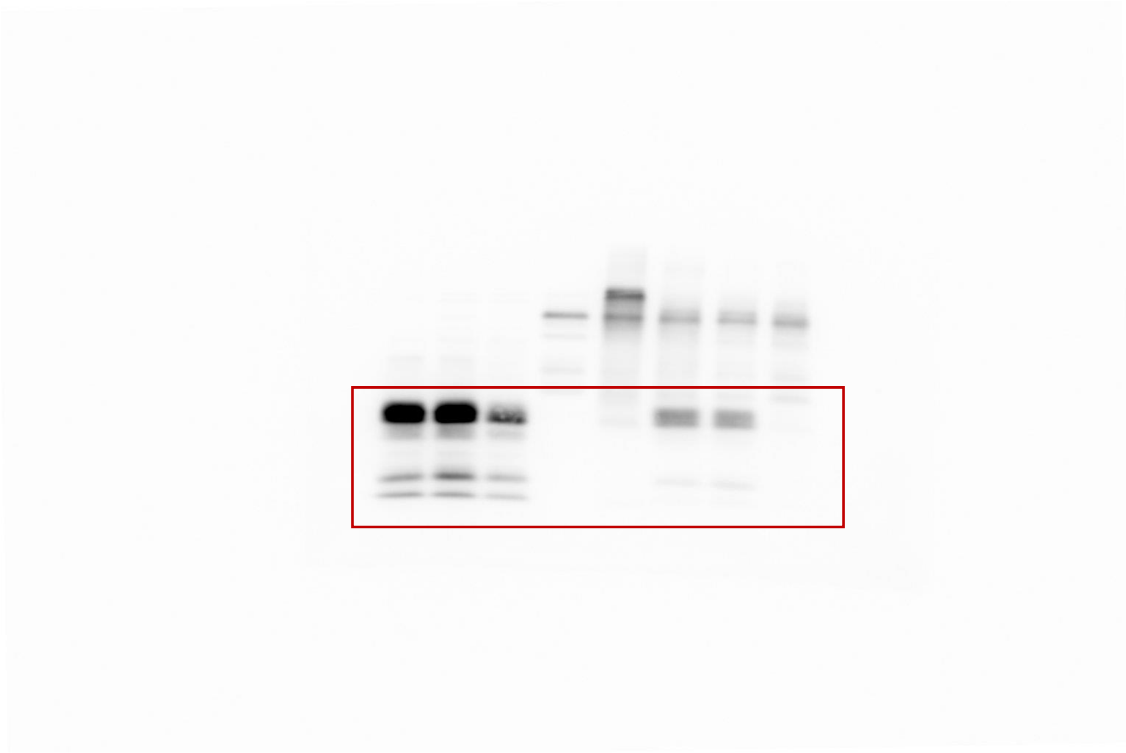

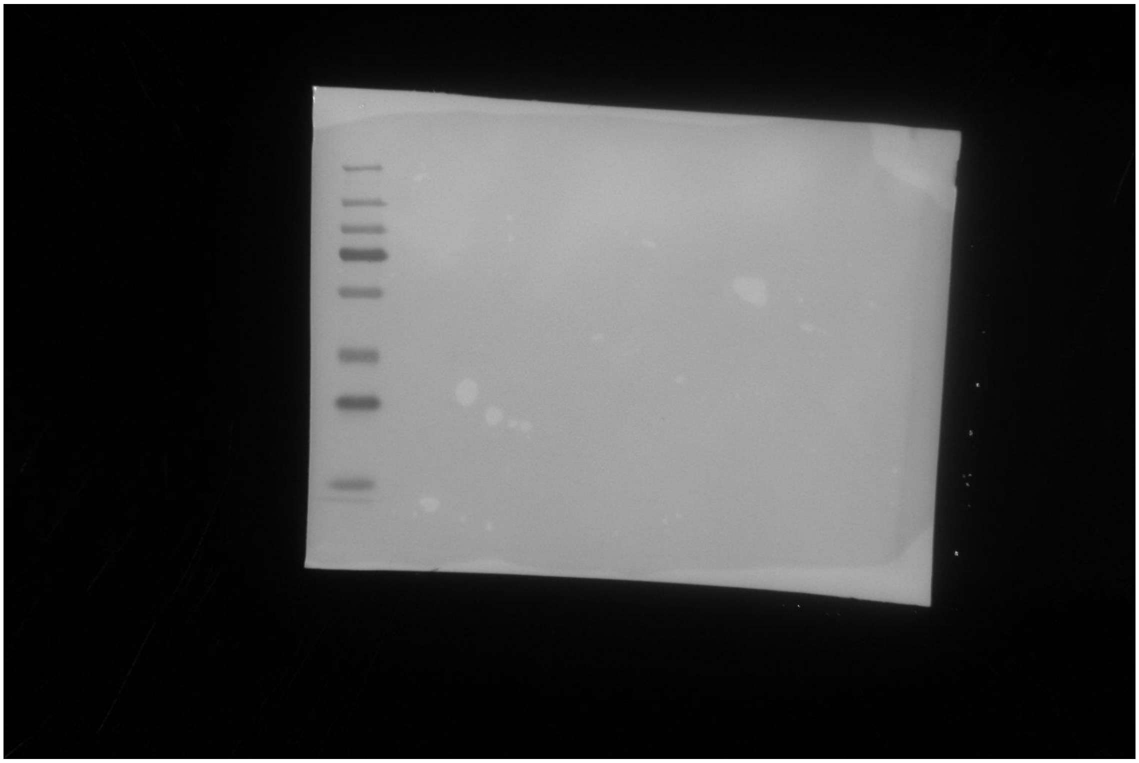

Figure 5F - IP – UW228

PUMA

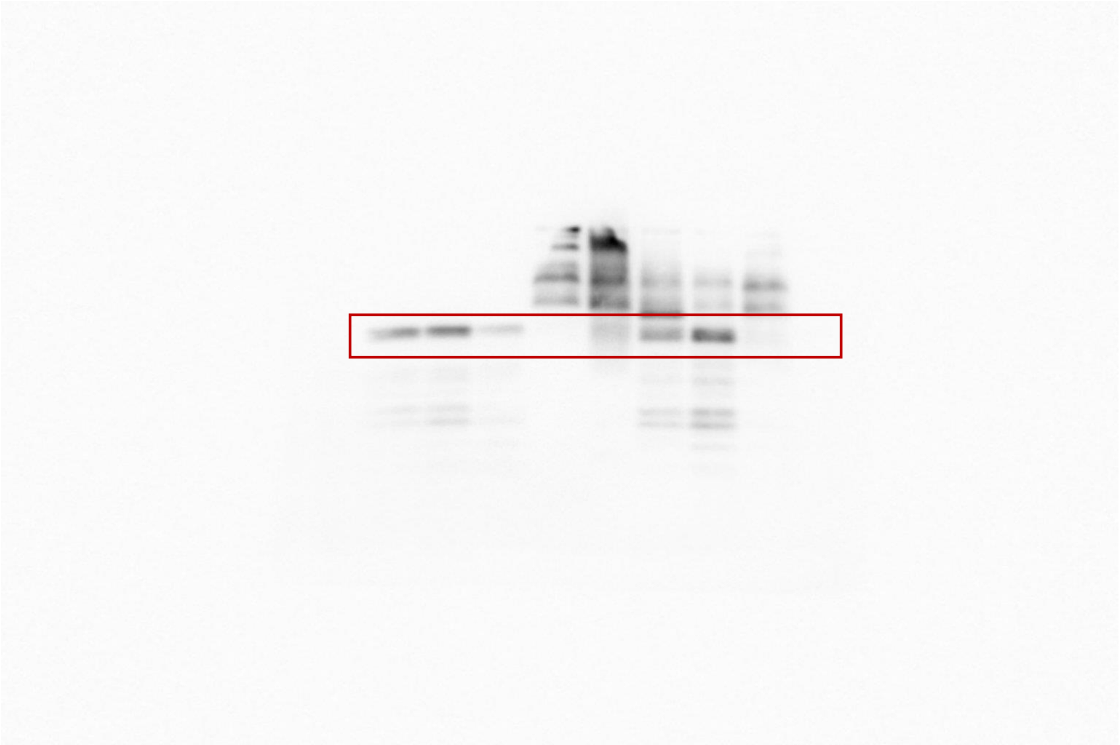

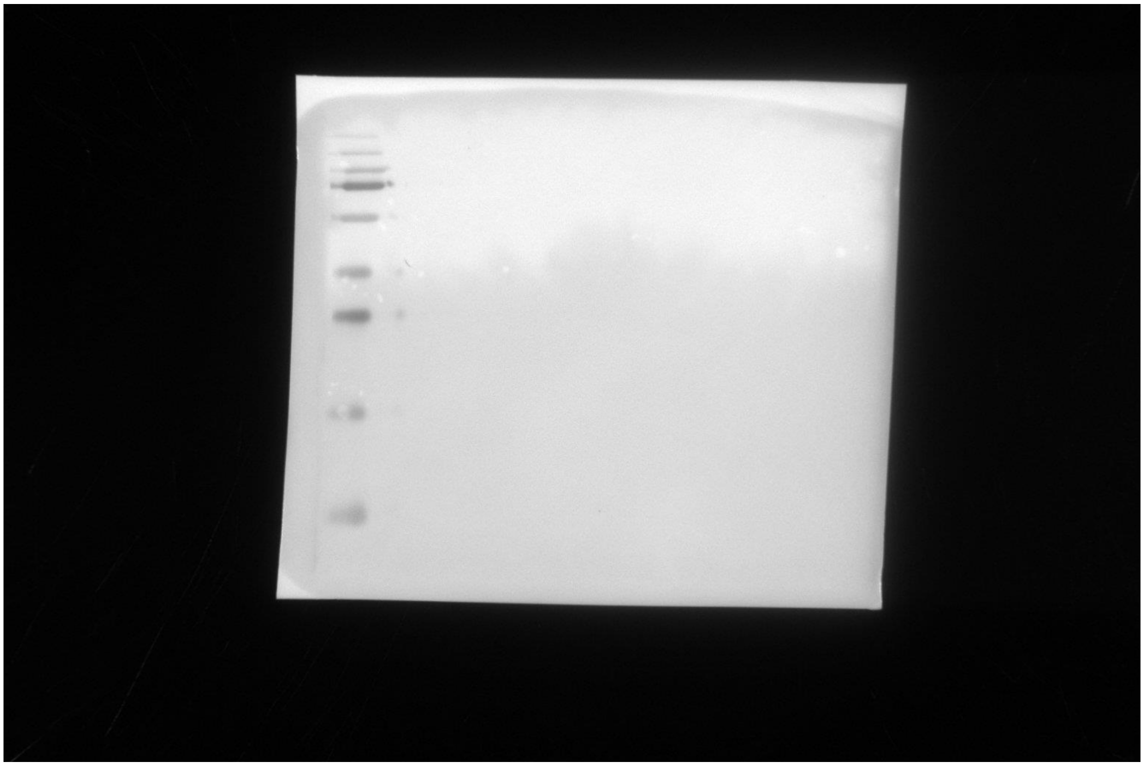

Figure 5F - IP – UW228

GAPDH

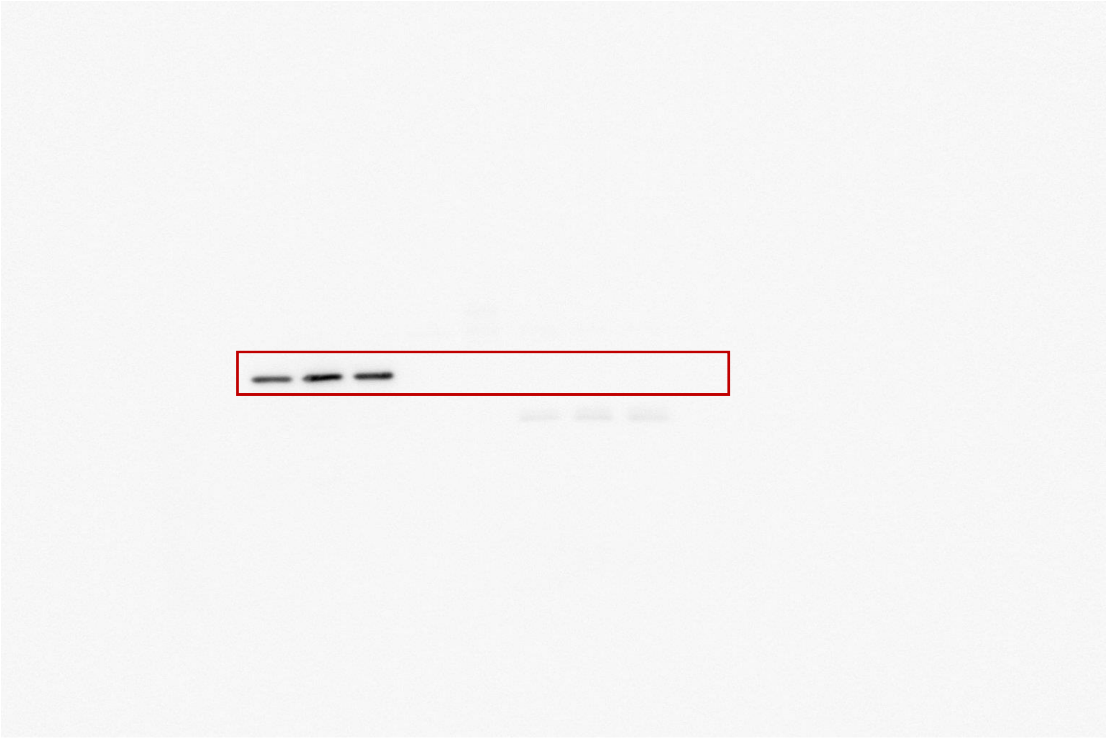

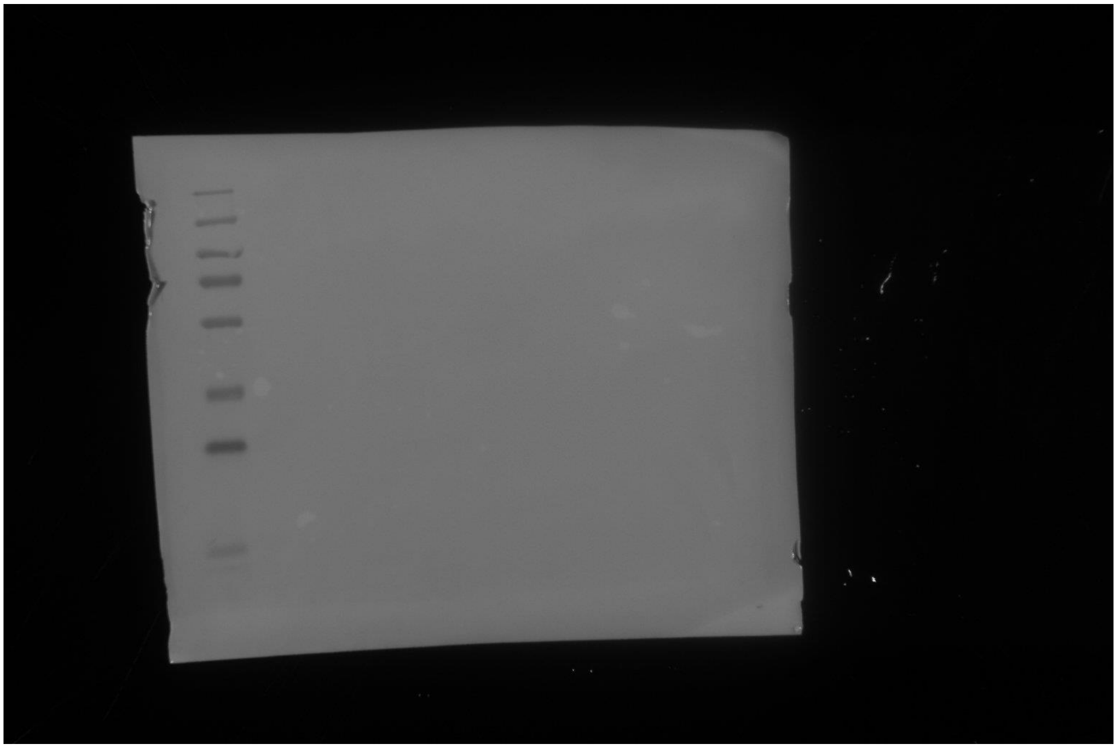

Supplementary figure 4A – ONS76

BAK

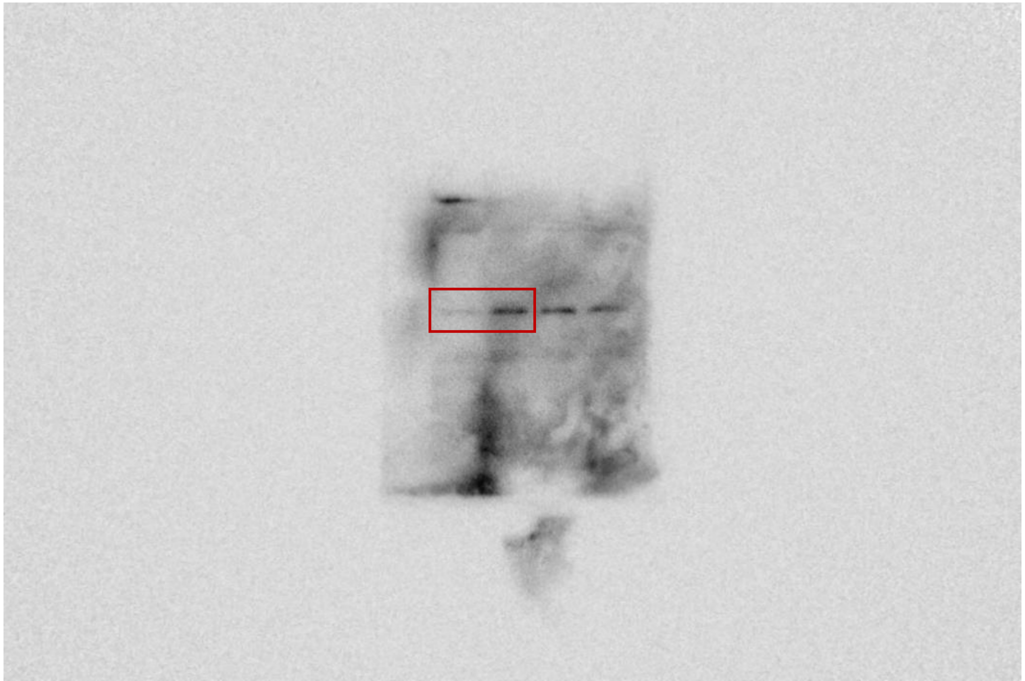

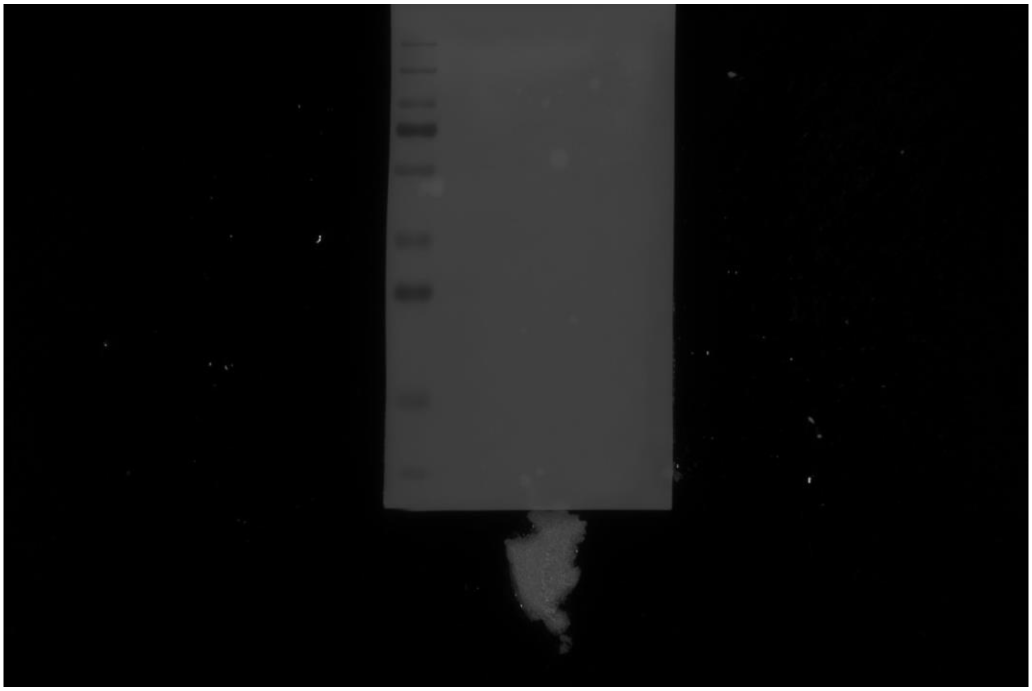

Supplementary figure 4A – ONS76

BAX

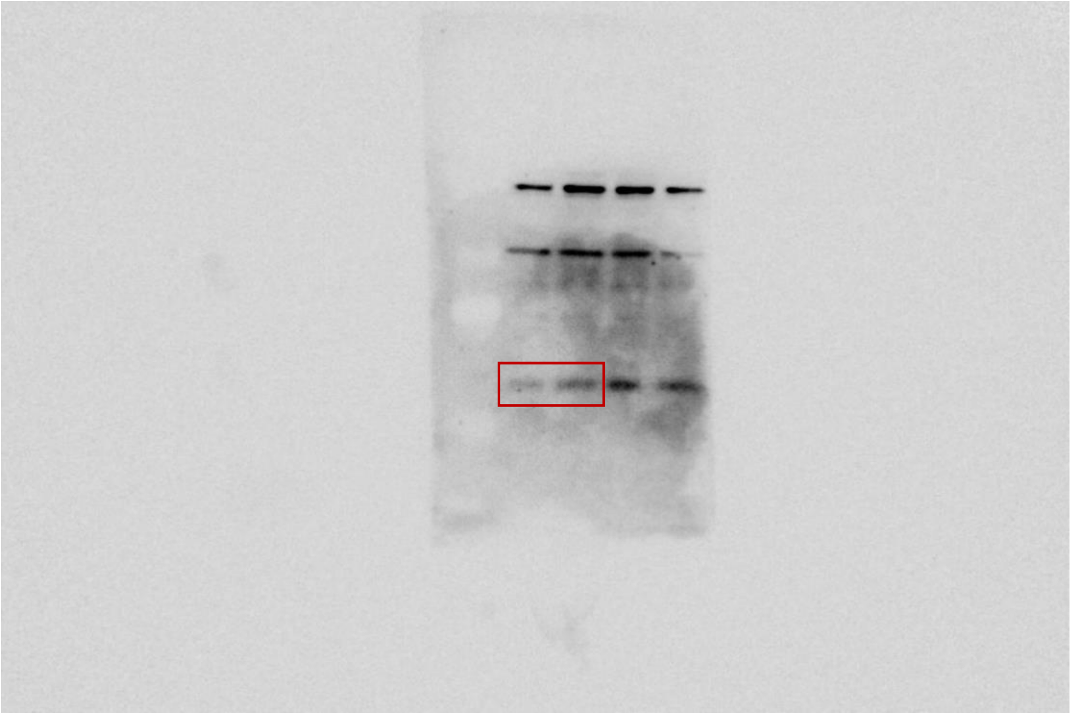

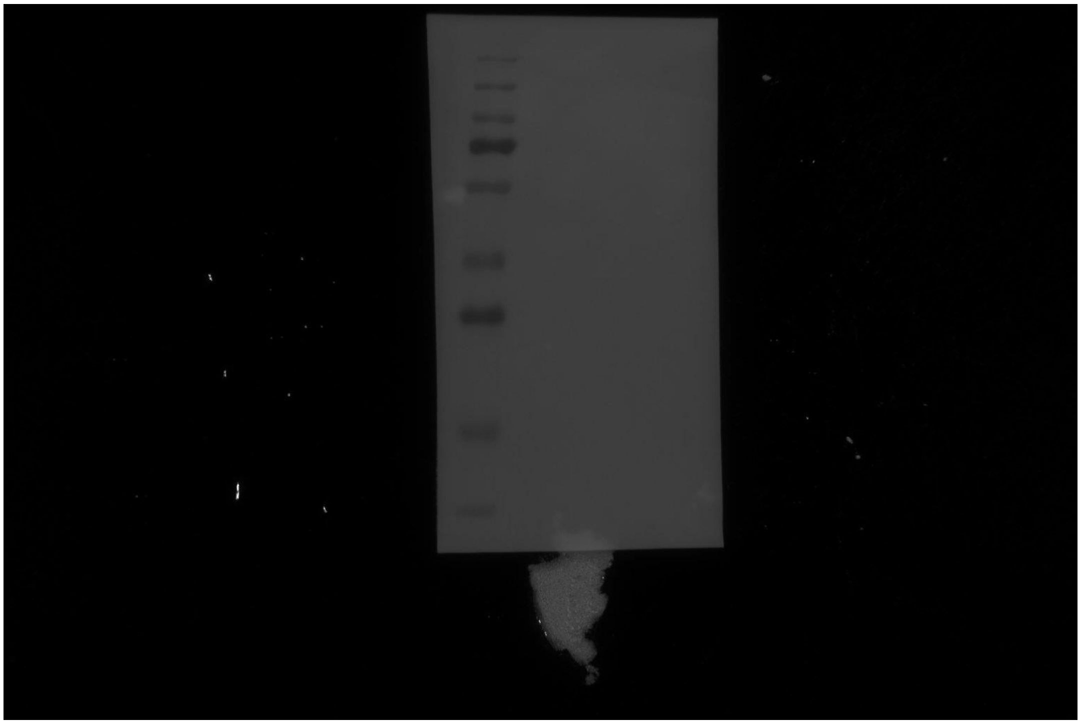

Supplementary figure 4A – ONS76

GAPDH

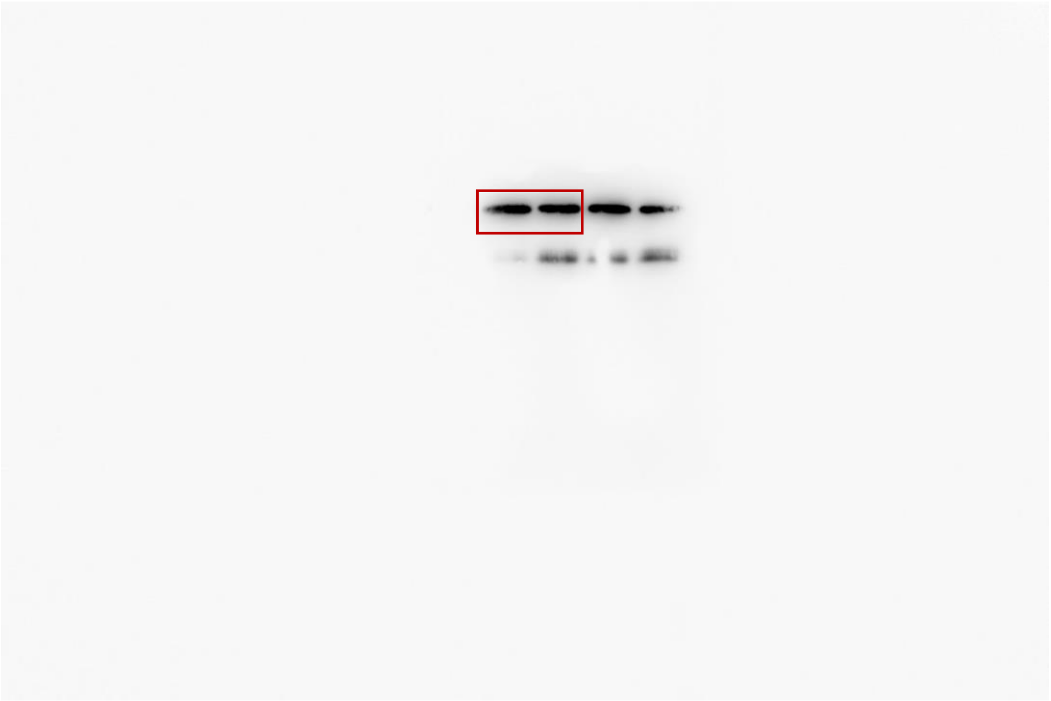

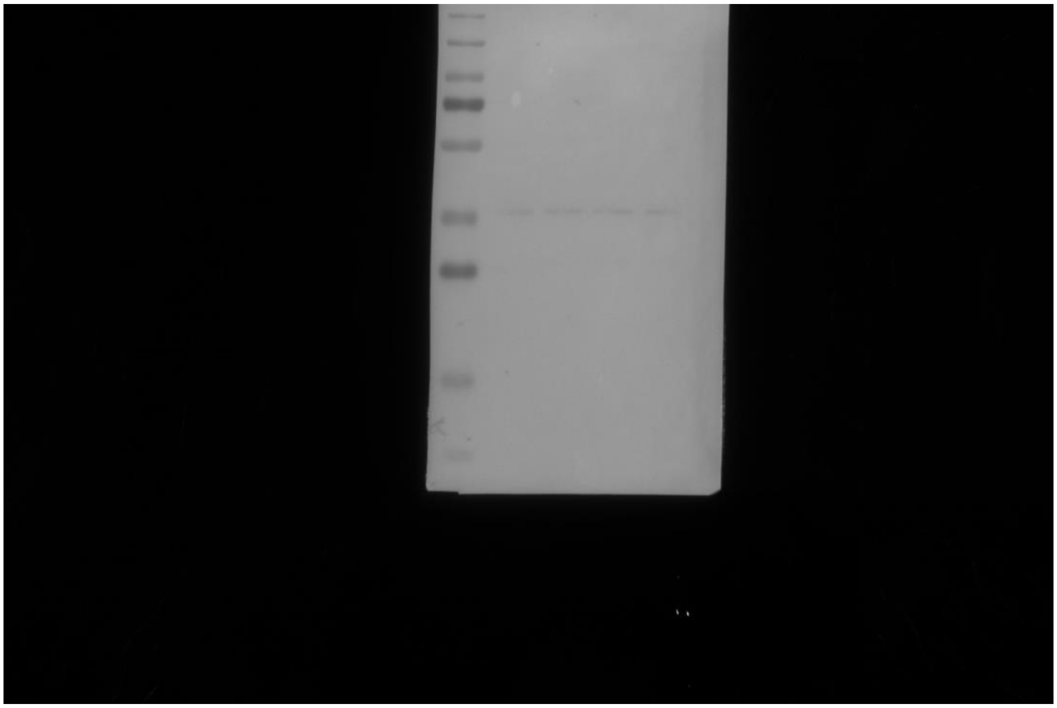

Supplementary figure 4A – ONS76

BIM

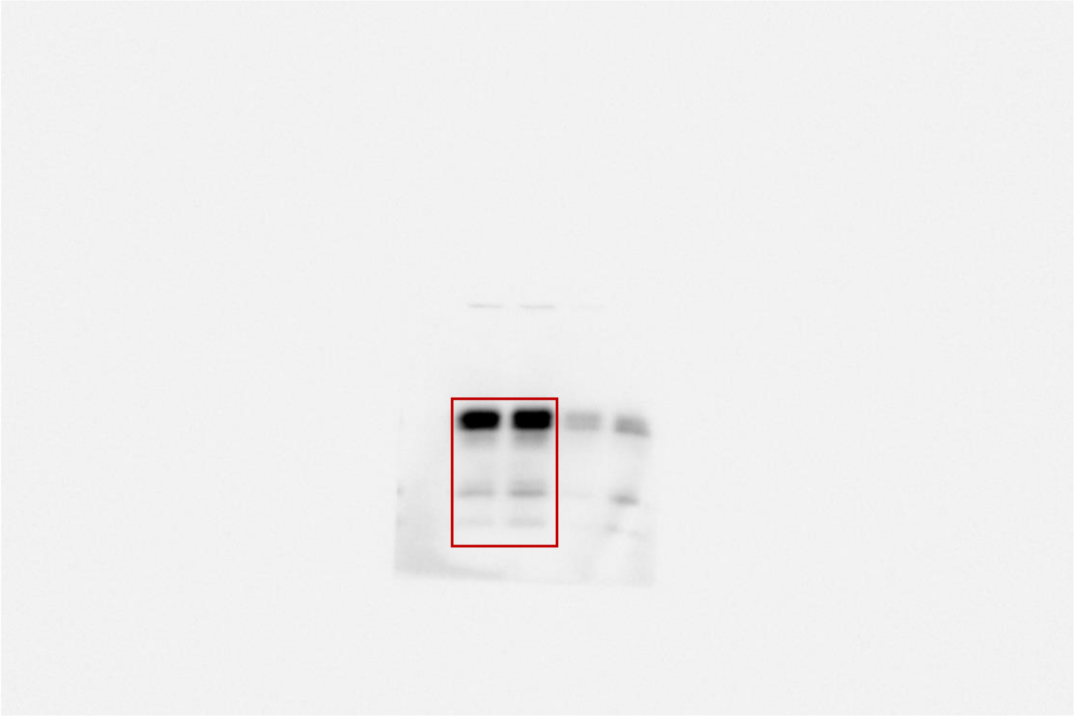

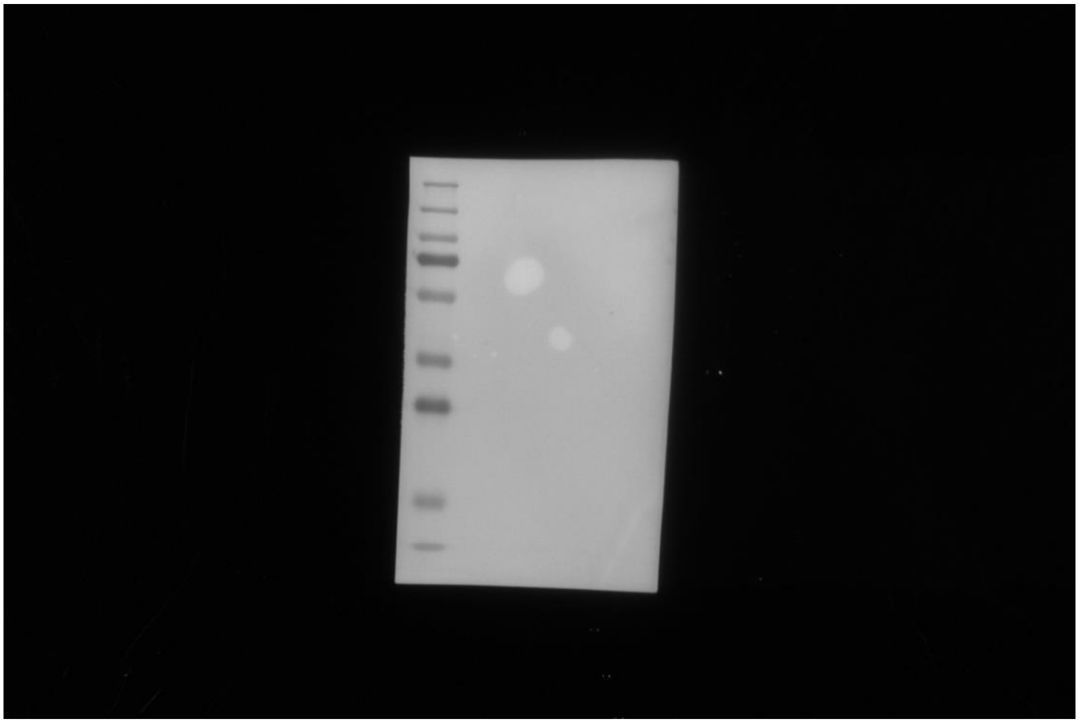

Supplementary figure 4A – ONS76

GAPDH

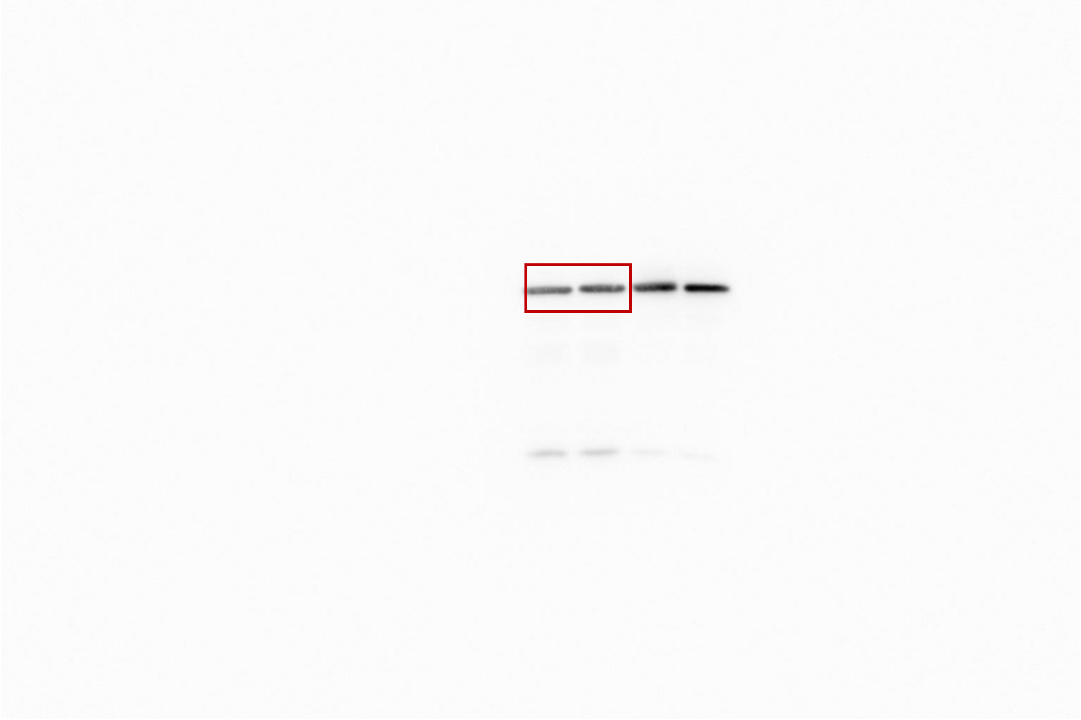

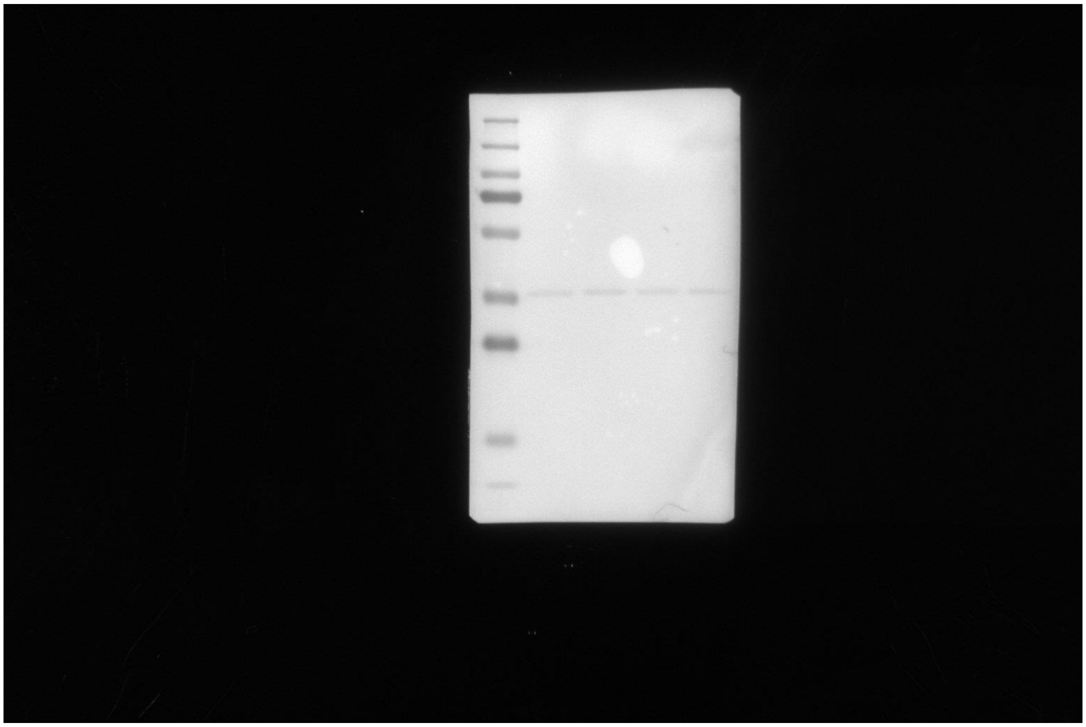

Supplementary figure 4A – ONS76

BID

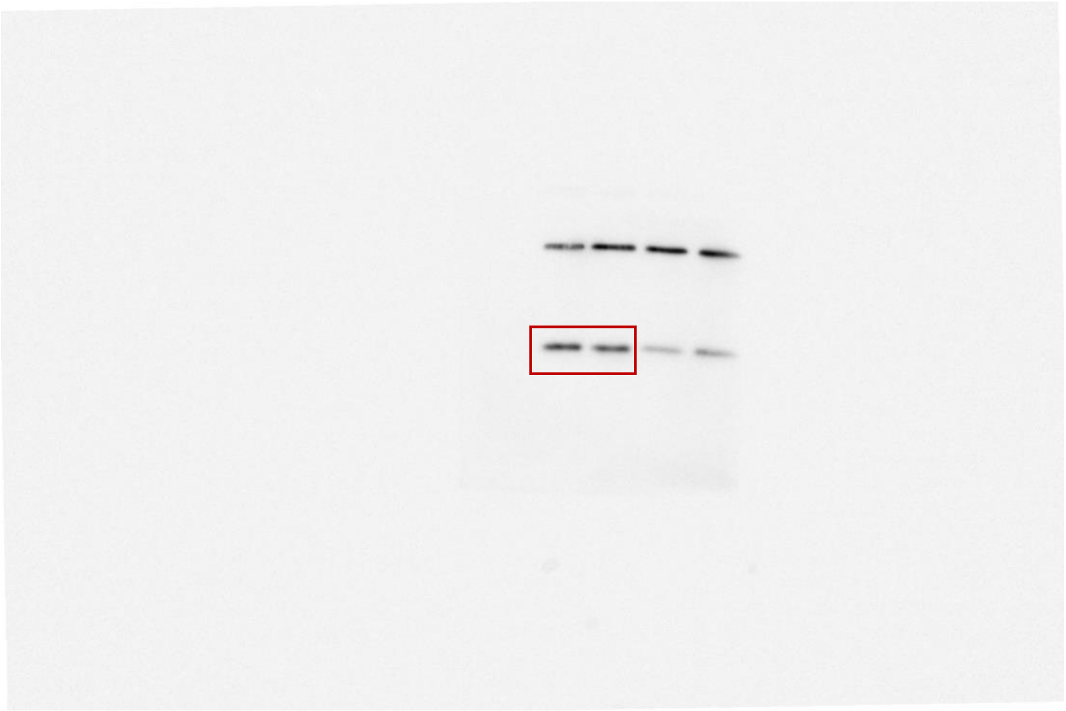

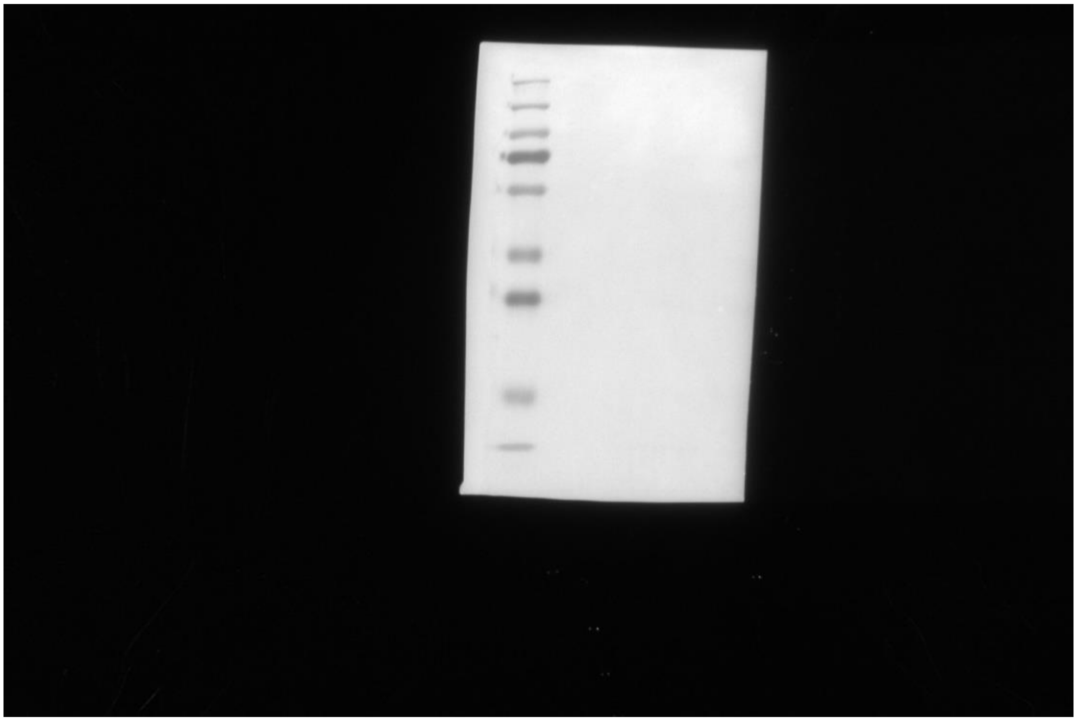

Supplementary figure 4A – ONS76

PUMA

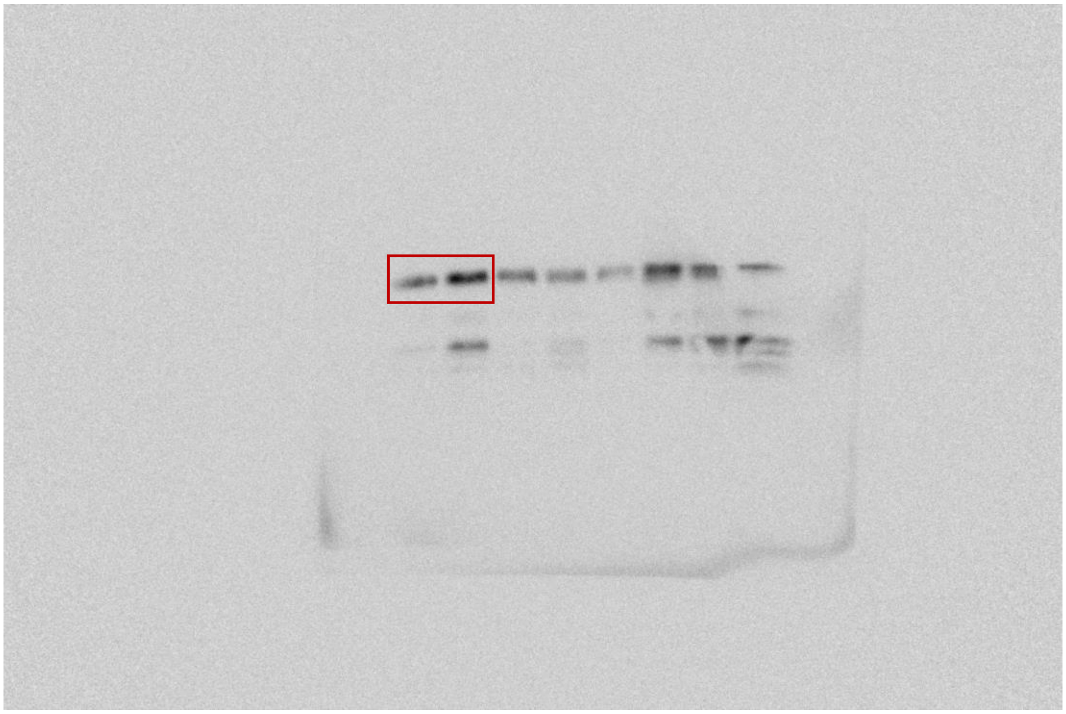

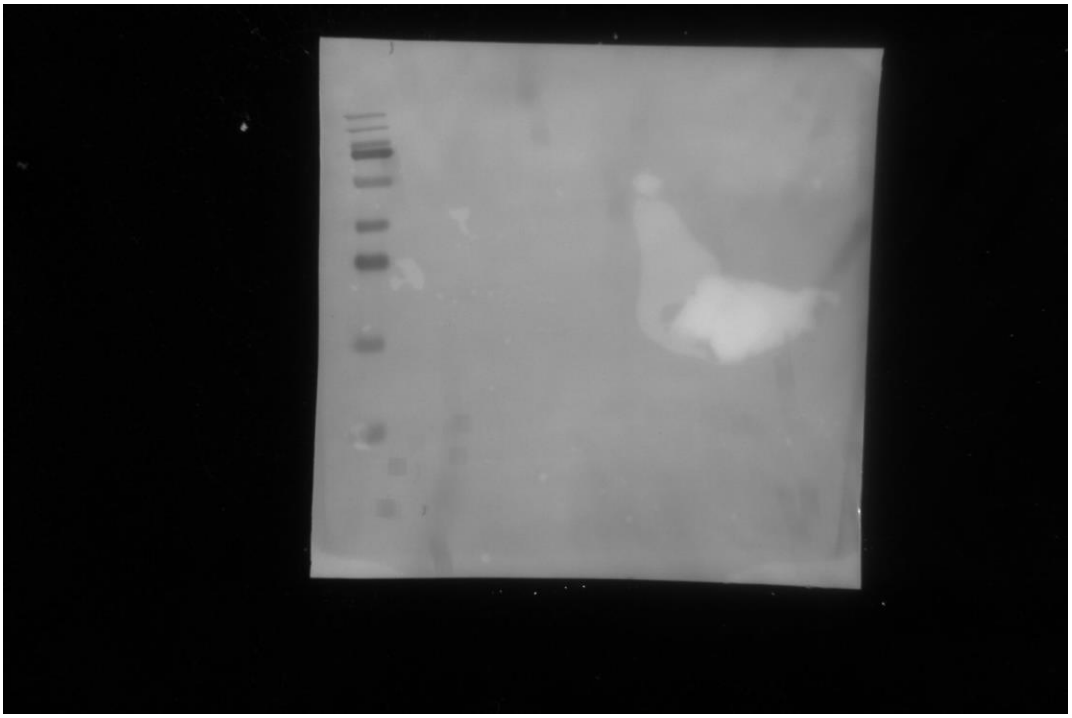

Supplementary figure 4A – ONS76

NOXA

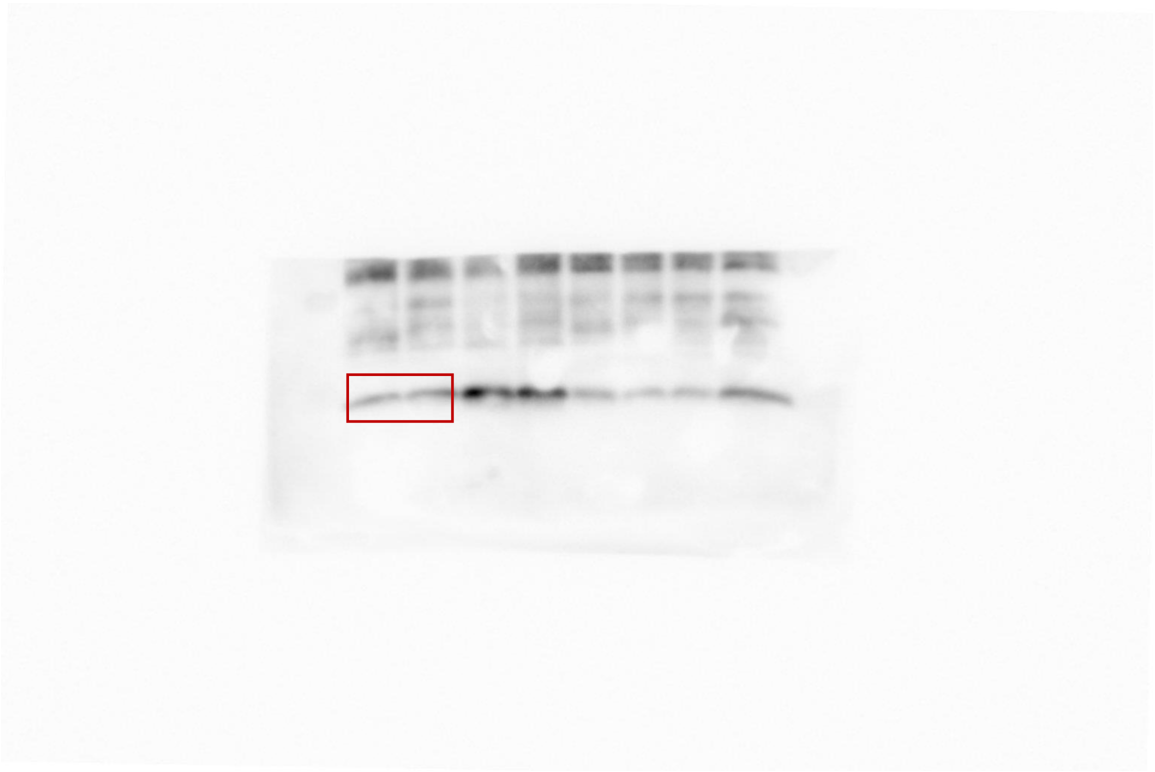

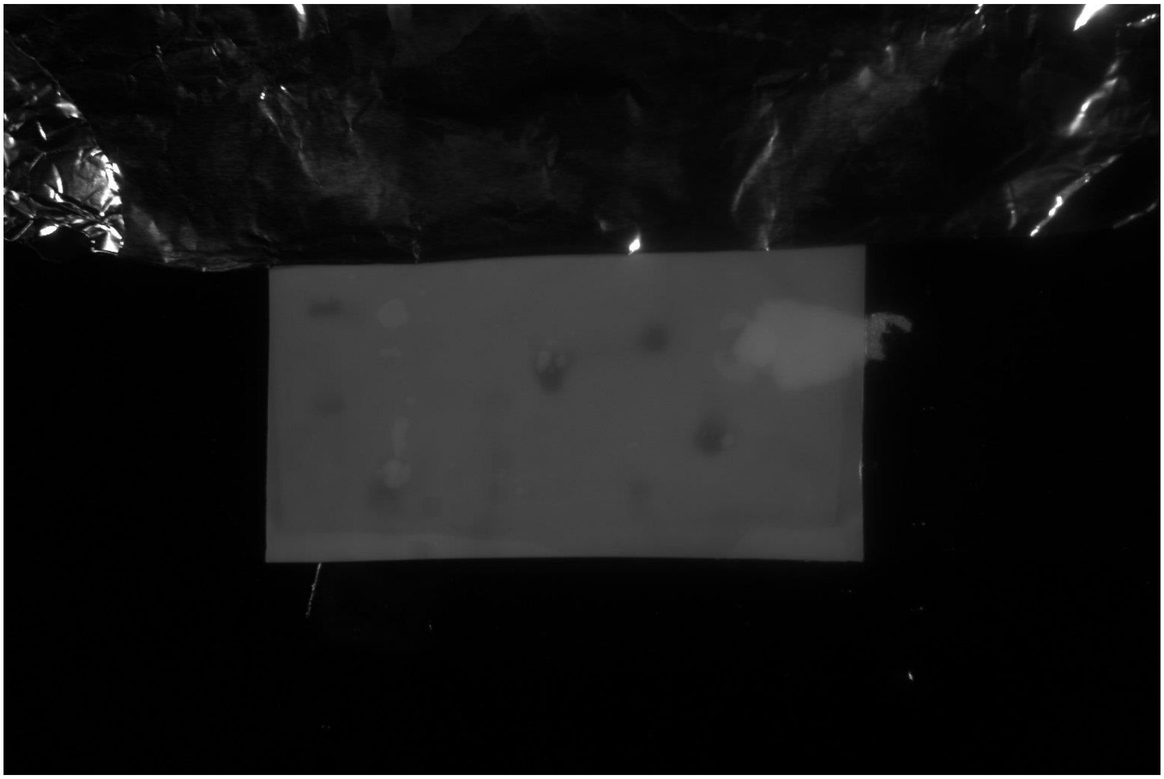

Supplementary figure 4A – ONS76

GAPDH

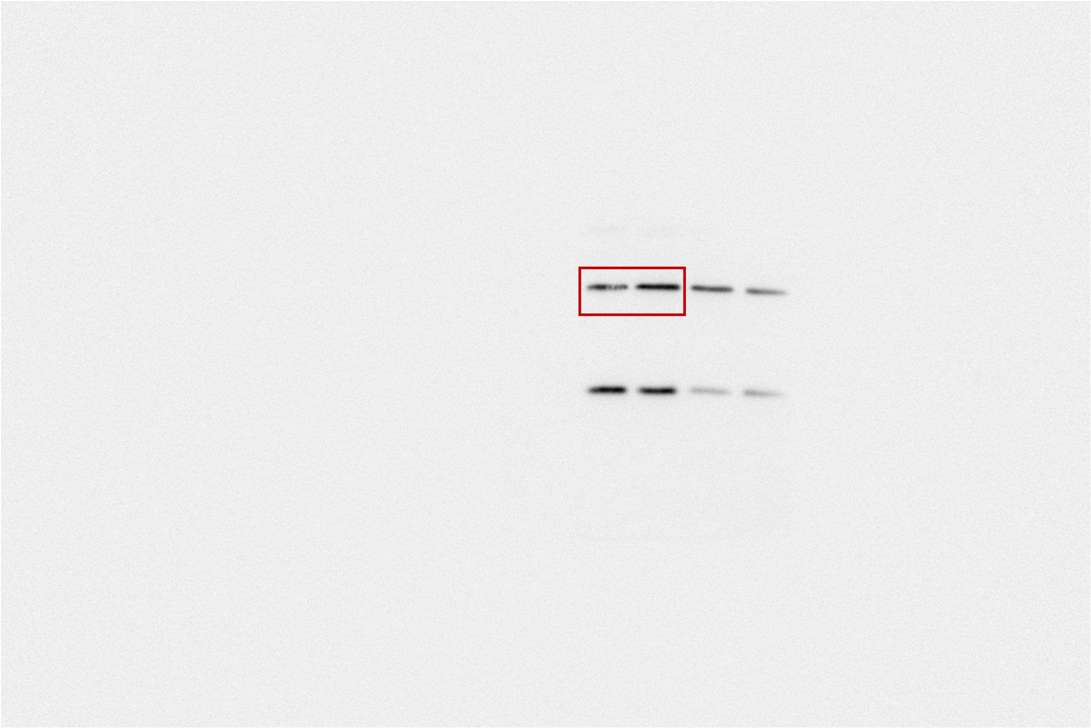

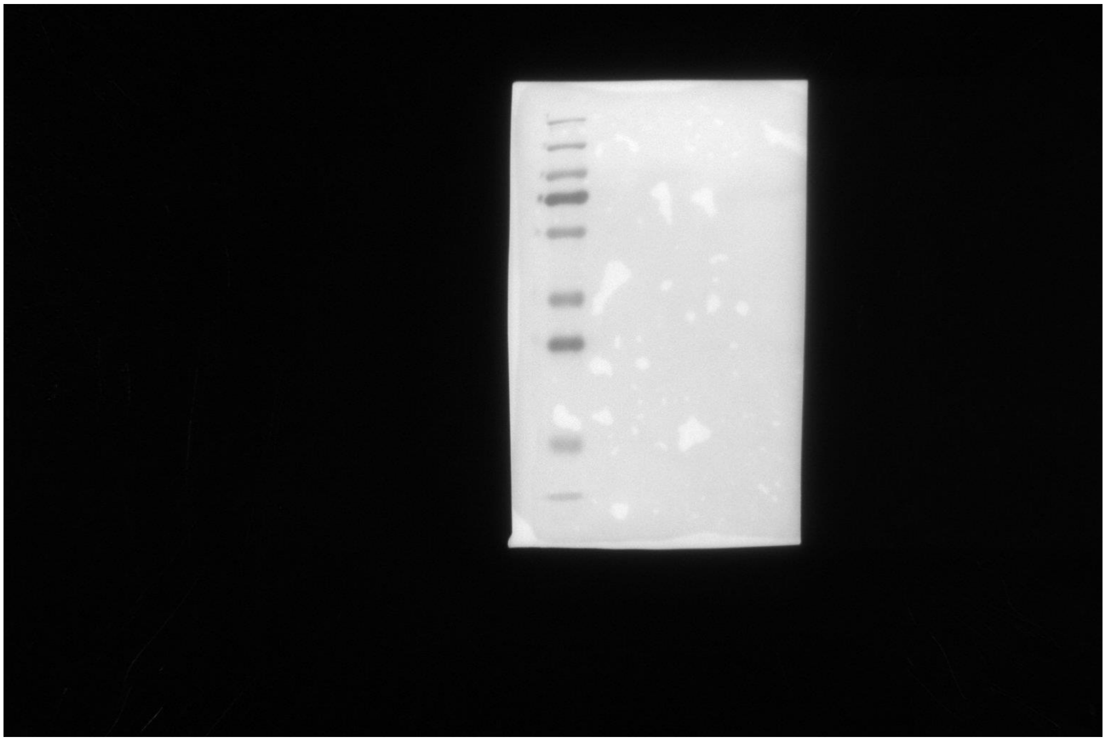

Supplementary figure 4B – UW228

BAK

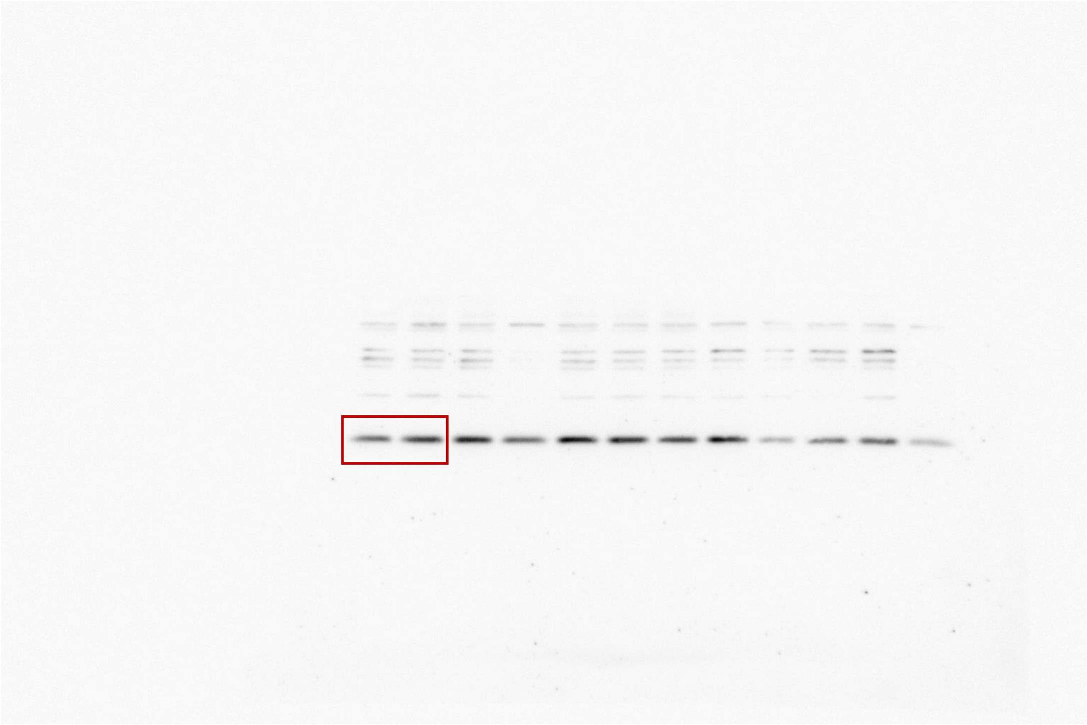

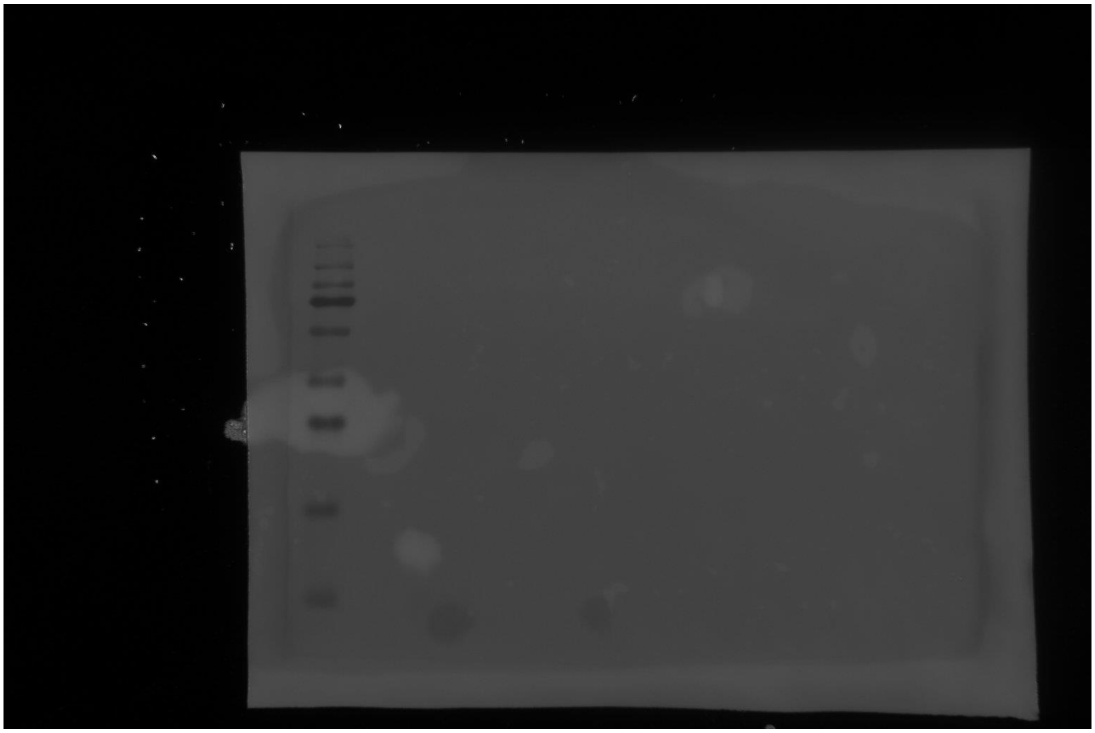

Supplementary figure 4B – UW228

BAX

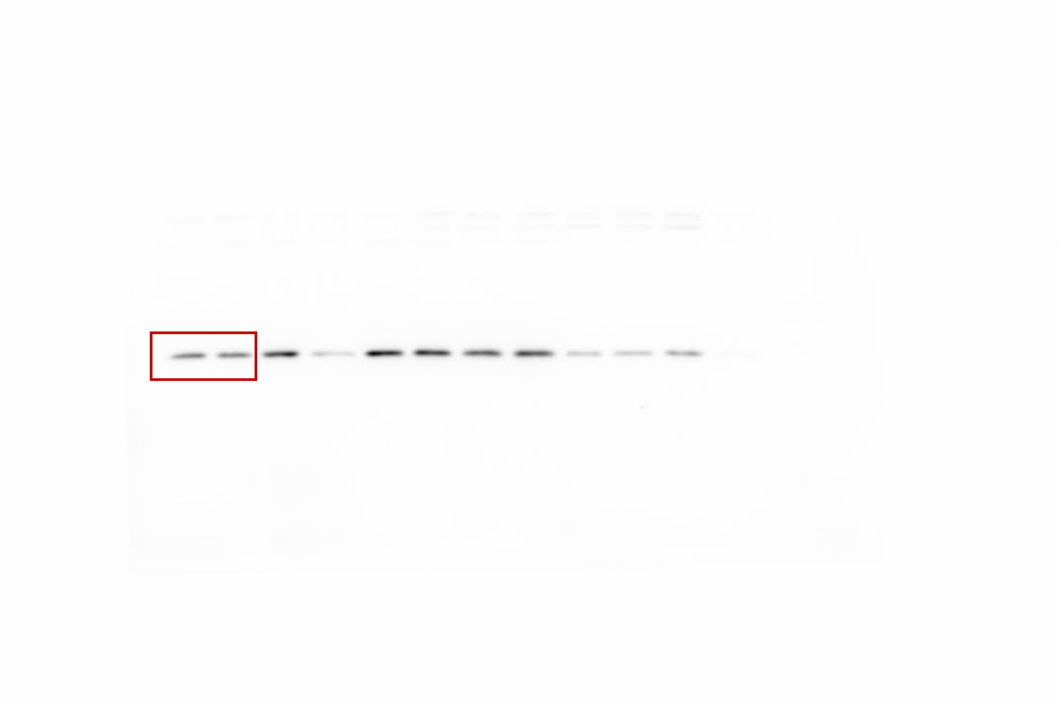

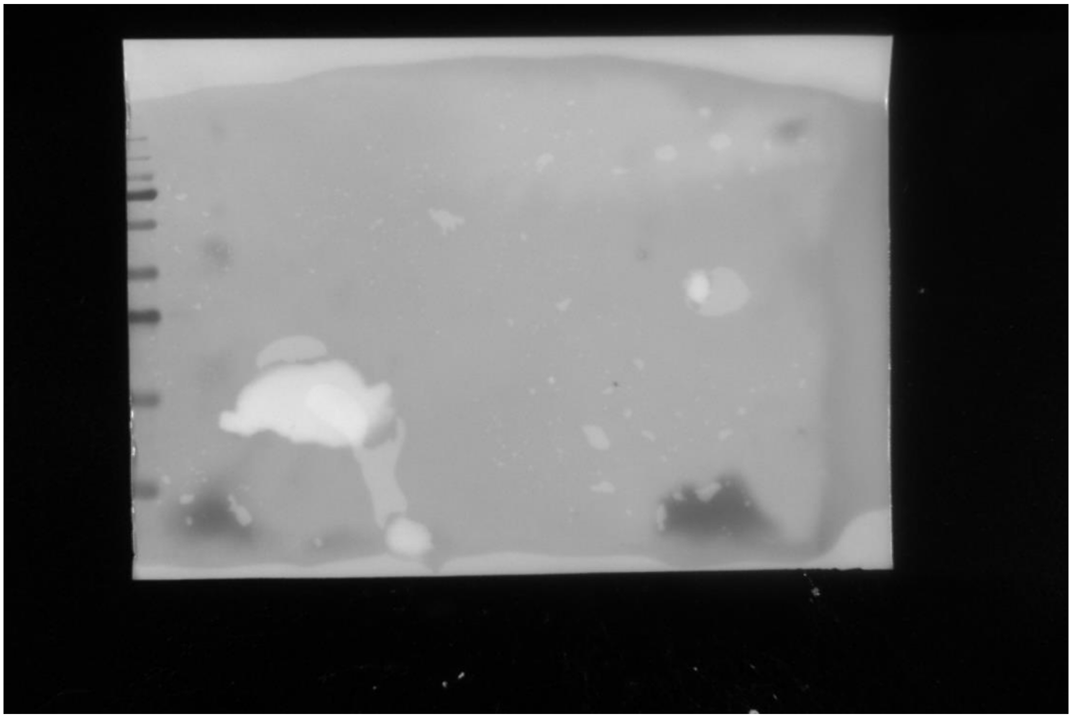

Supplementary figure 4B – UW228

GAPDH

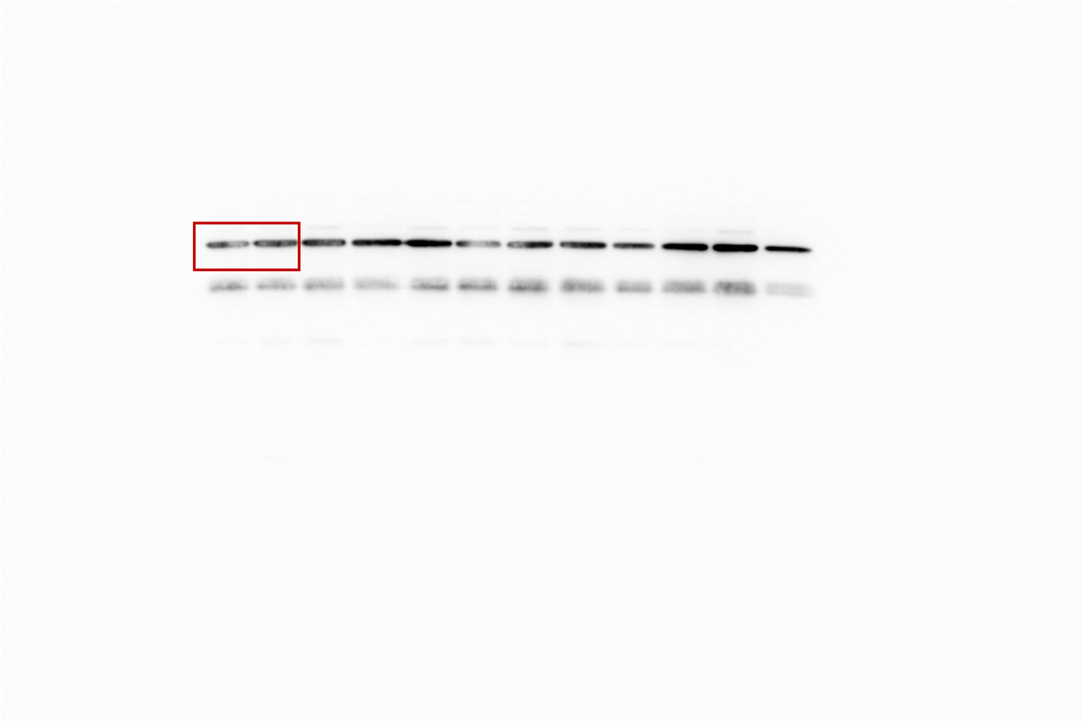

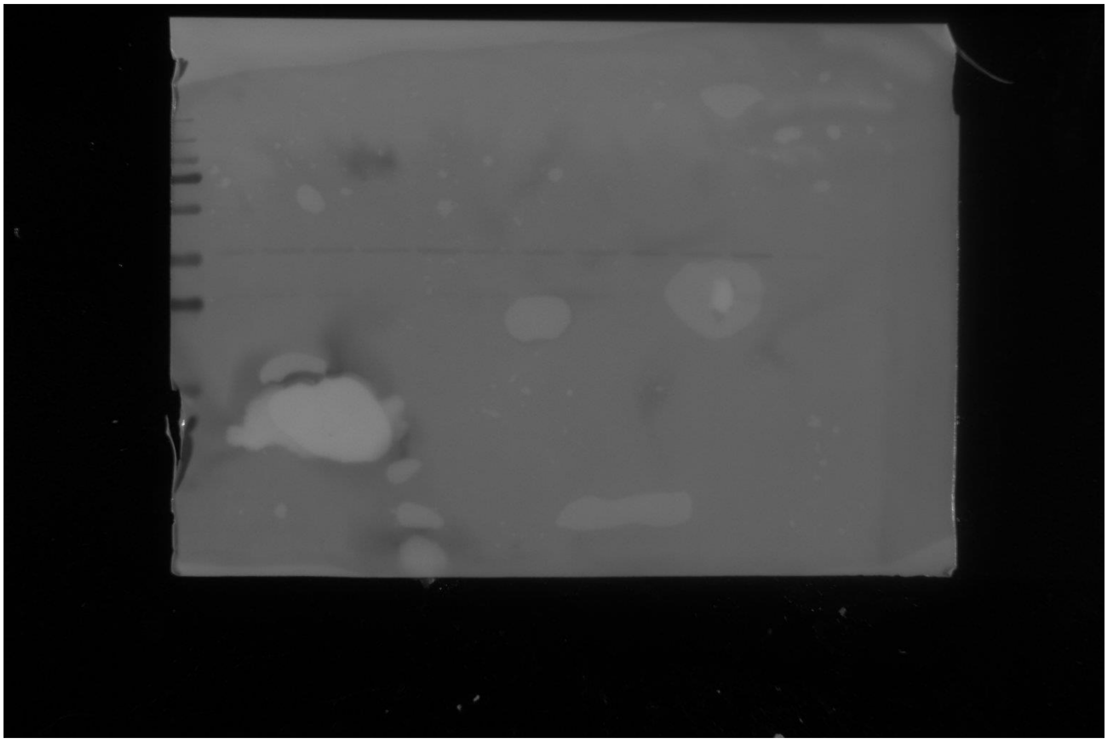

Supplementary figure 4B – UW228

BIM

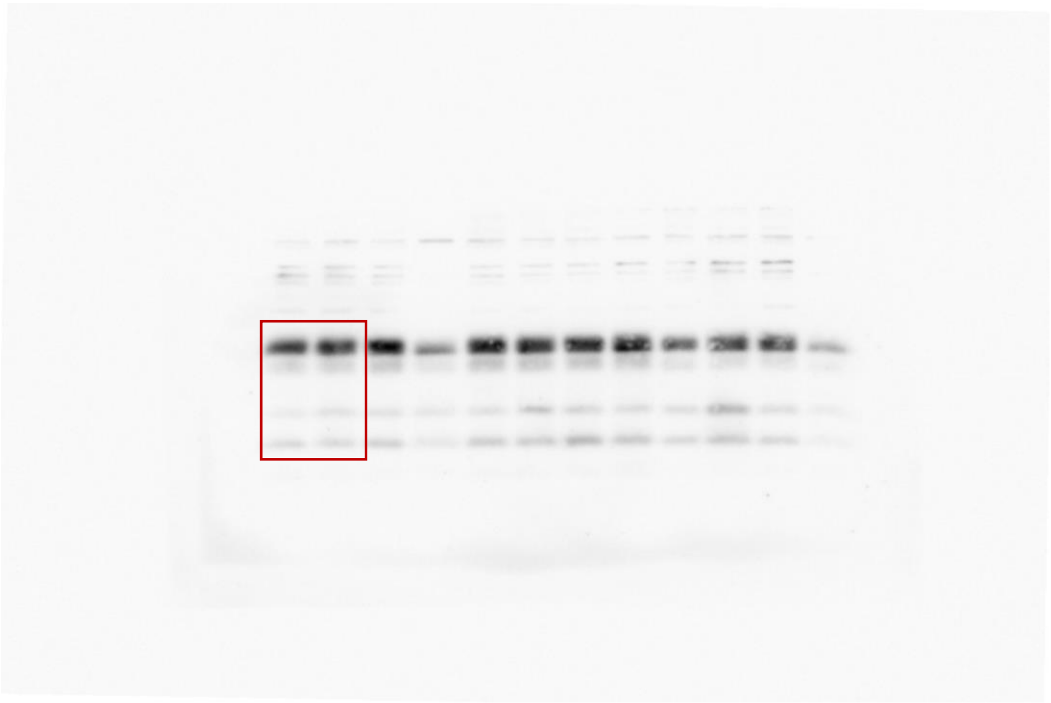

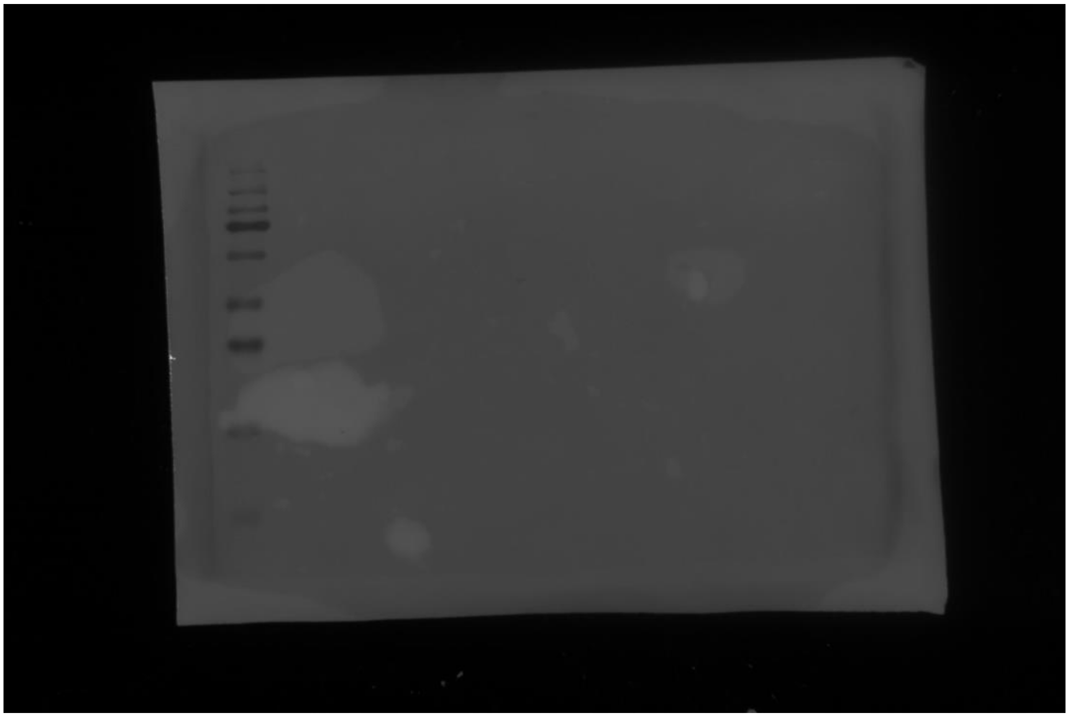

Supplementary figure 4B – UW228

GAPDH

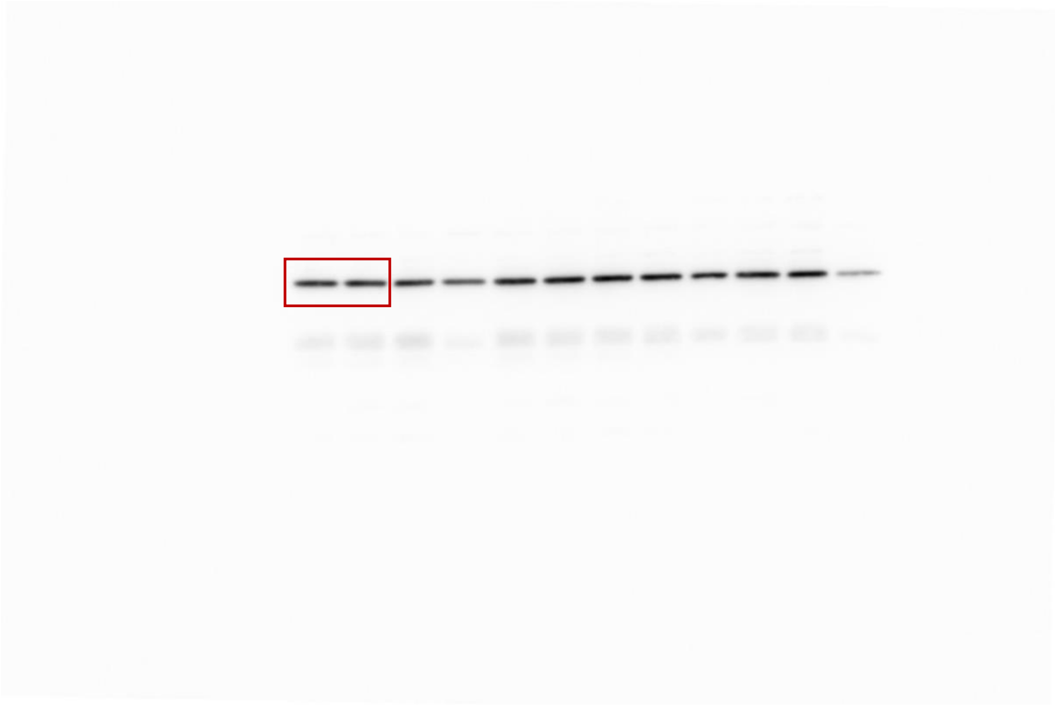

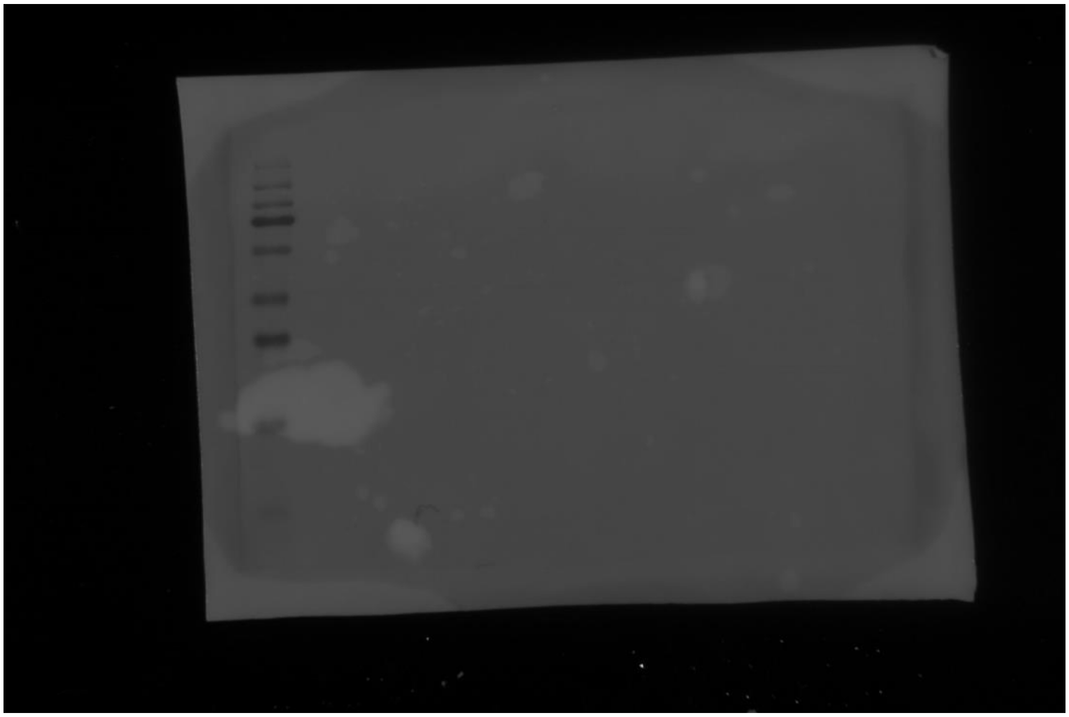

Supplementary figure 4B – UW228

BID

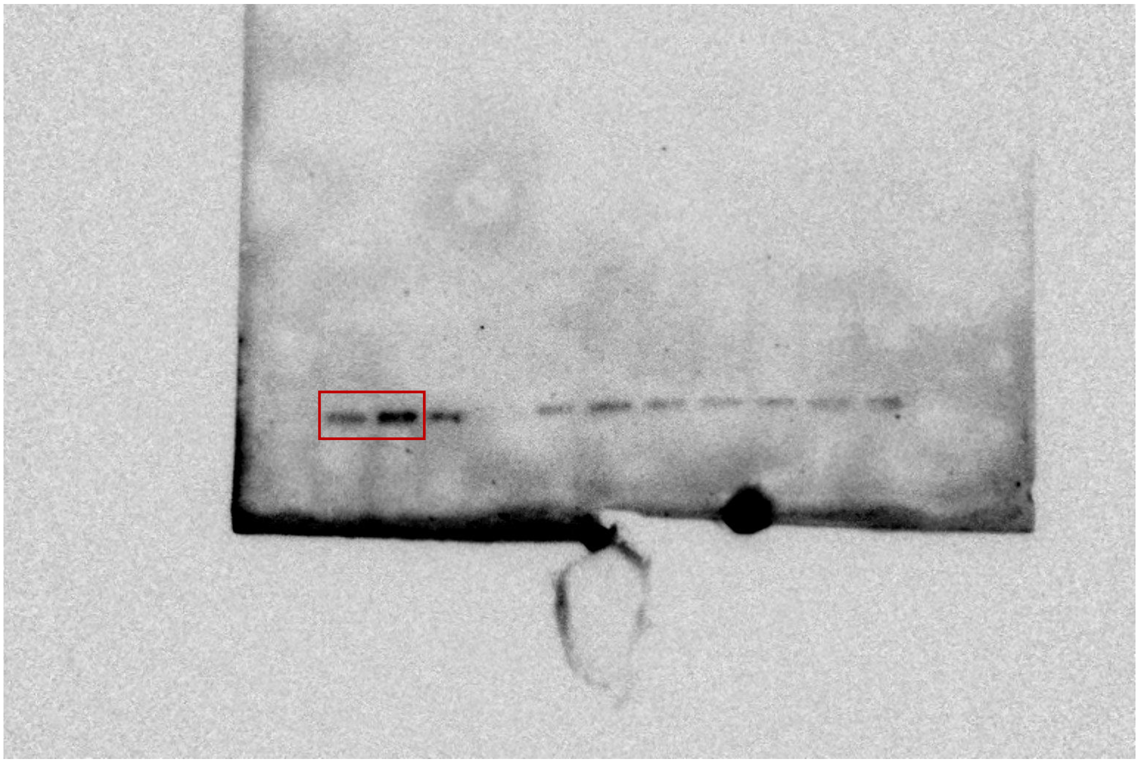

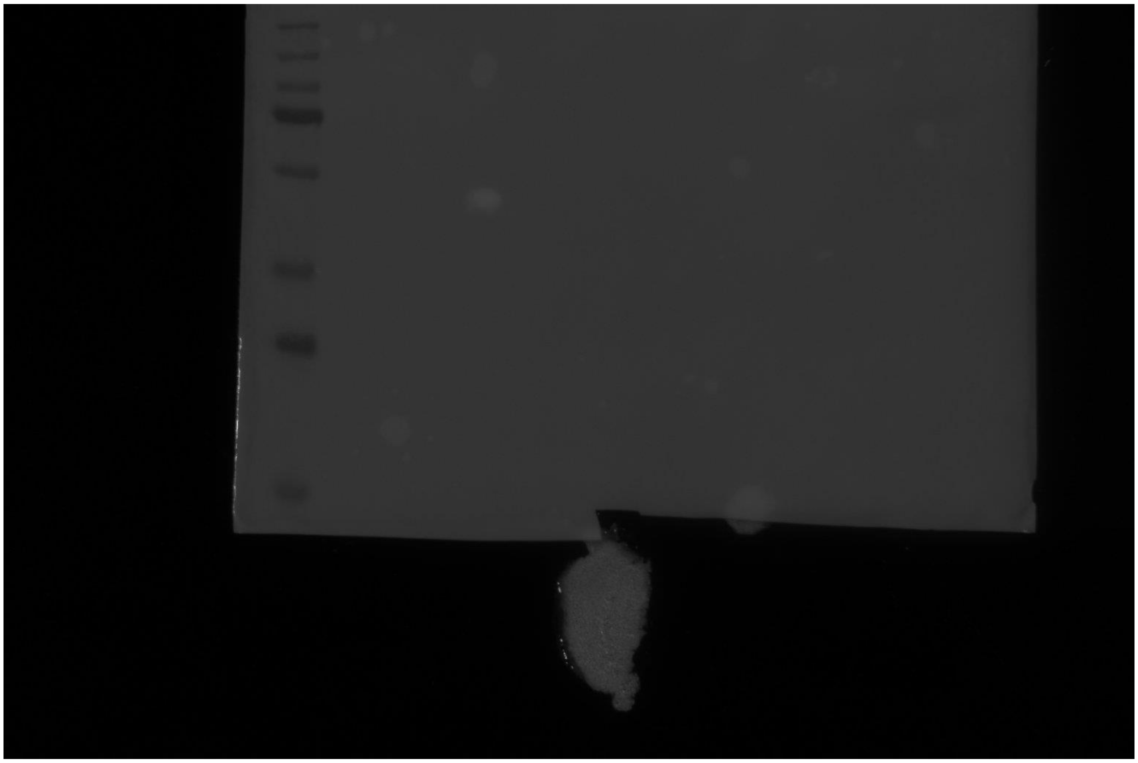

Supplementary figure 4B – UW228

PUMA

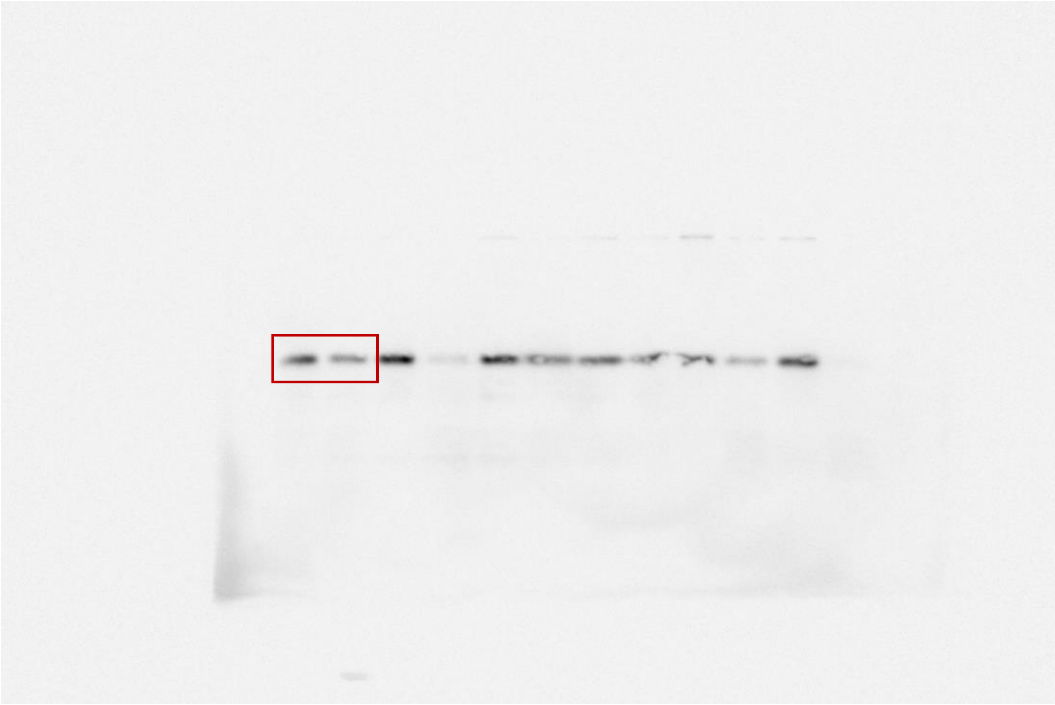

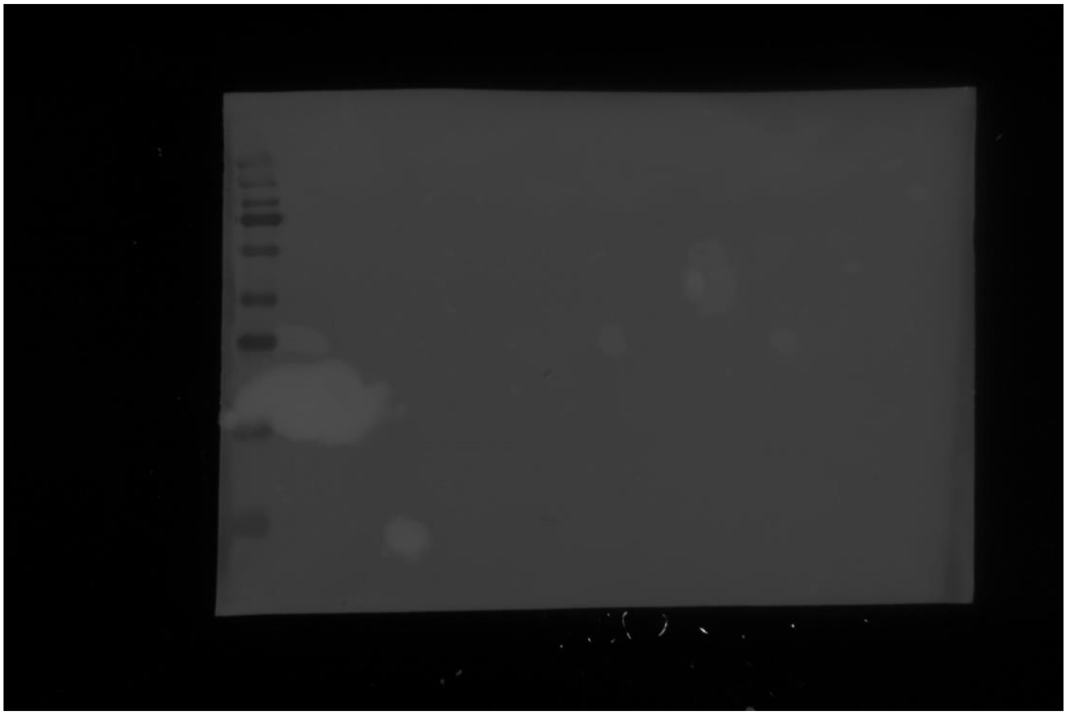

Supplementary figure 4B – UW228

NOXA

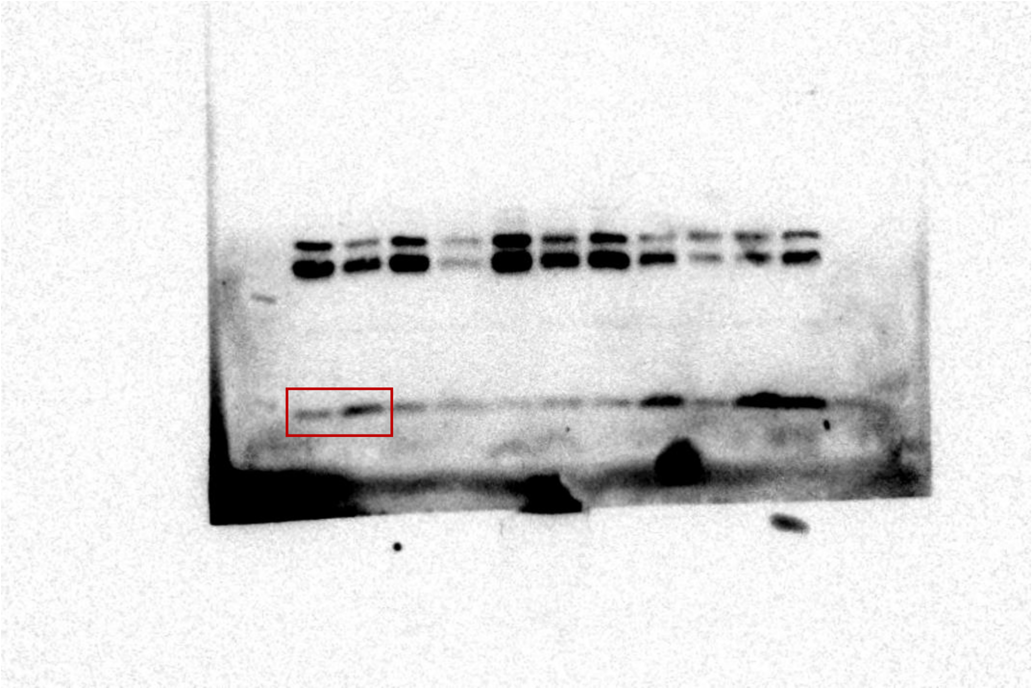

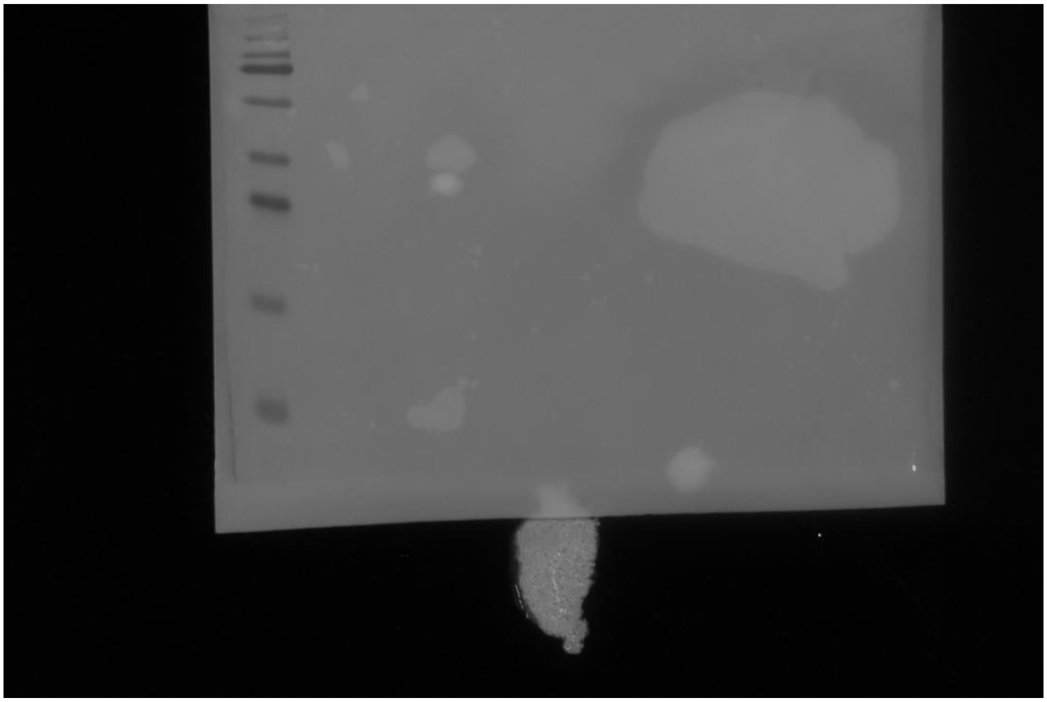

Supplementary figure 4B – UW228

GAPDH

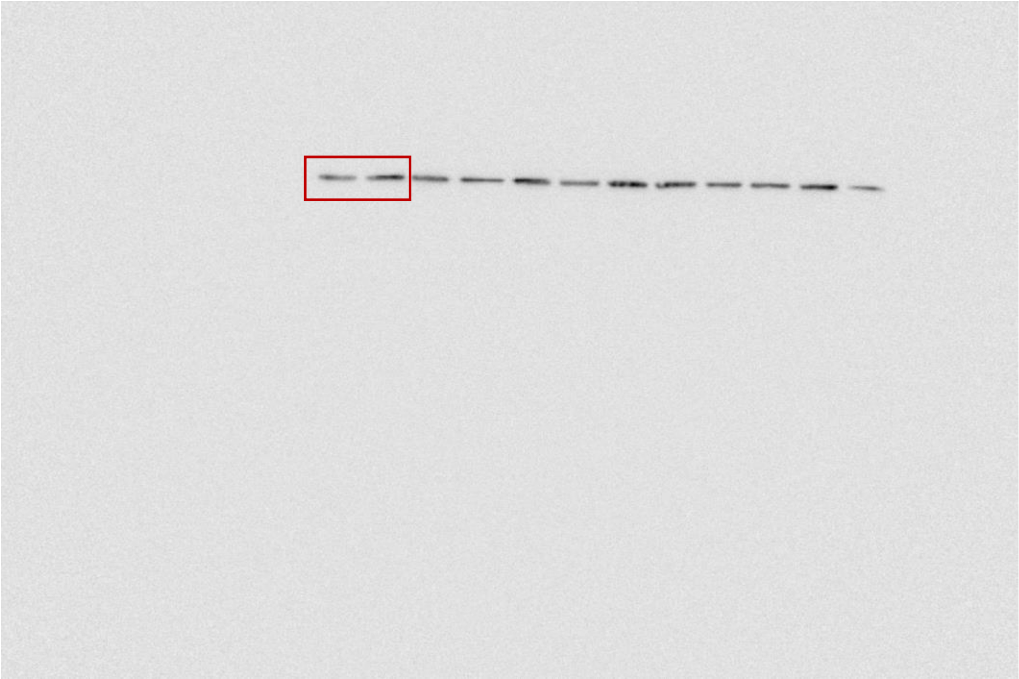

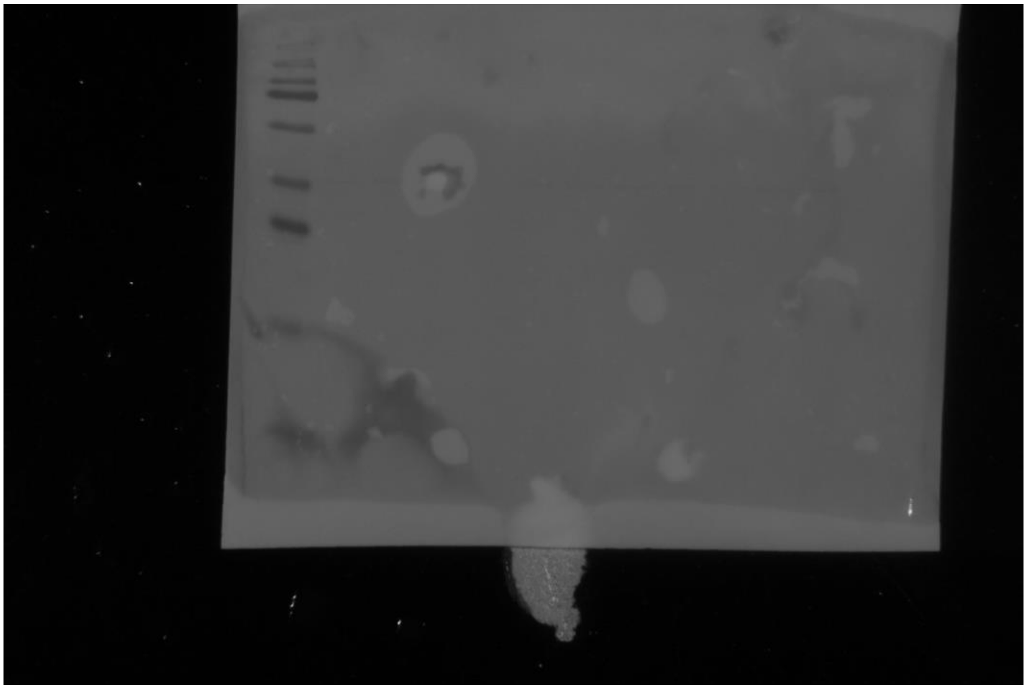

Supplementary figure 8A – ONS76

MCL-1

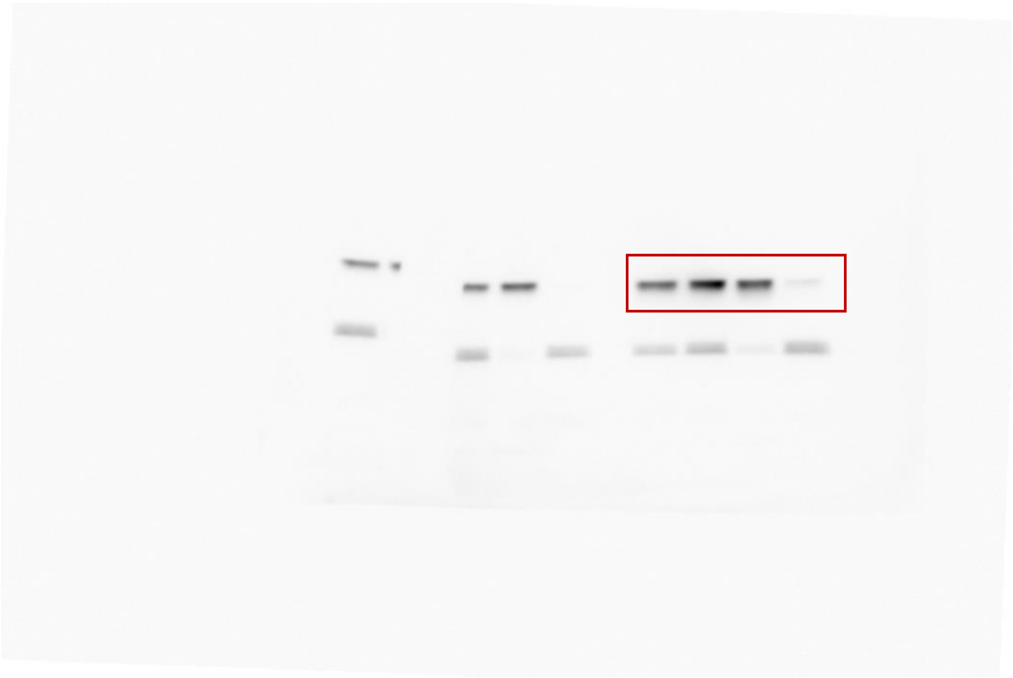

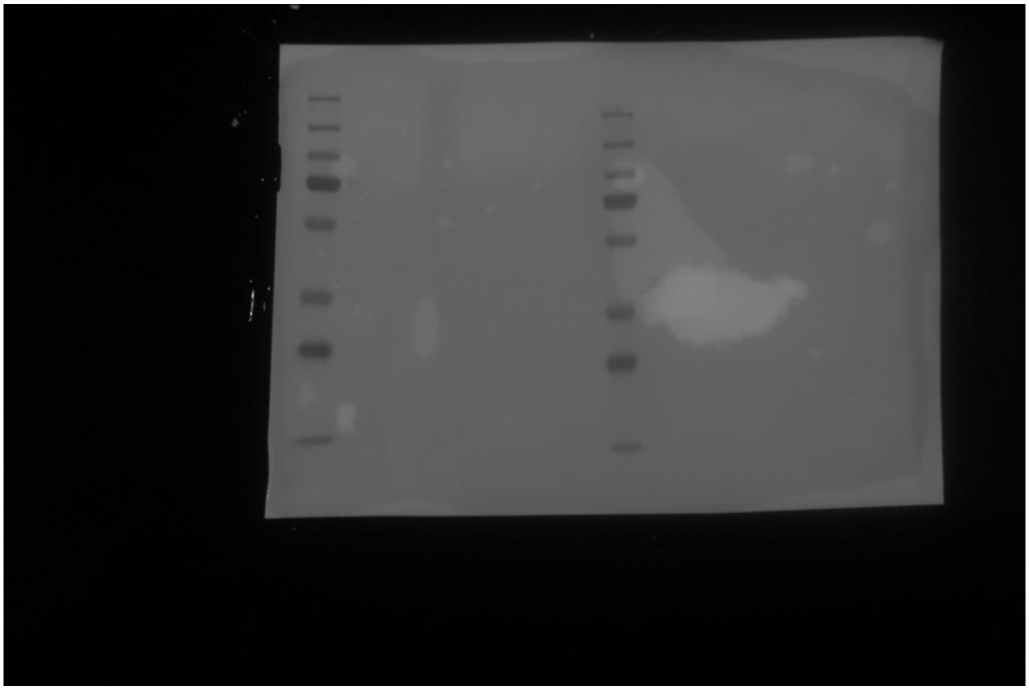

Supplementary figure 8A – ONS76

BCL-XL

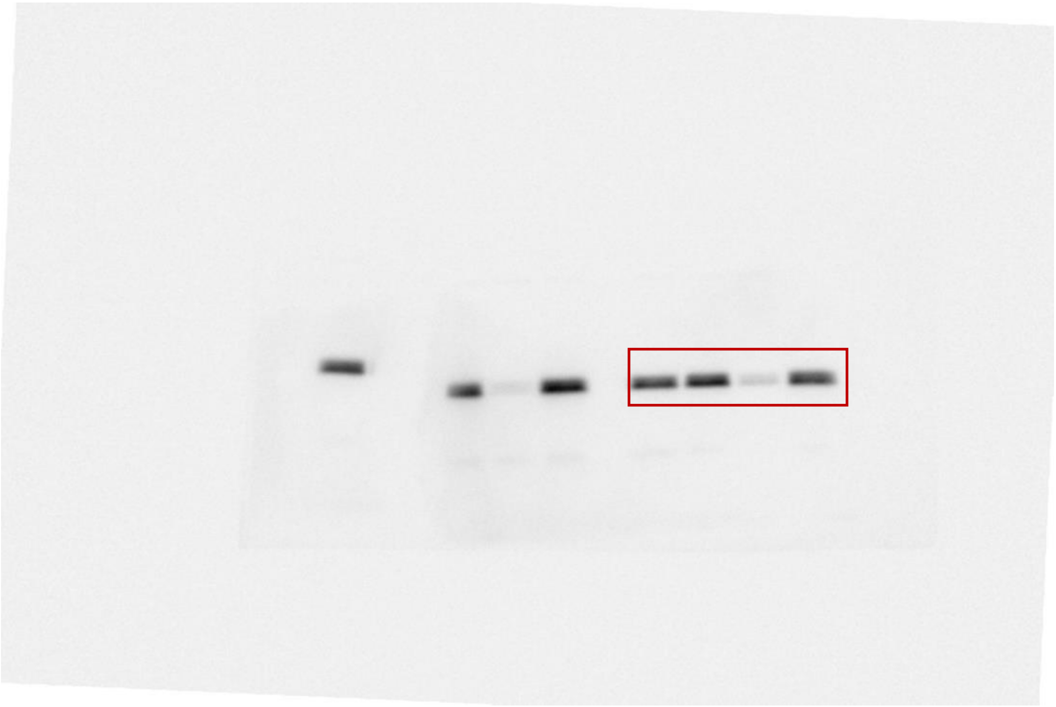

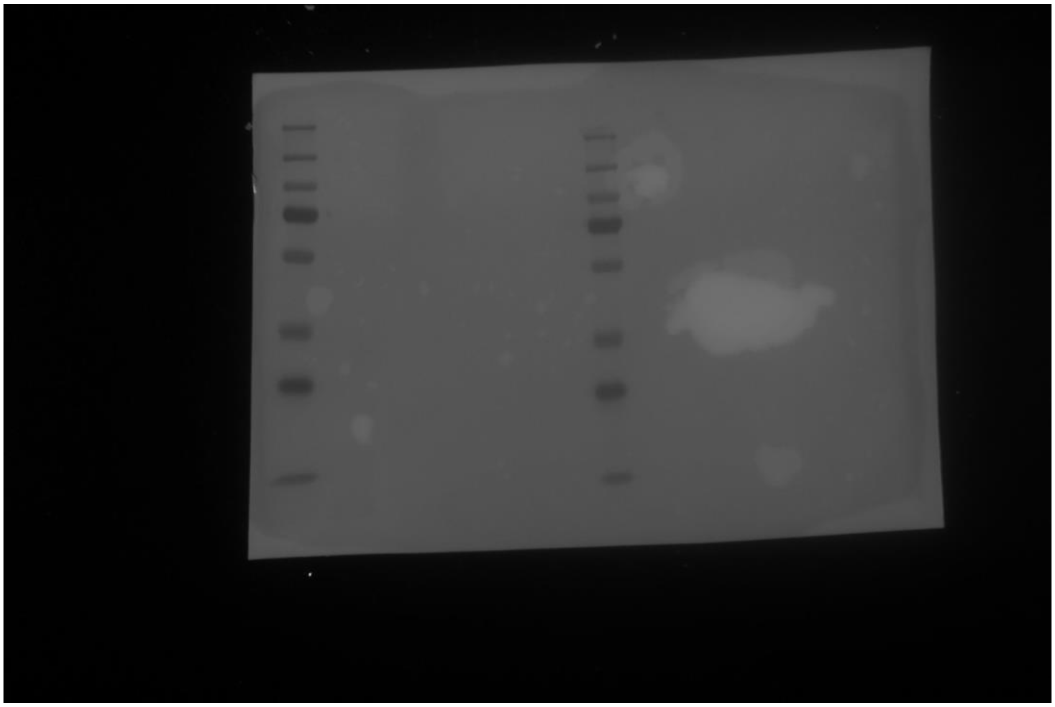

Supplementary figure 8A – ONS76

GAPDH

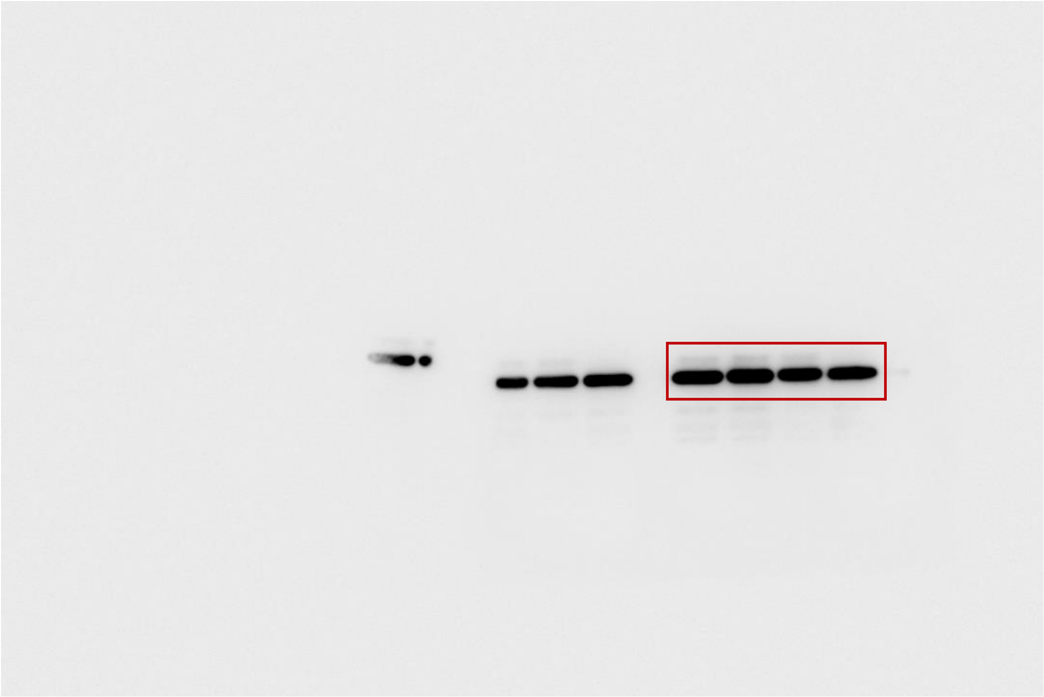

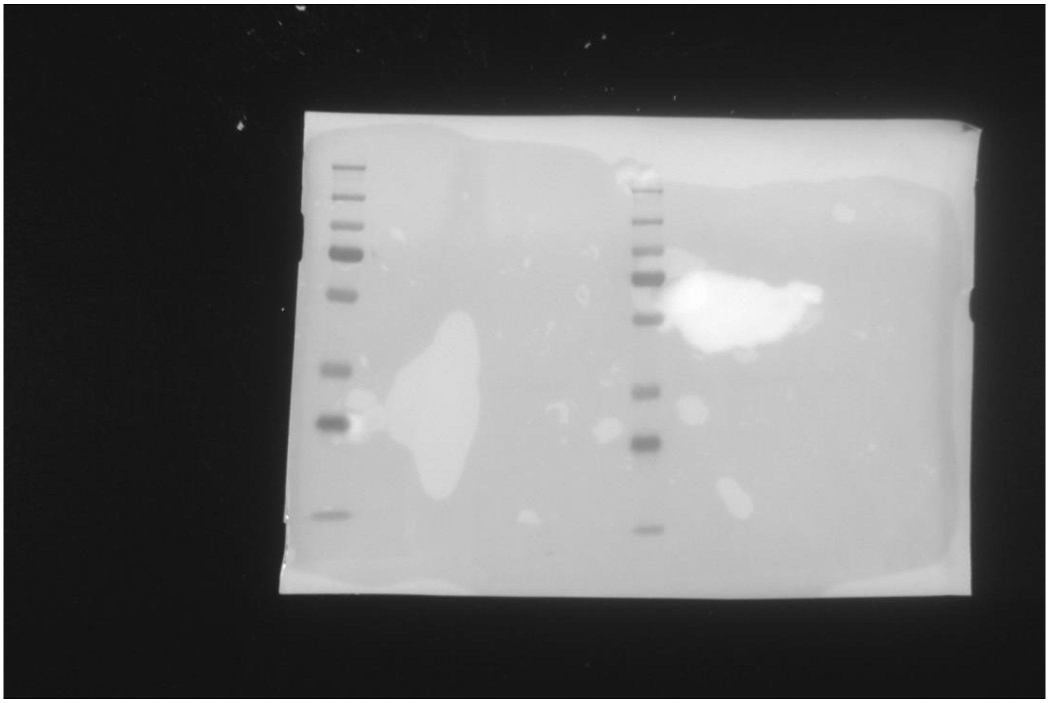

Supplementary figure 8B – UW228

MCL-1

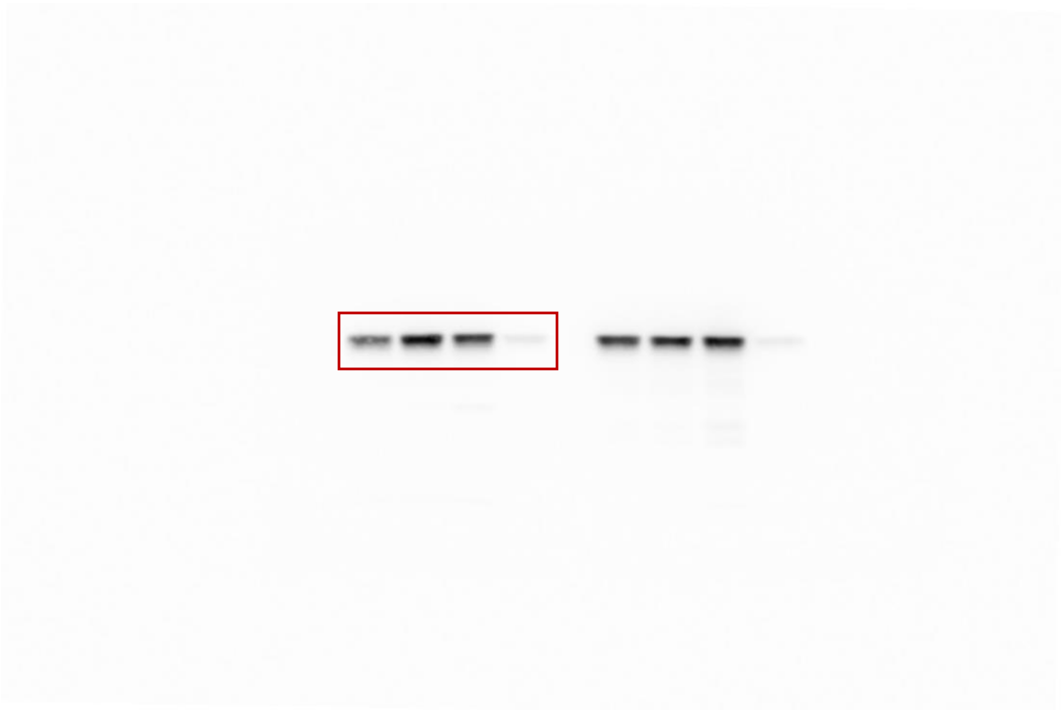

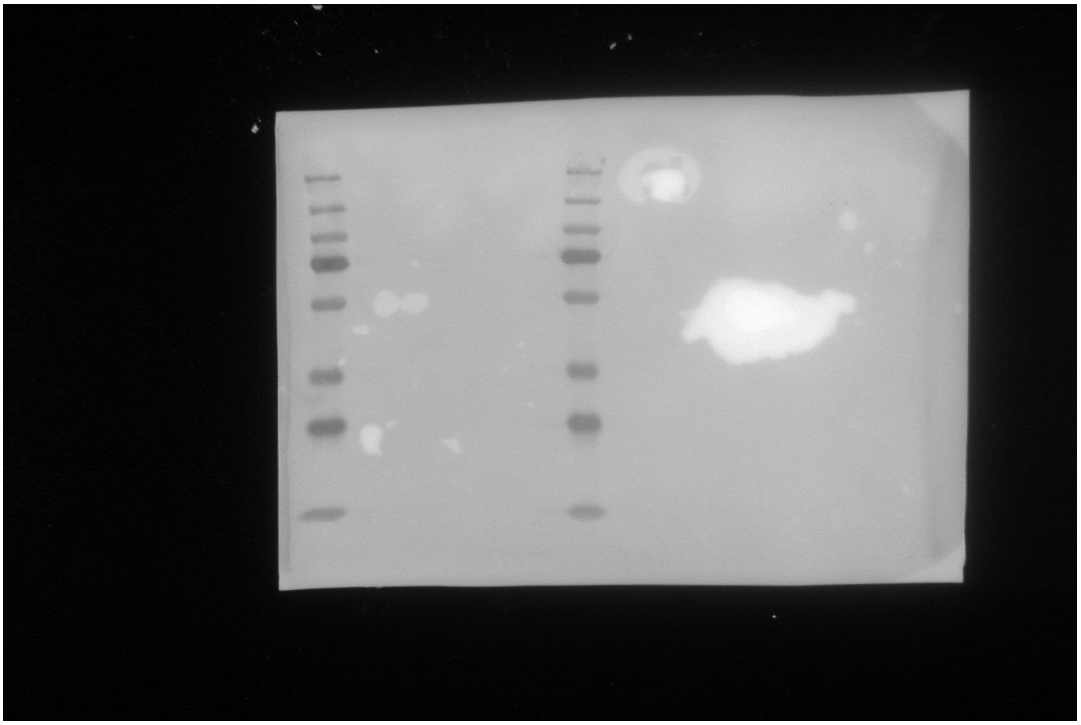

Supplementary figure 8B – UW228

BCL-XL

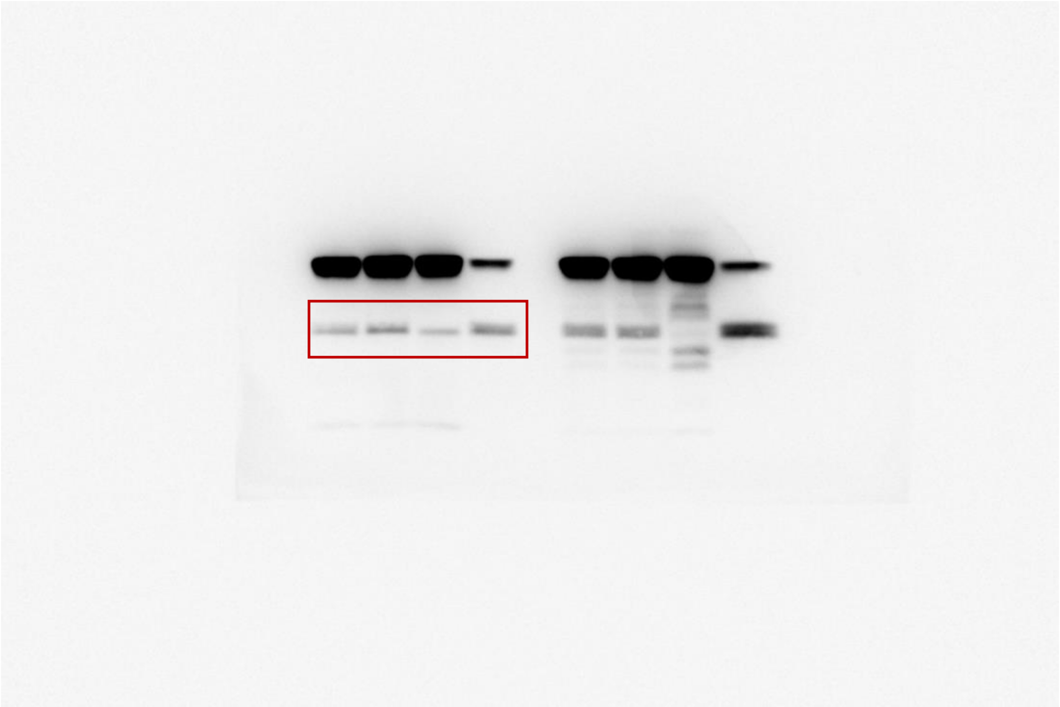

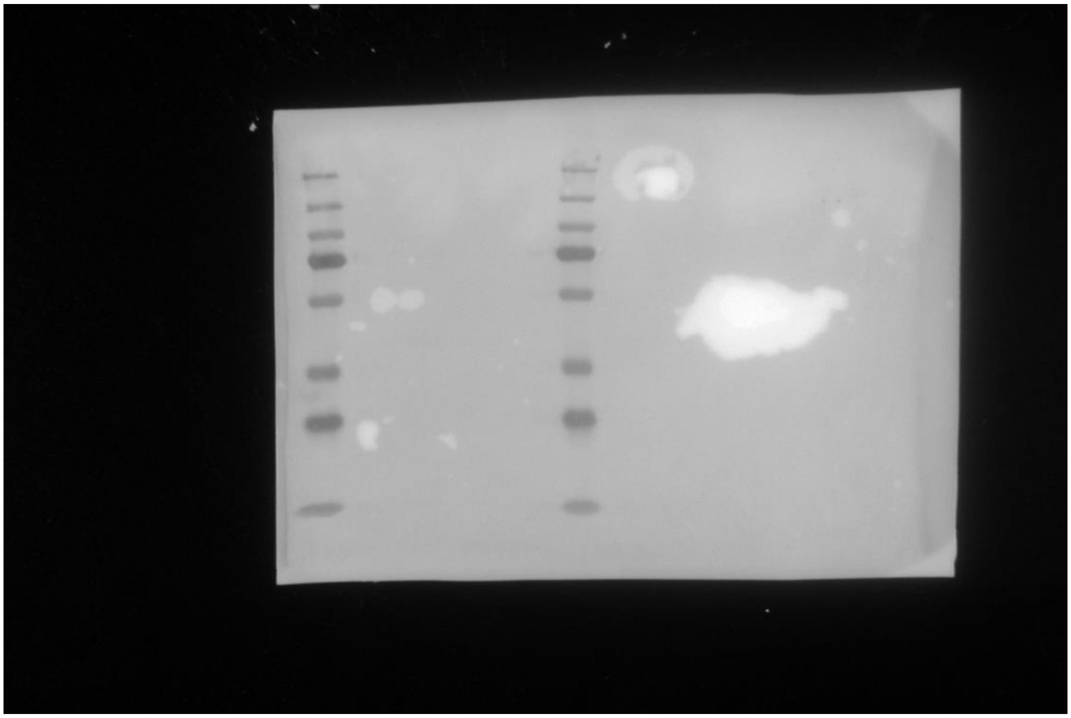

Supplementary figure 8B – UW228

GAPDH

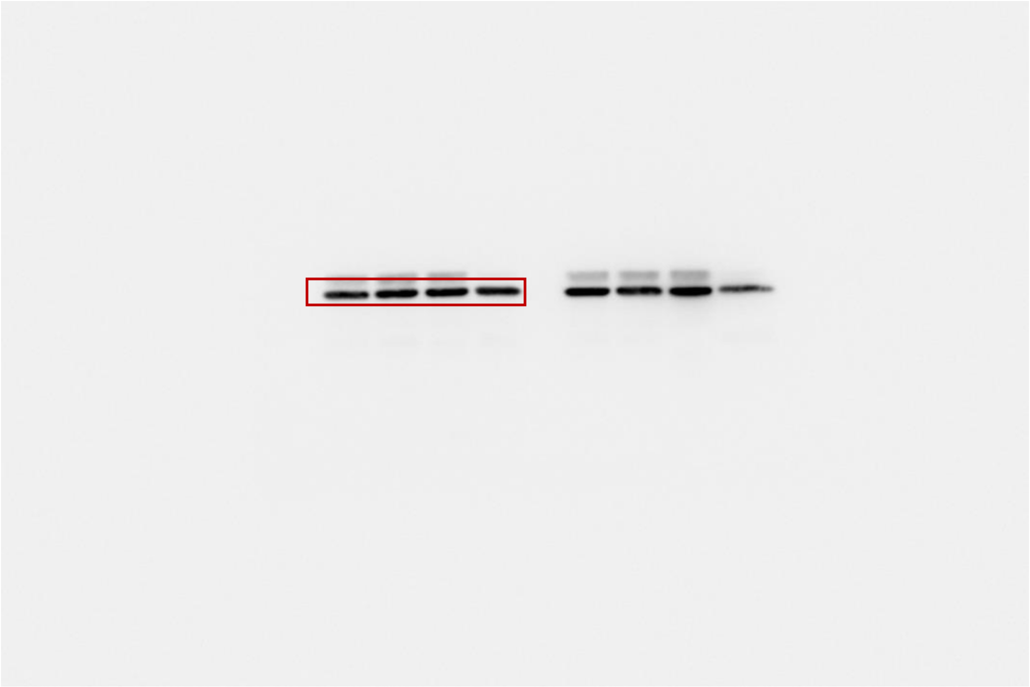

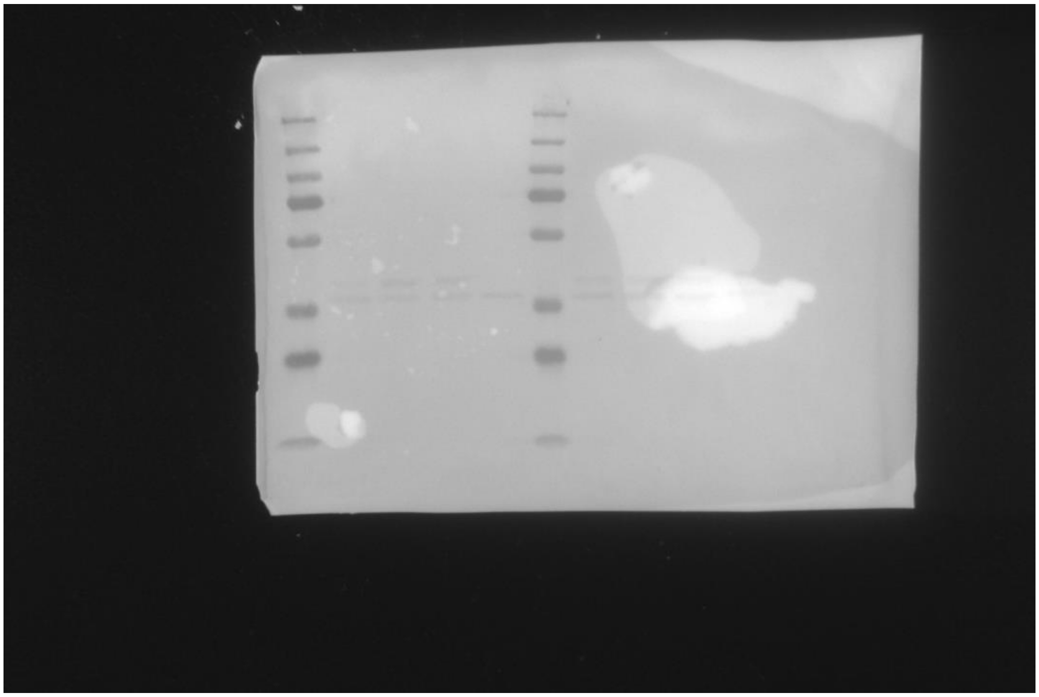

Figure 9A – ONS76

Caspase 3

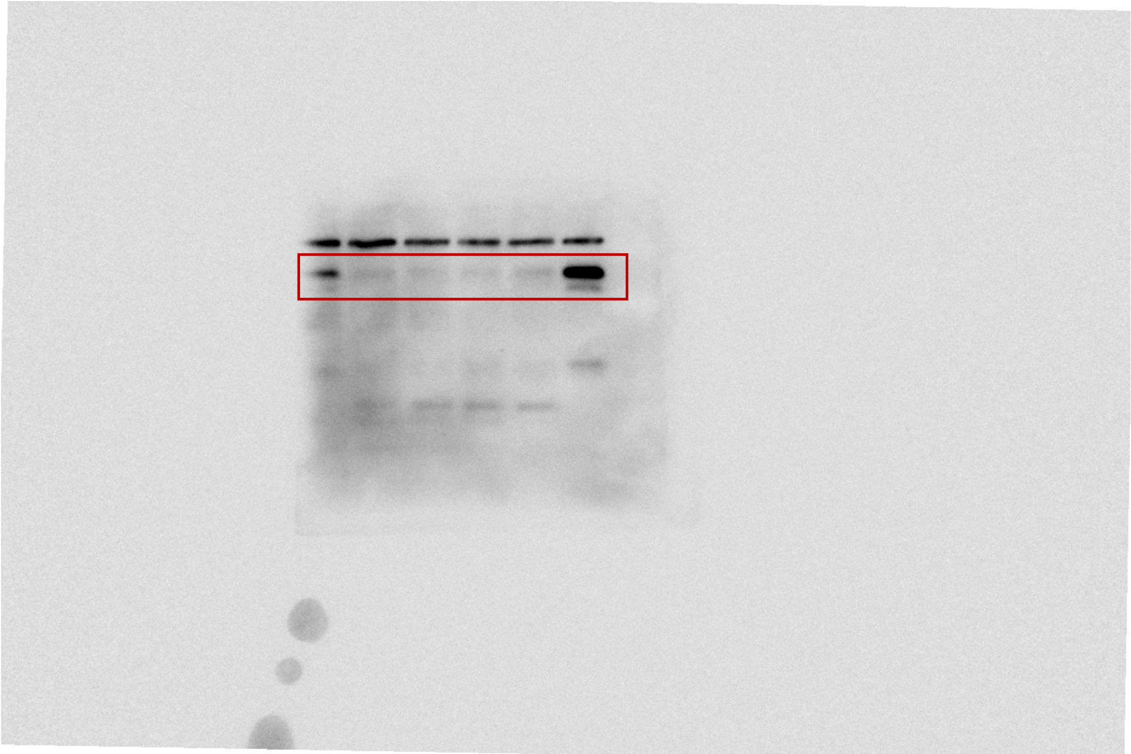

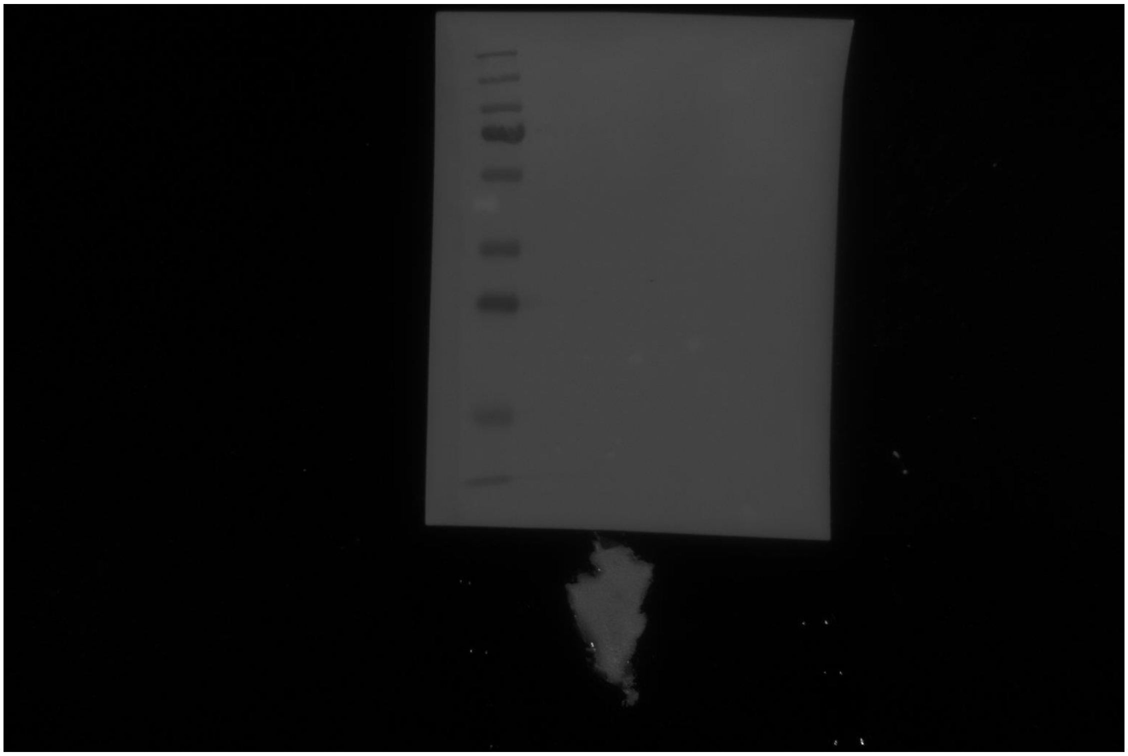

Figure 9A – ONS76

Cleaved caspase 3

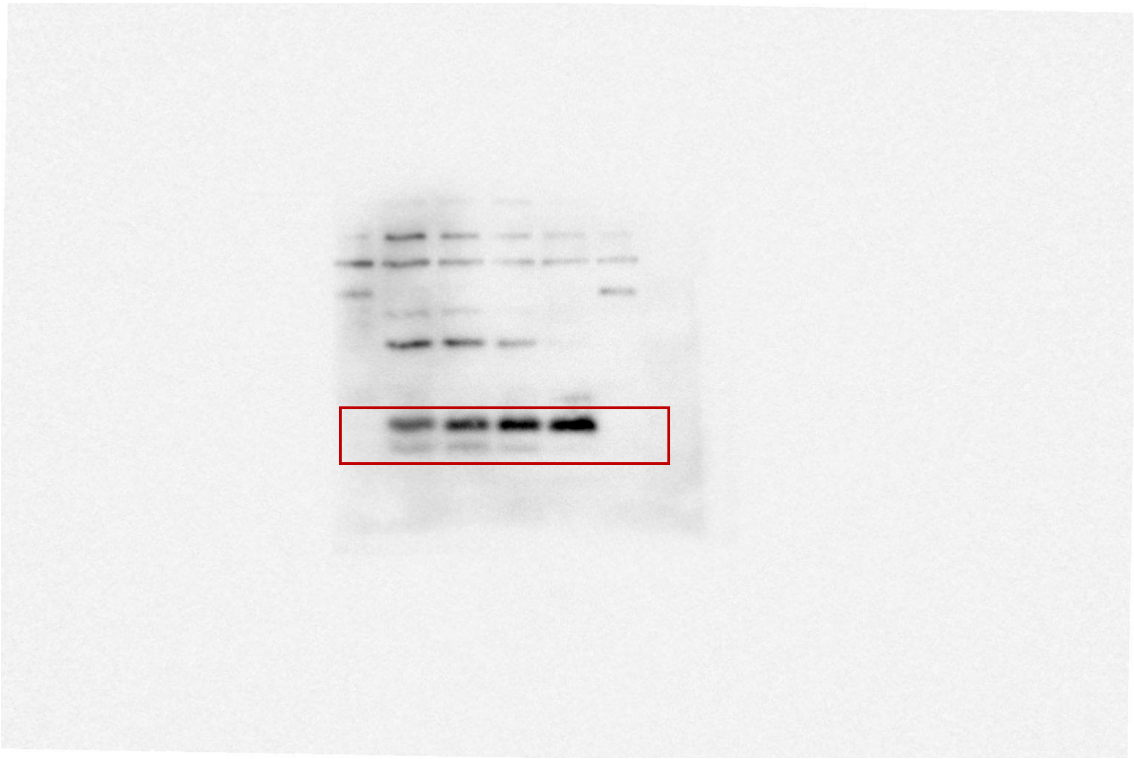

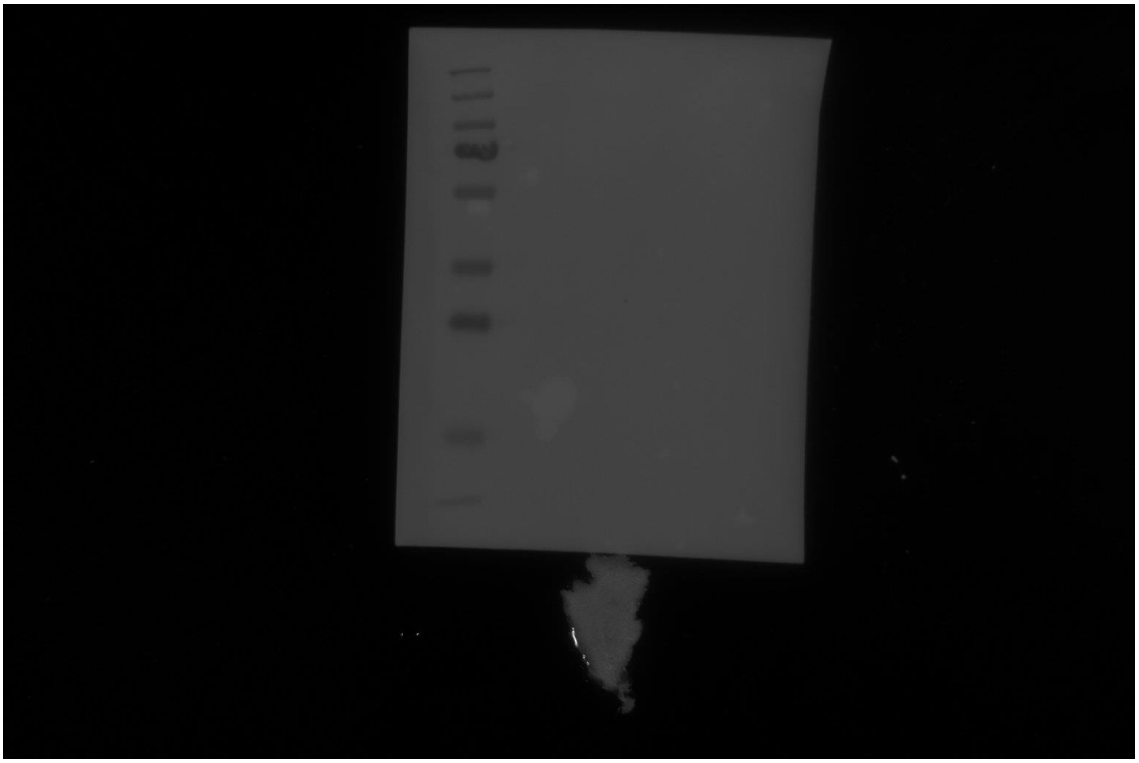

Figure 9A – ONS76

GAPDH

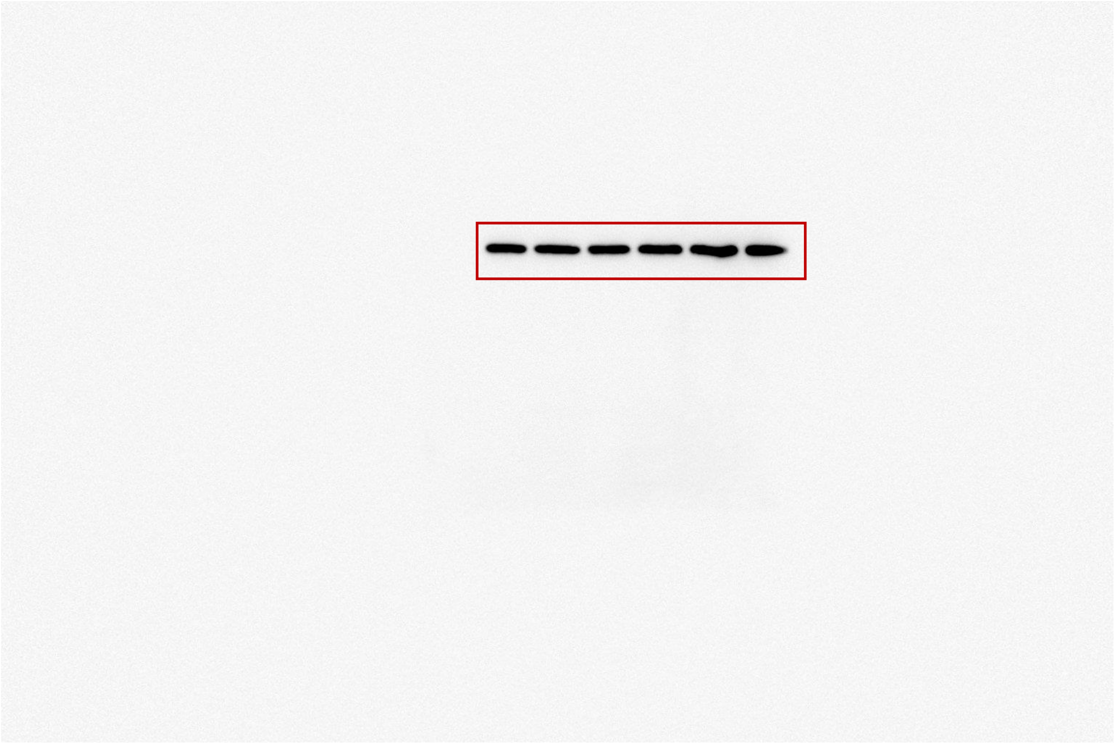

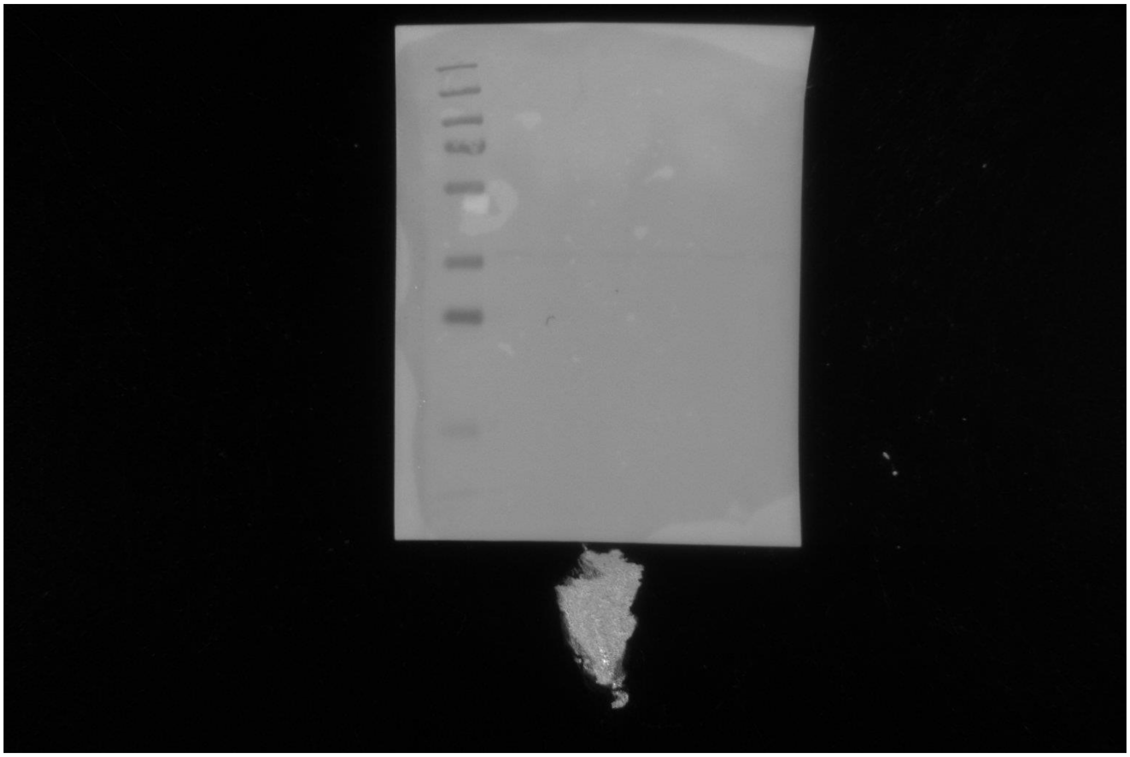

Figure 9A – ONS76

Caspase 7

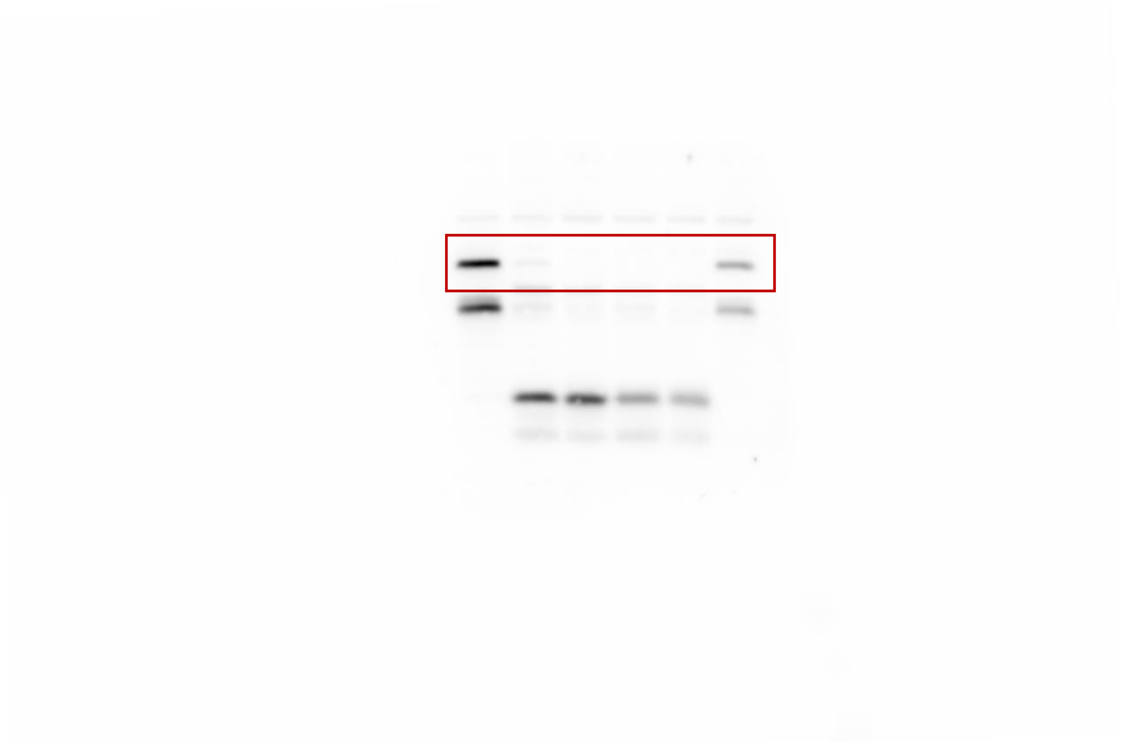

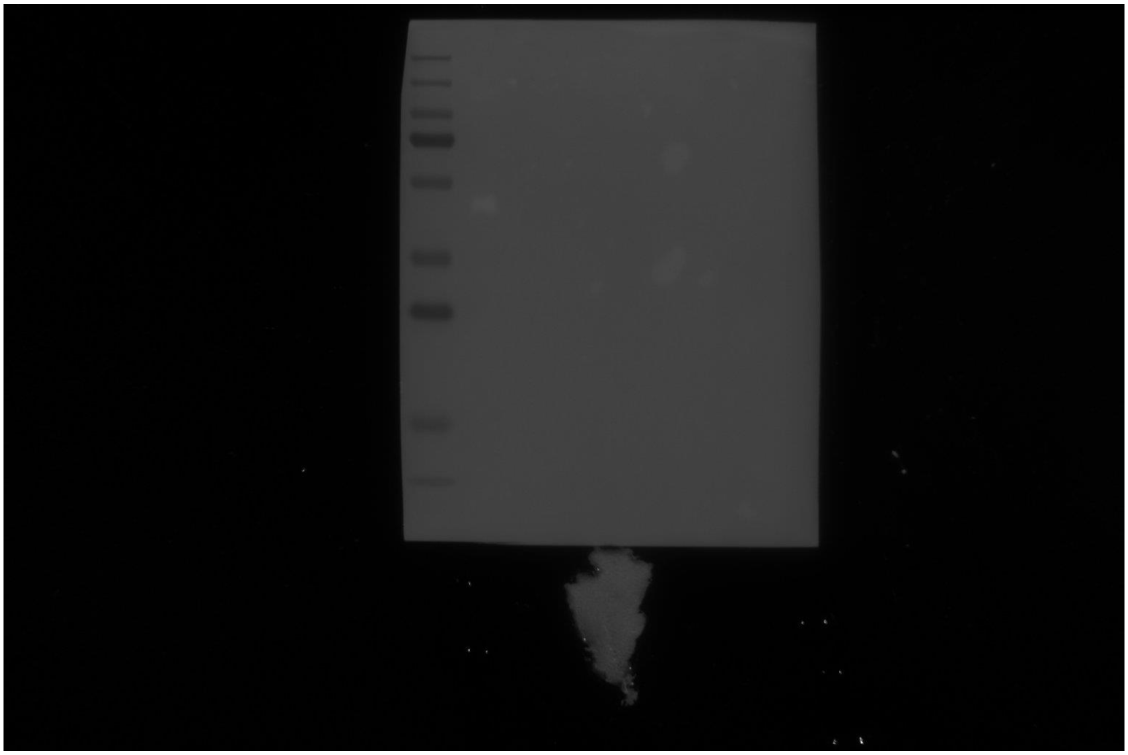

Figure 9A – ONS76

Cleaved caspase 7

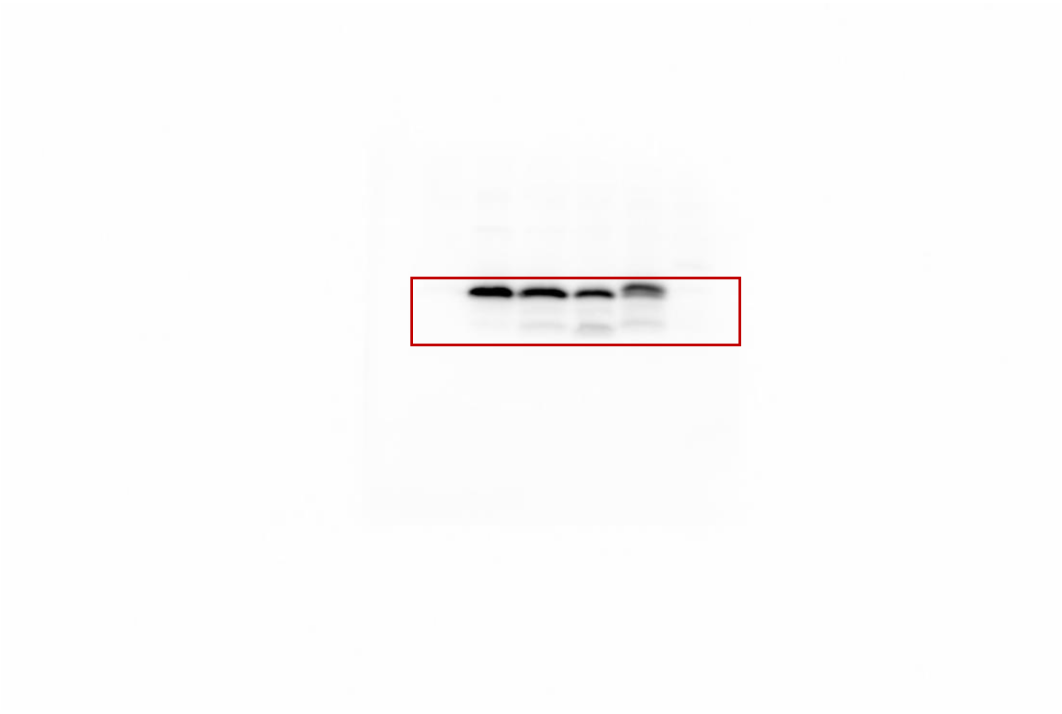

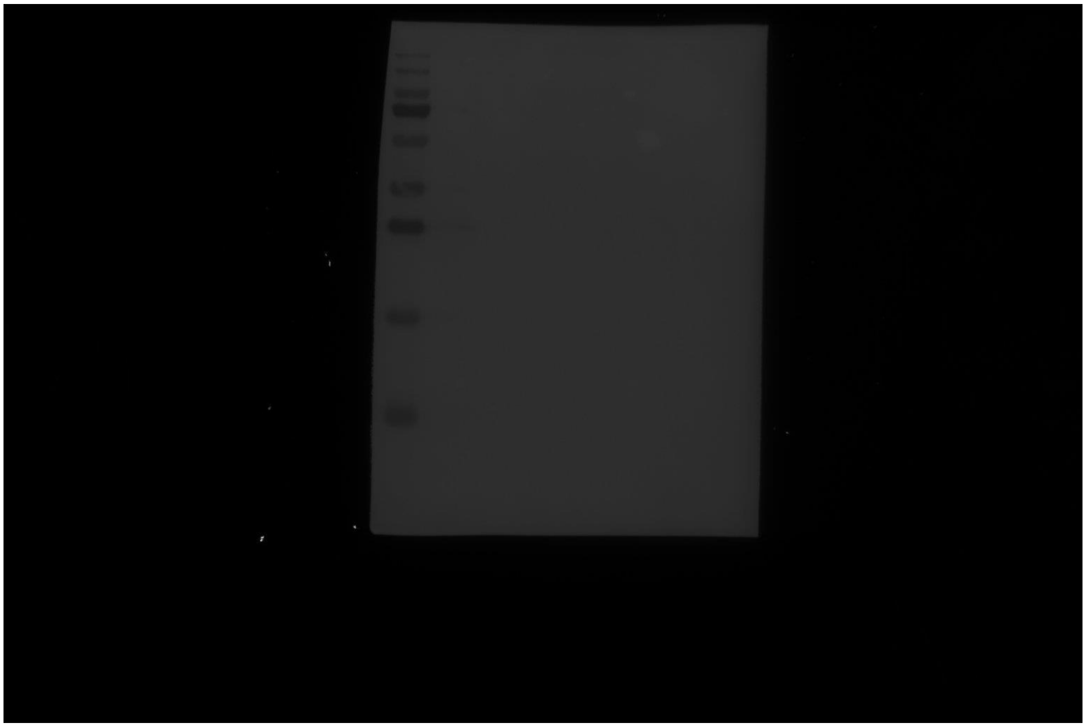

Figure 9A – ONS76

GAPDH

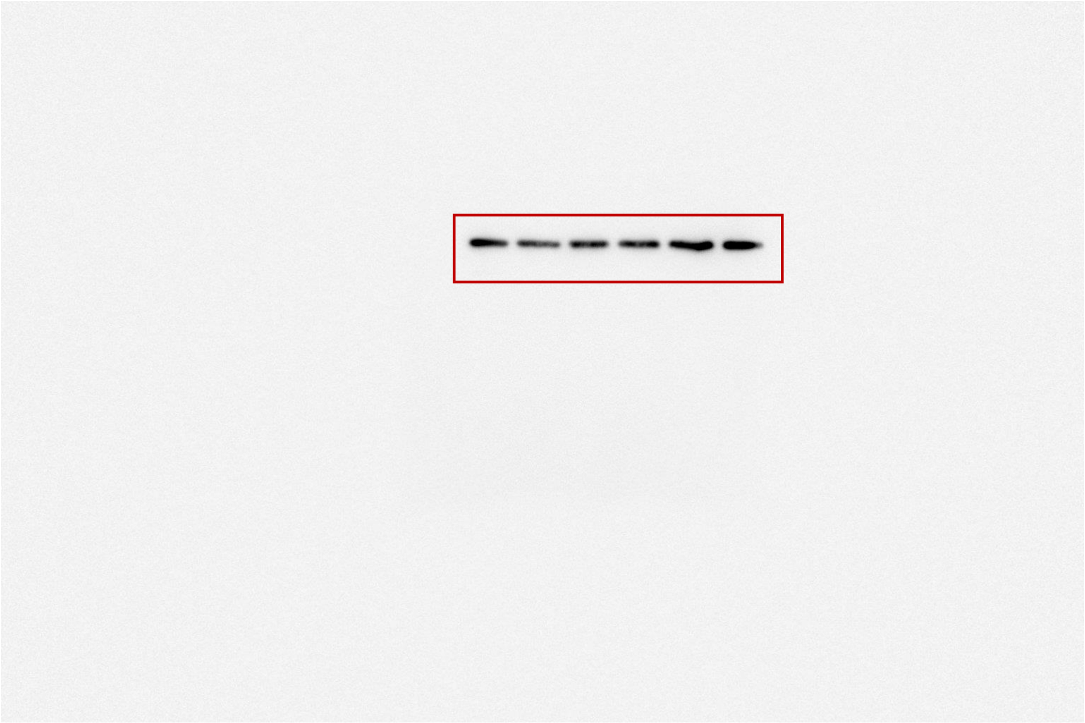

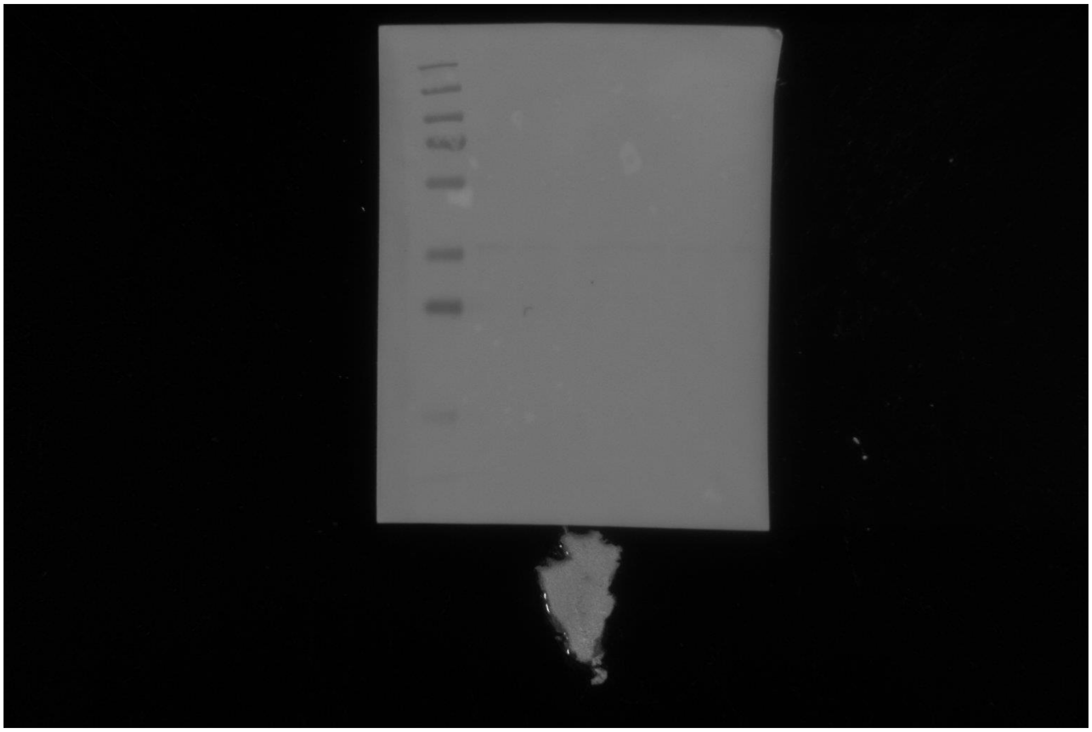

Figure 9A – ONS76

Caspase 9/cleaved caspase 9

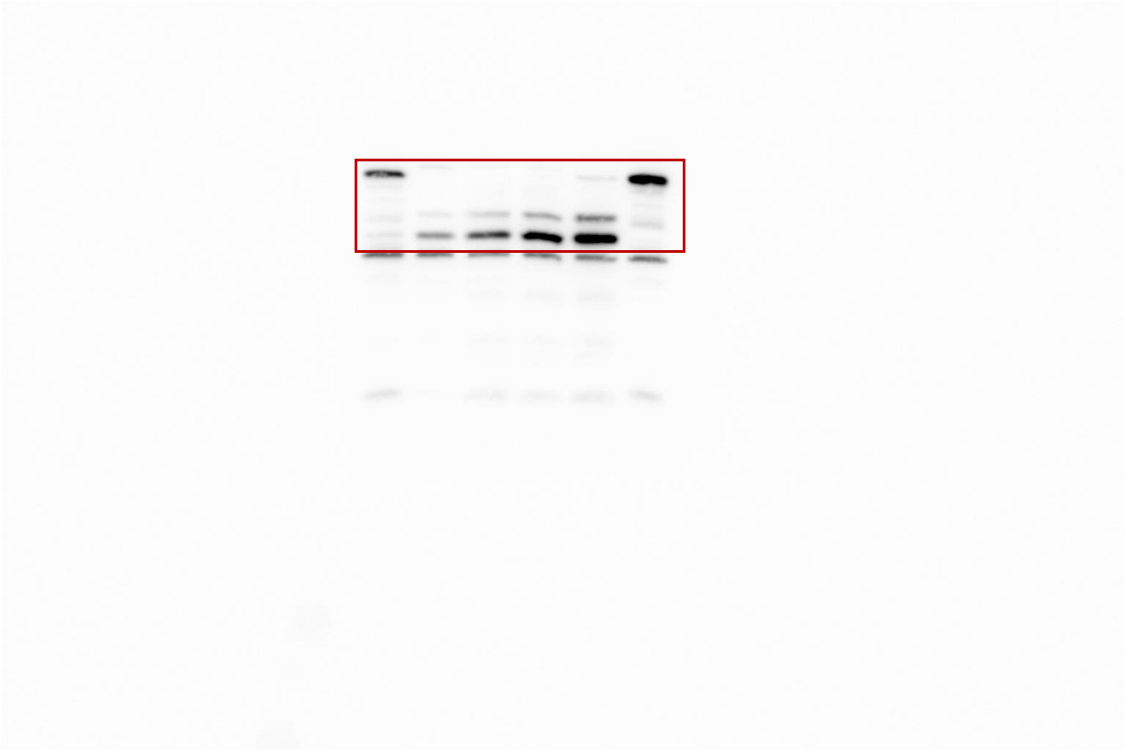

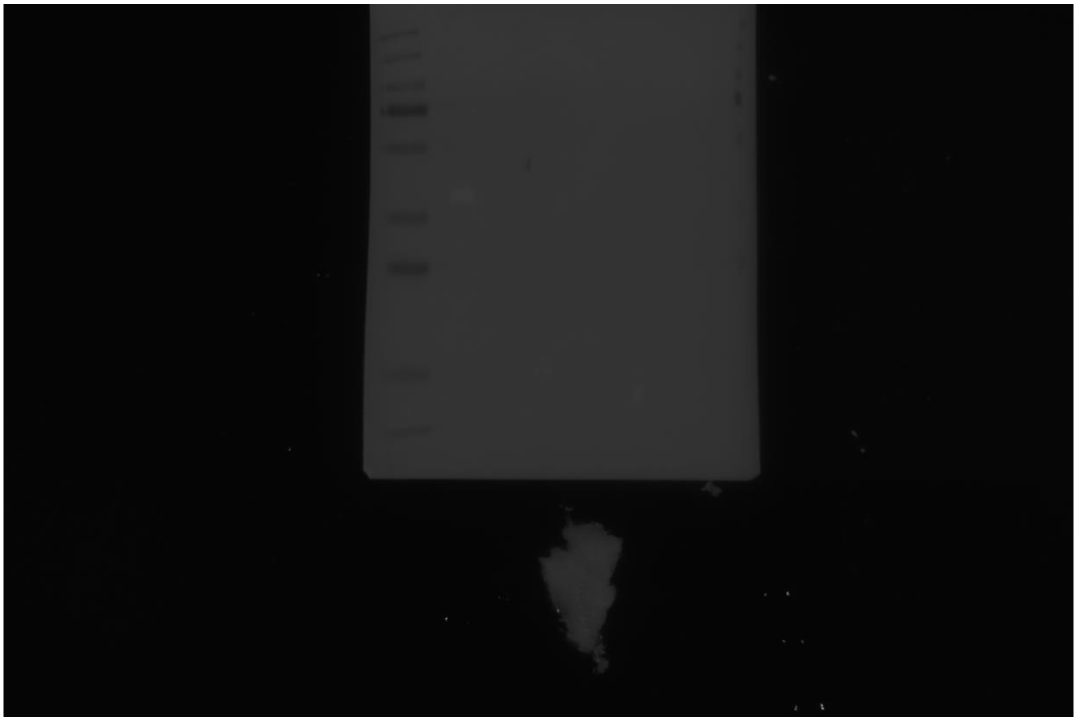

Figure 9A – ONS76

GAPDH

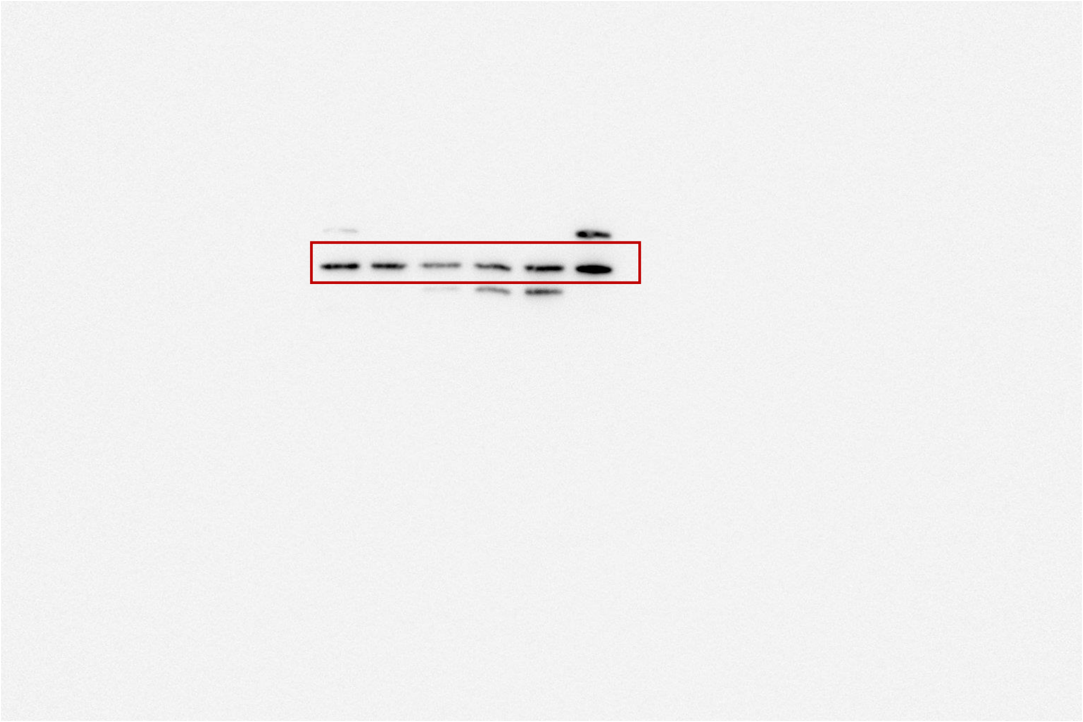

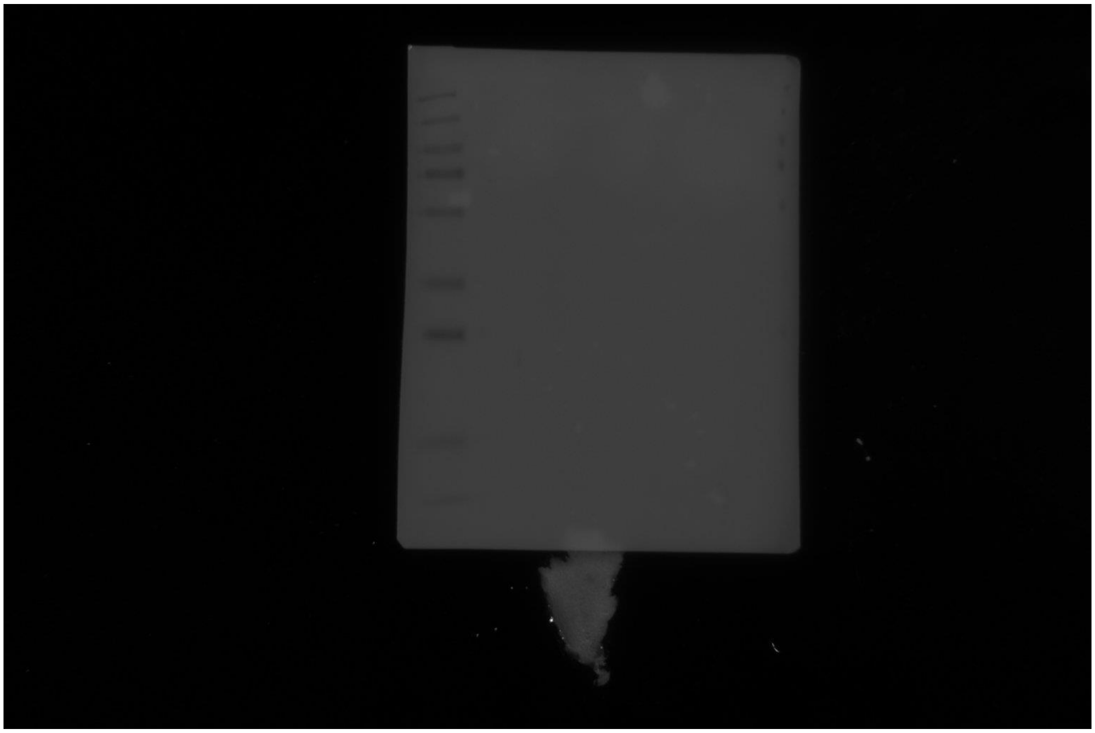

Figure 9B – UW228

Caspase 3

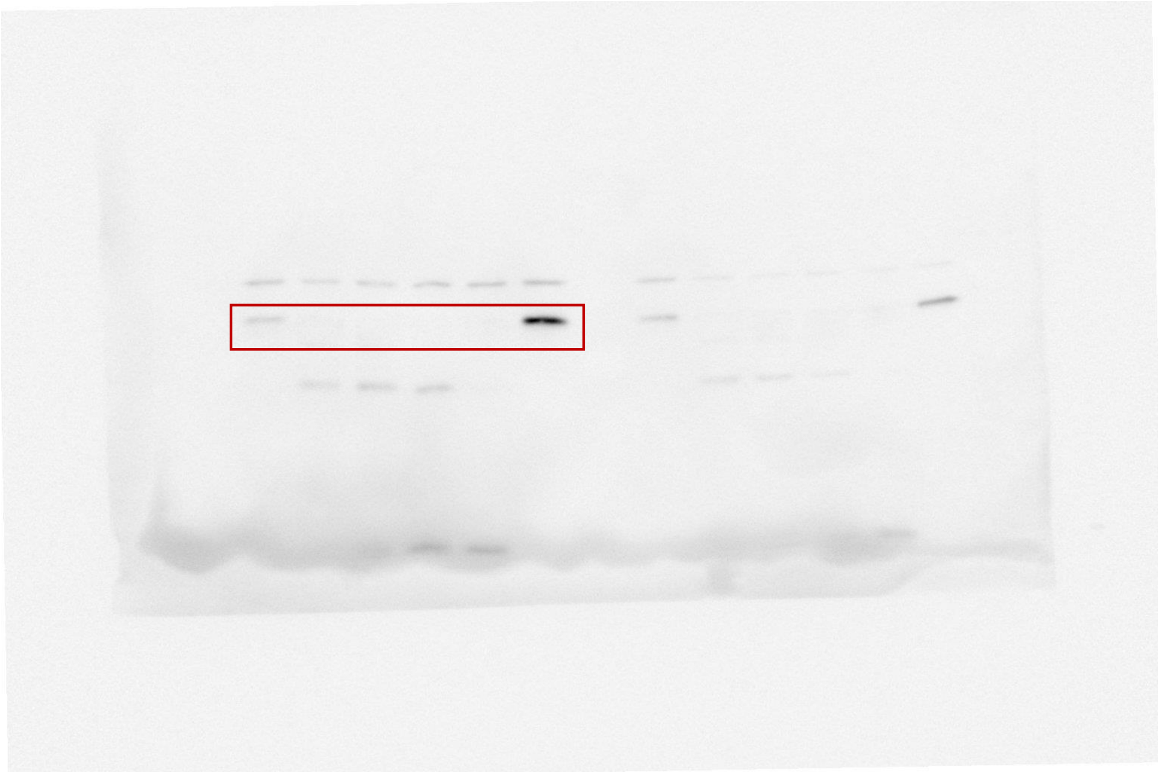

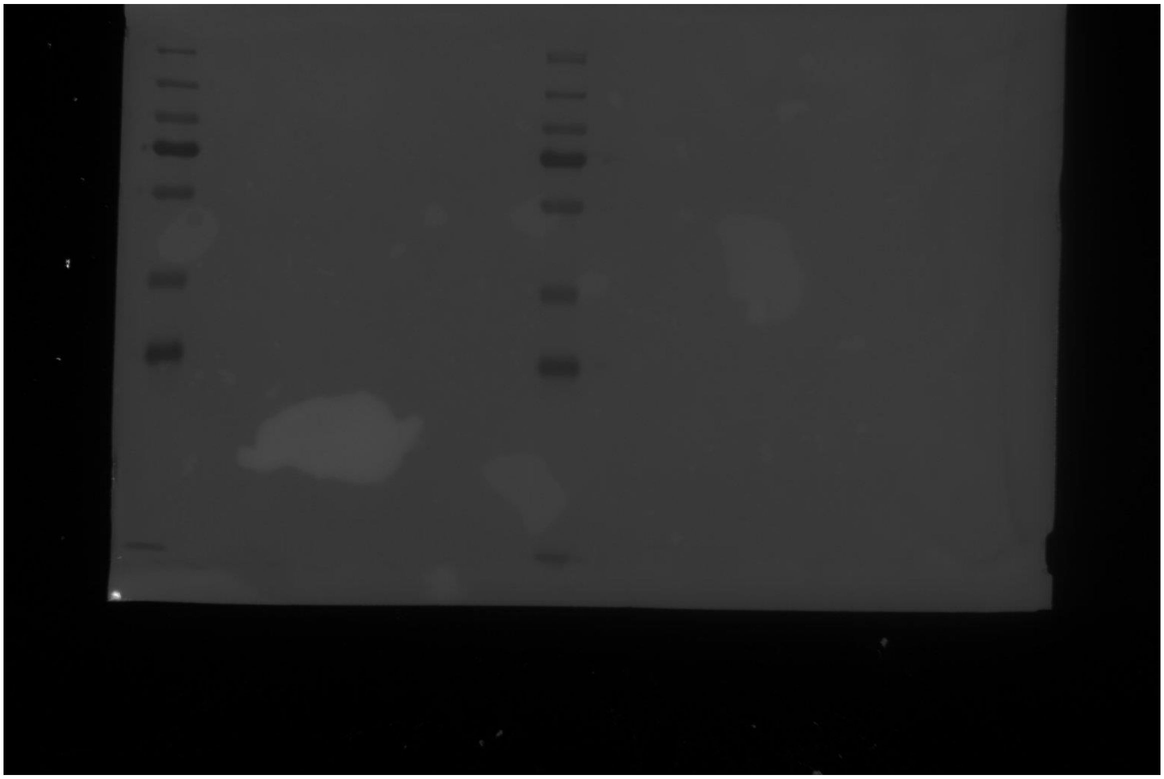

Figure 9B – UW228

Cleaved caspase 3

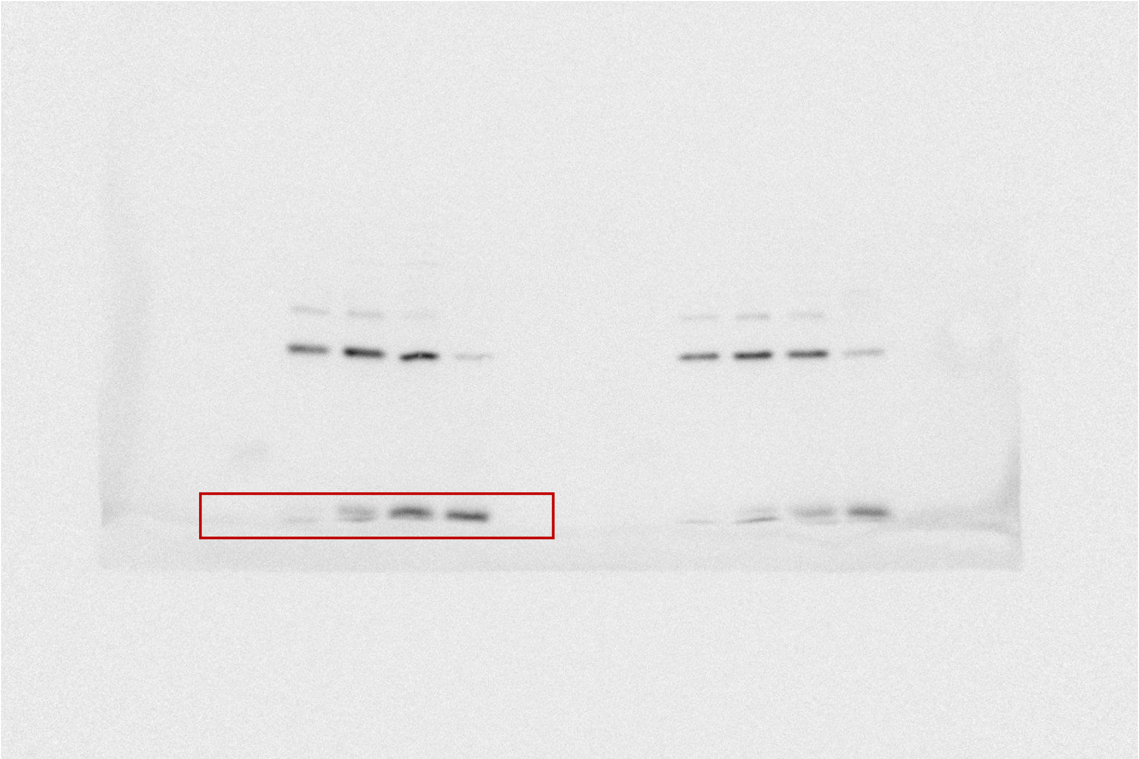

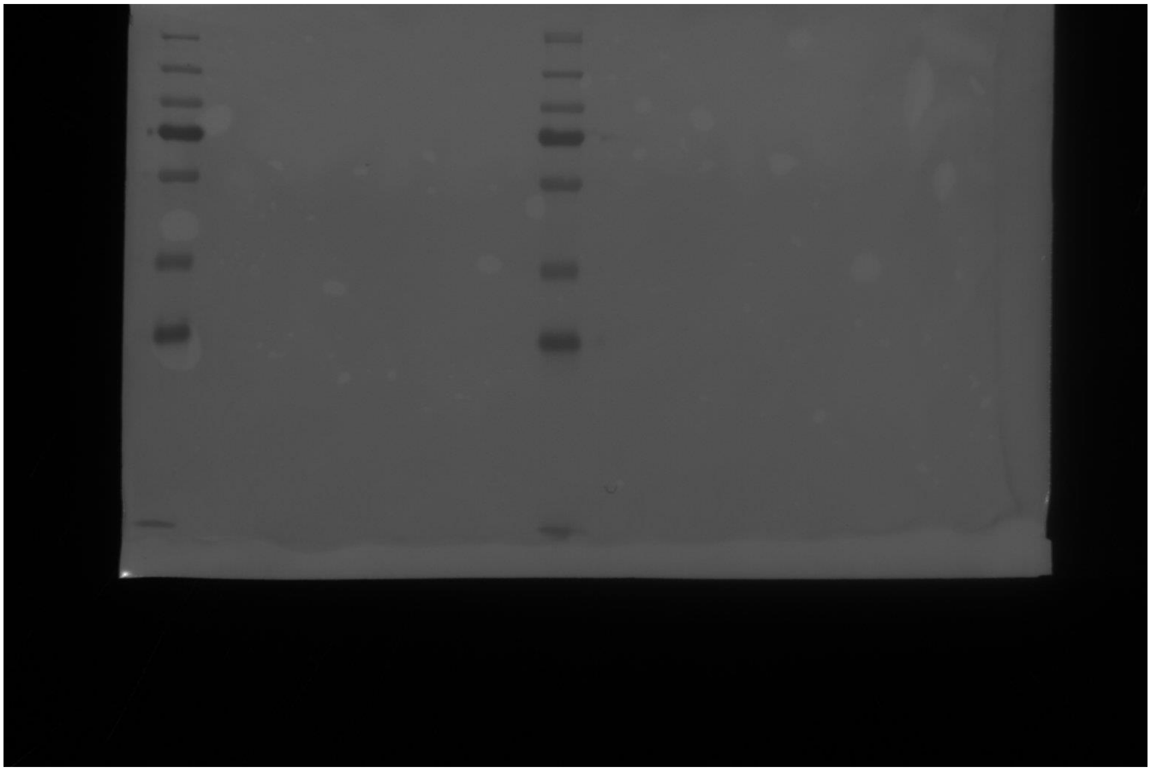

Figure 9B – UW228

GAPDH

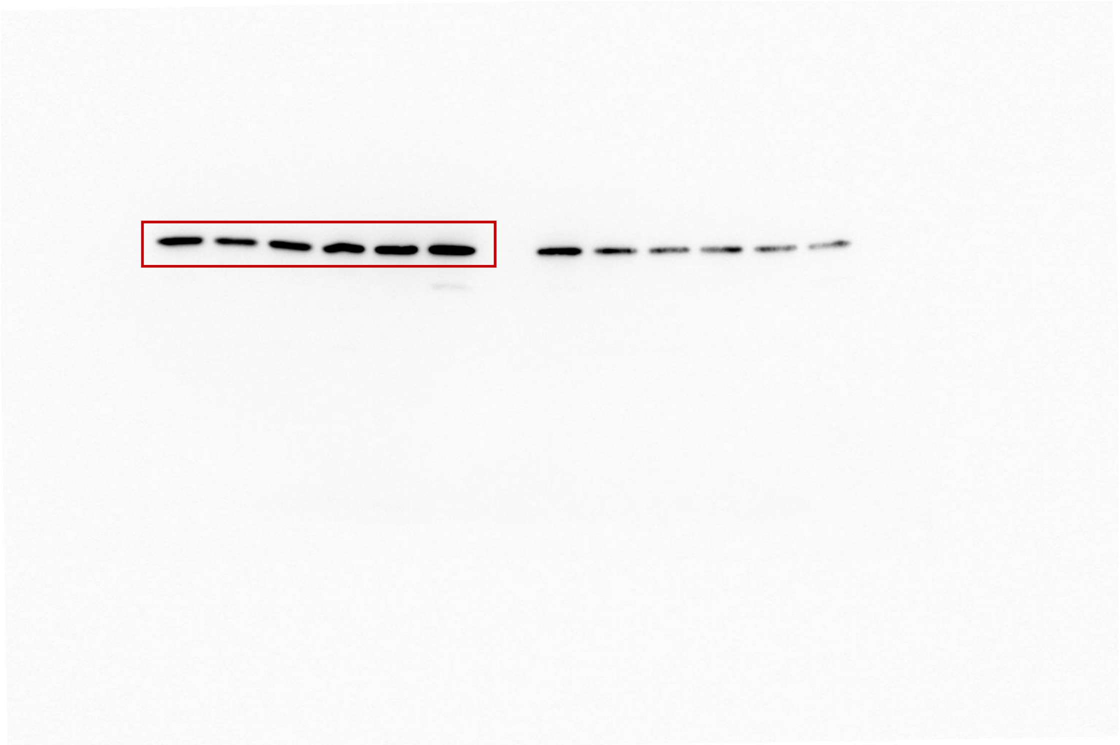

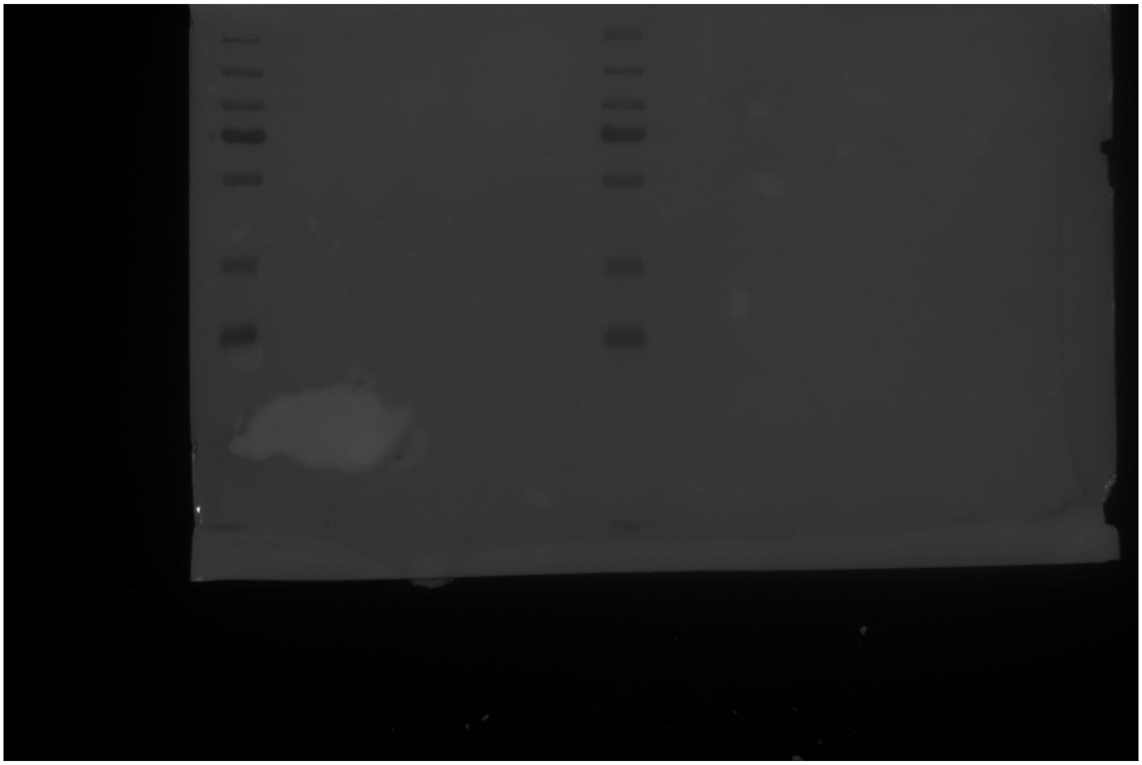

Figure 9B – UW228

Caspase 7/Cleaved caspase 7

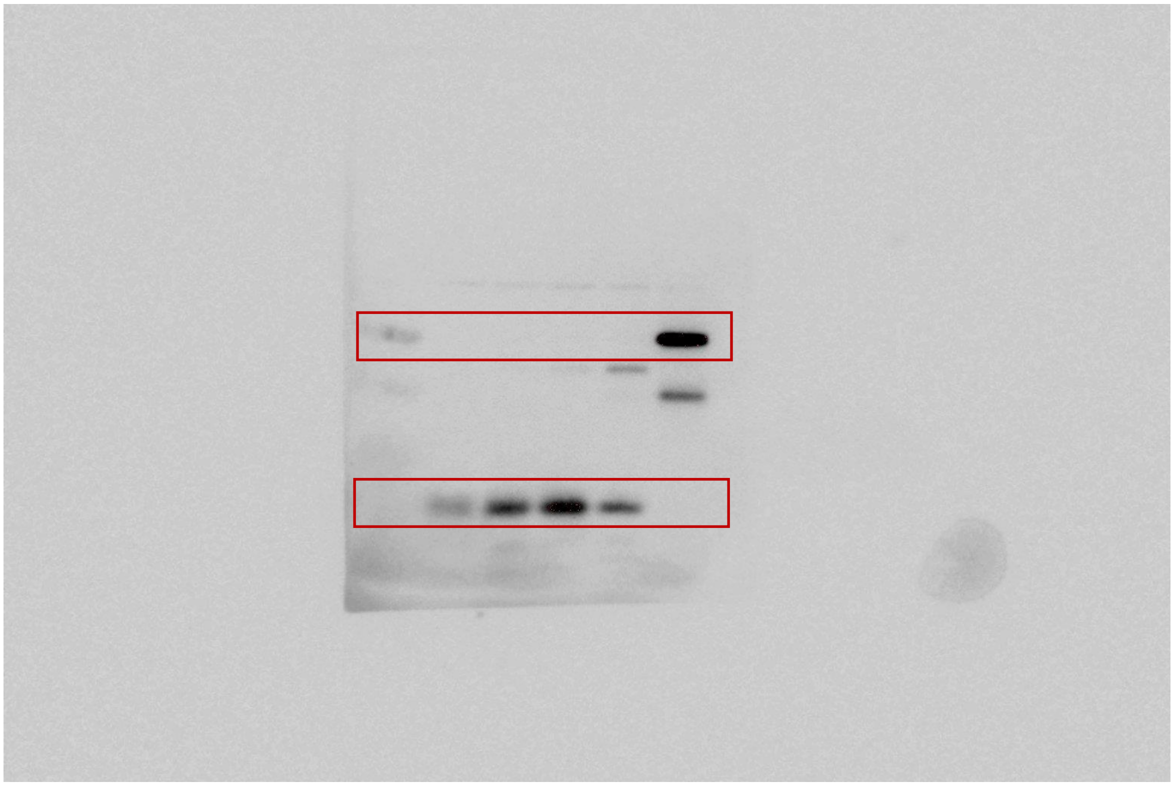

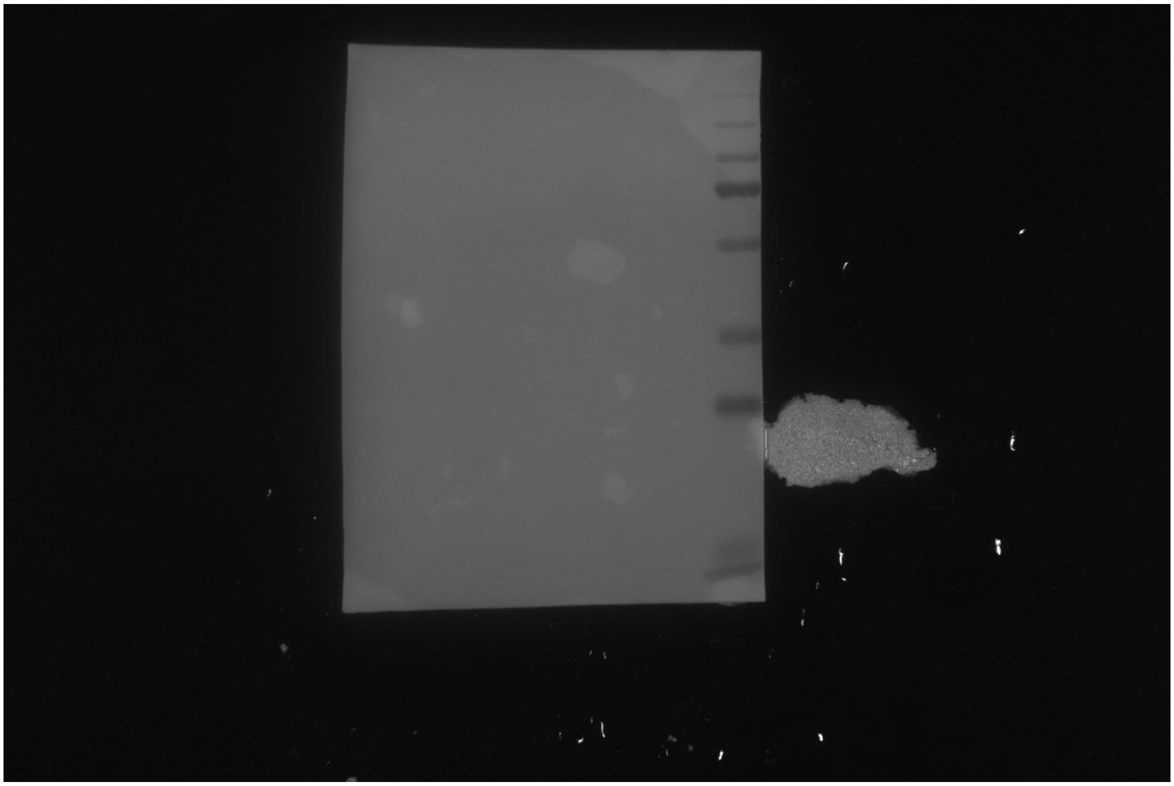

Figure 9B – UW228

GAPDH

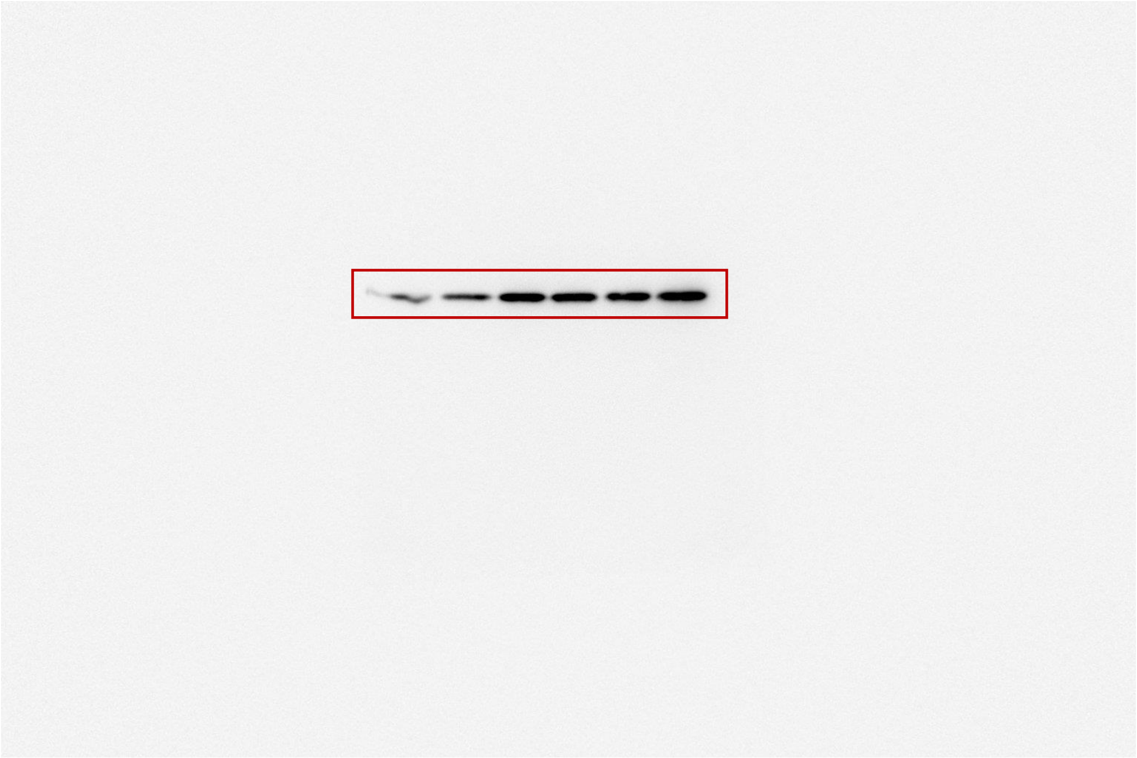

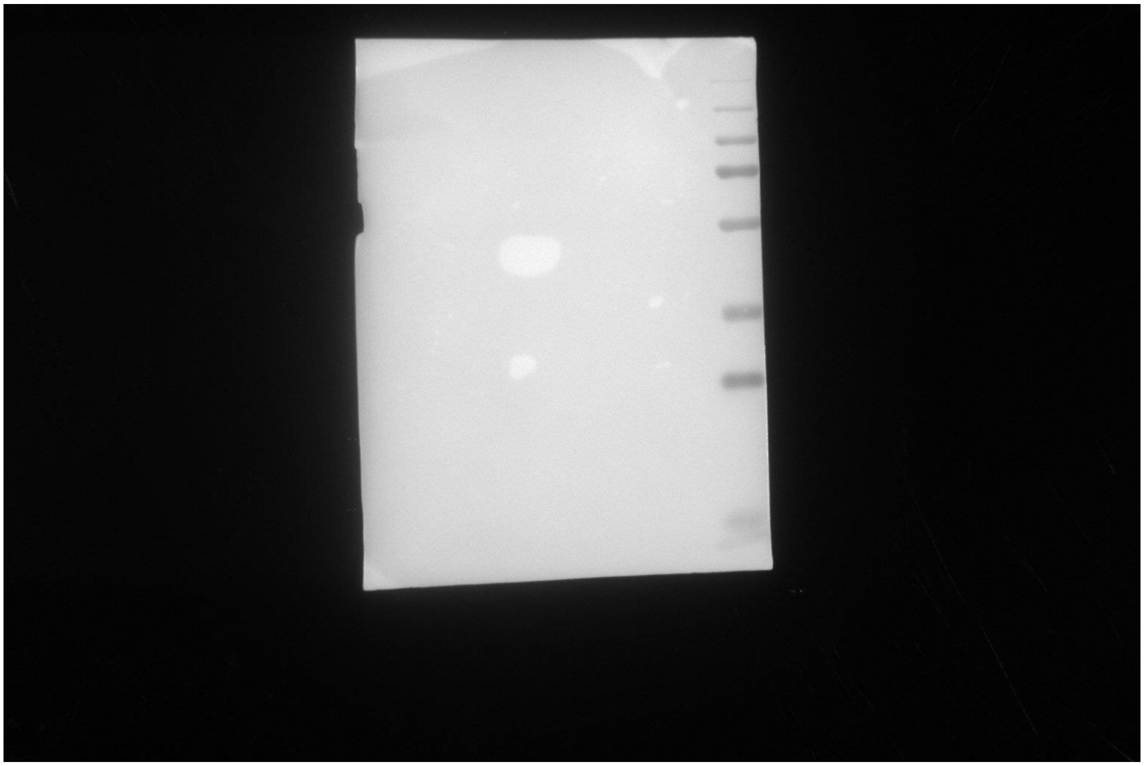

Figure 9B – UW228

Caspase 9/Cleaved caspase 9

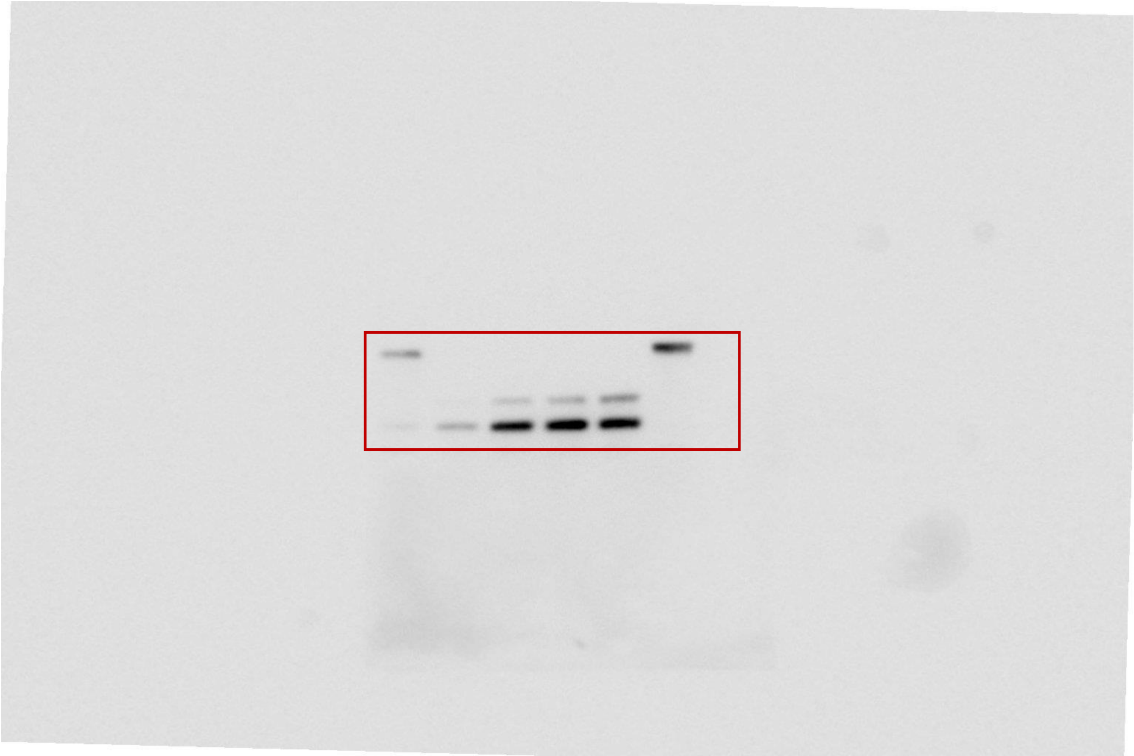

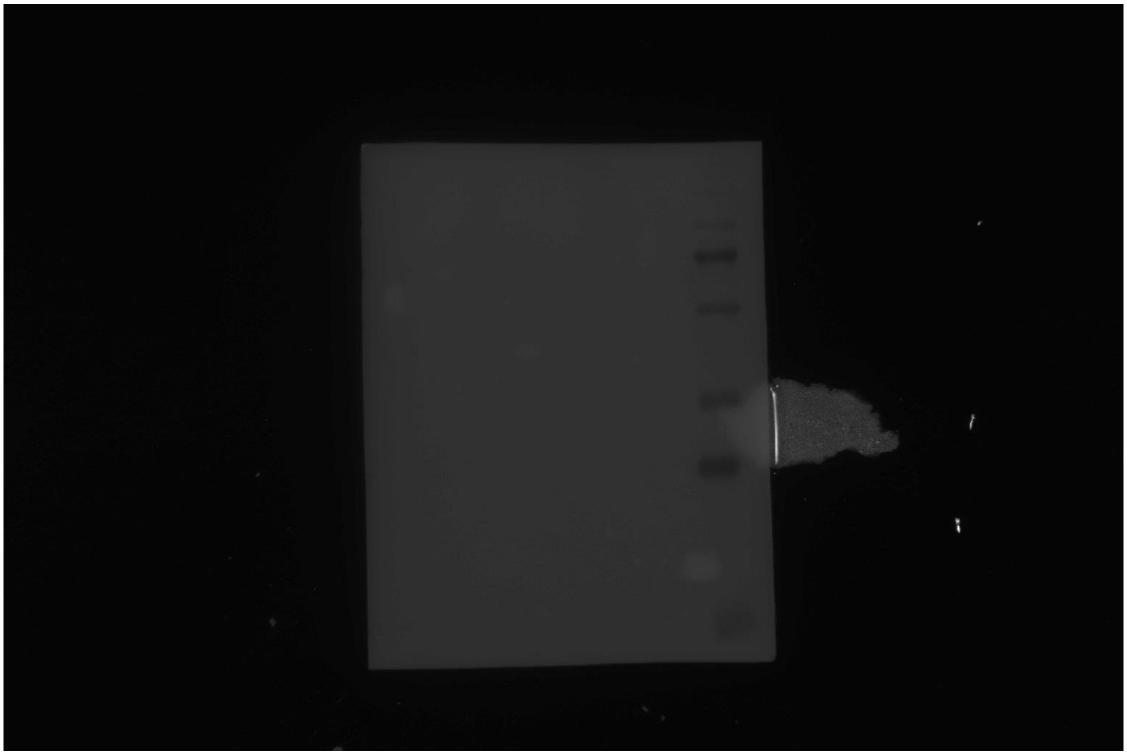

Figure 9B – UW228

GAPDH

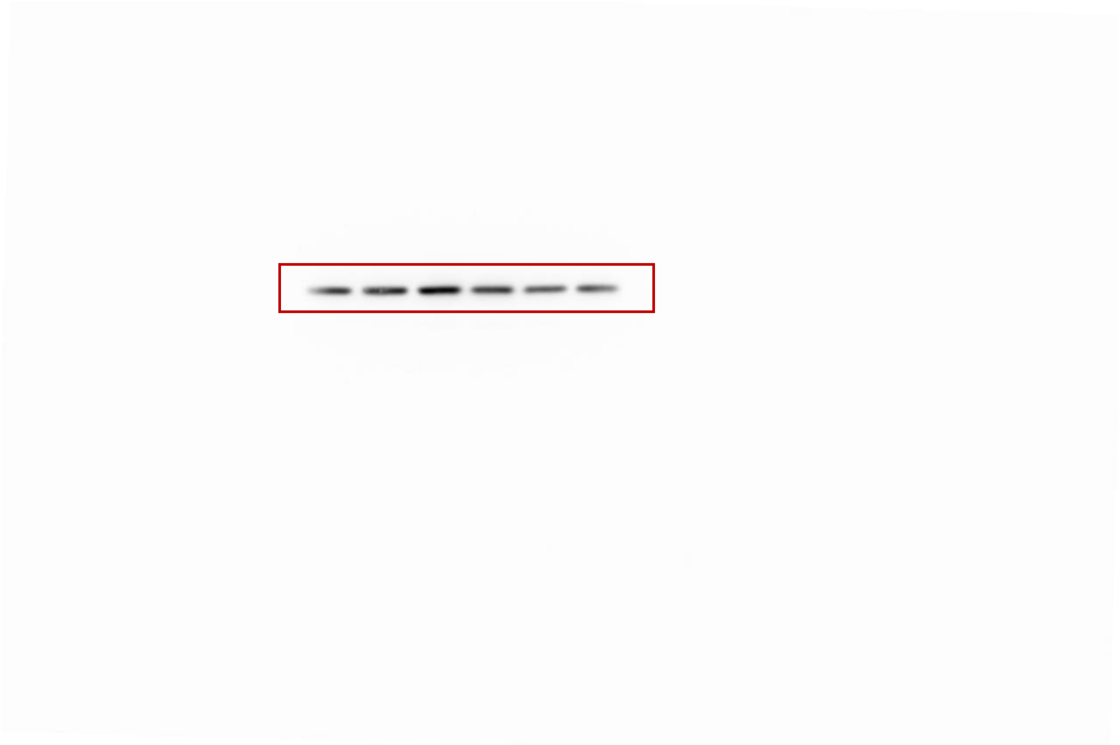

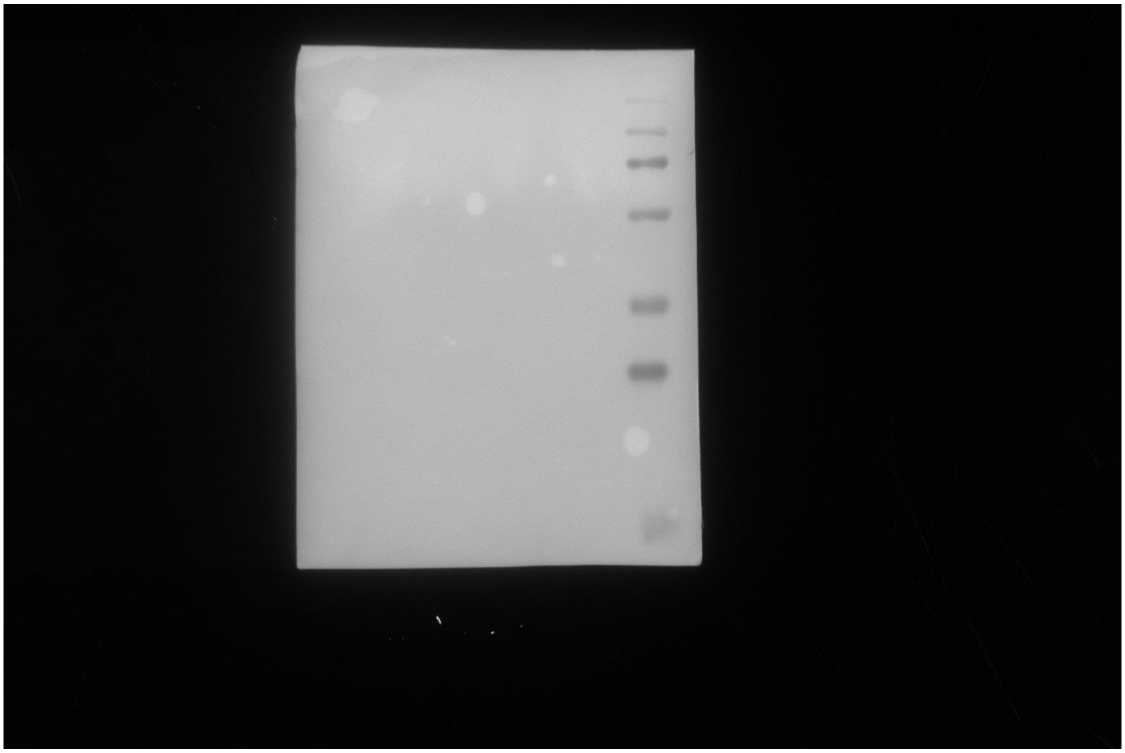

Supplement: Supplementary file 2 — Original Data File [file 41419_2023_6231_MOESM2_ESM.pdf]
